# 3D printed magnesium silicate/β-tricalcium phosphate scaffolds promote coupled osteogenesis and angiogenesis

**DOI:** 10.3389/fbioe.2024.1518145

**Published:** 2025-01-31

**Authors:** Lulu Wang, Mingkui Shen, Zhongxin Tang, Jun Tan, Kuankuan Li, Haijun Ma

**Affiliations:** ^1^ Department of Plastic Surgery, The Third People’s Hospital of Henan Province, Zhengzhou, China; ^2^ Department of Mini-Invasive Spinal Surgery, The Third People’s Hospital of Henan Province, Zhengzhou, China

**Keywords:** magnesium silicate, osteogenesis and angiogenesis, bone tissue engineering, RNA sequencing, bone regeneration

## Abstract

Fabricating bone tissue engineering substitutes with functional activity remains a challenge for bone defect repair requiring coordinated coupling between osteogenesis and angiogenesis. In this research, we evaluated and analyzed magnesium silicate/β-Tricalcium phosphate (MS/β-TCP) scaffold on angiogenesis and bone regeneration *in vitro* and *in vivo*, and the mechanism of its action were described. Achieving magnesium and silicon ions sustained release, 3D printed MS/β-TCP scaffolds possessed appropriate mechanical properties and had excellent biocompatibility that was suitable for osteoblastic MC3T3-E1 cells and human umbilical vein endothelial cells (HUVECs) with proliferation, adhesion, and migration. Combined techniques of Transwell co-culture, we studied the effect of MS/β-TCP scaffold activated cell-level specific regulatory network, which promotes the osteogenic differentiation of MC3T3-E1 and the endothelial formation of HUVEC by significantly up-regulating the expression of related genes and proteins. In addition, RNA sequencing (RNA-seq) revealed MS/β-TCP scaffold plays a dual role in osteogenesis and angiogenesis by activating PI3K/Akt signal pathway, whereas the expression of genes and proteins associated with osteogenesis and angiogenesis was significantly downregulated the PI3K/Akt signaling pathway was inhibited. Additionally, *in vivo* studies showed that MS/β-TCP scaffolds increased the growth of vascular and promoted the bone regeneration at the bone defect sites in rats. In summary, 3D printed MS/β-TCP scaffolds with effectively osteogenic and angiogenic induction will be an ideal bone substitute applied in bone defect repair for clinical application in the future.

## Introduction

At present, bioceramic materials have been widely used for bone transplantation, infection, tumors resection, and congenital defects (Yan et al., 2019; Shen et al., 2022a; Shen et al., 2022b). However, bioceramic materials have some limitations, such as calcium sulfate is absorbed rapidly in the body and its degradation usually occurs before new bone is formed (Giacomino et al., 2019; Haugen et al., 2019). Almost no hydroxyapatite (HA) is absorbed, this will hinder the formation and remodeling of new bone, and lead to poor local stability or permanent stress concentration (Stegen et al., 2019; Xu et al., 2022). Recent research shows that, β-Tricalcium phosphate (β-TCP) is relatively balanced between stent absorption and bone formation (Kusumbe et al., 2014; Li et al., 2019; Khorsandi et al., 2021). It is a good biodegradable ceramic material, which can provide a lot of calcium ions and sulfate ions as well as a scaffold structure for bone regeneration. However, β-TCP is the lack of osteoinduction and osteogenesis, which limits its application. Therefore, the development and application of β-TCP composites have become a research hotspot in recent decades, which use effectively of the excellent properties of other bone repair materials, because these materials biodegradability, bone inductance, osteogenesis, and bone conductivity which could make up for the shortcomings of single β-TCP and give β-TCP more biological and physical properties.

Like other connective tissues, the bone is a highly vascularized connective tissue (Stegen et al., 2019). Blood vessels are crucial to bone regeneration and repair. Previous study has suggested that bone vessels not only act as channels for blood flow and material transport, but also play a key regulatory role in bone genesis and mediate the new concept of “angiogenesis-osteogenesis” coupling (Kusumbe et al., 2014). During the repair of bone fracture, vascularization is prior to ossification (Zhu et al., 2019). There will be an obstacle *in vivo* application due to lack of vascularization within tissue-engineered scaffolds. Hence, further investigations are still needed to develop how angiogenesis will respond to tissue engineering scaffolds, which are vital to vascular remodelling and bone regeneration in bone tissue healing. In theory, ideal implants should not only have enough mechanical strength and wear resistance to match the patients’ autologous bone, but also have excellent ability to maintain angiogenesis and nutrition transmission. 3D printing technology could fabricate internally interconnected porous structures with precise and controllable shapes which provide conditions for the growth of bone vessels (Khorsandi et al., 2021; Ball et al., 2016; Ji et al., 2022). Using 3D printing technology, we could achieve adjustable mechanical properties and precise control of internal and external structures to develop vascularized and osteoinductive tissue engineered bone implants.

Bioactive ions have been found to provide an optimal bone microenvironment at a reasonable cost in numerous studies. Magnesium plays an important role in bone metabolism and growth and development, and is an essential trace element for human body (Reffitt et al., 2003). High concentration of magnesium ions *in vivo* plays an important role in maintaining vascular function (Cooke and Losordo, 2002). Magnesium ions can induce endothelial cells to produce nitric oxide, essentially the same mechanism as VEGF induces angiogenesis (Cooke and Losordo, 2002; Xu et al., 2019). Additionally, magnesium ions deficiency can lead to bone loss and even osteoporosis (Salma-Ancane et al., 2015). Magnesium is clinically used in magnesium phosphate bone cement and several bioglass. In addition, ion doping can improve the micropore porosity and specific surface area of the calcium phosphate scaffolds. Therefore, the apatite layer can be rapidly deposited on the surface of the scaffold (Abed and Moreau, 2009). Magnesium doped with calcium and phosphorus can increase the material density, promote the adhesion of osteoblasts, and increase the secretion of ALP. Therefore, bone repair materials containing magnesium have attracted the attention of many researchers. Alternately, silicon can be used as an active site for calcification in the initial stages of biomineralization, and precipitates hydroxyapatite from electrolyte solutions in the form of hydrated silicic acid in the later period of calcification (Jones et al., 2007). Silicon can effectively stimulate the activity of human cells, for instance promoting the proliferation and differentiation of osteoblast-like cells, the mineralization of osteoblasts, and the osteogenic differentiation of mesenchymal stem cells (Reffitt et al., 2003; Pietak et al., 2007). However, the mechanism of silicon-induced bone regeneration is not completely clear, and silicon could effectively promote the synthesis of the most abundant protein collagen in the bone matrix, and then stabilize the bone proliferation initially. In addition to its potential osteogenic effect, silicon may also have the ability to promote angiogenesis. In calcium silicate material without phosphorus, the dissolved silicon can induce expression of VEGF in human skin fibroblasts (Dashnyam et al., 2017), which in turn upregulates the secretion of endothelial nitric oxide synthase and nitric oxide thus promoting angiogenesis. Hence, an approach to stimulate bone formation that incorporates magnesium and silicon ions into β-TCP scaffold has the potential to be practical and effective.

**SCHEME 1:**
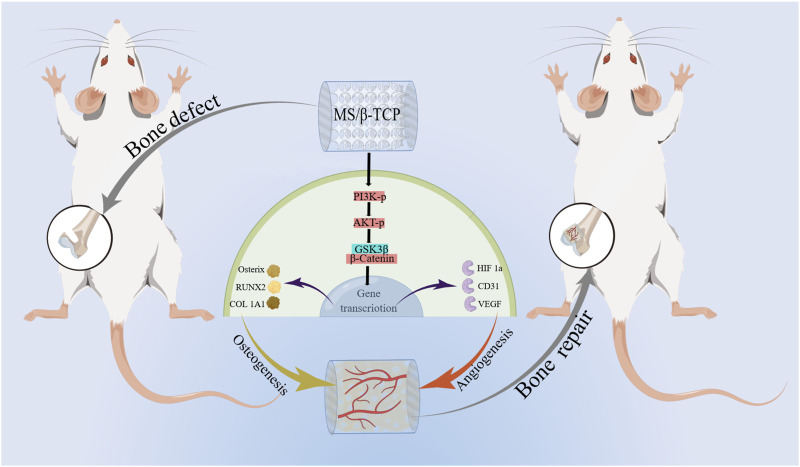
Schematic illustration of magnesium silicate modified β-tricalcium phosphate scaffolds promote coupled osteogenesis and angiogenesis by activating PI3K/Akt signaling pathway for bone defect repairing.

In this research, we developed, designed, and produced MS/β-TCP scaffolds with 3D interconnected multi-stage macroporous structure to repair bone defects (Scheme 1). *In vitro* surface characterization, mechanical properties, and release profile of magnesium and silicon ions among different concentrations of MS/β-TCP scaffolds were investigated. In addition, *in vivo* evaluated biocompatibility and biological function of murine-derived preosteoblasic cell line (MC3T3-E1) and human umbilical endothelial cells (HUVECs) on MS/β-TCP scaffolds. Then, transcriptome sequencing analysis was used to detect differentially expressed genes in biological function and signal pathway which were enriched to reveal the characteristics of symbiosis niches formed by MS/β-TCP scaffolds with different components. Furthermore, *in vivo* the bone repair capacity was investigated by implanting MS/β-TCP scaffolds in rat femur condyle defect and observed the growth of the bone tissue and intraosseous blood vessels. It is expected that the 3D printed MS/β-TCP scaffold could possess excellent biocompatibility, suitable mechanical property, and effectively osteogenic and angiogenic induction simultaneously, which would be an ideal bone substitute applied in bone defect repair for clinical application in the future.

## Materials and methods

### Synthesis of magnesium silicate

The 437 g magnesium sulfate heptahydrate was prepared into a solution with 4% magnesium oxide concentration, and then 1 kg sodium silicate with 3.5 modulus was added to adjust the pH value to 9.1. After the heating and insulation reaction, 2.5 g of each polyacrylamide with 7.5 million and 15 million molecular weights were added as flocculant, and then centrifuged to obtain the wet product magnesium hexasilicate. After adding 0.4 g polyethylene glycol as strength improver, white magnesium silicate (MS) particles were obtained by spray granulation.

### Fabrication of MS/β-TCP scaffolds

Commercial β-tricalcium phosphate (β-TCP) was orderd from Zhongke Leiming Technology Co., LTD (Beijing, China). The β-TCP powders containing different proportions of MS particles (5, 10, 15 wt%) by physical mixing were used to prepare the printable inks. The scaffold model which was a 3 mm diameter cylinder with 3D interconnected multi-stage macroporous structure, was designed by using General CAD software ANSYS SpaceClaim 2021 R2 (ANSYS Workbench, United States). X-CUBE 3D printer (Steke Technology Co., Ltd., Foshan, China) was used to fabricated the β-TCP, 5MS/β-TCP, 10MS/β-TCP, and 15MS/β-TCP scaffolds with an aperture of 0.5 mm, and then calcined in a muffle furnace at 1,100°C for 2 h.

### Scaffold characterization

The surface morphology of different scaffolds at source voltage of 10 kV and magnification ranging from ×100 and ×20,000 was observed by scanning electron microscope (SEM) (FEI-F50, ZEISS, Germany). Element composition distribution was detected using an energy-dispersive X-ray spectrometer (EDS) (Octane SDD, EDAX, United States) at source voltage of 20 keV and magnification at ×100. The electron beam diameter was 0.8 nm, and the vacuum pressure was 4.45 × 10^−4^ Pa. The micropore structure and roughness changes were measured by atomic force microscope (AFM) (Dimension Edge, Bruker, United States).

Electronic universal testing machine (ZwickRoell, Germany) was measured the compressive strength of scaffolds. We placed the scaffold vertically between two parallel fixtures, and then pressed down at a speed of 1 mm/min through a 20 kN load cell until it cracked. The load size of the specimen at fracture was considered as the maximum compressive strength.

### Release of magnesium and silicon ions

To evaluate the release of magnesium and silicon ions, 5MS/β-TCP, 10MS/β-TCP, and 15MS/β-TCP scaffolds were immersed in simulated body fluids (SBF) with the extraction ratio of 1.25 cm^2^/mL according to the previous research results (Song et al., 2020). The preparation method of the stent extract was referred to the national standard GB/T16886.13-2005 (ISO10993-12:2002). The scaffolds were placed in incubators at 37°C, and the SBF was replaced at 1, 5, 10, 15, 20, 25 and 27 days, respectively. The corresponding extracts were collected and frozen at −80°C. Then the concentrations of magnesium and silicon ions were measured by an inductively coupled plasma mass spectrometer (7700X, Agilent, United States).

### Cell culture

The human-derived cell line HUVECs and the murine-derived preosteoblasic cell line MC3T3-E1 were bought from the China Center for Type Culture Collection. HUVECs were cultured in endothelial cell medium (ScienCell, United States). 90% Dulbecco’s modified Eagle’s medium (DMEM) (Gibco, United States) containing 10% fetal bovine serum (FBS) (Gibco, United States) and 1% penicillin-streptomycin (Gibco, United States) was used to culture MC3T3-E1 cells. HUVECs and MC3T3-E1 cells had the same culture method which in a cell incubator at 37°C with 5% CO_2_, and were passaged at 80% confluency using for *in vitro* experiments.

### Cell proliferation, viability and adhesion on the scaffolds

We used the technique of Transwell co-culture to investigated the proliferation, viability, and adhesion properties of MC3T3-E1 cells or HUVECs on the scaffolds. The 0MS/β-TCP, 5MS/β-TCP, 10MS/β-TCP, and 15MS/β-TCP scaffolds were immersed in 1 mL of standard DMEM media or endothelial cell medium in the lower chambers, while HUVECs or MC3T3-E1 cells were seeded in the upper chambers. Then MC3T3-E1 cells or HUVECs were seeded on the surface of scaffolds with a density of 5 × 10^4^ cells/L in the 24-well plates, and incubated for 1, 3, 5, and 7 days at 37°C with 5% CO_2_ in a cell incubator. At testing time point, 100 μL of CCK8 reagents (Abbkine, China) was added to each well and incubated for 1 h, and then the absorbance was measured at a wavelength of 450 nm using a UV-Vis spectrophotometer.

To evaluate the biocompatibility of scaffolds, the viability of MC3T3-E1 cells or HUVECs was analyzed using live and dead cell staining. Briefly, the composite scaffold containing cells was stained by a Live/Dead Cell Imaging kit (Invitrogen, United States) after 3 days of co-culture as described above. The samples were then examined on a confocal fluorescence microscope (Nikon, Japan) and representative images were obtained. In addition, the cells on scaffold were fixed with 4% paraformaldehyde (PFA) for 20 min, and then stained with AbFluor 488-Phalloidin (Abbkine, China) for MC3T3-E1 cells or AbFluor 594-Phalloidin (Abbkine, China) for HUVECs for 30 min, and stained with DAPI (Abbkine, China) for 5 min. The adhesion properties of MC3T3-E1 cells and HUVECs on the scaffold were detected under confocal fluorescence microscopy, and representative images were obtained.

### Wound healing assay

MC3T3-E1 cells and HUVECs were inoculated in 6-well plates of the co-culture system. When cell confluence reached 95%, straight lines were scratched across the middle of the cell monolayer using a SPLScar scrather in each well. After washing with PBS, the serum-free medium was used to culture cells. Digital images of the wounds were acquired using a microscope at the time point of 24 h or 48 h. Image-J software was used to calculate the scratch area in the images. The migration of MC3T3-E1 cells or HUVECs was calculated as follows:

$${\text{Migration}}_{\text{MC}3T3‐E1}\;\left(\%\right)=\frac{0\;h\;wound\;area\;–\;24\;h\;wound\;area}{0\;h\;wound\;area}\times 100\%$$

$${\text{Migration}}_{\text{HUVECs}}\;\left(\%\right)=\frac{0\;h\;wound\;area\;–\;48\;h\;wound\;area}{0\;h\;wound\;area}\times 100\%$$

### Transwell migration assay

For the transwell migration assay, MC3T3-E1 cells or HUVECs were inoculated in the upper chambers with 1 × 10^5^ cells and cultured with 200 μL of serum-free medium. 1 mL of complete DMED medium or endothelial cell medium and scaffolds were added to the lower chamber. In addition, MC3T3-E1 cells or HUVECs were seeded in the lower chambers with 1 × 10^5^ cells as the control group. After culture for 24 h, cells in the upper chamber were removed, and invasive cells embedded in the membrane of the transwells were fixed with 4% PFA for 20 min, and stained with crystal violet (Solarbio, China) for 20 min. Digital images were acquired using a microscope. Moreover, the stained samples were immersed in 10% acetic acid for 10 min, and absorbance of the supernatant was measured at a wavelength of 570 nm using a UV-Vis spectrophotometer. The mean cell number in the control group was set as 1 for determination of the relative cell number. ImageJ software was used to analyze the results.

### *In vitro* osteogenesis evaluation

#### Alkaline phosphatase (ALP) activity and staining

Quantitative determination of ALP activity and ALP staining were used to evaluate the osteogenic induction performance of the scaffold. Briefly, MC3T3-E1 cells were seeded in the lower chambers cultured with complete DMED medium, and HUVECs were inoculated in the lower chambers cultured with endothelial cell culture medium. When cell confluence reached approximately 90%, the culture medium of MC3T3-E1 cells was replaced with the extract of the scaffolds immersed in osteogenic medium (Cyagen, China), and changed the medium every 3 days. On days 7 and 14 of osteogenesis induction, MC3T3-E1 cells were lysed with 0.1% Triton X-100 (Solarbio, China) to release ALP, which was detected by an ALP assay kit (Beyotime, China). Osteogenic induction samples were also stained with an alkaline phosphatase staining kit (Beyotime, China) and digital images were obtained using a microscope.

#### Alizarin Red S (ARS) staining

ARS staining was used to detect osteogenic induced mineralized nodules. After osteogenic induction performed as described above for ALP detection for 14 and 21 days, MC3T3-E1 cells were fixed with 4% PFA for 20 min, and stained with Alizarin Red dye stain solution (Cyagen, China) for 10 min. After washing with PBS, the culture plates were placed under microscope to observe the effect of osteogenic staining. Subsequently, the mineral deposits were dissolved in 10 mM cetylpyridine chloride and quantified (Macklin, China), and the optical density (OD) was measured at 570 nm using a UV-Vis spectrophotometer.

### *In vitro* angiogenesis evaluation

#### Tube formation assay

Tube formation assay were used for angiogenesis *in vitro*. Briefly, the 96-well plates were placed on the ice, and 60 μL of BD Matrigel (Corning, United States) was added to each well. Then the composite plates were placed in a 37°C cell incubator for 30 min to solidify the matrix gel. HUVECs were seeded in composite 96-well plates with 1 × 10^5^ cells per well and cultured with 50 μL of extract of 0MS/β-TCP, 5MS/β-TCP, 10MS/β-TCP, and 15MS/β-TCP scaffolds immersed in endothelial cell medium. After incubation for 4 h in the cell incubator, the cells were stained with 50 μL of 6.25 μg/mL caclcein-AM (Abbkine, China) for 30 min. After washing with PBS, the digital images of vascular network were captured using the Lionheart FX automated cell viability analyzer (BioTek, United States) and analyzed through ImageJ software.

#### Chorioallantoic membrane (CAM) assay

In order to test the angiogenesis *in vivo* that the chicken CAM assay was performed. Briefly, fresh fertilized eggs (within 1 week of production) with similar weight (50 ± 5 g) and no skin contamination and damage were selected, and wiped with 75% alcohol for disinfection. The fertilized eggs were then placed in an incubator with a relative humidity of nearly 60% at 37.5°C. After hatching on day 7, an approximate 5 mm hole was formed at the top end of the egg, the egg shell membrane was extracted using a sterile ophthalmic forceps, and then 0.5 mL of extract of scaffolds immersed in endothelial cell medium were added and immersed between the egg shell membrane and the CAM. Sterilized clear glue was used to close the holes, and the egg samples continued to be cultured. After 11 days of cultivation, the egg shell and egg shell membrane above the CAM plane were removed with tweezers to expose the CAM, and pictures were captured with a digital camera.

### Real-time polymerase chain reaction (RT-PCR)

In order to verify the effectiveness of scaffolds in promoting osteogenesis and angiogenesis, we further analyzed the expression level of genes (RUNX2, Osterix, COL 1A1) related to osteogenesis and genes (HIF1a, CD31, VEGF) related to angiogenesis by RT-PCR. Briefly, both MC3T3-E1 cells induced into osteogenesis for 7 and 14 days and HUVEC cultured for 3 and 5 days with medium extract of scaffolds in a co-culture system, the total RNAs were extracted with the FastPure Cell/Tissue Total RNA Extraction Kit (Vazyme, China), quantified through an ultramicrobiological detector (Thermo, United States), and used to synthesis complementary DNA (cDNA). The cDNA, primers for osteogenesis and angiogenesis (Table 1), and Taq Pro Universal SYBR qPCR Master Mix (Vazyme, China) into the PCR microplate. RT-PCR was performed using StepOnePlus real-time PCR system (Biosystems, United States). The multiple change of mRNA expression level was calculated by ΔΔCt method. The expression of glyceraldehyde-3-phosphate dehydrogenase (GAPDH) was used to standardize the relative expression level of target genes.

**TABLE 1:** Primers for real-time RT-PCR.

Gene	Forward primer (5′–3′)	Reverse primer (5′–3′)
RUNX2	CAT​GGC​CGG​GAA​TGA​TGA​G	TGT​GAA​GAC​CGT​TAT​GGT​CAA​AGT​G
Oxterix	TGA​CTG​CCT​GCC​TAG​TGT​CTA​CA	TGGATGCCCGCCTTGT
COL 1A1	GAC​ATG​TTC​AGC​TTT​GTG​GAC​CTC	GGG​ACC​CTT​AGG​CCA​TTG​TGT​A
HIF1a	GTC​CCA​GCT​ACG​AAG​TTA​CAG​C	CAG​TGC​AGG​ATA​CAC​AAG​GTT​T
CD31	GAA​CAA​ACT​TGC​AAG​GAG​CAG​GAA	CAC​GGA​GCA​AGA​AAG​ACT​CTG​A
VEGF	GCT​CTC​TTG​GGT​GCA​CTG​GA	CAC​CGC​CTT​GGC​TTG​TCA​CA
GAPDH	GGC​ACA​GTC​AAG​GCT​GAG​AAT​G	ATG​GTG​GTG​AAG​ACG​CCA​GTA

### Western blotting

According to Western blot assay, the expression of osteogenesis and angiogenesis related proteins was analyzed. After both MC3T3-E1 cells induced into osteogenesis for 14 days and HUVEC cultured for 5 days with medium extract of scaffolds in a co-culture system, total proteins were extracted from the MC3T3-E1 cells and HUVECs on the scaffoldsusing RIPA lysis buffer (Epizyme, China), and quantified by BCA Protein Assay Kit (Abbkine, China). Protein samples from each group were separated on SDS-PAGE gels (GenScript, China) and transferred to methanol-activated polyvinylidene fluoride (PVDF) membranes (Millipore, United States). The PVDF membranes were blocked with 5% w/v skim milk (BD, United States), and then incubated with primary antibodies against RUNX2, Osterix, COL 1A1, HIF 1a, CD31, VEGF, β-Catenin, GSK 3β, AKT, PI3K, and GAPDH overnight at 4°C. The next day, the samples were washed with TBST and then incubated with secondary antibodies at room temperature. The results showed that the sample bands were visualized using a chemiluminescence kit (Abbkine, China) on an automatic chemiluminescence system (Sage Creation, China) and representative images were obtained.

### Ribonucleic acid sequencing (RNA-seq) analysis

RNA-Seq was used to analysis the mechanism and differentially expressed genes related to osteogenesis and angiogenesis between β-TCP, and 10MS/β-TCP scaffolds on MC3T3-E1 cells and HUVECs. Briefly, the extraction of RNA samples was based on the RT-PCR process. The next, the RNA-Seq was conducted in Annoroad Gene Technology Anoroad Inc. (Annoroad, China) based on Illumina sequencing platform. A genetic screening protocol of differentially expressed genes (DEGs) was defined as |log2 Fold change| ≥ 1 and q < 0.05. Mapping and enrichment analysis were performed as previously described (Shen et al., 2022a). The volcano map, Gene Ontology (GO) statistics, and enrichment of Kyoto Encyclopedia of Genes and Genomes (KEGG) of DEGs were drawn according to the upregulated genes in each comparison group.

### Animal experiment

*In vivo* the osteogenic capability of 3D printed MS/β-TCP scaffolds was further evaluated in rat femur condyle defect model. All experimental protocols were permitted by the Animal Care Committee of Southern University of Science and Technology (Project Number: SUSTech-JY2020215), and all operations were performed by the same surgeon. Eight-week-old Sprague-Dawley rats (male, weighting approximately 250 g) were purchased from Guangdong Medical Laboratory Animal Center, and fed for 1 week before the start of the experiment. In total, thirty-six rats were divided into equally divided into three groups: blank group, β-TCP group, and 10MS/β-TCP group. Briefly, the rats were secured to the operating table in a prone position under inhalation anesthesia. After shaving and disinfecting the site of left femoral condyle, a 1.5 cm incision was made in the skin and the femoral condyle was exposed by separating the subcutaneous tissue. A 3 mm femur condyle defect was produced using an electric drill with a diameter of 3 mm and rinsed with saline throughout the process. After removing the bone, the prepared scaffold was randomly implanted into the defect site. The wound was closed with 3-0 silk thread. To prevent wound infection after operation, 80,000 units of penicillin per rat were intramuscular injected for 3 days.

### Imaging and histological evaluation

#### Micro-computed tomography (micro-CT) analysis

In order to evaluate the bone repair *in vivo*, the rat femurs were harvested and fixed in 4% PFA for 48 h, and then scanned at 4, 8, and 12 weeks using a micro-CT (SkyScan1276, Bruker, Belgium) at source voltage of 70 kV and electricity of 100 μA with a 0.4° of rotation step. Regions of interest (ROI) were selected to distinguish scaffolds and new bone tissue, and then bone volume/total volume (BV/TV), bone mineral density (BMD), and trabecular number (TbN) were calculated using the CTAn software. In addition, the data of ROI were used to reconstitute the 3D image through the CTvox software to better visualize bone repair in the defect area.

#### Histologic and immunofluorescence analysis

For evaluation of bone histology, the harvested rat femurs were subsequently decalcified with EDTA decalcification fluid (OKA, China) for 8 weeks. To evaluate the biological safety of MS/β-TCP scaffold *in vivo* also includes heart, liver, spleen, lung, and kidney organs, which are fixed with 4% PFA. Then those samples were dehydrated and embedded in paraffin, and 5 μm thick sections were made to be used for histologic and immunofluorescence staining. After dewaxing and dehydration, the sections were stained with hematoxylin and eosin (HE) (Baso, China). In addition, the bone tissue sections were stained with Masson Trichome (Solarbio, China) to observe the formation of bone collagen fibrils. For immunostaining to observed the bone neovascularization, the bone tissue sections were dewaxed, underwent antigen repair in citric acid solution, blocked with 5% bovine serum albumin (BSA) (China Biotech), and then incubated with primary antibody (CD31 and vWF) at 4°C overnight. The next day, rinse the sample with PBS, and incubate it with the second antibody for 1 h, and then stained with stained with DAPI for 5 min at room temperature. After resin sealing, the samples were visualized using a confocal fluorescence microscope and representative images were acquired.

### Statistical analysis

The data obtained are shown as mean ± standard deviation (SD). Use the GraphPad Prism program (GraphPad Prism 9.0, GraphPad) to conduct statistical analysis of the data. The differences between the groups were analyzed by single factor analysis of variance (ANOVA) and paired Tukey post-mortem analysis. The data between two groups were statistically analyzed by Student’s t-test. P value <0.05 was considered statistically significant.

## Results and discussion

### Fabrication and characterization of scaffolds

**FIGURE 1:**
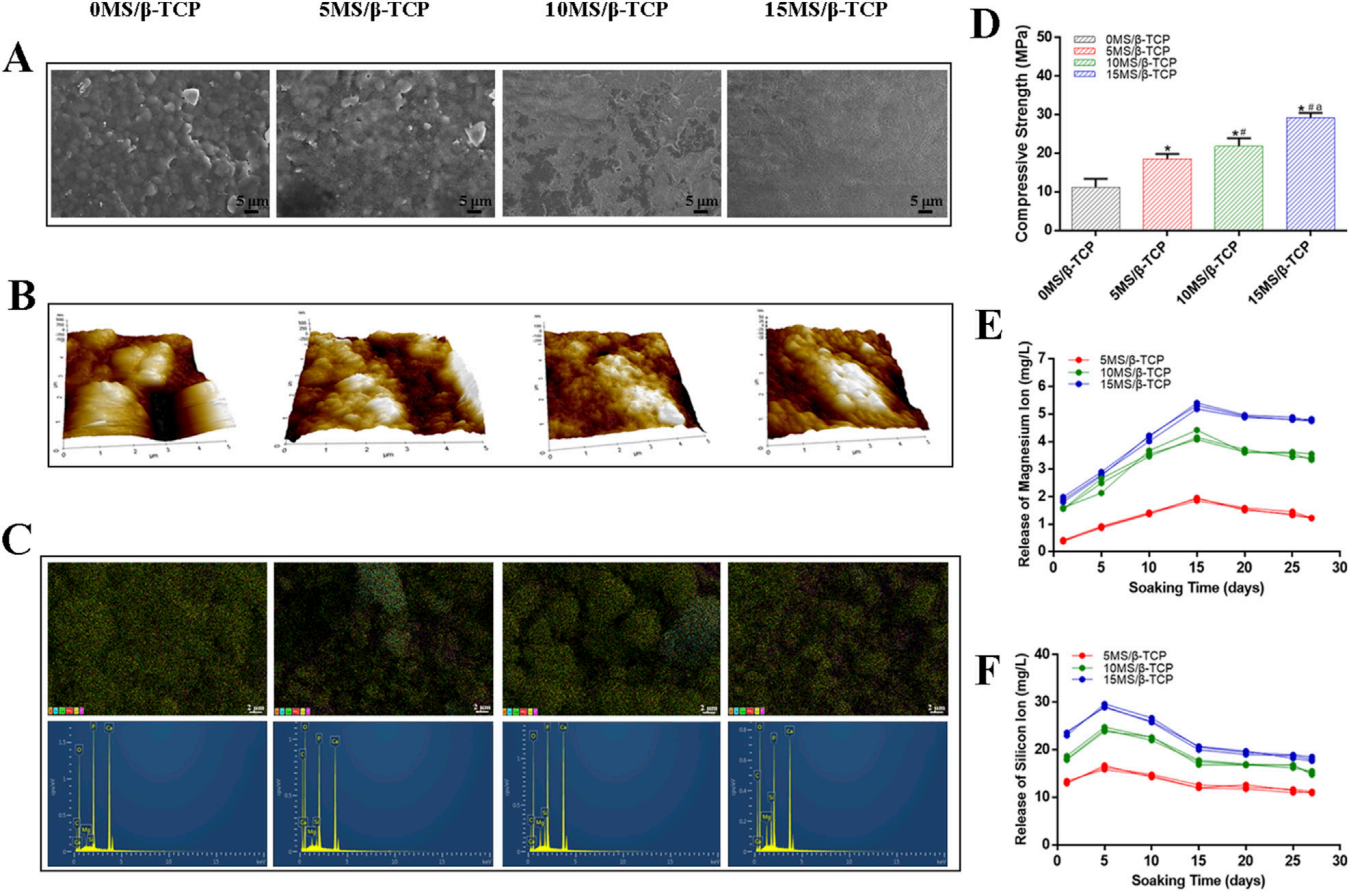
Characterization of the scaffolds. **(A)** Field emission scanning electron microscope (FE-SEM) and **(B)** Atomic force microscope (AFM) photographs of the surface of scaffolds. **(C)** EDS element distribution and element characteristic peak. **(D)** Compressive strength analysis of scaffolds. *p < 0.05 versus 0MS/β-TCP group, ^#^p < 0.05 versus 5MS/β-TCP group, ^a^p <0.05 versus 10MS/β-TCP. The profiles of cumulative magnesium ions **(E)** and silicon ion **(F)** release from scaffolds.

The surface morphology of scaffolds from different groups was shown in Figure 1A. The surface roughness increased with the increase of magnesium silicate content. The pore structure of the surface structure and the macroscopic pore structure could facilitate cell migration and adhesion. Previous studies have shown that material composition, surface morphology, surface chemistry (wettability) and microstructure (porosity, interconnection, pore shape, pore size) significantly affect cell adhesion on the surface of biomaterials (Zhao et al., 2020; Sun et al., 2020; Miao et al., 2022). In addition, the result of AFM images (Figure 1B) was consistent with the SEM images, demonstrating that the 10MS/β-TCP and 15MS/β-TCP scaffolds were more likely to adhere to cells. EDS analysis of different scaffolds was performed by point scanning. The sampling depth was approximately 1 μm to detect microzone elements at a certain location. The surface chemical element composition distribution of different scaffolds was shown in Figure 1C. The proportion of main chemical components on the surface of scaffolds was shown in Table 2. The β-TCP scaffold mainly contained C, O, P, and Ca elements. The characteristic peaks of Si and Mg appeared in the three groups of scaffoldings doped with magnesium silicate, and uniformly distributed in the scaffolds. With the increase of magnesium silicate doping concentration in the β-TCP scaffold, the content of Si and Mg elements also showed a gradual increase trend.

**TABLE 2:** Surface element composition distribution of scaffolds in different groups.

Element (%)	0MS/β-TCP	5MS/β-TCP	10MS/β-TCP	15MS/β-TCP
C	22.90 ± 5.22	39.01 ± 1.56	25.13 ± 5.27	31.63 ± 4.75
O	52.89 ± 2.38	44.63 ± 1.91	50.45 ± 1.93	46.74 ± 2.97
Mg	0.07 ± 0.13	0.27 ± 0.21	1.45 ± 0.12	1.68 ± 0.21
Si	0.04 ± 0.05	0.17 ± 0.20	1.63 ± 0.11	2.31 ± 0.14
P	8.96 ± 1.72	5.48 ± 0.27	8.49 ± 1.02	6.22 ± 0.46
Ca	15.18 ± 2.39	10.43 ± 1.20	12.83 ± 2.38	11.43 ± 1.05
Total	100	100	100	100

Representative compressive strength of scaffolds was illustrated in Figure 1D. The compressive modulus of scaffold was 11.18 ± 2.21 MPa, that of 5MS/β-TCP, 10MS/β-TCP, and 15MS/β-TCP scaffold was 18.54 ± 1.26 MPa, 21.82 ± 2.14 MPa, and 29.20 ± 1.28 MPa, respectively. Meanwhile, the compression modulus increased with the increase of zinc silicate mixing ratio (*p* < 0.05). The ion release behavior was shown in Figures 1E, F, it can be found that the MS/β-TCP scaffold can release magnesium ions and silicate ions continuously and stably in simulated body fluids over time by observing the release curve of magnesium ions and silicate ions. Within the first 5 days, the release rate of the silicate ions was faster, and the release rate decreased slowly from 5 to 15 days. Whereas, the release rate of the magnesium ions was faster within the first 15 days, and the release rate decreased slowly from 15 to 20 days. After that, the release rates of the two kinds of ions tended to be stable. The silicate ion release rates of the 5MS/β-TCP, 10MS/β-TCP, and 15MS/β-TCP scaffolds were respectively stable at 11.40 ± 0.28 mg/L, 16.61 ± 0.29 mg/L, 18.21 ± 0.32 mg/L, and the magnesium ion release rates of the 5MS/β-TCP, 10MS/β-TCP, and 15MS/β-TCP scaffolds were respectively stable at 1.38 ± 0.05 mg/L, 3.54 ± 0.07 mg/L, 4.83 ± 0.04 mg/L. This study proved that with the increase of magnesium silicate ratio to 15%, the surface roughness, compression modulus, silicate ions and magnesium ions release of the scaffolds increased under the condition of consistent printing parameters. The increase of surface roughness could promote cell adhesion, and the incorporation of magnesium silicate could improve the strength of β-TCP scaffold and achieve slow release of ions.

### Biocompatibility assessment of scaffolds

**FIGURE 2:**
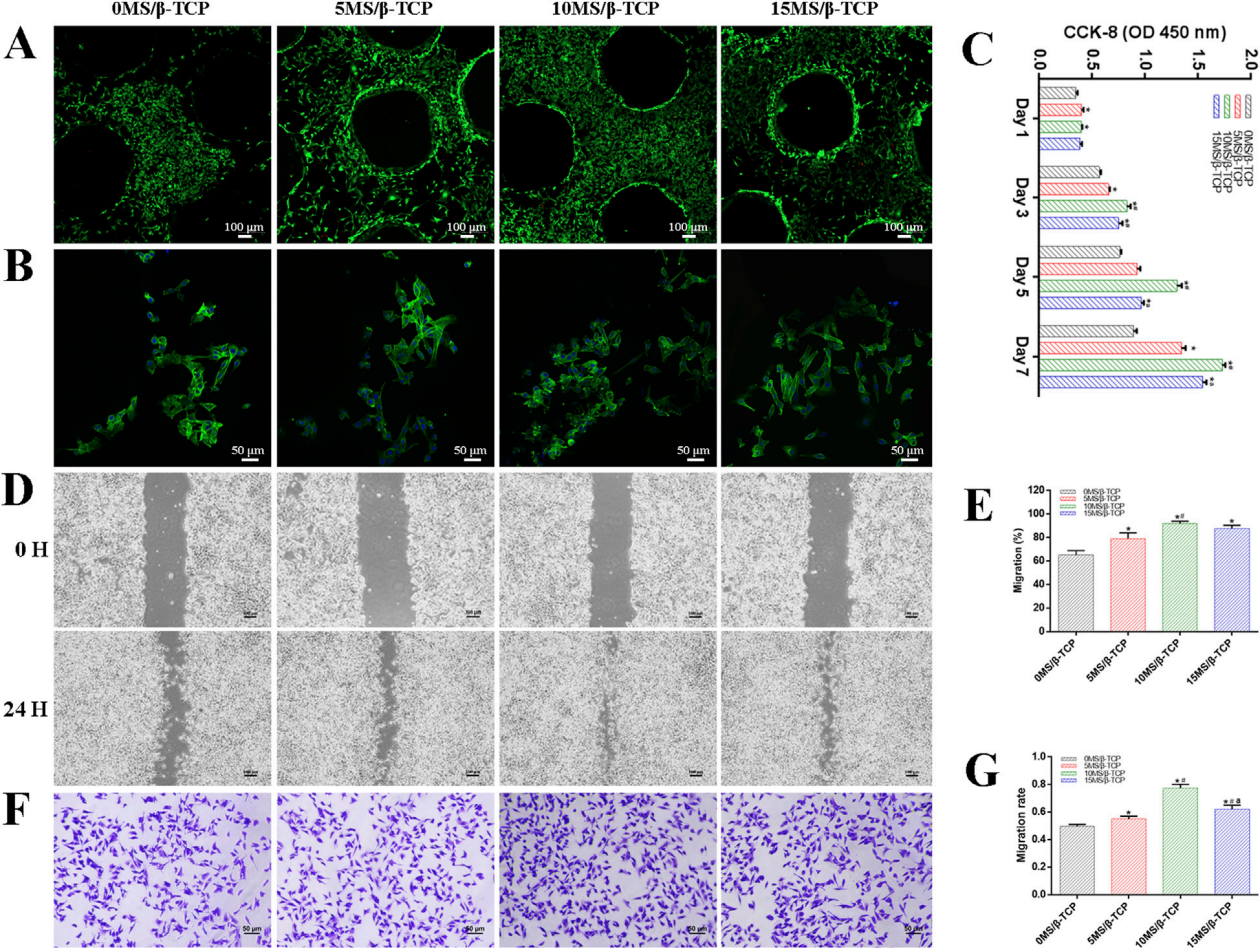
*In vitro* cell biocompatibility of MC3T3-E1 cells with scaffolds. **(A)** Representative images of live/dead staining of MC3T3-E1 cells cultured on scaffolds. Green represented live cells and red represented dead cells. **(B)** Representative images of adhesion properties of MC3T3-E1 cells cultured on scaffolds. Green represented cytoskeleton and blue represented cell nucleus. **(C)** Proliferation of MC3T3-E1 cells cocultured on scaffolds for 1, 3, 5, and 7 days. MC3T3-E1 migration was evaluated by wound-healing assay **(D)** and quantitative analysis **(E)**. MC3T3-E1 migration was evaluated by transwell migration assay **(F)** and quantitative analysis **(G)**. *p < 0.05 versus 0MS/β-TCP group, ^#^p < 0.05 versus 5MS/β-TCP group, ^a^p < 0.05 versus 10MS/β-TCP group.

**FIGURE 3:**
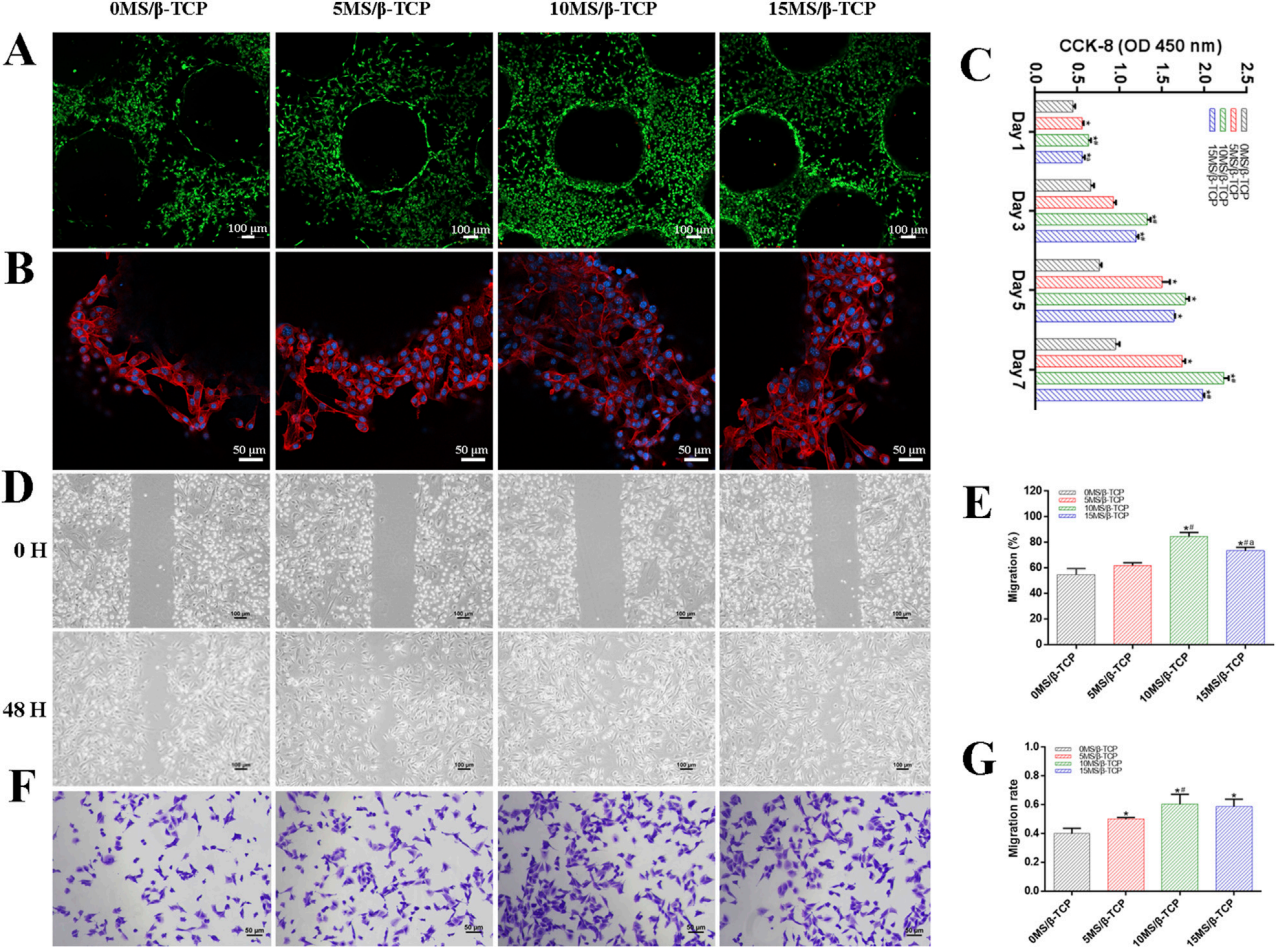
*In vitro* cell biocompatibility of HUVECs with scaffolds. **(A)** Representative images of live/dead staining of HUVECs cultured on scaffolds. Green represented live cells and red represented dead cells. **(B)** Representative images of adhesion properties of HUVECs cultured on scaffolds. Red represented cytoskeleton and blue represented cell nucleus. **(C)** Proliferation of HUVECs cocultured on scaffolds for 1, 3, 5, and 7 days. HUVECs migration was evaluated by wound-healing assay **(D)** and quantitative analysis **(E)**. HUVECs migration was evaluated by transwell migration assay **(F)** and quantitative analysis **(G)**. *p < 0.05 versus 0MS/β-TCP group, ^#^p < 0.05 versus 5MS/β-TCP group, ^a^p <0.05 versus 10MS/β-TCP group.

To evaluate the cell viability of β-TCP, 5MS/β-TCP, 10MS/β-TCP, and 15MS/β-TCP scaffolds, MC3T3-E1 cells and HUVECs were cultured on the scaffolds and REF viability was analyzed by cell viability/cytotoxicity detection kit, respectively. As shown in Figures 2A, 3A, the results demonstrated that all the scaffolds were not statistical differences in cell viability, indicating the incorporation of magnesium silicate in the β-TCP would not generate cytotoxicity. In addition, the number of MC3T3-E1 cells and HUVECs on 10MS/β-TCP scaffolds were significantly higher than those in other three groups for 5 days incubation. Previous studies have shown that silicate ions and magnesium ions affect many cell functions, including signal transduction, energy metabolism, regulation of cell proliferation and differentiation (Staiger et al., 2006; Saris et al., 2000). In addition, the cytotoxicity occurs when the concentration of magnesium ion is higher than 22.8 mg/L (Nabiyouni et al., 2018), but does not occur when the release of silicate ion reaches 95 mg/L (Sun et al., 2017). In this study, the concentrations of silicate and magnesium ions released from the composite scaffold were lower than those reported above. Hence, the fabrication of 10MS/β-TCP scaffolds had good biocompatibility which provided usability for *in vivo* applications in the future.

The adhesion properties of MC3T3-E1 cells and HUVECs were observed by confocal images to detect the interaction between cells and scaffolds. As shown in Figures 2B, 3B, it was obviously observed that the spreading area of MC3T3-E1 cells and HUVECs around the scaffolds were examined by immunofluorescence staining. The results showed that the average spreading area of MC3T3-E1 cells and HUVECs in the 10MS/β-TCP group and 15MS/β-TCP group was larger than that in the β-TCP group and 5MS/β-TCP group. Hence, both MC3T3-E1 cells and HUVECs were more prone to adhere to the 10MS/β-TCP, and 15MS/β-TCP scaffolds than that of β-TCP and 5MS/β-TCP scaffolds, which was in accordance with cell viability assessment.

The proliferation of MC3T3-E1 cells and HUVECs on different scaffolds was evaluated at 1, 3, 5, and 7 days, and quantified results were shown in Figures 2C, 3C. The number of cells increased significantly with the incubation time. Within 7 days of culture, the number of cells in 10MS/β-TCP group was significantly higher than those in other three groups. 5MS/β-TCP and 15MS/β-TCP group showed an obvious increase in cell number and proliferation rate than β-TCP group. The above results indicated that the addition of 5%–15% magnesium silicate could promote the proliferation of MC3T3-E1 cells and HUVECs. In addition, the number of both cells in β-TCP scaffold containing 10% magnesium silicate was the largest. When magnesium silicate was increased to 15%, the number of cells decreased instead of increasing, and the effect of promoting the proliferation of MC3T3-E1 cells and HUVECs was not as significant as that containing 10% magnesium silicate. Thus, 10MS/β-TCP scaffolds were not only conductive to cell adhesion but also promoted cell proliferation.

By wound healing assay and transwell migration assay to evaluate the migration of MC3T3-E1 cells and HUVECs as shown in Figures 2D–G, 3D–G. It was obviously observed that 10MS/β-TCP group performed better cell migration than those other groups. Quantitative analysis of scratch area suggested that the MC3T3-E1 migration in 5MS/β-TCP (79.08% ± 4.07%), 10MS/β-TCP (91.75% ± 1.70%), 15MS/β-TCP (87.72% ± 2.2 3%) groups was noticeably faster than that in β-TCP group (65.08% ± 3.19%), whereas the HUVECs migration in 10MS/β-TCP (84.30% ± 2.76%) and 15MS/β-TCP (73.24% ± 2.26%) groups was significantly faster than that in β-TCP (54.66% ± 3.94%) and 5MS/β-TCP (61.85% ± 1.70%) groups. The transwell assays also showed that the migration of MC3T3-E1 cells and HUVECs was significantly enhanced by 10MS/β-TCP scaffold than that of other groups. In addition, the addition of magnesium silicate in β-TCP scaffolds also increased the migration activity of cells. Previous studies had found that tissue repair might be related to the ability of cell migration to the site of injury (Yan et al., 2019; Nanba et al., 2021). Therefore, 10MS/β-TCP scaffold could effectively promote the ingrowth of new bone tissue by increasing cell migration.

### MS/β-TCP scaffold promotes osteogenesis differentiation *in vitro*

**FIGURE 4:**
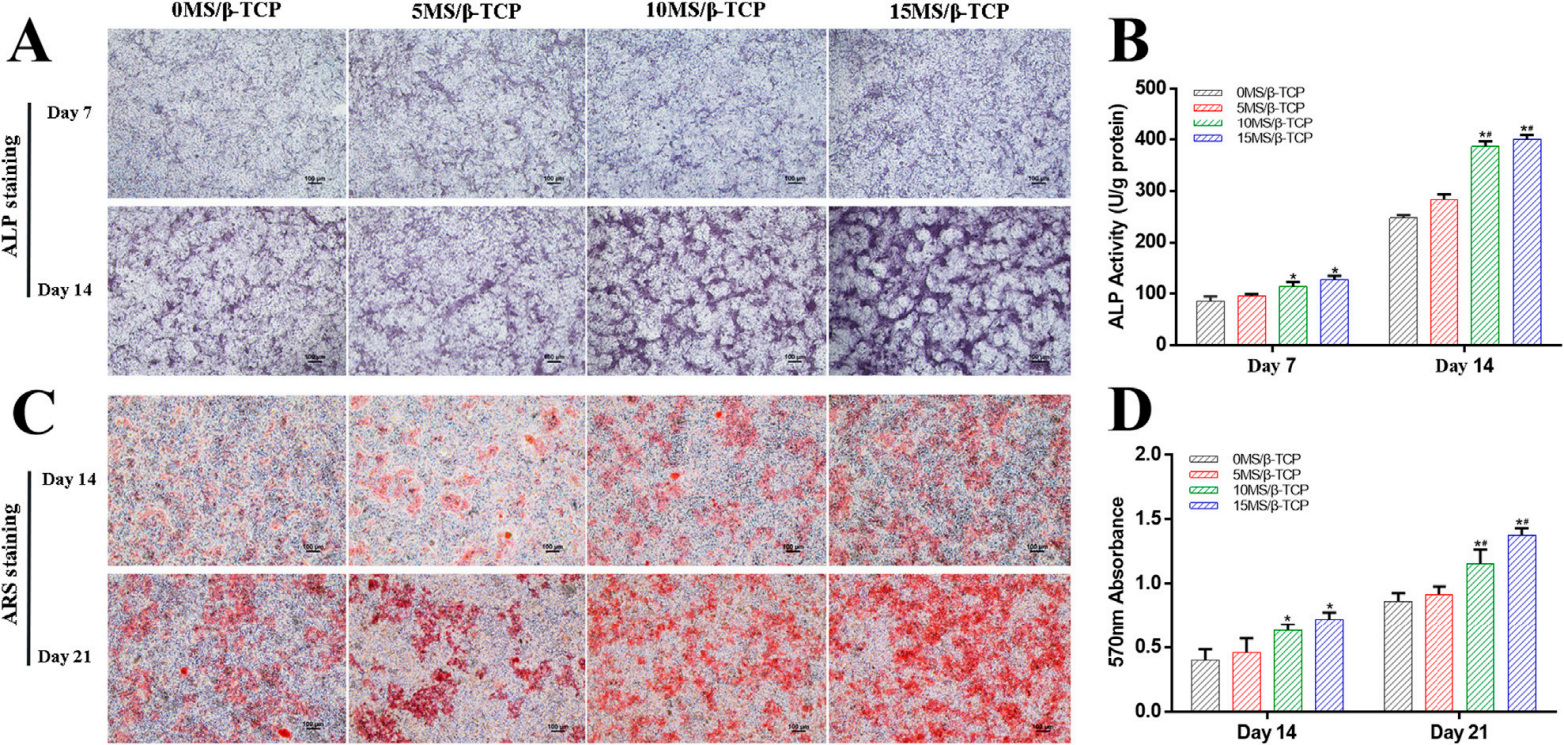
*In vitro* evaluation of osteogenic differentiation of MC3T3-E1 cells cultured with scaffolds. ALP staining **(A)** was used to detect the ALP proteins secreted by MC3T3-E1 cells and ALP activity **(B)** was quantitatively analyzed at 7 and 14 days of osteogenic induction. ARS staining **(C)** was used to detect the mineralized matrix produced by MC3T3-E1 cells and mineralized nodules were quantitatively analyzed **(D)** at 14 and 21 days of osteogenic induction. *p < 0.05 versus 0MS/β-TCP group, ^#^p < 0.05 versus 5MS/β-TCP group, ^a^p < 0.05 versus 10MS/β-TCP group.

MC3T3-E1 cells were induced by osteogenesis with scaffolds extracts, and ALP staining and activity determination were performed on MC3T3-E1 cells at 7 and 14 days after osteogenic induction, and detect the mineralized nodule by Alizarin Red S staining at 14 and 21 days after osteogenic induction. As shown in Figure 4, the results showed that the ALP staining (Figure 4A) and Alizarin Red S staining (Figure 4C) were gradually deepened with the increase of the proportion of magnesium silicate in the β-TCP scaffolds. The relative ALP activity (Figure 4B) and quantitative analysis of mineralized nodules (Figure 4D) both indicated that 10% and 15% magnesium silicate could better promote the ALP activity of MC3T3-E1 cells expression and early osteogenic differentiation than that of 0MS/β-TCP and 5MS/β-TCP groups. Abed reported that magnesium ions stimulated osteoblast proliferation, migration, and differentiation through melastatin-like transient receptor potential 7 channels (Abed and Moreau, 2009). Xing reported that silicon ions stimulated osteogenic differentiation instead of enhancing proliferation (Xing et al., 2018). Magnesium and silicon ions are also related to bone biomineralization, and indirectly affect mineral metabolism and the crystallization process and pattern formation of inorganic mineral phases through the activation of ALP (Saris et al., 2000; Mao et al., 2017). Hence, the MS/β-TCP composite scaffold significantly promoted osteogenic induction through sustained magnesium and silicon ions.

### MS/β-TCP scaffold promotes angiogenesis

**FIGURE 5:**
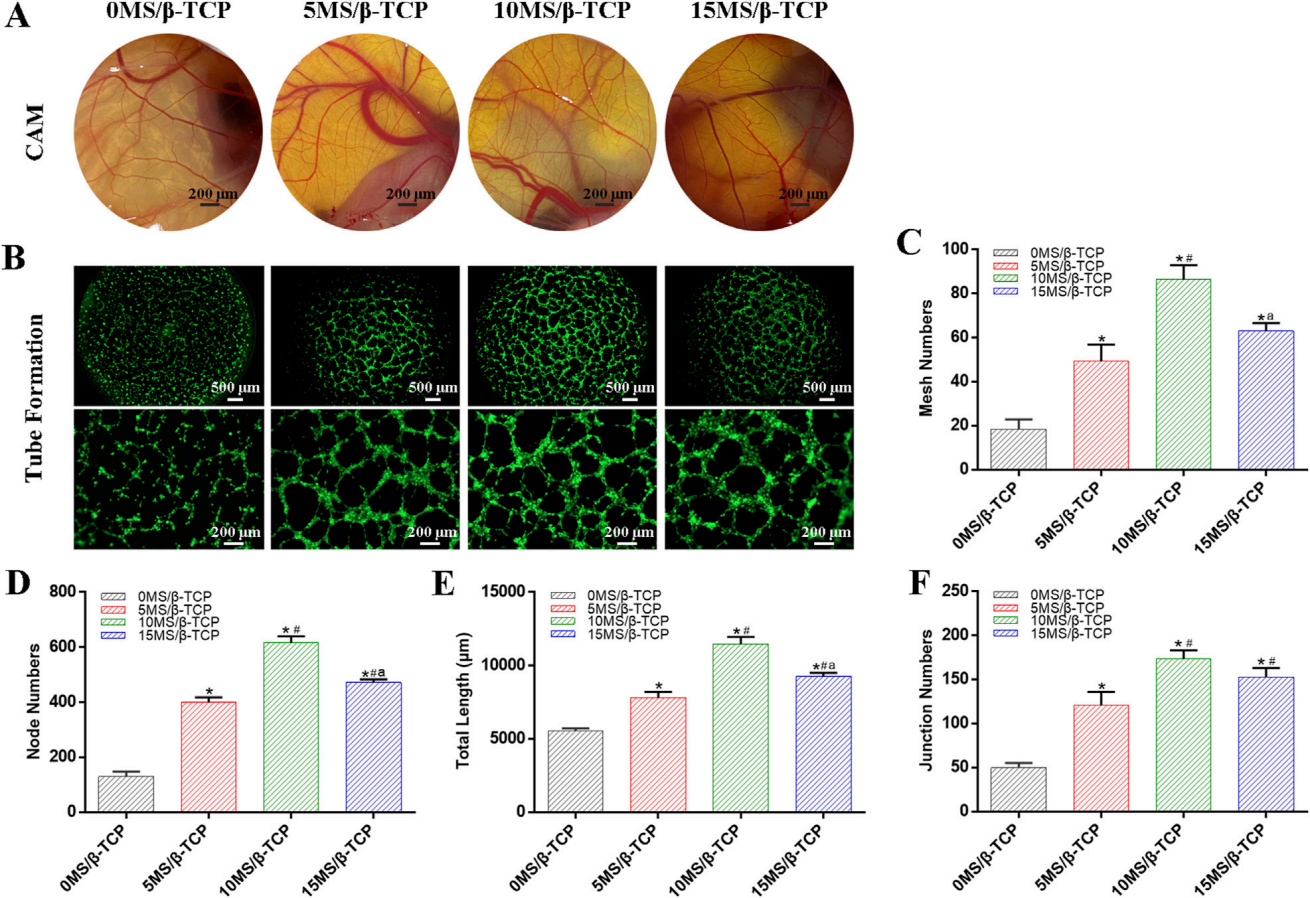
Blood vessel formation of different scaffolds. **(A)** *In vivo* CAM assay. **(B)** *In vitro* tube formation assays of HUVECs. Tube formation was quantified by mesh numbers **(C)**, node numbers **(D)**, total length **(E)**, and junction numbers **(F)**. *p < 0.05 versus 0MS/β-TCP group, ^#^p < 0.05 versus 5MS/β-TCP group, ^a^p < 0.05 versus 10MS/β-TCP group.

In the process of bone development and regeneration, angiogenesis and osteogenesis are closely combined. CAM angiogenesis and tube formation of HUVEC are shown in Figures 5A, B to evaluated the angiogenic induction ability of the 0MS/β-TCP, 5MS/β-TCP, 10MS/β-TCP, and 15MS/β-TCP scaffolds. The results show that compared with the mere β-TCP scaffold, the addition of magnesium silicate in β-TCP scaffolds could effectively promote the formation of vascular networks, and 10MS/β-TCP scaffold had the highest number of vascular networks and capillary vessels in CAM and most effectively induced HUVECs to form vascular contours among the groups.

The quantitative analysis of tube formation was shown that the mesh numbers (Figure 5C), node numbers (Figure 5D), total length (Figure 5E), and junction numbers (Figure 5F) were 18.33 ± 3.68, 129.67 ± 15.33, 5,544.67 ± 146.57 μm, and 49.67 ± 4.64 for the 0MS/β-TCP group, respectively; 49.33 ± 6.13, 400.33 ± 14.88, 7794.33 ± 348.29 μm, and 120.67 ± 12.68 for the 5MS/β-TCP group, respectively; 86.33 ± 5.44, 616.00 ± 19.60, 11,465.67 ± 397.73 μm, and 173.67 ± 7.85 for the 10MS/β-TCP group, respectively; and 63.00 ± 2.94, 472.00 ± 9.63, 9,277.67 ± 185.29 μm, and 153.00 ± 8.29 for the 10MS/β-TCP group, respectively. Previous studies have shown that the appropriate concentration of magnesium ion has confirmed the osteogenic function and promoted the proliferation and vascularization of endothelial cells, which has shown the therapeutic value for bone defect repair (Chen et al., 2022; Zhang et al., 2016a). Those results indicated that 10MS/β-TCP scaffold exhibited a significant angiogenic activity to stimulate the vascular system reconstruction function during bone regeneration.

**FIGURE 6:**
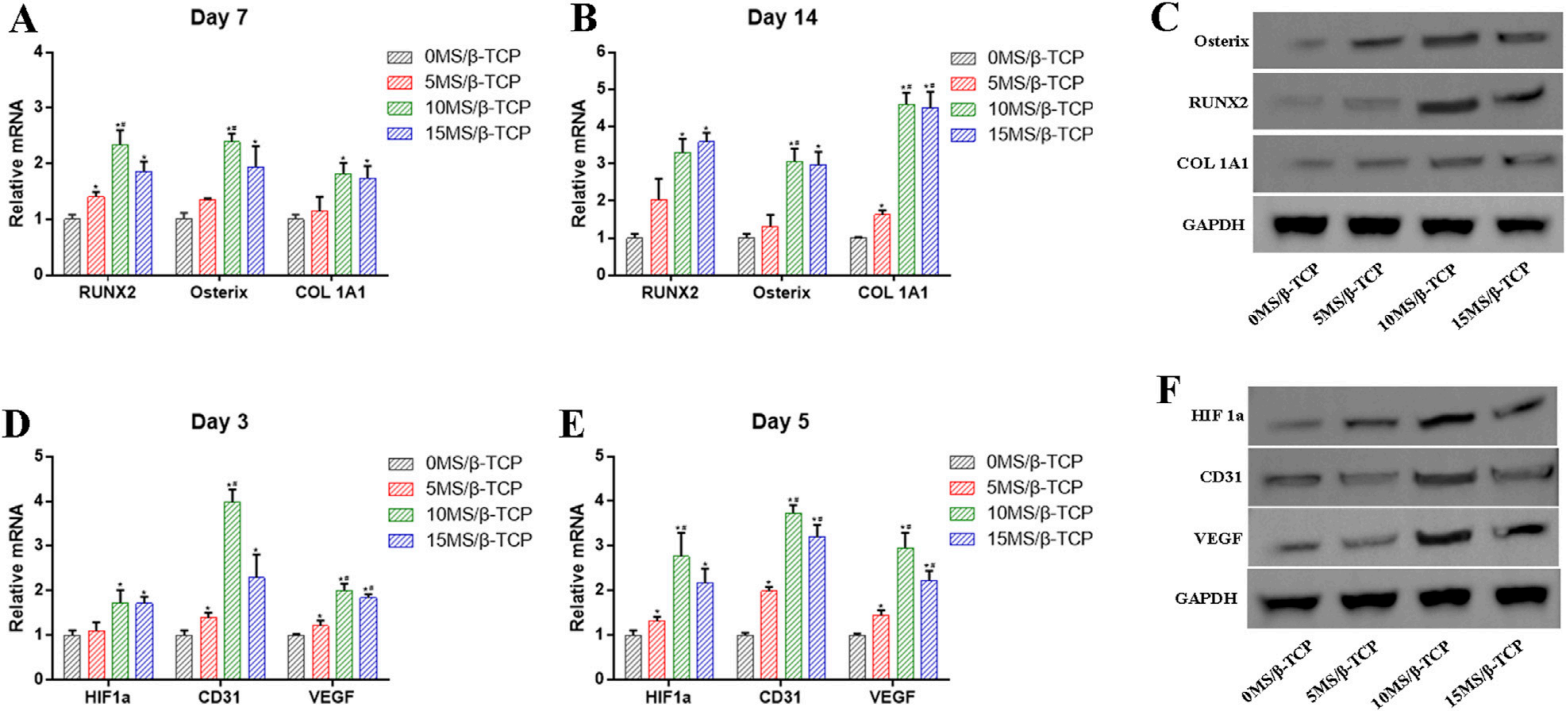
**(A, B)** mRNA levels of genes (RUNX2, Osterix, COL 1A1) related to osteogenesis were detected by RT- PCR at 7 and 14 days of osteogenic induction. **(C)** Protein levels of RUNX2, Osterix, COL 1A1 were examined by Western blot. **(D, E)** mRNA levels of genes (HIF1a, CD31, VEGF) related to angiogenesis were detected by RT- PCR at 3 and 5 days of induction. **(F)** Protein levels of HIF1a, CD31, VEGF were examined by Western blot. *p < 0.05 versus 0MS/β-TCP group, ^#^p < 0.05 versus 5MS/β-TCP group, ^a^p < 0.05 versus 10MS/β-TCP group.

To further prove that the MS/β-TCP scaffold effectively promoted osteogenesis and angiogenesis, the expression levels of related genes and proteins were analyzed by RT-PCR and Western blot assays as shown in Figure 6. The results showed that the mRNA levels of osteogenesis-related RUNX2, Osterix, and COL 1A1 in MC3T3-E1 cells, and angiogenesis-related HIF1a, CD31, and VEGF in HUVECs were significantly higher in 10MS/β-TCP and 15MS/β-TCP groups than that of 0MS/β-TCP group. In addition, Western blot also demonstrated higher protein expression of RUNX2, Osterix, COL 1A1, HIF1a, CD31, and VEGF in 10MS/β-TCP group, which had the same gene expression result as RT-PCR, indicating that MS/β-TCP scaffold played a dual role in osteogenesis and angiogenesis.

### MS/β-TCP scaffold promotes osteogenesis and angiogenesis by activating PI3K/Akt signaling pathway

**FIGURE 7:**
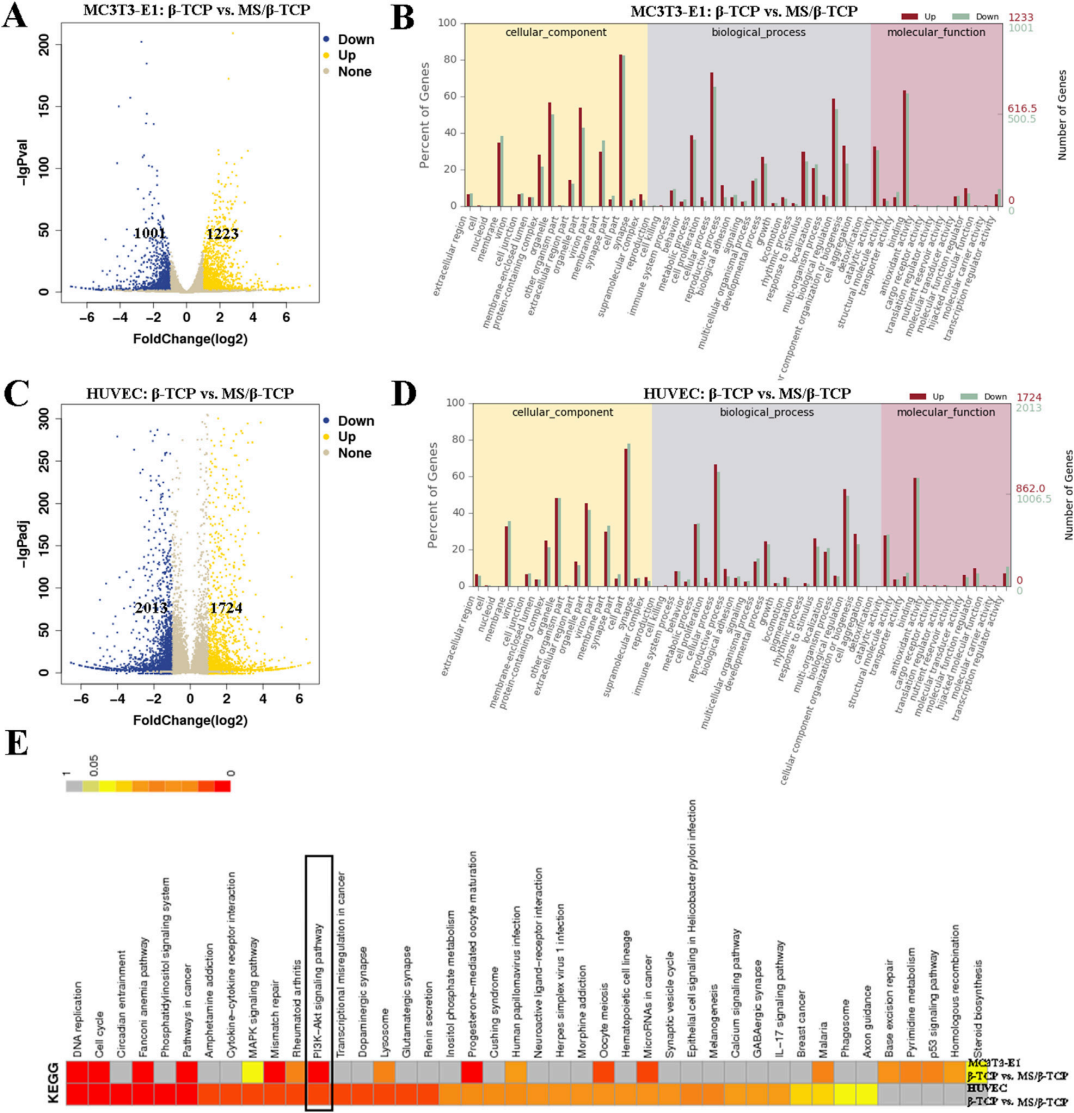
Transcriptome analysis revealed differences in the regulation of cells by scaffolds. Differentially expressed genes **(A)** and Go enrichment **(B)** of MC3T3-E1 cells cultured in β-TCP and 10MS/β-TCP groups. Differentially expressed genes **(C)** and Go enrichment **(D)** of HUVECs cultured in β-TCP and 10MS/β-TCP groups. **(E)** KEGG pathway gene enrichment analysis was carried out to clarify that the PI3K/Akt signaling pathway was highly enriched both in MC3T3-E1 and HUVECs cultured in β-TCP and 10MS/β-TCP groups.

The RNA-Seq was used to analyze the mechanism and differentially expressed genes (DEGs) related to osteogenesis in MC3T3-E1 cells and angiogenesis in HUVECs between β-TCP and 10MS/β-TCP groups, as shown in Figure 7. Compared with β-TCP group, there were 1223 upregulated DEGs and 1001 downregulated DEGs relative to MC3T3-E1 cells (Figure 7A), and 1,724 upregulated DEGs and 2013 downregulated DEGs relative to HUVECs (Figure 7C) in 10MS/β-TCP group. In order to further analyze the role of DEGs at the cellular level, GO enrichment was used to describe the role of DEG in molecular components, biological processes and biological processes, and molecular function (Figures 7B, D). In biological process, the cell proliferation, cellular process, biological adhesion, signaling, developmental process, growth, and biological regulation were upregulated in 10MS/β-TCP group, which indicated that 10MS/β-TCP scaffold promoted the proliferation, adhesion, and migration of MC3T3-E1 and HUVECs, thus providing conditions for bone regeneration and repair. The cellular level regulatory mechanisms were revealed by KEGG pathway analysis as shown in Figure 7E, presenting that the PI3K/AK signaling pathway was the most significantly enriched pathway in both MC3T3-E1 cells and HUVECs. Therefore, 10MS/β-TCP scaffold promoted osteogenesis and angiogenesis by activating PI3K/Akt signal pathway. PI3K/Akt pathway is an intracellular signal transduction pathway, which responds to extracellular signals to promote metabolism, proliferation, cell survival, growth, and angiogenesis (Wang et al., 2020). This process is mediated by serine or threonine phosphorylation of a series of downstream substrates. Previous studies have demonstrated that promoting osteogenesis enhanced bone regeneration or repair via the PI3K/Akt pathway in cellular level studies (Zhao et al., 2020; Yang et al., 2019; Zhang et al., 2016b; Wang et al., 2022). PI3K/Akt signaling pathways played an important role in the regulation of many important biological processes, especially in bone formation, maintenance and reconstruction (Sun et al., 2020; Wang et al., 2022).

Magnesium ions are well-documented to regulate cellular activities critical for bone formation (de Baaij et al., 2015). Studies have demonstrated that magnesium can activate the PI3K/Akt signaling pathway, which is pivotal for promoting osteoblast differentiation and mineralization (Wu et al., 2021). For example, magnesium ions upregulate osteogenic markers such as RUNX2, OPN, and OCN through PI3K/Akt-mediated pathways, facilitating the progression of bone formation. Additionally, magnesium also enhances endothelial cell survival and proliferation, contributing to angiogenesis (Gong et al., 2024). Similarly, silicon ions have been shown to enhance osteogenic differentiation and angiogenesis via the PI3K/Akt signaling pathway (Kong et al., 2022). Silicon promotes the expression of VEGF, a key factor in angiogenesis, through PI3K/Akt activation (Fu et al., 2020). This pathway also upregulates osteogenic factors, supporting coupled bone and vessel formation. Evidence suggests that silicon ions can modulate the activity of integrins and growth factors, further amplifying PI3K/Akt signaling to synergistically enhance osteogenesis and angiogenesis. In this study, the combination of magnesium and silicon ions may have a synergistic effect on the PI3K/Akt pathway, as both ions are known to independently target this signaling cascade to enhance bone and vascular tissue regeneration. The ability of MS/β-TCP scaffolds to release these bioactive ions and activate PI3K/Akt signaling highlights their potential for promoting coupled osteogenesis and angiogenesis in bone regeneration applications.

**FIGURE 8:**
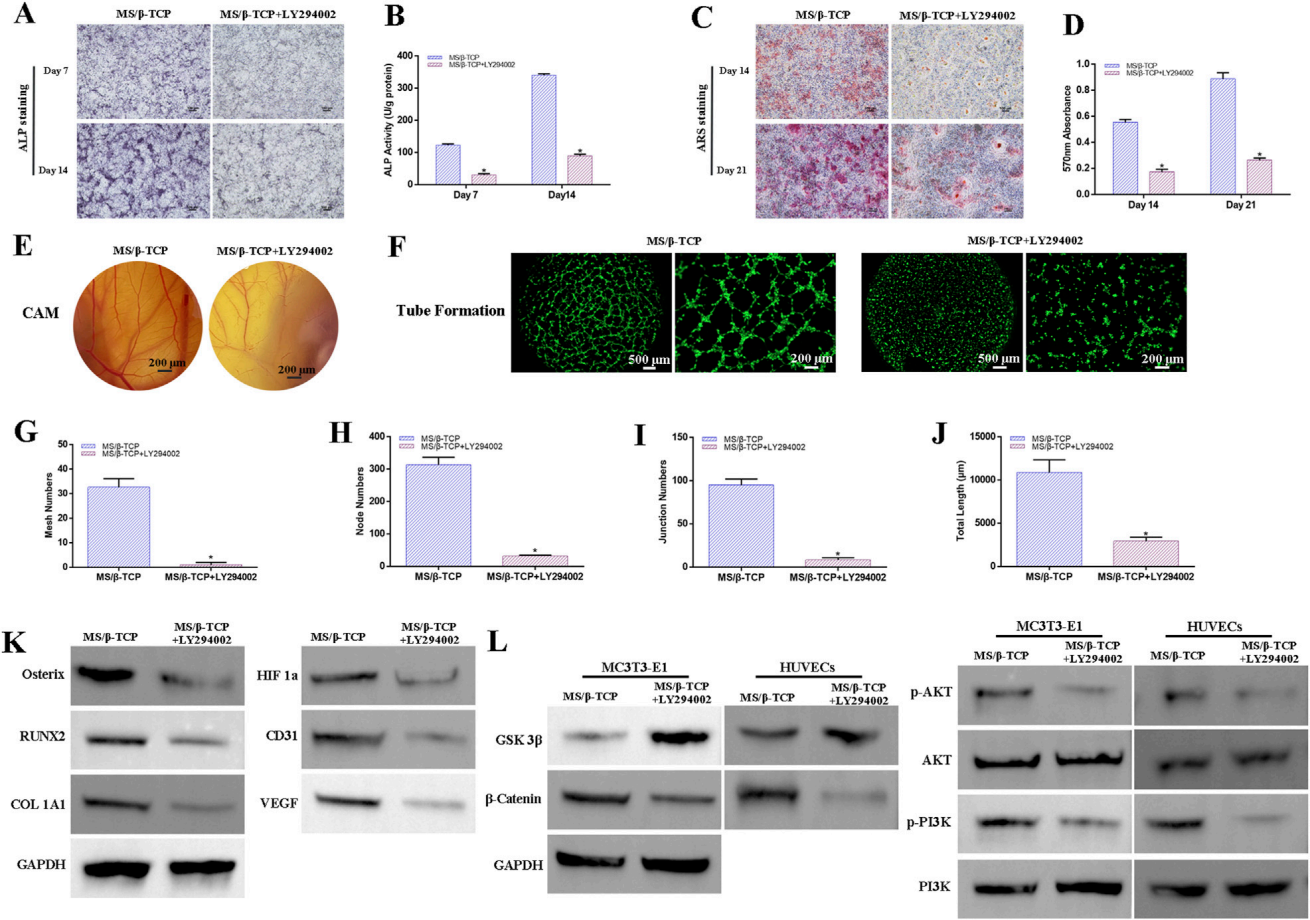
MS/β-TCP scaffolds promoted osteogenesis and angiogenesis by activating PI3K/Akt signaling pathway *in vitro*. ALP proteins **(A)**, ALP activity **(B)**, the mineralization nodules **(C)** and quantitative analysis **(D)** were significantly decreased in MS/β-TCP + LY294002 group compared with MS/β-TCP group. *In vivo* CAM assay **(E)**, *in vitro* tube formation assay **(F)**, and quantitative analysis of tube formation **(G–J)** showed that the angiogenic ability of MS/β-TCP was significantly weakened after treatment with LY294002. **(K)** Protein expression level of osteogenesis-related Osterix, RUNX2, COL 1A1 and angiogenesis-related HIF1a, CD31, VEGF, and **(L)** PI3K/Akt pathway-related proteins were significantly suppressed in MS/β-TCP + LY294002 group compared with MS/β-TCP group. *p < 0.05 versus MS/β-TCP group.

In order to further verify its mechanism of action, LY294002, an inhibitor of PI3K signaling pathway (Miao et al., 2022), was added to 10MS/β-TCP scaffold extracts to culture the MC3T3-E1 cells and HUVECs. After blocking the PI3K/Akt signaling pathway, 10MS/β-TCP scaffold containing LY294002 greatly hindered the osteogenic differentiation of MC3T3-E1 cells (Figures 8A, B), the ALP staining and ALP activity were significantly decreased in 10MS/β-TCP + LY294002 group than that of 10MS/β-TCP group. In addition, the Alizarin Red S staining (Figure 8C) and quantitative analysis of mineralized nodules (Figure 8D) showed consistent results that the area of mineralized nodules was less in 10MS/β-TCP + LY294002 group than that of 10MS/β-TCP group. In addition, according to the results of CAM measurement *in vivo* (Figure 8E) and tube formation measurement *in vitro* (Figure 8F), the angiogenic ability was further evaluated. The results showed that 10MS/β-TCP + LY294002 prevented the formation of blood vessels and reduced the tube formation of HUVECs compared to 10MS/β-TCP group. The quantified analysis of tube formation was consistent with macrographs in term of mesh numbers (Figure 8G), node numbers (Figure 8H), total length (Figure 8I), and junction numbers (Figure 8J). Western blot also demonstrated lower protein expression of RUNX2, Osterix, COL 1A1 related to osteogenesis, and HIF1a, CD31, and VEGF related to angiogenesis in 10MS/β-TCP + LY294002 group (Figure 8K). In addition, the expression of proteins related to PI3K/Akt signaling pathway were regulated by LY294002 mediated. As shown in Figure 8L, β-Catenin, phosphorylated AKT (p-AKT), and phosphorylated PI3K (p-PI3K) were downregulated expression, whereas the GSK 3β was upregulated expression, indicating that PI3K/Akt signaling pathway was inhibited in both MC3T3-E1 cells and HUVECs cultured with 10MS/β-TCP + LY294002. Therefore, MS/β-TCP scaffold played a dual role in osteogenesis and angiogenesis by activating the PI3K/Akt signaling pathway.

### MS/β-TCP scaffold promotes bone defect repair *in vivo*

**FIGURE 9:**
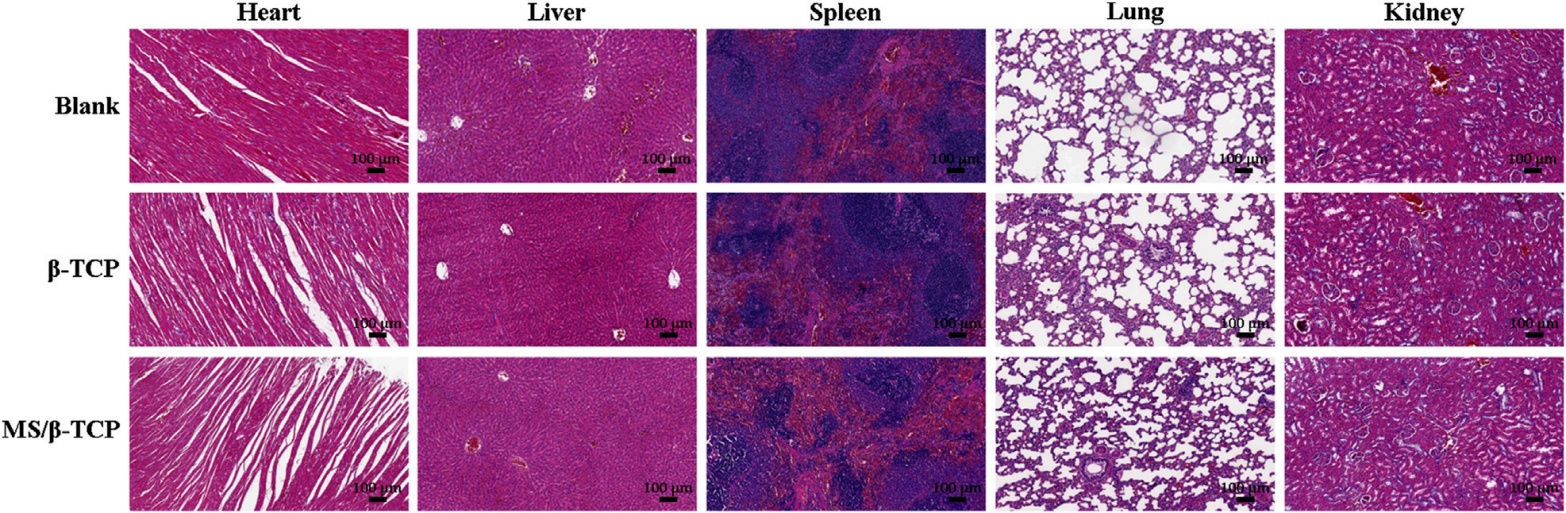
*In vivo* biosafety evaluation by H&E-stained heart, liver, spleen, lung, and kidney tissues.

Despite the results *in vitro*, *in vivo* biological effects were further evaluated. The biosafety of scaffolds was assessed by hematoxylin and eosin (HE) staining of the heart, liver, spleen, lung, and kidney after implantation for 4°weeks (Figure 9). It was obviously observed that neither inflammatory cells nor histopathological abnormalities occurred in organs, indicating that MS/β-TCP scaffolds had good biocompatibility without any toxicity, which provided the possibility for clinical translational application.

**FIGURE 10:**
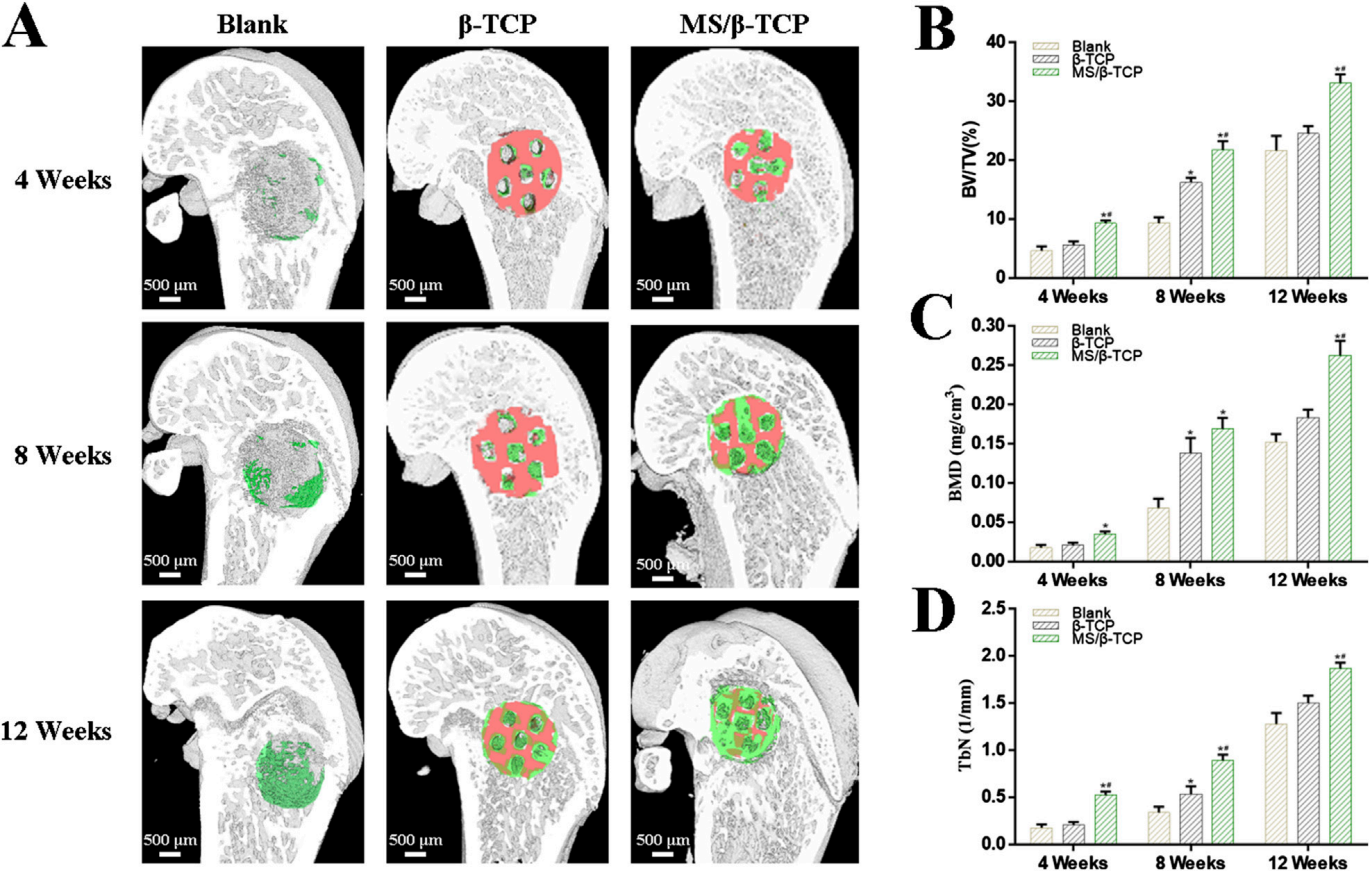
Micro-CT evaluated the effect of the scaffolds on bone defect repair. **(A)** 3D reconstructed micro-CT images of new bone formation at 4, 8, and 12 weeks. Green represented new bone tissue, and Orange represented scaffolds. **(B–D)** Quantitative analysis of the bone tissue volume/total tissue volume (BV/TV), bone mineral density (BMD), trabecular numbers (TbN) in the defect area at 4, 8, and 12 weeks. *p < 0.05 versus blank group, ^#^p < 0.05 versus β-TCP group.

According to the results of the *in vitro* study, the bone regeneration ability of rats with critical size skull defects *in vivo* was evaluated by micro-CT at the 4th, 8th, and 12th weeks (Figure 10). Figure 10A showed 3D reconstructed images of new bone formation. Micro-CT images showed that the blank group and β-TCP group had only limited new bone formation, while MS/β-TCP group promoted significant new bone formation. With the extension of bone repair time, the bone formation of MS/β-TCP group was better than that of blank group and β-TCP. The result showed that the 3D printing of MS/β-TCP scaffold was more likely to promote bone repair and significantly enhance the connectivity of new bone tissue. The micro-CT parameters of BV/TV (Figure 10B), BMD (Figure 10C), and TbN (Figure 10D) for quantitative analysis were also obtained to further evaluate the observation results. The overall trend is consistent with the 3D reconstruction image. In this study, the BV/TV ratios were 4.66% ± 0.75%, 9.35% ± 0.98%, and 21.66% ± 2.50% for the blank group; 5.65% ± 0.59%, 16.29% ± 0.75%, and 24.51% ± 1.34% for the β-TCP group; and 9.33% ± 0.45%, 21.79% ± 1.45%, and 33.18% ± 1.43% for the MS/β-TCP group in the 4th, 8th, and 12th weeks, respectively. Meanwhile, the BMDs were 0.02 ± 0.003 g/cm^3^, 0.07 ± 0.012 g/cm^3^, and 0.15 ± 0.010 g/cm^3^ for the blank group; 0.02 ± 0.003 g/cm^3^, 0.14 ± 0.019 g/cm^3^, and 0.18 ± 0.011 g/cm^3^ for the β-TCP group; and 0.04 ± 0.003 g/cm^3^, 0.17 ± 0.014 g/cm^3^, and 0.26 ± 0.019 g/cm^3^ for the MS/β-TCP group in the 4th, 8th, and 12th weeks, respectively. Trabecular bone existed in the bone marrow cavity and were the extension of cortical bone, mainly presenting an irregular three-dimensional network structure in cancellous bone covered with intraosseous cells and connected with the surrounding osteoblasts. In addition, the TbN measures were 0.18 ± 0.034 1/mm, 0.34 ± 0.062 1/mm, and 0.28 ± 0.118 1/mm for the blank group; 0.21 ± 0.030 1/mm, 0.54 ± 0.082 1/mm, and 1.43 ± 0.127 1/mm for the β-TCP group; and 0.52 ± 0.041 1/mm, 0.89 ± 0.061 1/mm, and 1.74 ± 0.096 1/mm for the MS/β-TCP group in the 4th, 8th, and 12th weeks, respectively. The results showed that the BV/TV, BMD, and TbN of MS/β-TCP group at each time point are the highest among all groups, indicating that MS/β-TCP group showed faster and better bone regeneration.

**FIGURE 11:**
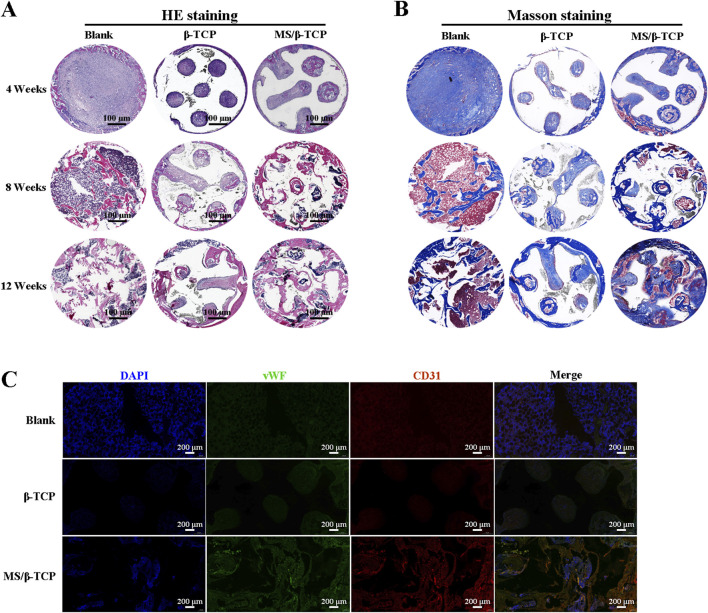
The newly formed tissues were evaluated histologically by HE staining **(A)** and Masson staining **(B)** at 4, 8 and 12 weeks after operation. **(C)** Immunofluorescence images of endogenous cells (DAPI, CD31, and vWF) in new tissue sections 12 weeks after operation.

In order to further verify the effect of bone induction, we performed HE staining and Masson staining on the new bone tissue at the 4th, 8th, and 12th weeks. As shown in Figures 11A, B, the results showed fibrous tissue and new bone in the area of the bone defect, respectively. In the 4th week, few new bone tissues were formed and scaffolds were barely degraded in the bone defect repair. However, fiber tissue in MS/β-TCP group was more than that in blank group and β- TCP. From week 8–12, the scaffolds implanted in the body degraded from the edge to the center, and the degradation rate of MS/β-TCP scaffold was faster than that of β-TCP scaffold. At the same time, mature bone tissue was observed at the bone defect. This is consistent with the results of micro-CT analysis, and the bone defect area showed greater evidence of new bone formation in the MS/β-TCP group than that of blank and β-TCP groups. Moreover, more new bone tissues grew into the MS/β-TCP scaffold than that of β-TCP scaffold. In order to study the angiogenesis activity in the bone defect repair area, fluorescent markers (CD31 and vWF) of vascular endothelial cells were performed in the staining of tissue sections as shown in Figure 11C. The results showed that the expression of CD31 and vWF in MS/β-TCP group was higher than that in blank group and β- TCP group. In other words, MS/β-TCP scaffold resulted in an increase in blood vessel density and new bone thickness *in vivo*. Due to the limitation of oxygen and nutrient exchange, uneven cell distribution and poor migration in the blank group (Yan et al., 2019; Eftekhari et al., 2017; Nyberg et al., 2017), new bone tissue was uniformly generated at the bone defect site with the degradation of the MS/β-TCP scaffold. This revealed that MS/β-TCP scaffold was easier to induce angiogenesis during bone regeneration. To sum up, the research results showed that the of 3D printing of MS/β-TCP scaffold had the best effect in osteogenesis and angiogenesis for repairing bone defects.

## Conclusion

In this research, we successfully fabricated MS/β-TCP scaffolds with 3D interconnected multi-stage macroporous structure to repair bone defects. MS/β-TCP scaffolds had a better mechanical property and could release magnesium and silicon ions continuously. By adding magnesium silicate to play its synergistic role, the biological activity of β-TCP material in terms of the compatibility, bone conductivity, osteogenesis induction, angiogenesis and other properties of scaffolds could be further improved. MS/β-TCP induced endothelial cells to generate neovascularization and osteogenic differentiation by promoting the activity of osteoblasts, and promoted the adhesion and proliferation of osteoblasts and vascular endothelial cells. Moreover, Transcriptome analysis results show that 3D printed MS/β-TCP scaffold plays a dual role in osteogenesis and angiogenesis by activating PI3K/Akt signal pathway, whereas the expression of genes and proteins associated with osteogenesis and angiogenesis was significantly downregulated the PI3K/Akt signaling pathway was inhibited. In addition, *in vivo* confirmed that MS/β-TCP scaffolds had better bone defect repair effect on angiogenesis and bone regeneration in rat femur condyle defect model. However, long-term and in-depth research is still needed, this study explored the optimal incorporation ratio of magnesium silicate mixed with β-TCP to prepare scaffolds to promote bone regeneration, providing cytological and animal experimental basis for subsequent transformation applications.

## Data Availability

The datasets presented in this article are not readily available because the RNA sequencing data associated with this study are currently being used for ongoing research projects and are not yet ready for public release. Consequently, the authors are unable to make this dataset publicly available at this time. Requests to access the datasets should be directed to the corresponding author.
