# Supplementary figures and images for: 3D printed magnesium silicate/β-tricalcium phosphate scaffolds promote coupled osteogenesis and angiogenesis (part 1 of 2)
Source: Front Bioeng Biotechnol. 2025 Jan 31;12:1518145. doi: 10.3389/fbioe.2024.1518145 (PMC11841418; doi:10.3389/fbioe.2024.1518145)

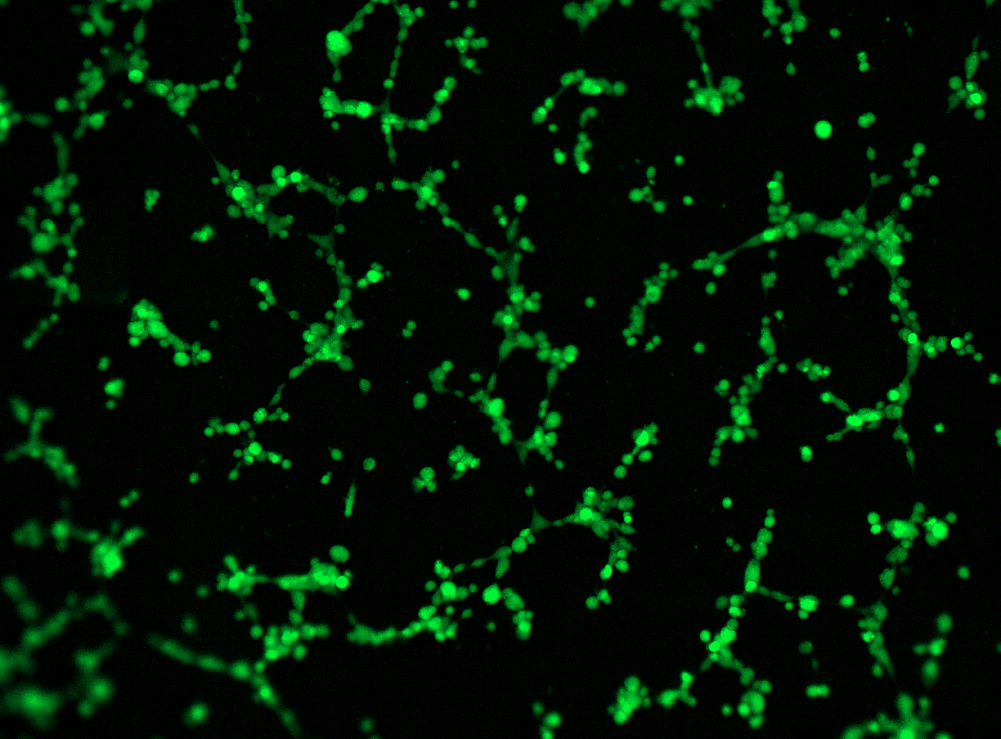

Supplement: Supplementary file 2 [file DataSheet3.zip › Figure 5 and 6/Figure 5b/0MS-1.tif]

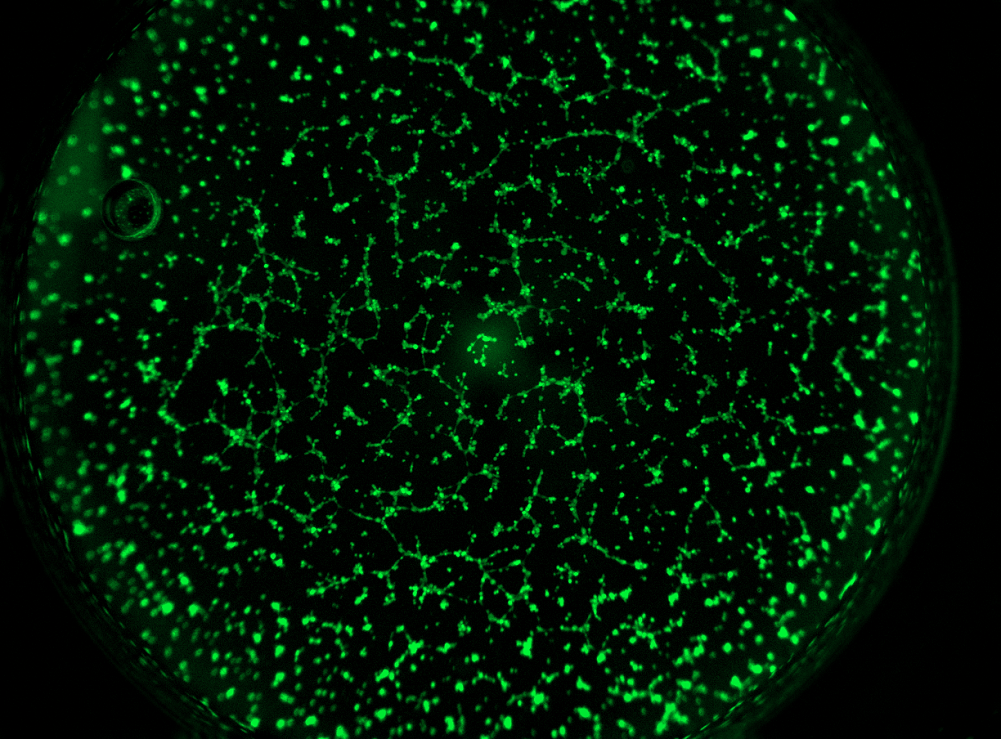

Supplement: Supplementary file 2 [file DataSheet3.zip › Figure 5 and 6/Figure 5b/0MS.tif]

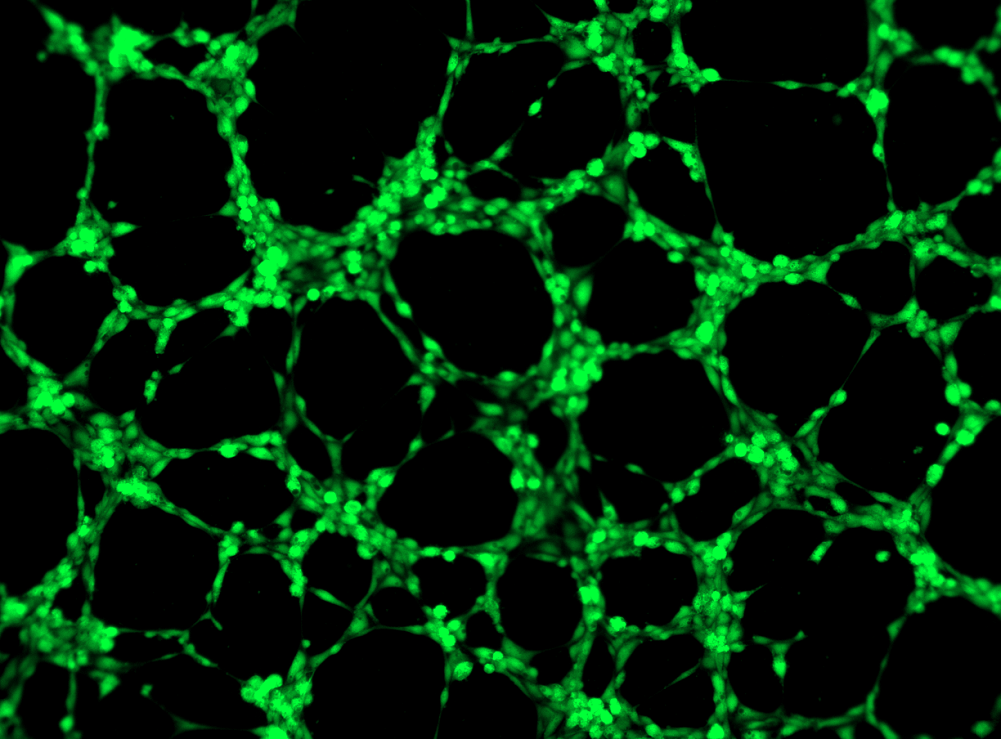

Supplement: Supplementary file 2 [file DataSheet3.zip › Figure 5 and 6/Figure 5b/10MS-1.tif]

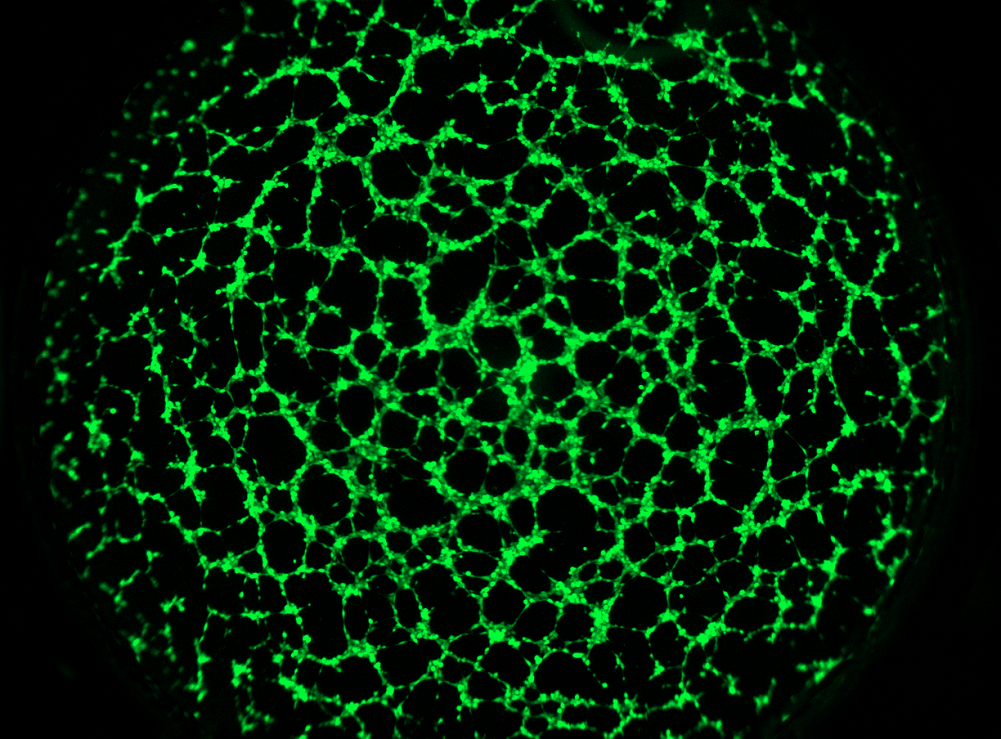

Supplement: Supplementary file 2 [file DataSheet3.zip › Figure 5 and 6/Figure 5b/10MS.tif]

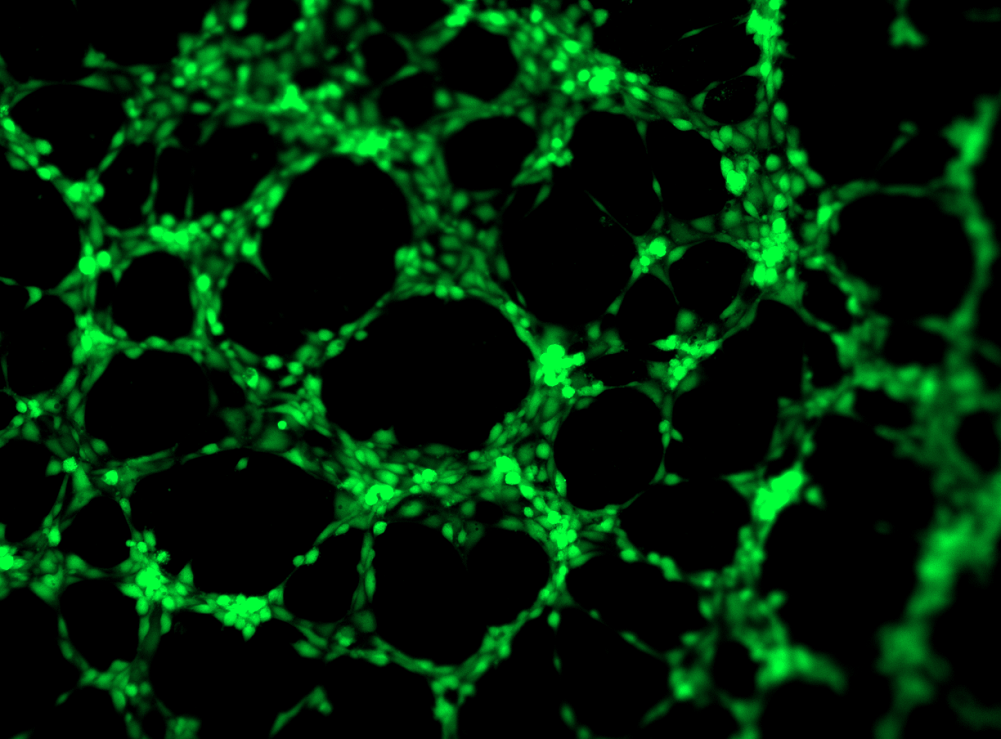

Supplement: Supplementary file 2 [file DataSheet3.zip › Figure 5 and 6/Figure 5b/15MS-1.tif]

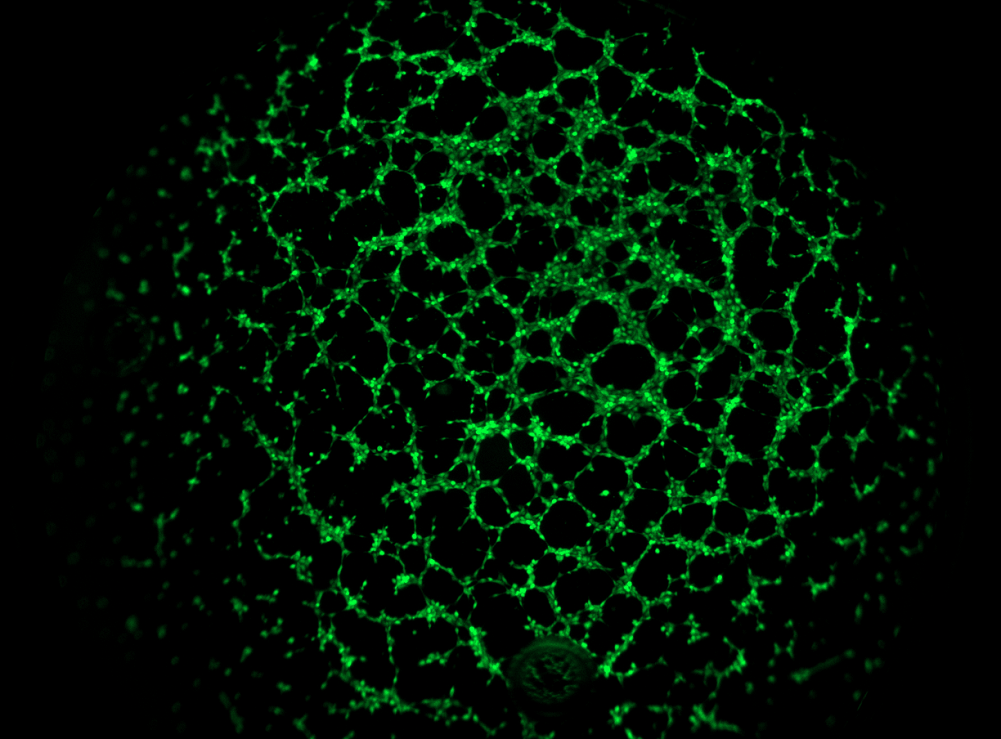

Supplement: Supplementary file 2 [file DataSheet3.zip › Figure 5 and 6/Figure 5b/15MS.tif]

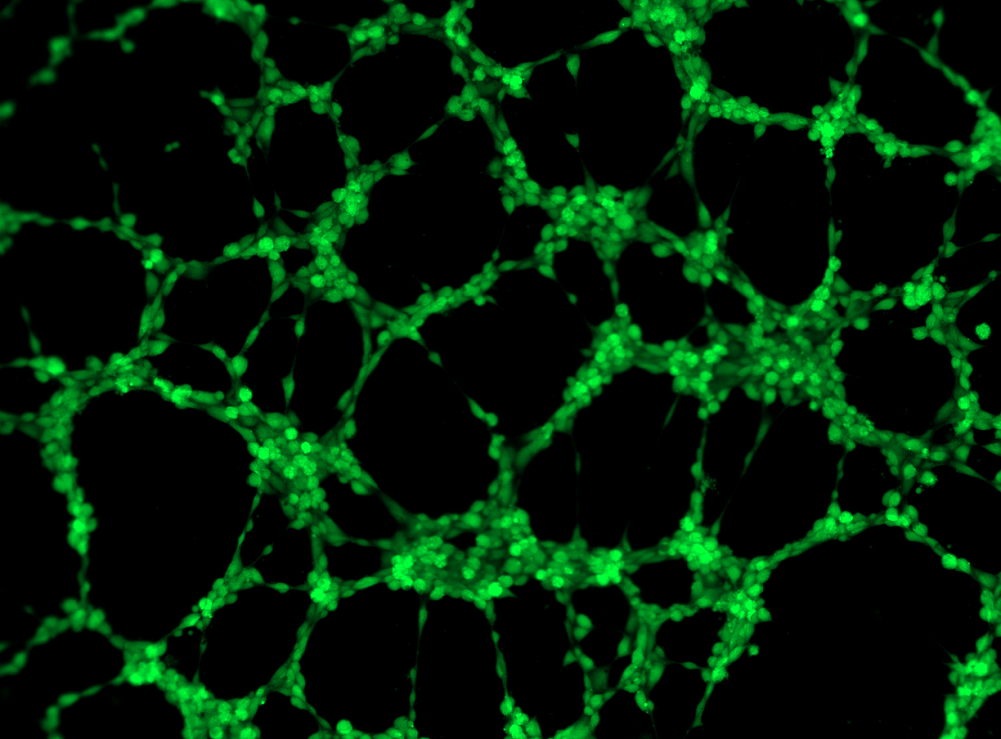

Supplement: Supplementary file 2 [file DataSheet3.zip › Figure 5 and 6/Figure 5b/5MS-1.tif]

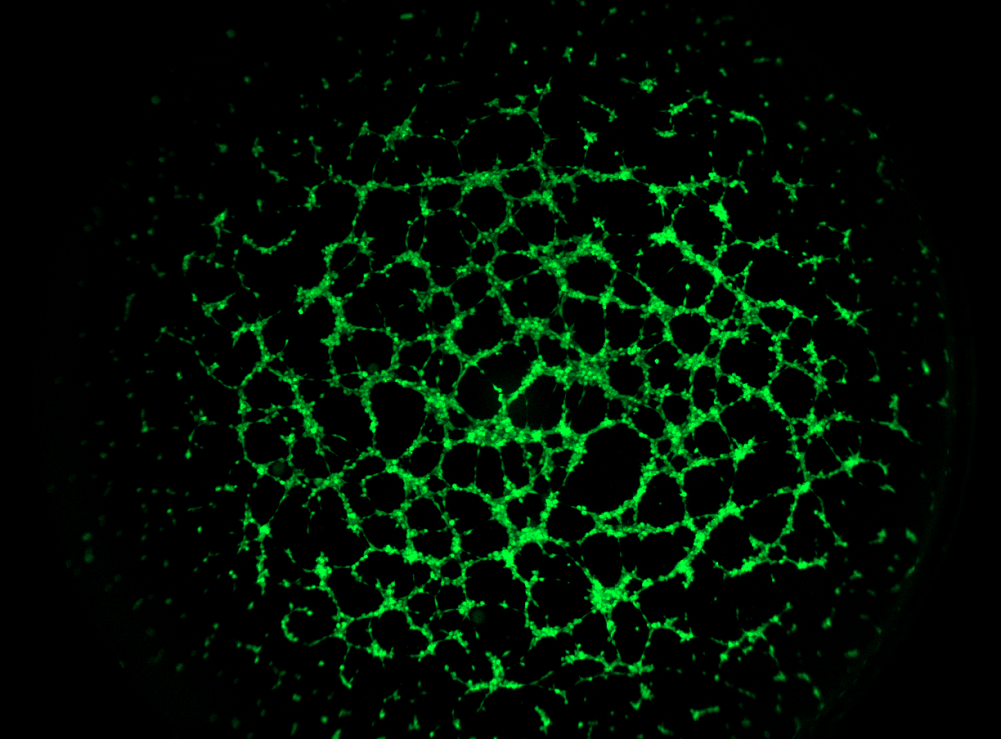

Supplement: Supplementary file 2 [file DataSheet3.zip › Figure 5 and 6/Figure 5b/5MS.tif]

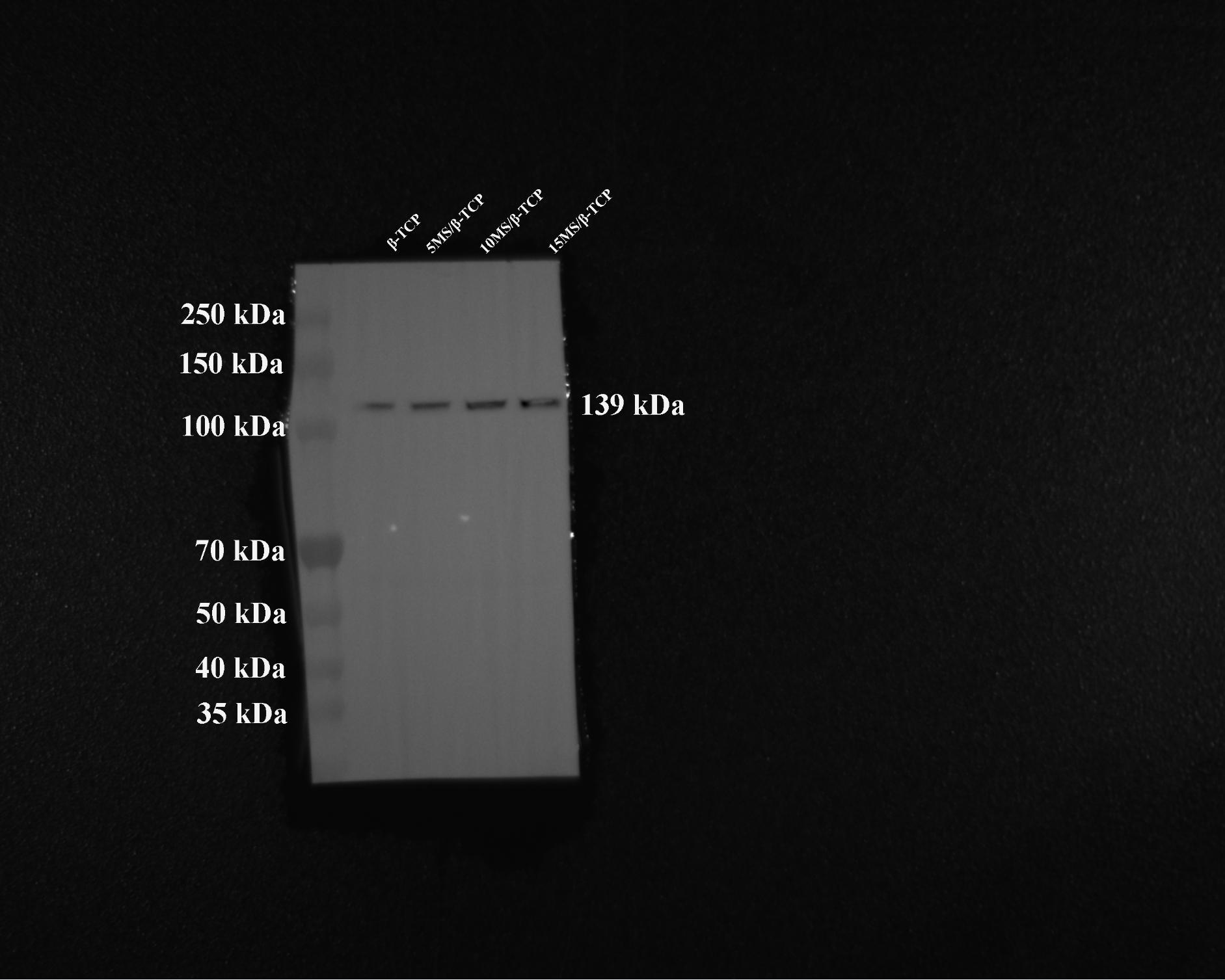

Supplement: Supplementary file 2 [file DataSheet3.zip › Figure 5 and 6/Figure 6/Figure 6C Col1a1.tif]

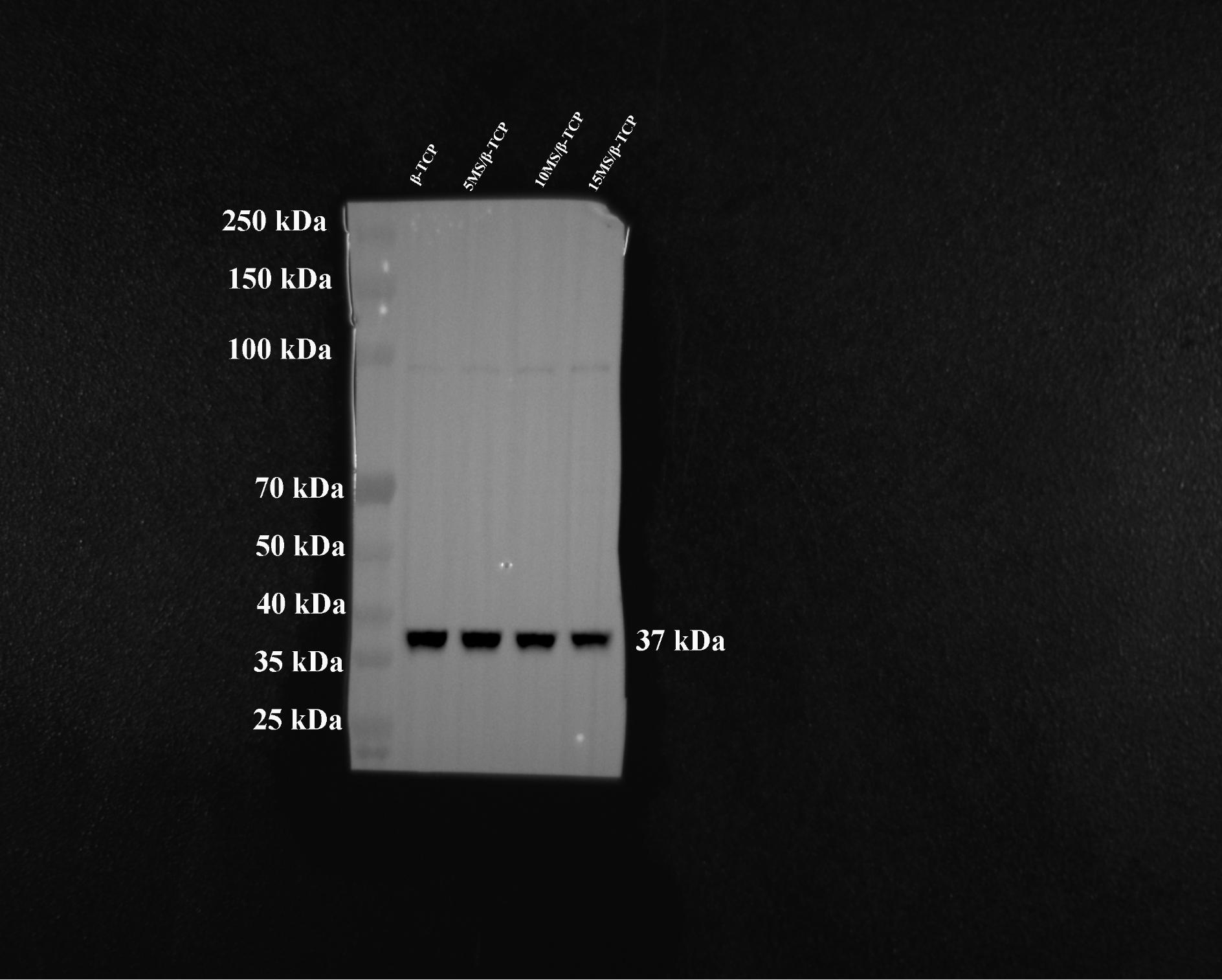

Supplement: Supplementary file 2 [file DataSheet3.zip › Figure 5 and 6/Figure 6/Figure 6C GAPDH - C.tif]

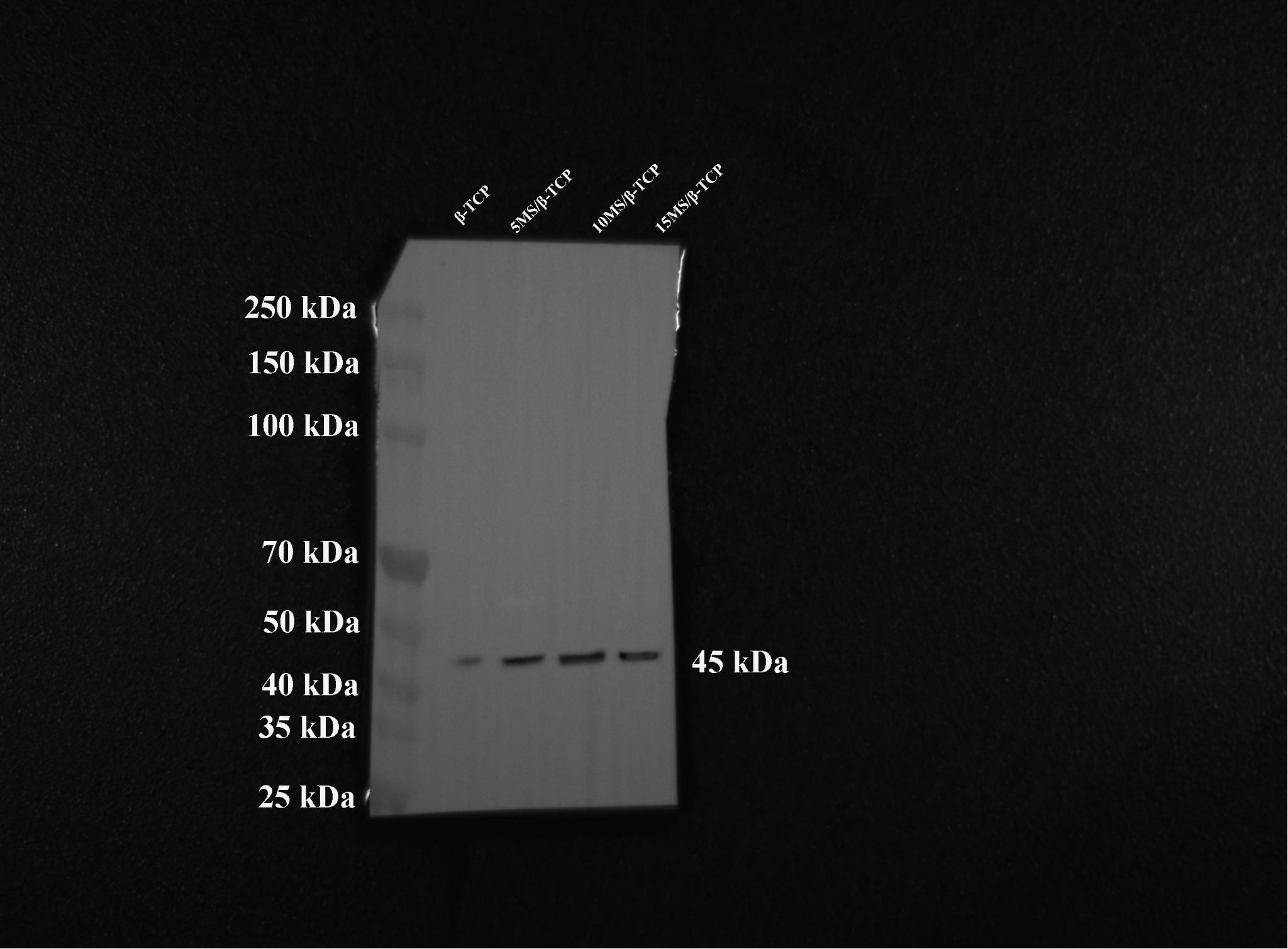

Supplement: Supplementary file 2 [file DataSheet3.zip › Figure 5 and 6/Figure 6/Figure 6C Osterix.tif]

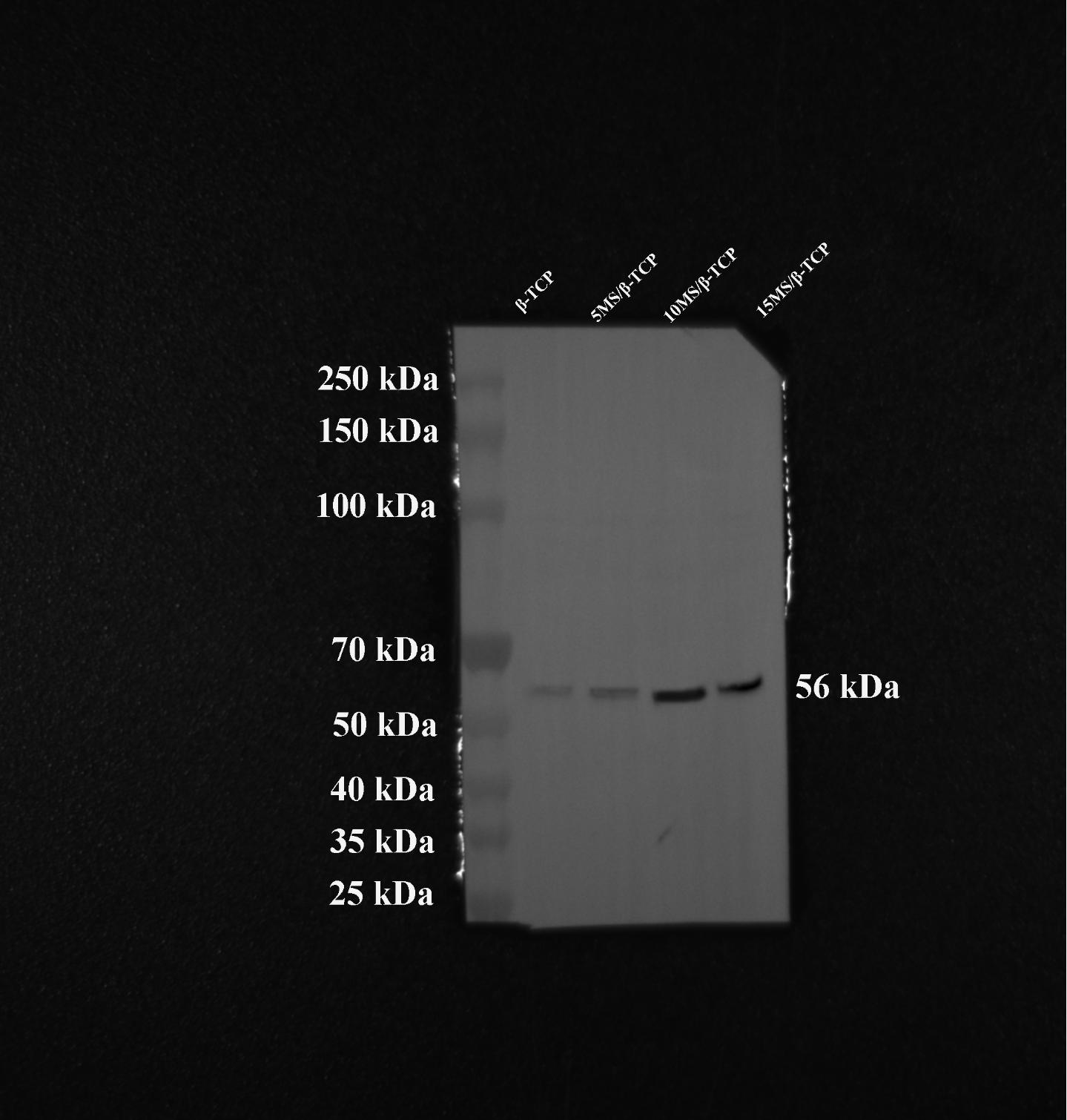

Supplement: Supplementary file 2 [file DataSheet3.zip › Figure 5 and 6/Figure 6/Figure 6C RUNX2.tif]

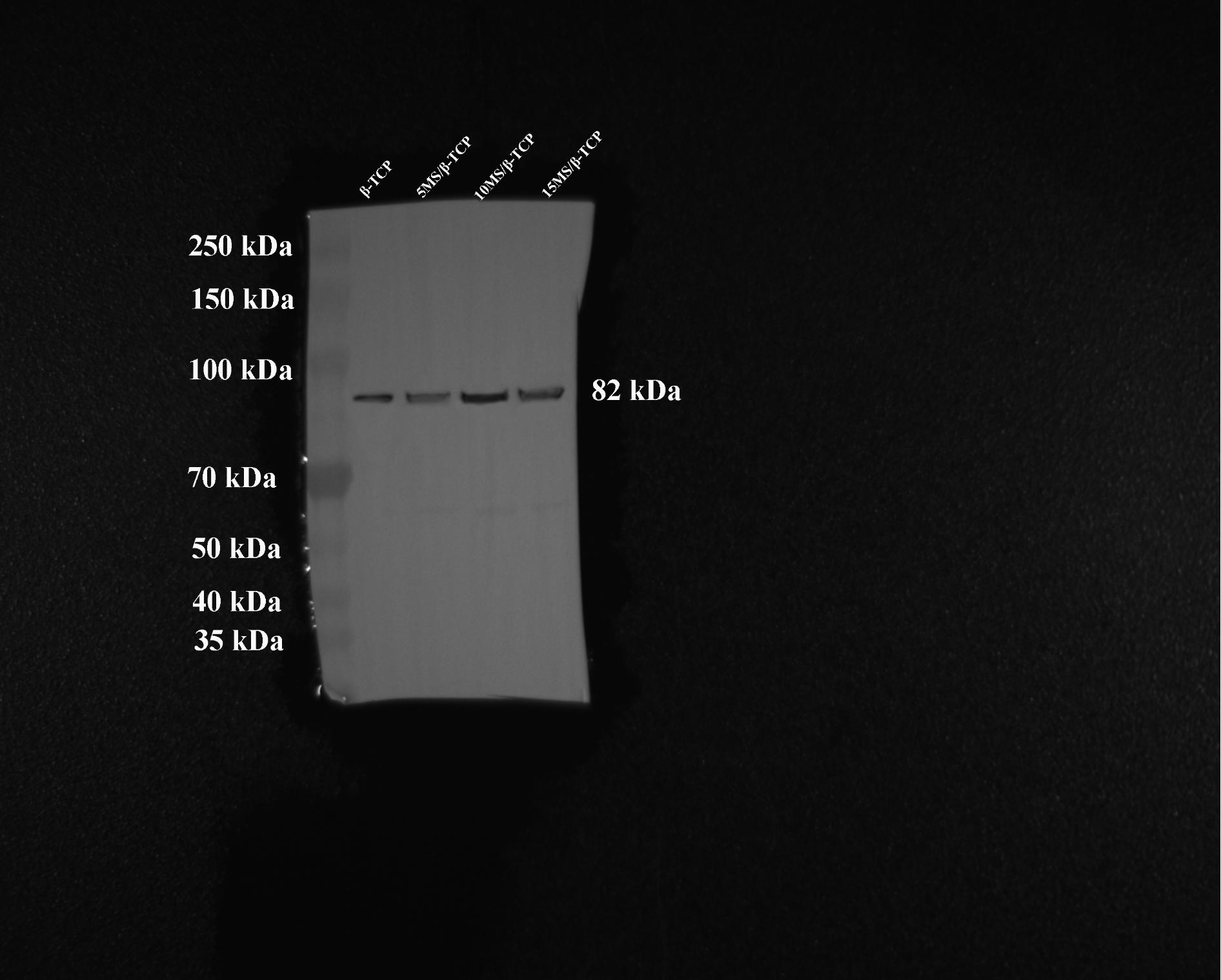

Supplement: Supplementary file 2 [file DataSheet3.zip › Figure 5 and 6/Figure 6/Figure 6F CD31.tif]

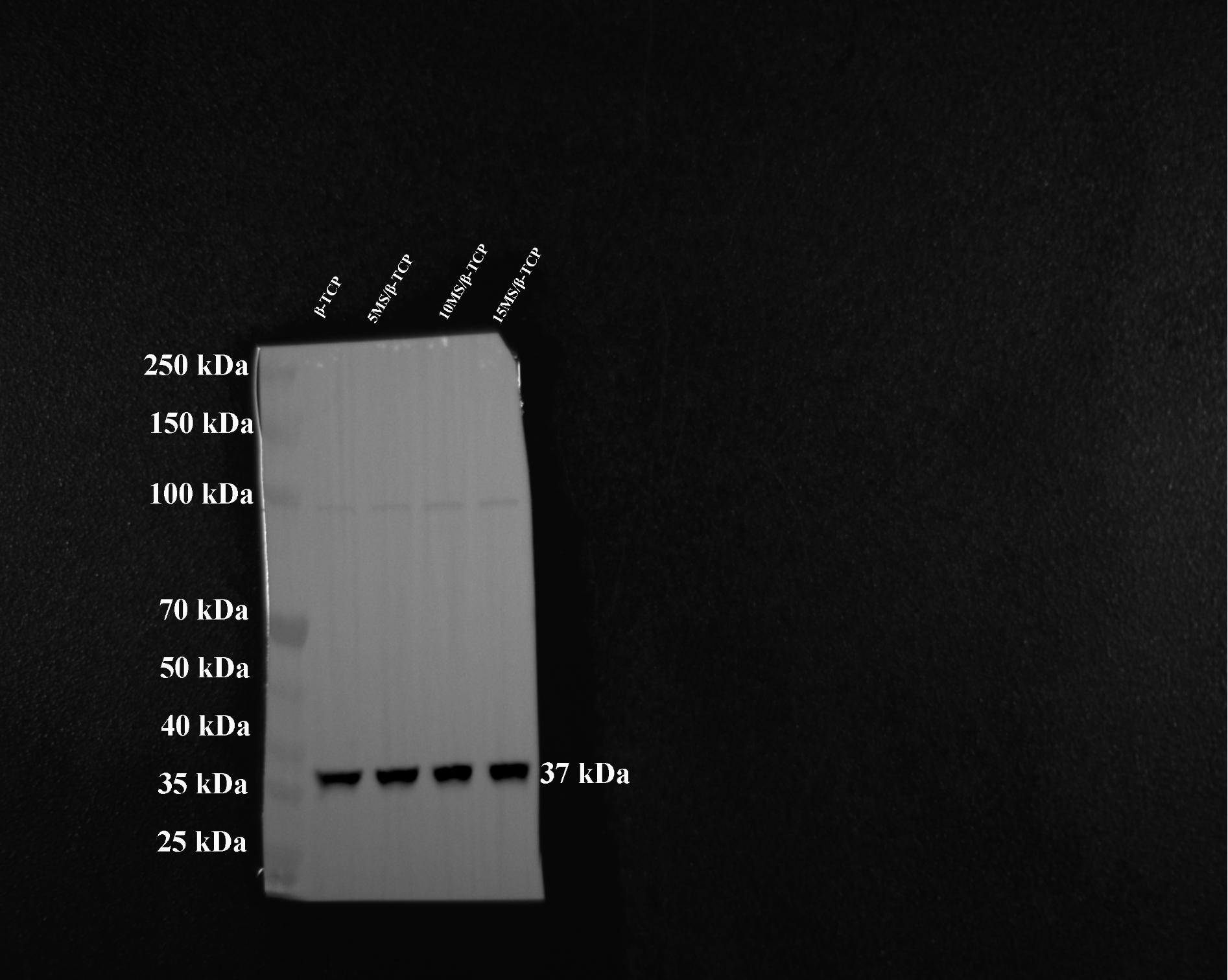

Supplement: Supplementary file 2 [file DataSheet3.zip › Figure 5 and 6/Figure 6/Figure 6F GAPDH - F.tif]

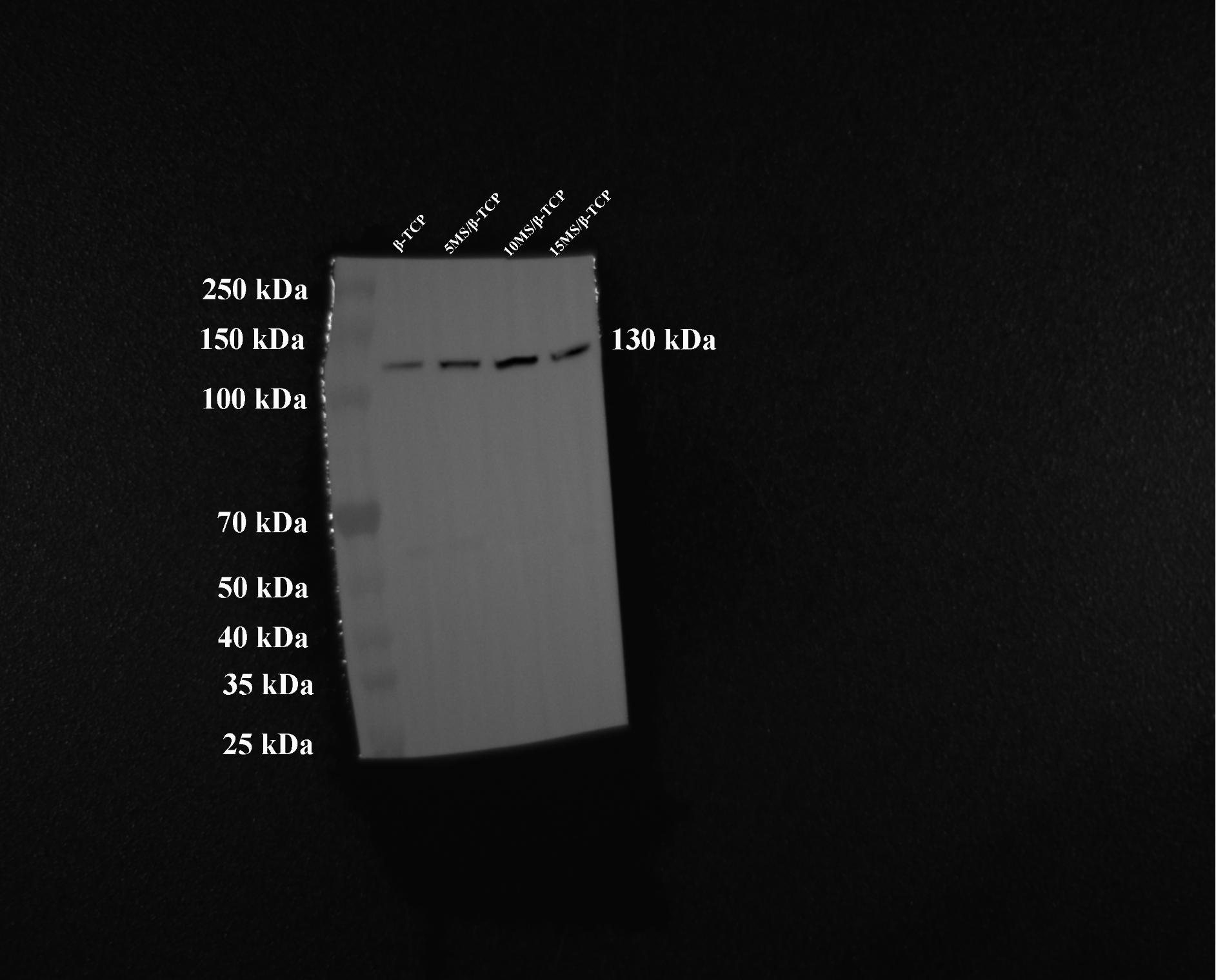

Supplement: Supplementary file 2 [file DataSheet3.zip › Figure 5 and 6/Figure 6/Figure 6F HIF 1a.tif]

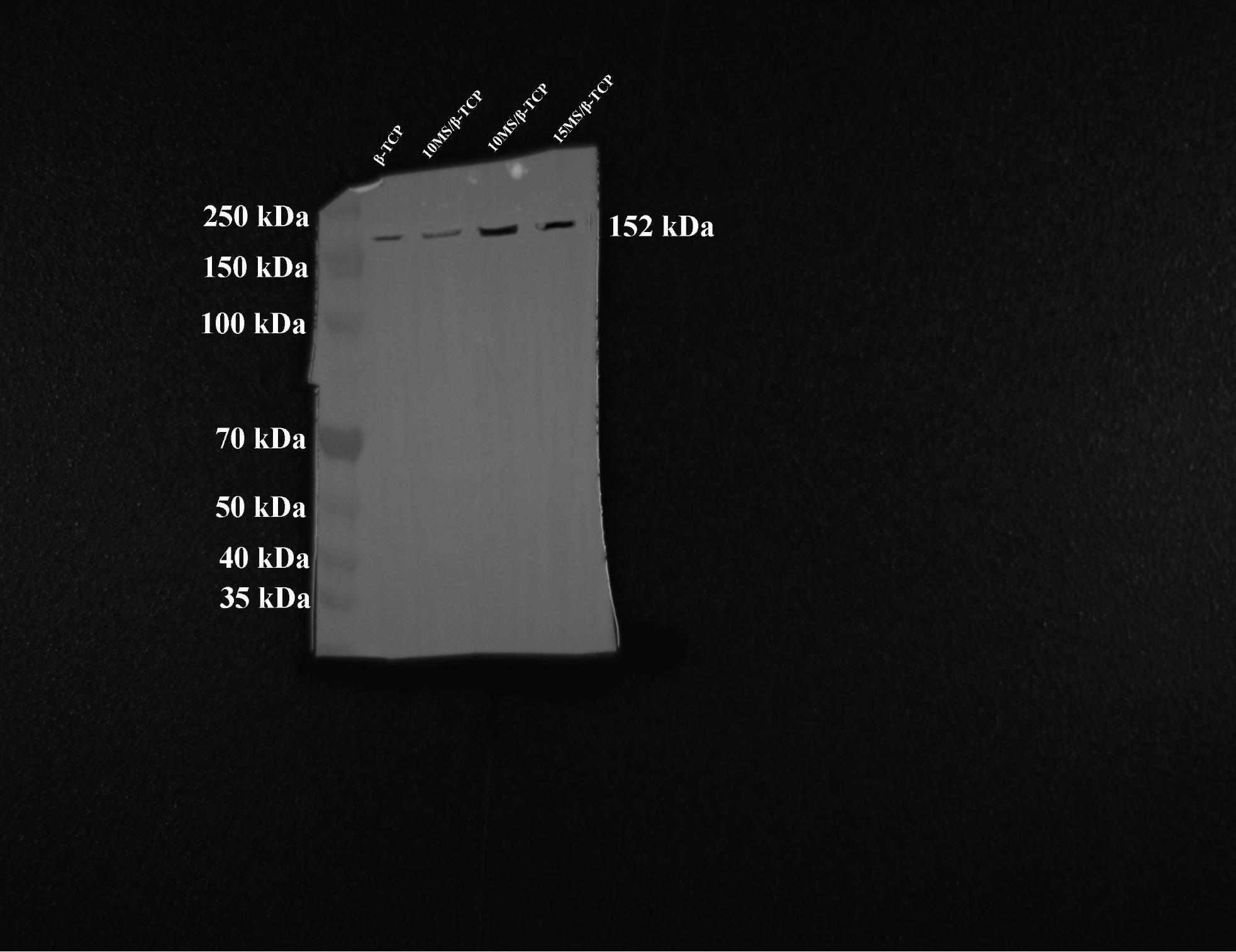

Supplement: Supplementary file 2 [file DataSheet3.zip › Figure 5 and 6/Figure 6/Figure 6F VEGF.tif]

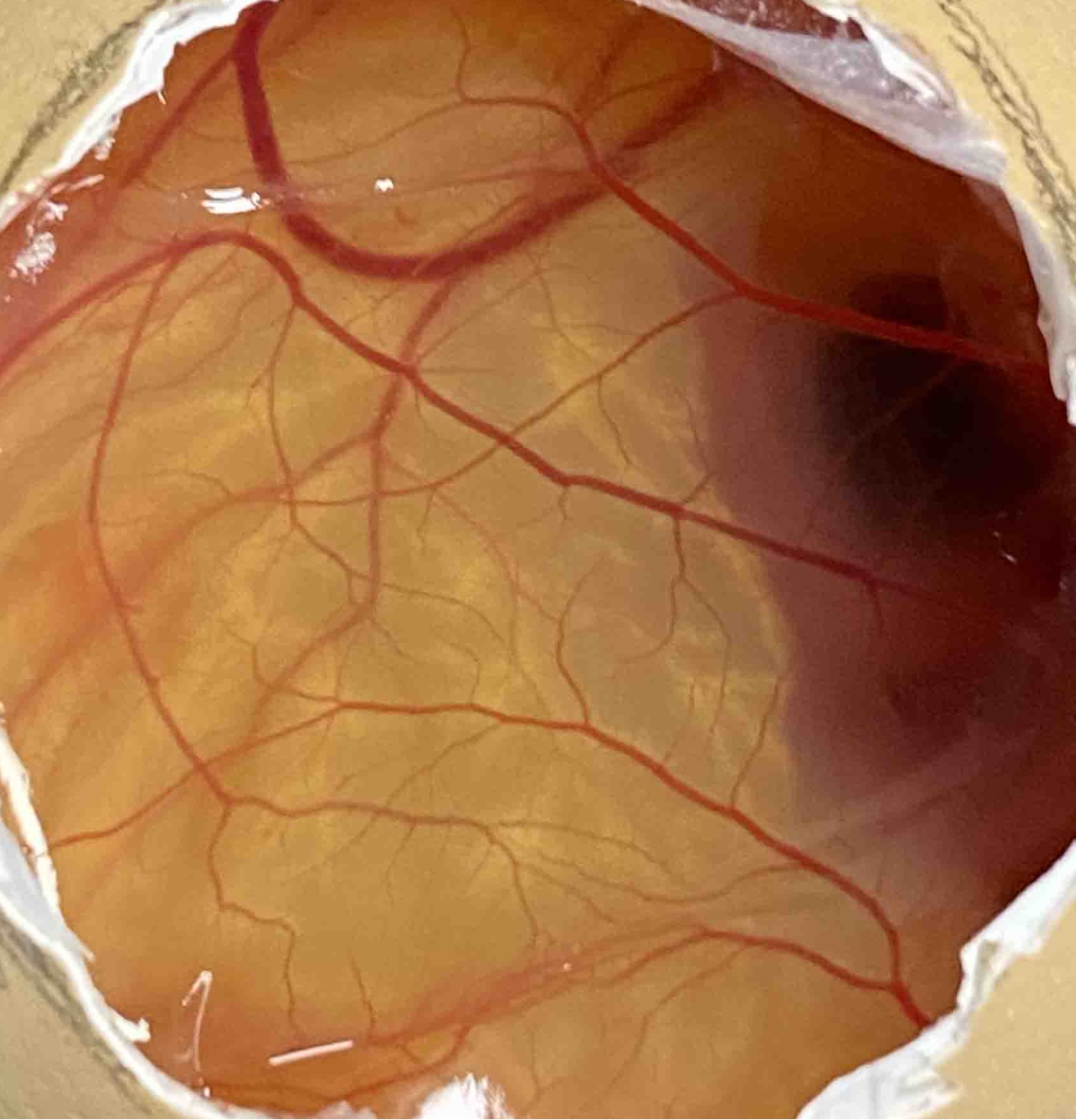

Supplement: Supplementary file 2 [file DataSheet3.zip › Figure 5 and 6/figure 5a/0MS.png]

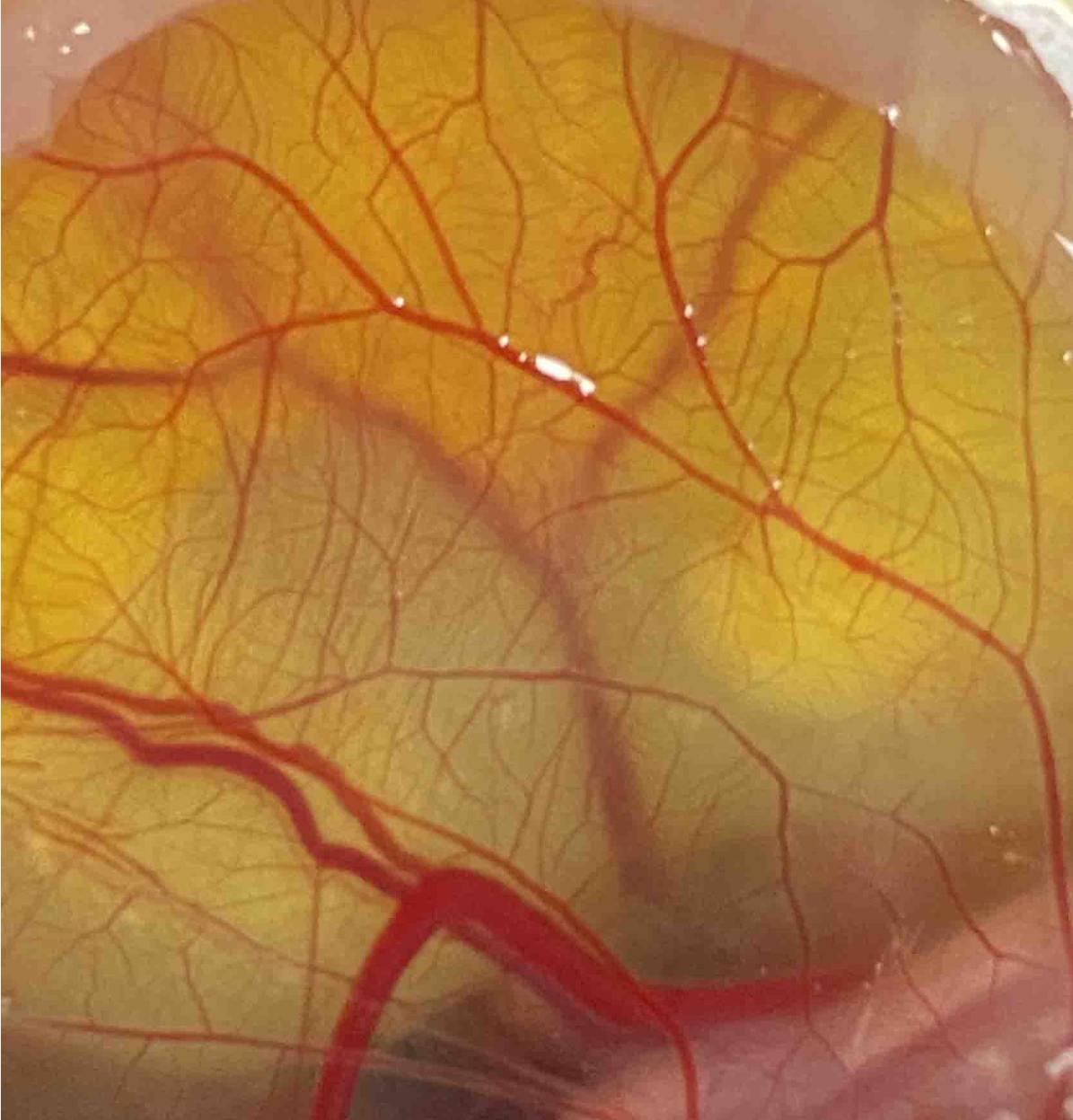

Supplement: Supplementary file 2 [file DataSheet3.zip › Figure 5 and 6/figure 5a/10MS.png]

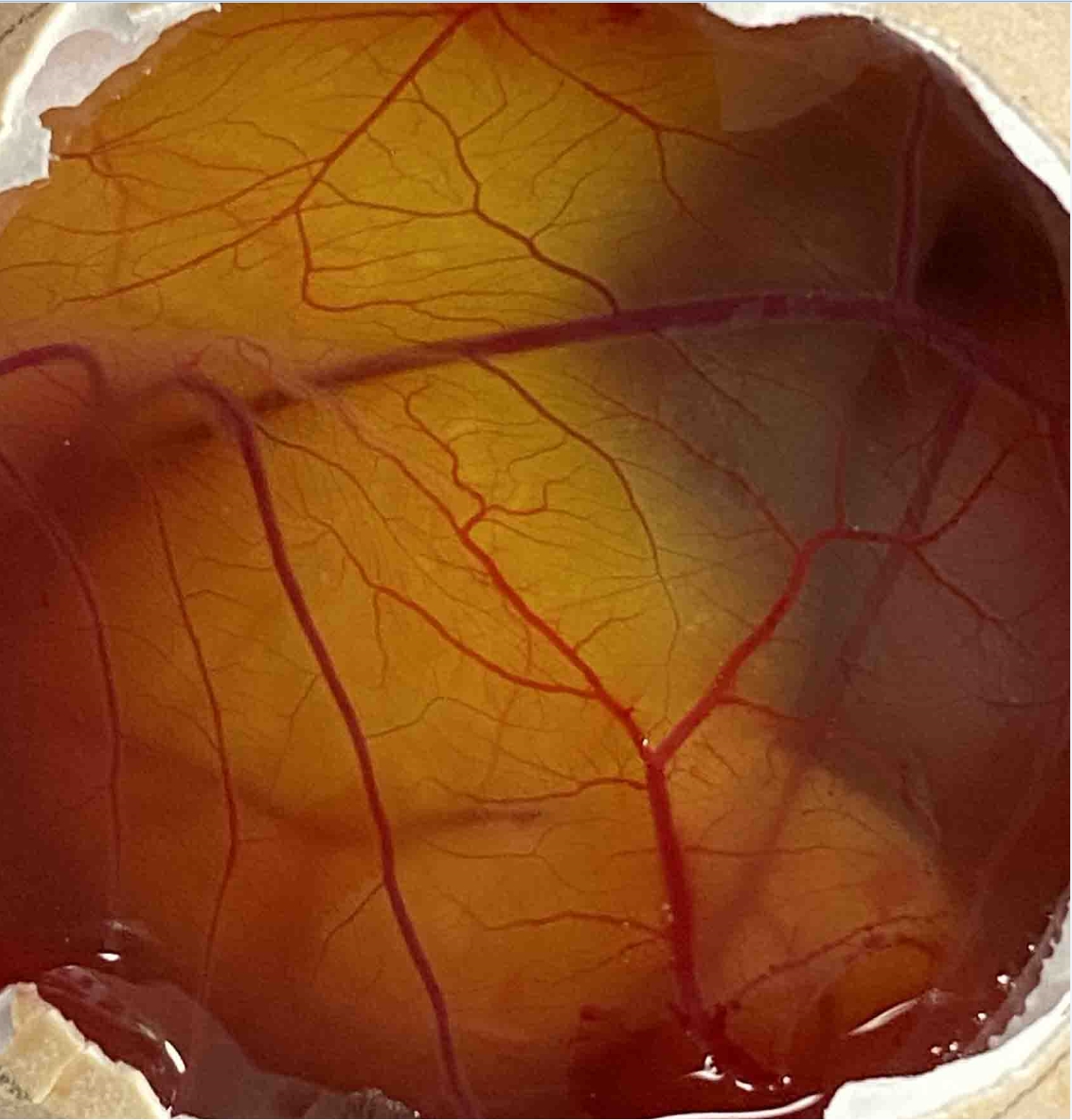

Supplement: Supplementary file 2 [file DataSheet3.zip › Figure 5 and 6/figure 5a/15MS.png]

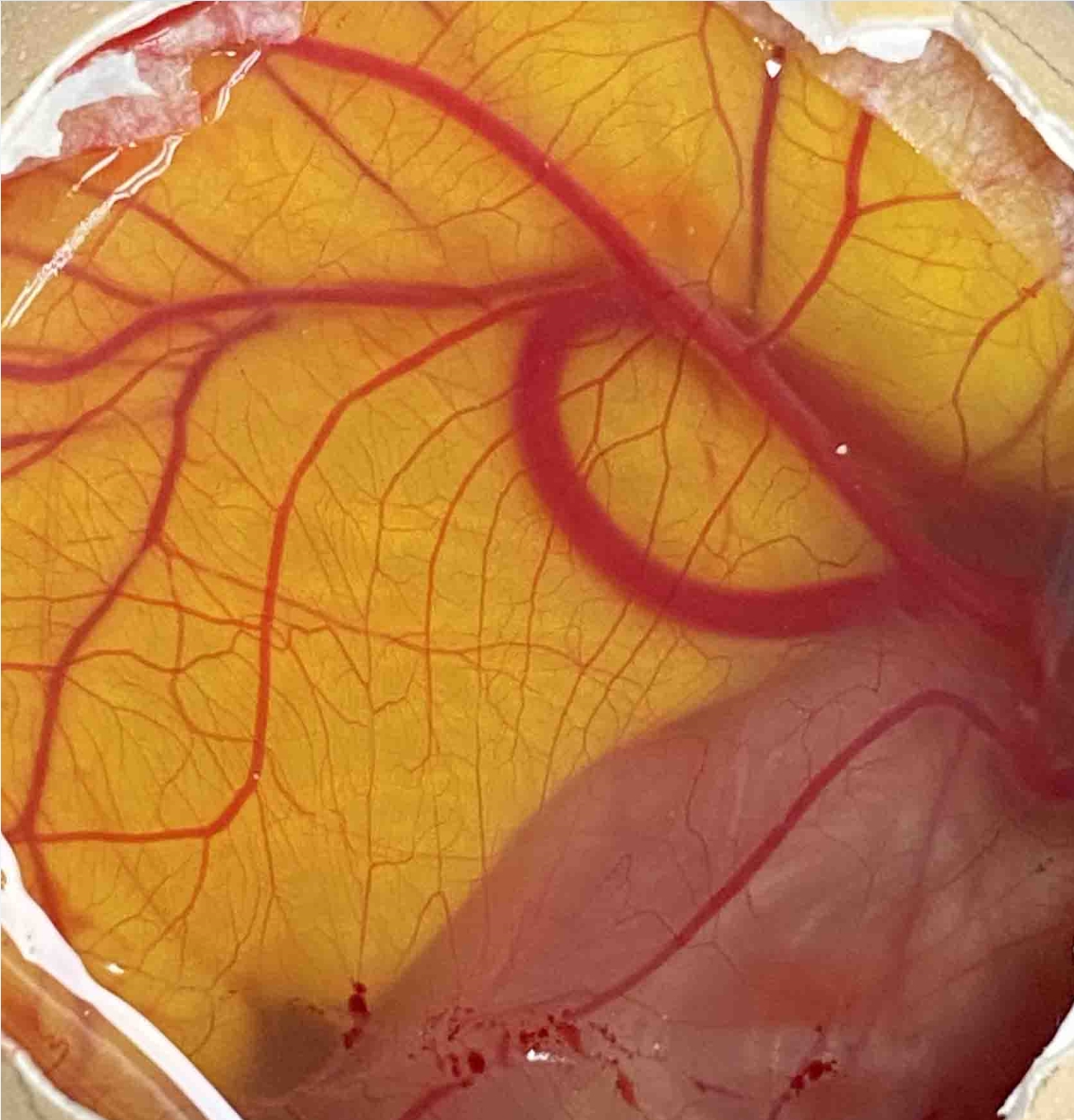

Supplement: Supplementary file 2 [file DataSheet3.zip › Figure 5 and 6/figure 5a/5MS.png]

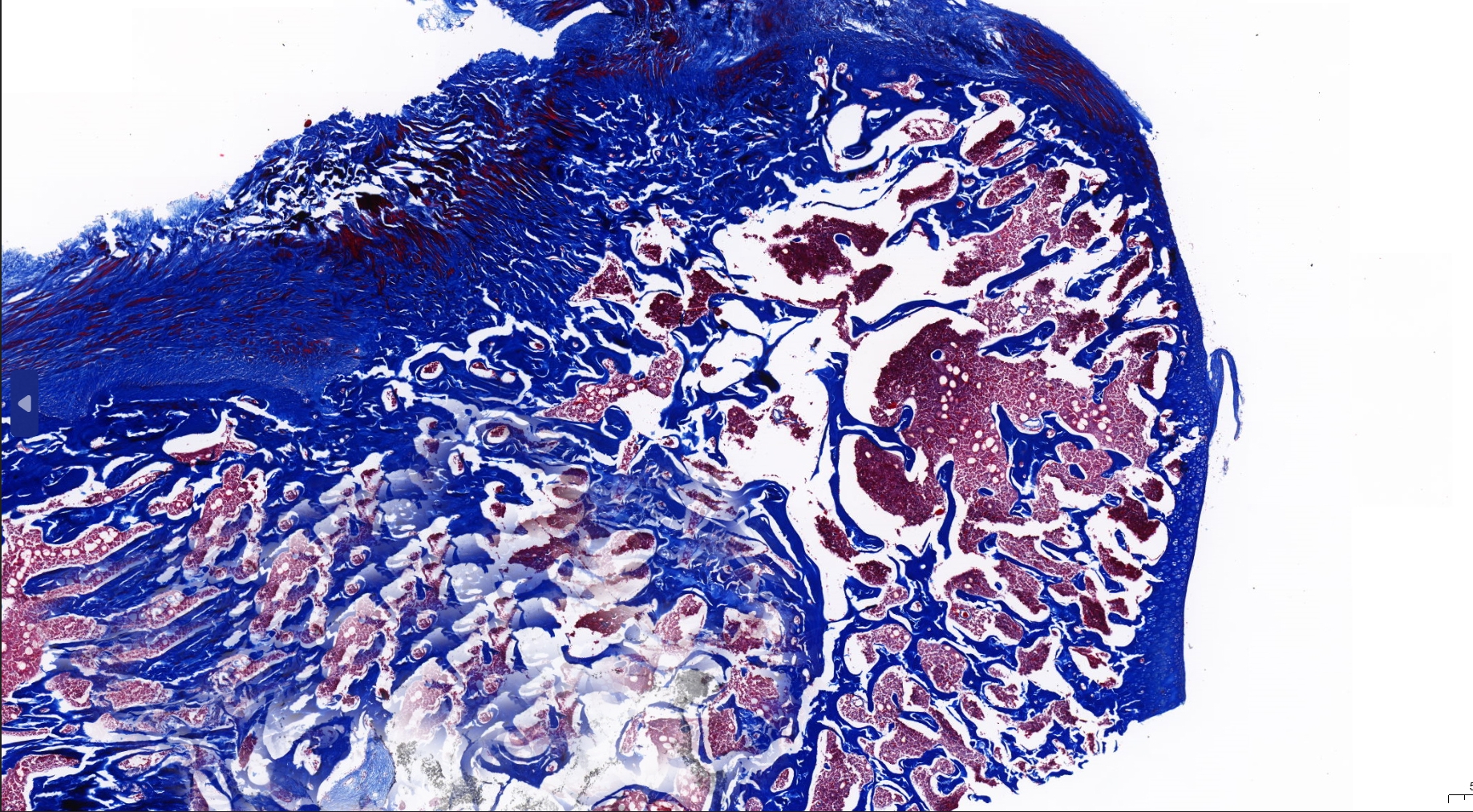

Supplement: Supplementary file 3 [file DataSheet8.zip › raw data_Figure 11B and C/Blank 12 W.png]

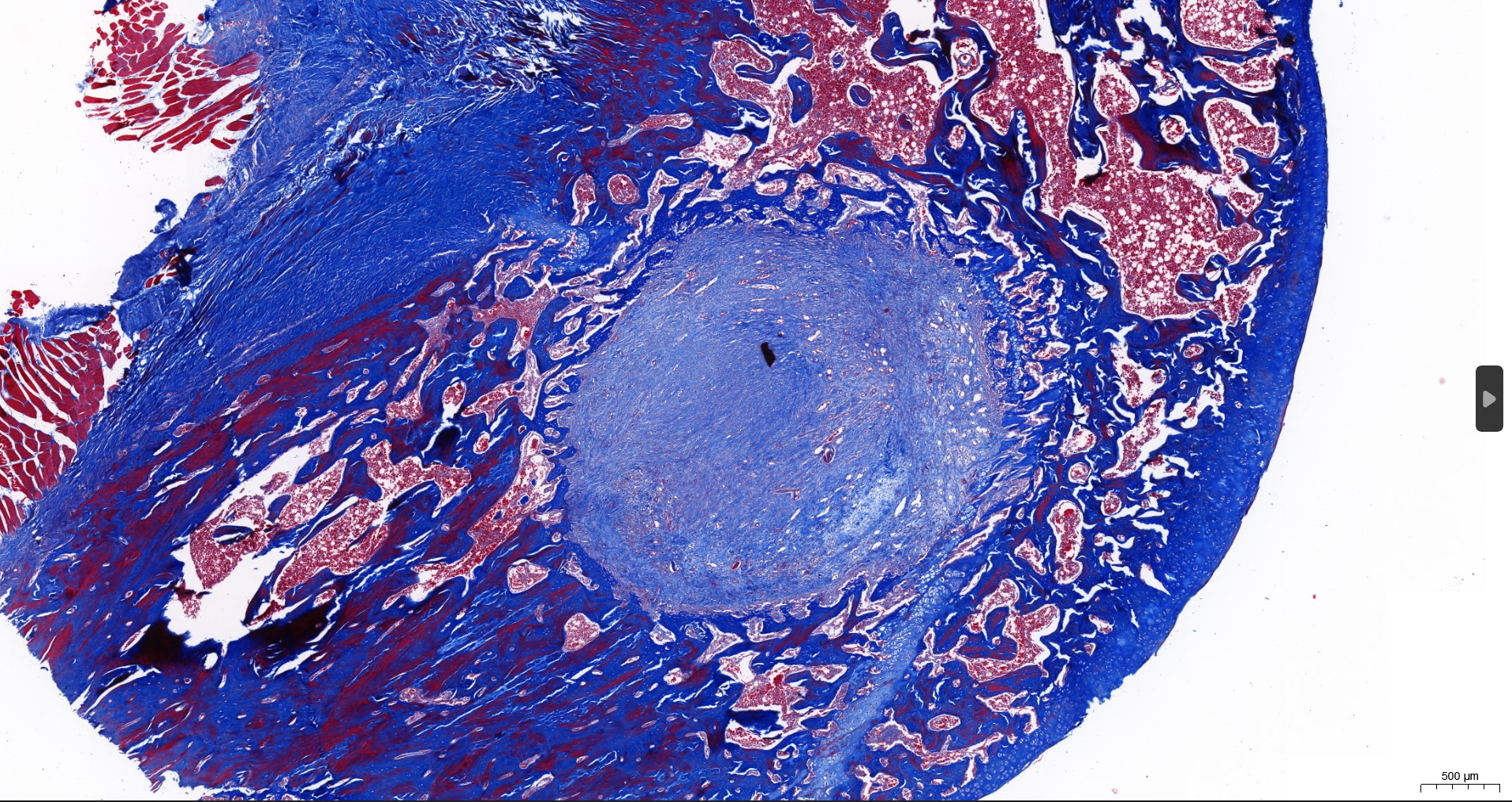

Supplement: Supplementary file 3 [file DataSheet8.zip › raw data_Figure 11B and C/Blank 4W.png]

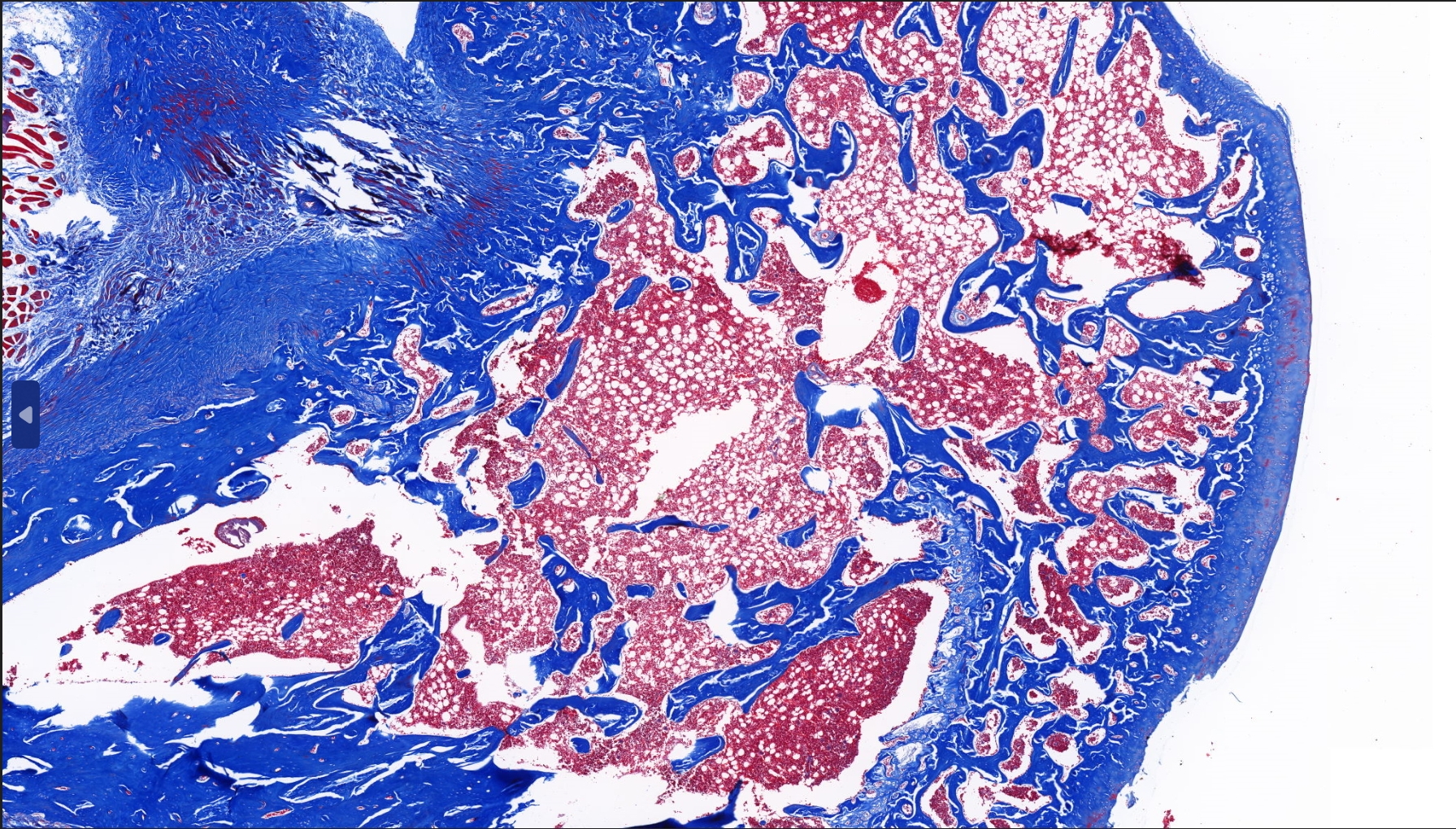

Supplement: Supplementary file 3 [file DataSheet8.zip › raw data_Figure 11B and C/Blank 8W.png]

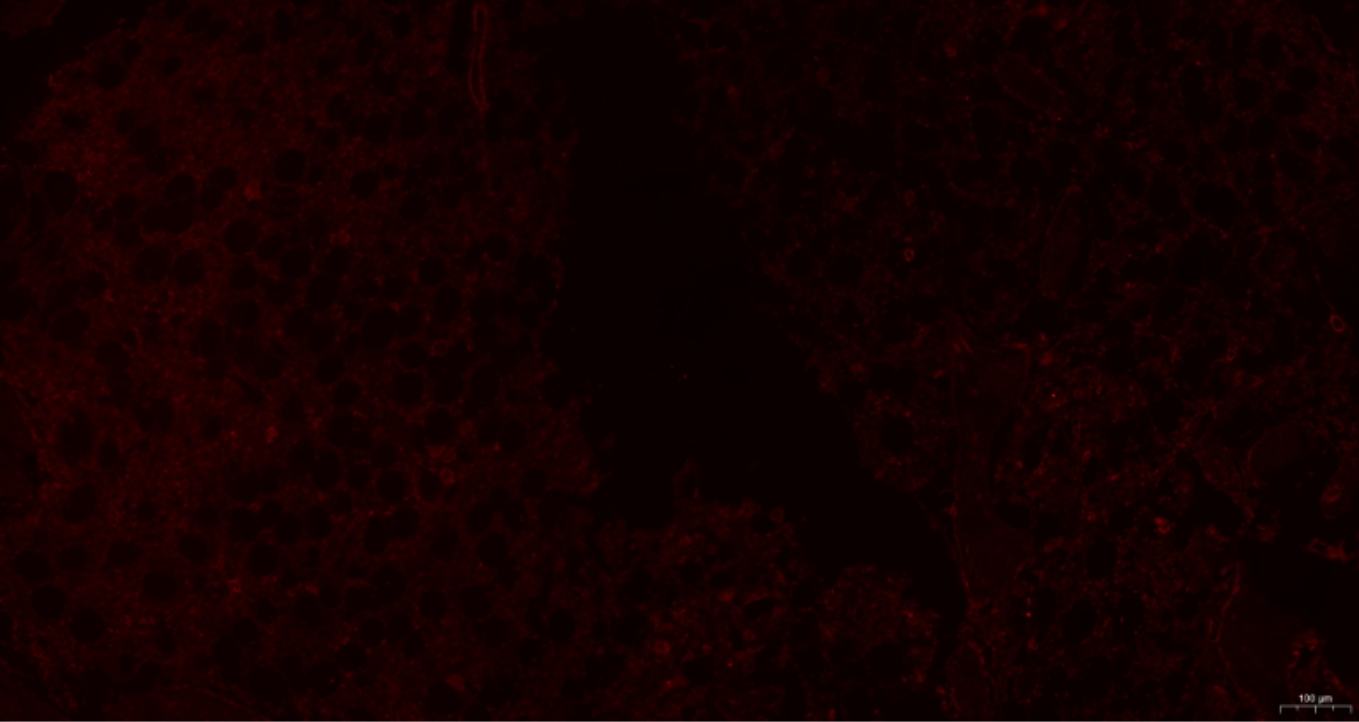

Supplement: Supplementary file 3 [file DataSheet8.zip › raw data_Figure 11B and C/C/Blank-CD31.png]

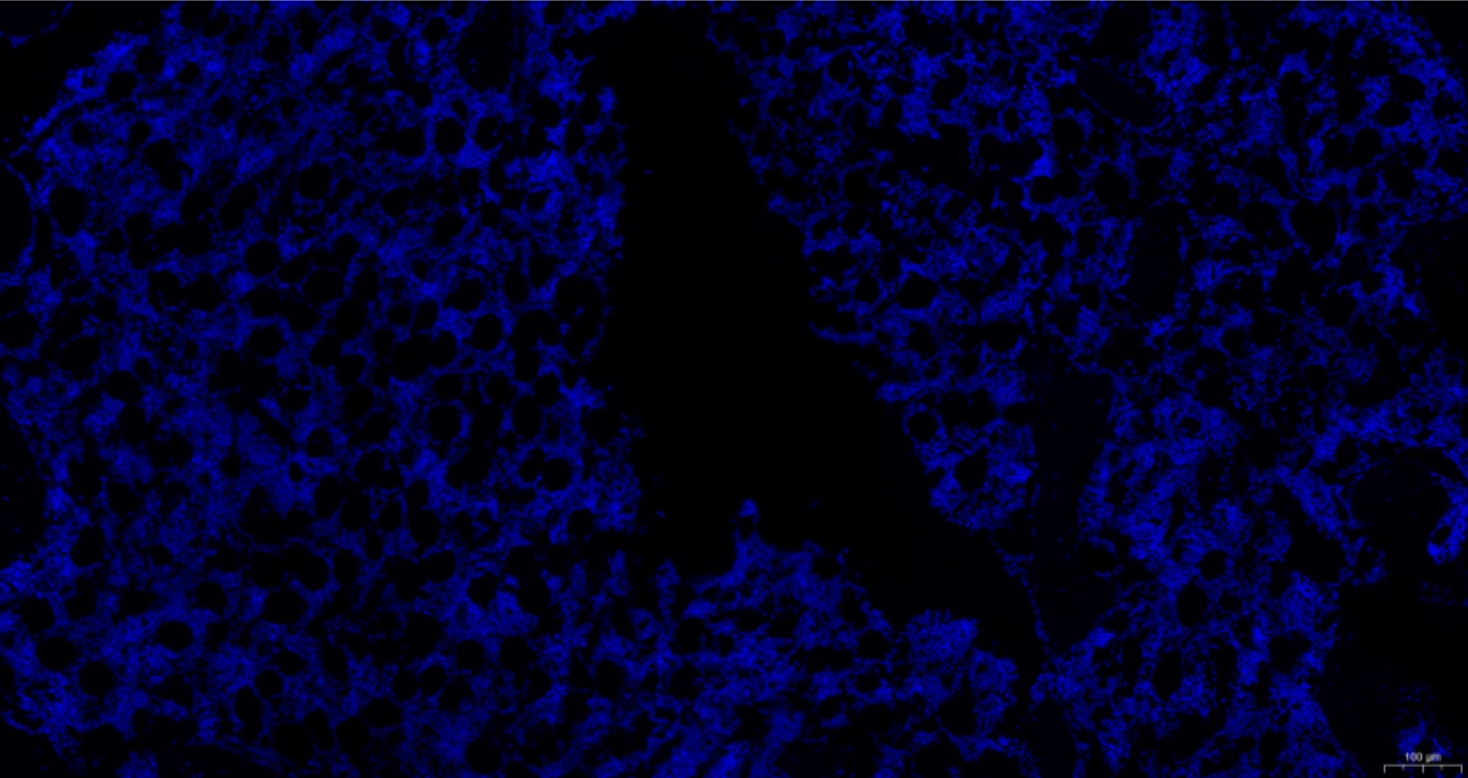

Supplement: Supplementary file 3 [file DataSheet8.zip › raw data_Figure 11B and C/C/Blank-DAPI.png]

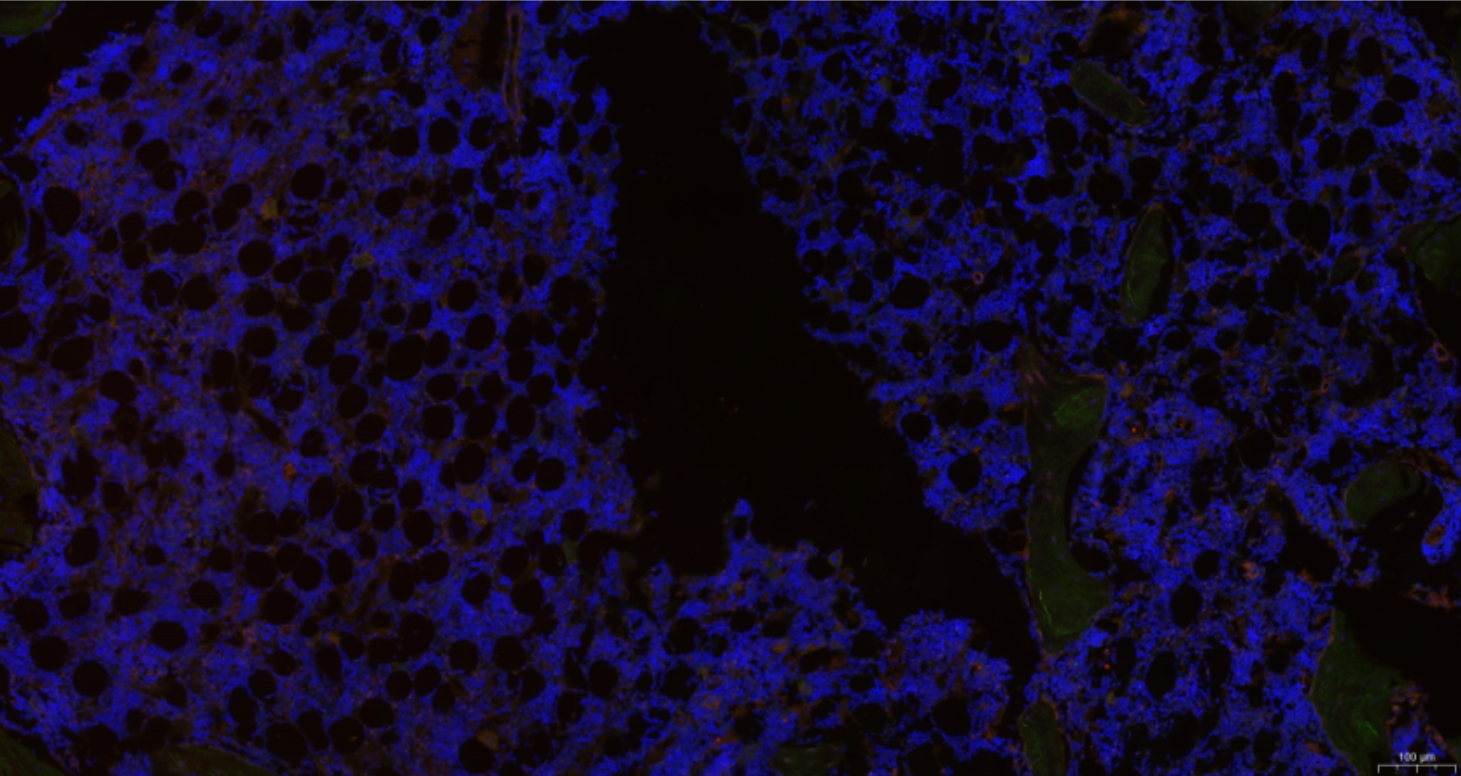

Supplement: Supplementary file 3 [file DataSheet8.zip › raw data_Figure 11B and C/C/Blank-Merge.png]

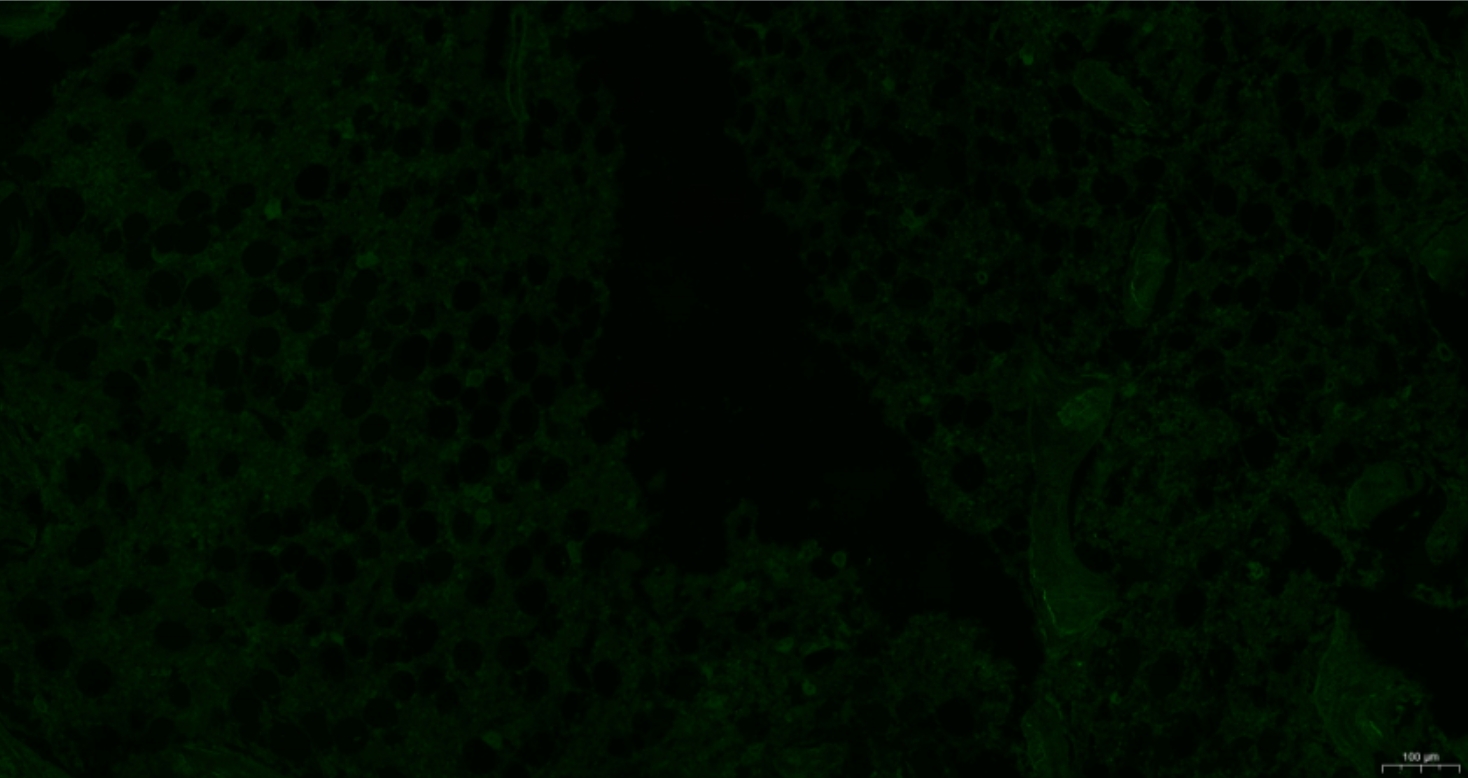

Supplement: Supplementary file 3 [file DataSheet8.zip › raw data_Figure 11B and C/C/Blank-vWF.png]

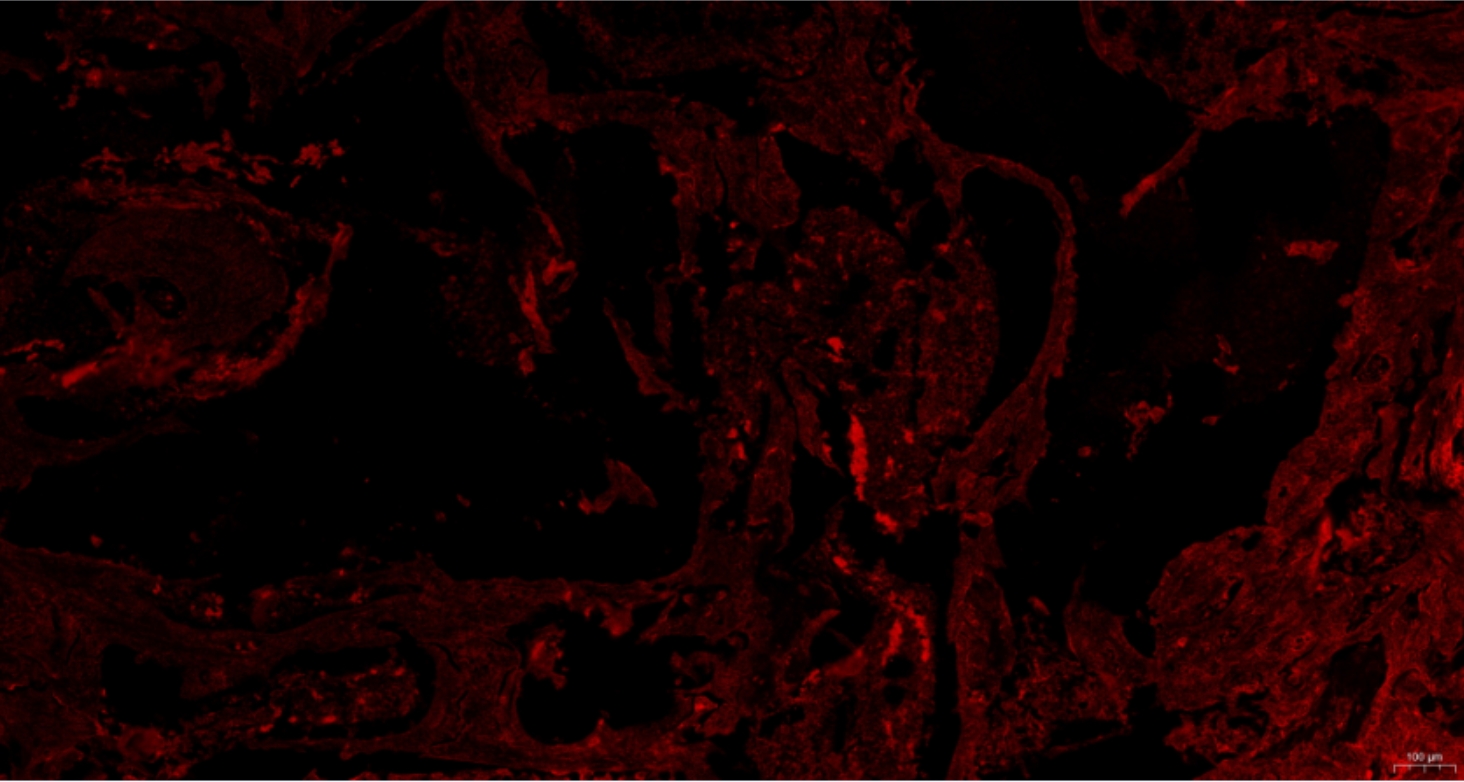

Supplement: Supplementary file 3 [file DataSheet8.zip › raw data_Figure 11B and C/C/MS-CD31.png]

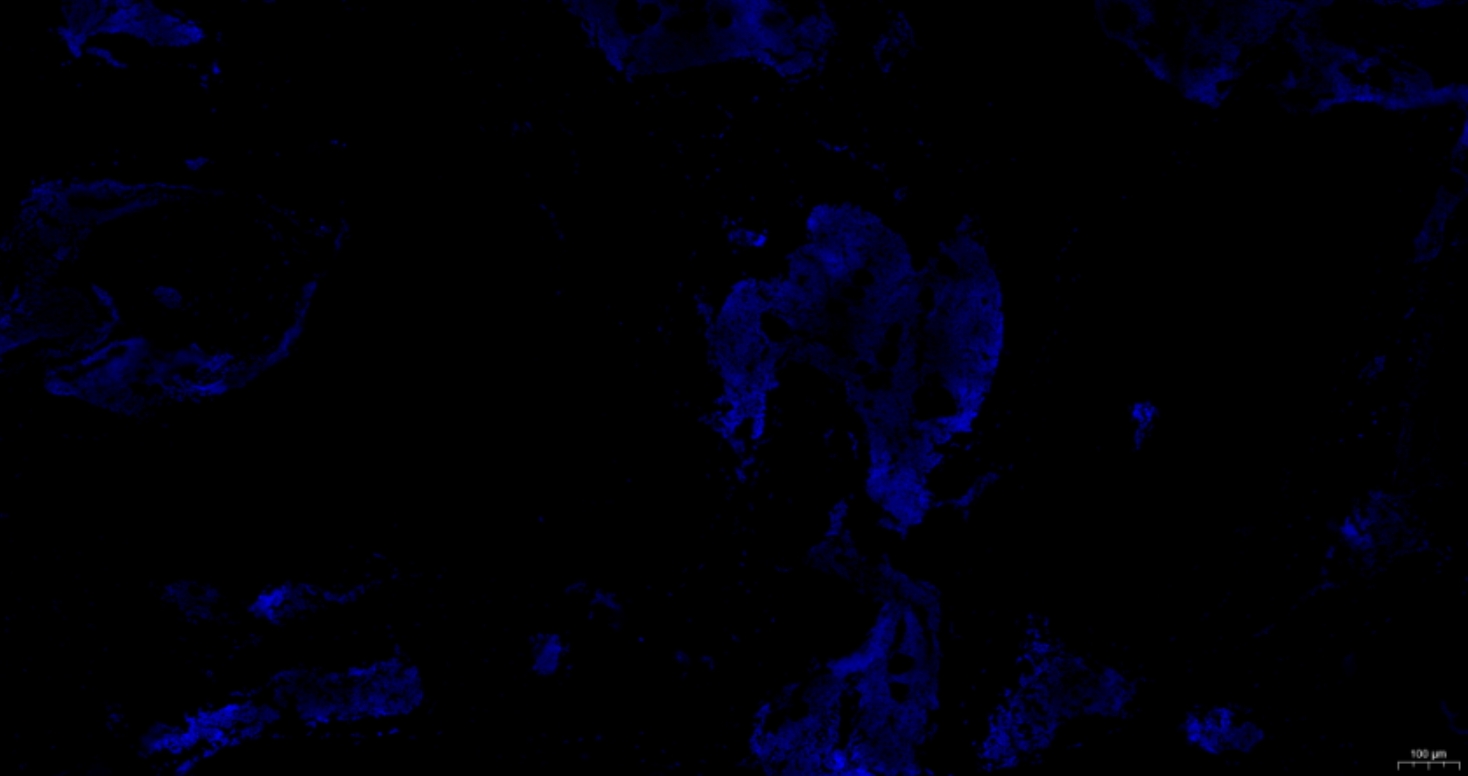

Supplement: Supplementary file 3 [file DataSheet8.zip › raw data_Figure 11B and C/C/MS-DAPI.png]

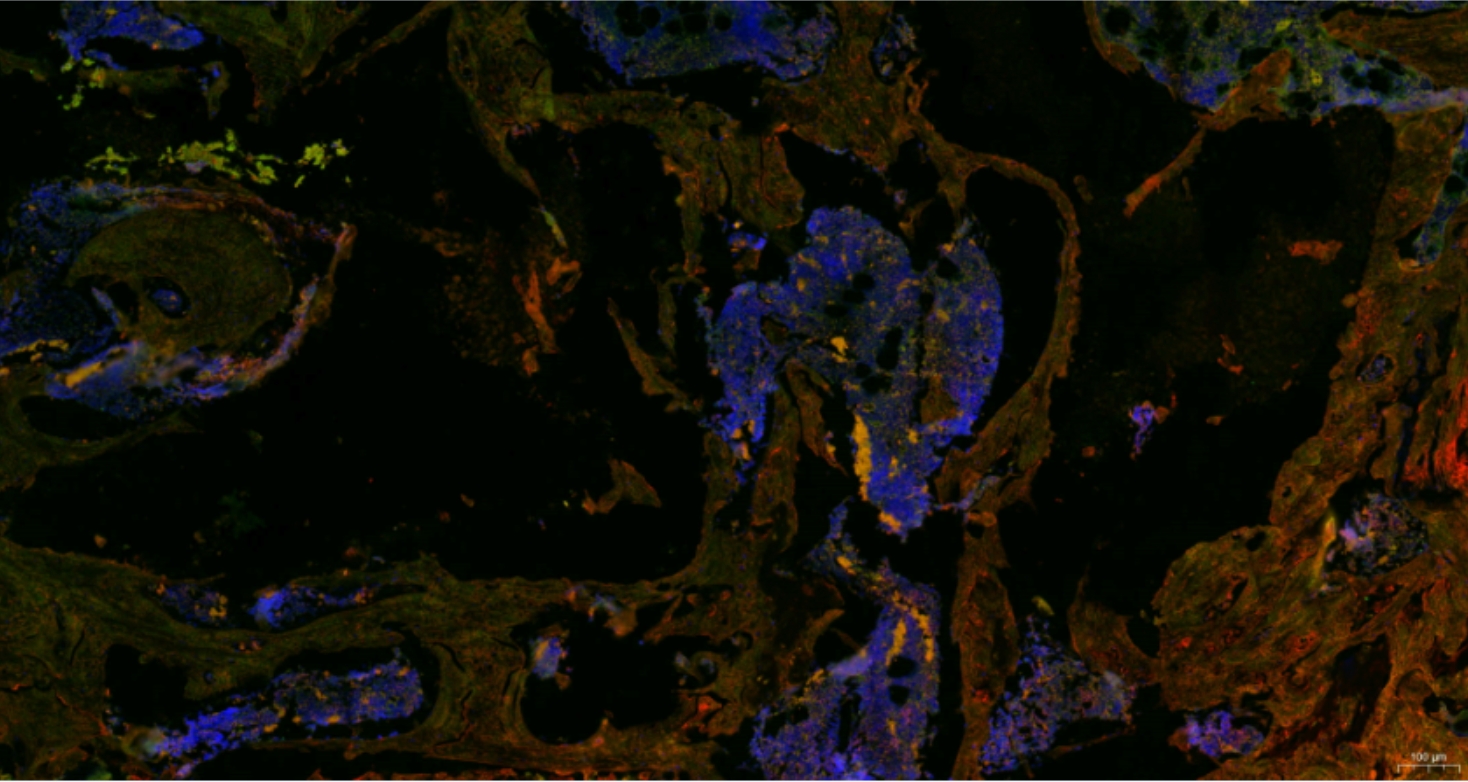

Supplement: Supplementary file 3 [file DataSheet8.zip › raw data_Figure 11B and C/C/MS-MERGE.png]

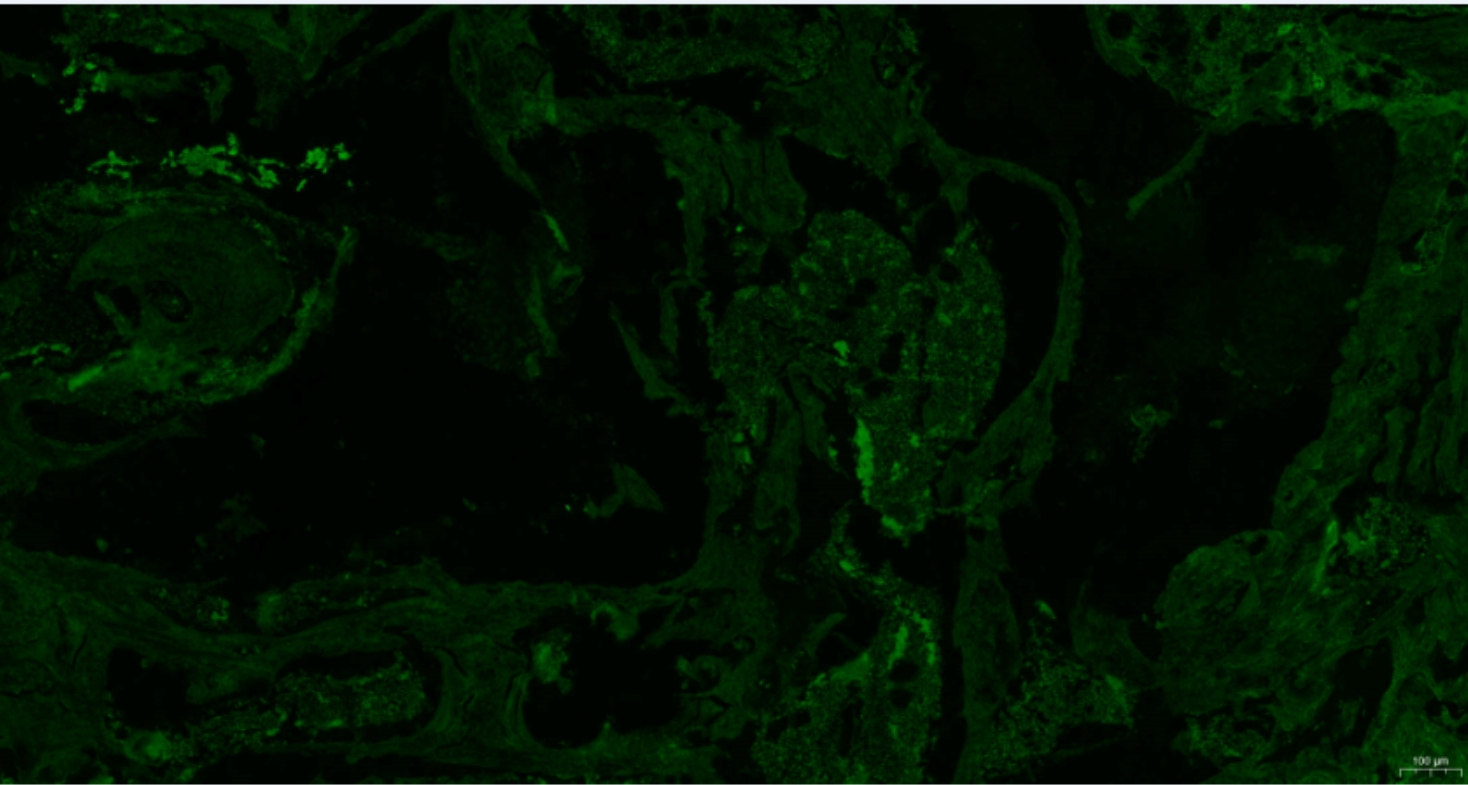

Supplement: Supplementary file 3 [file DataSheet8.zip › raw data_Figure 11B and C/C/MS-VWF.png]

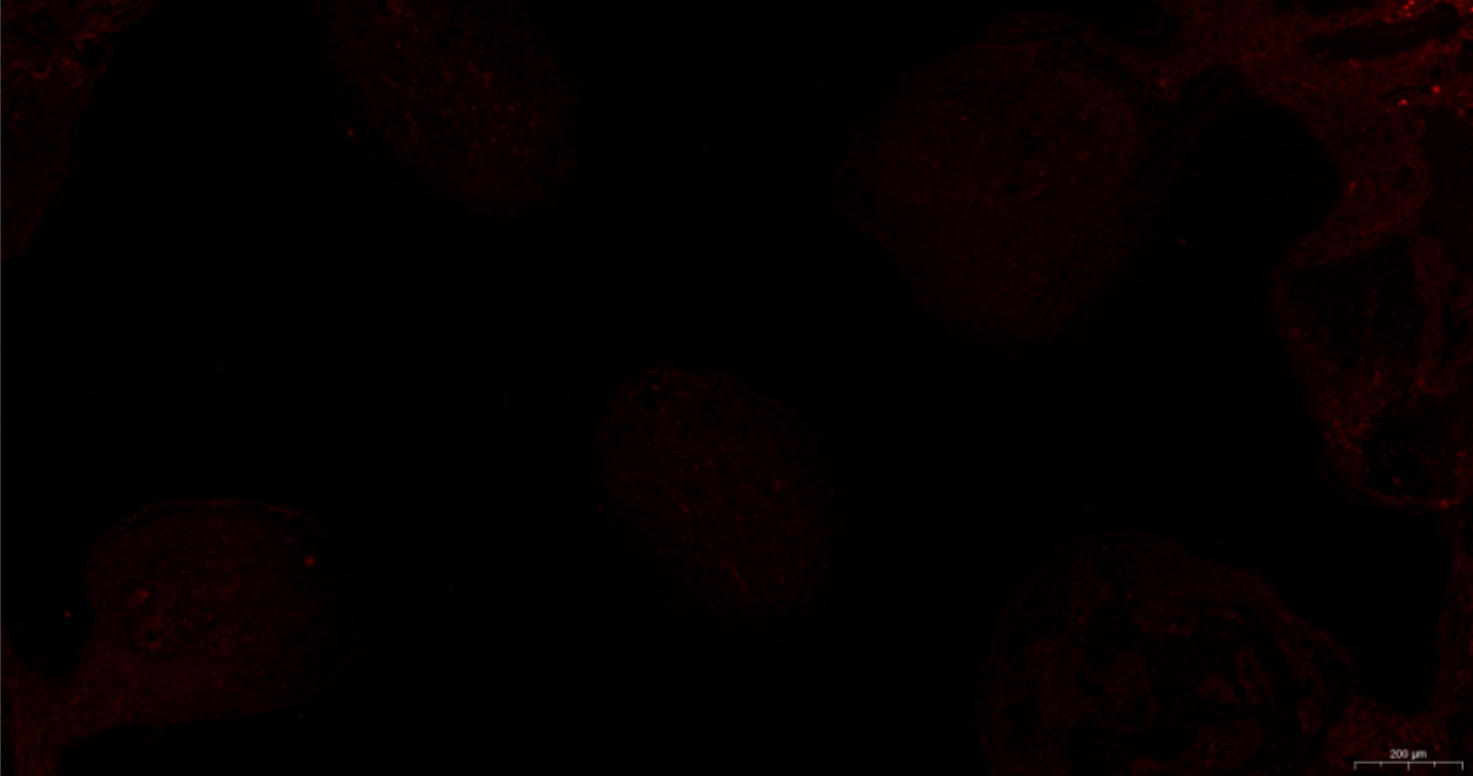

Supplement: Supplementary file 3 [file DataSheet8.zip › raw data_Figure 11B and C/C/TCP-CD31.png]

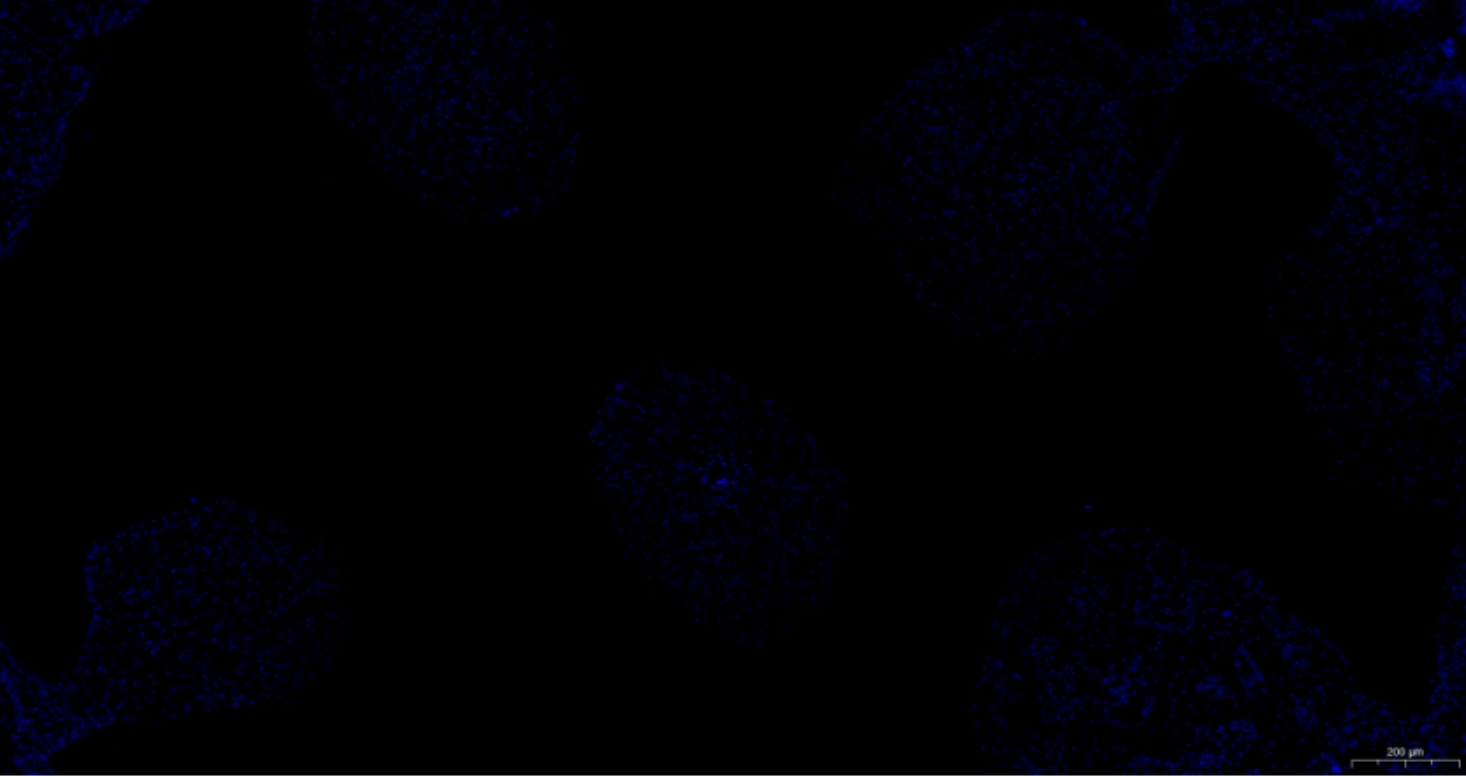

Supplement: Supplementary file 3 [file DataSheet8.zip › raw data_Figure 11B and C/C/TCP-DAPI.png]

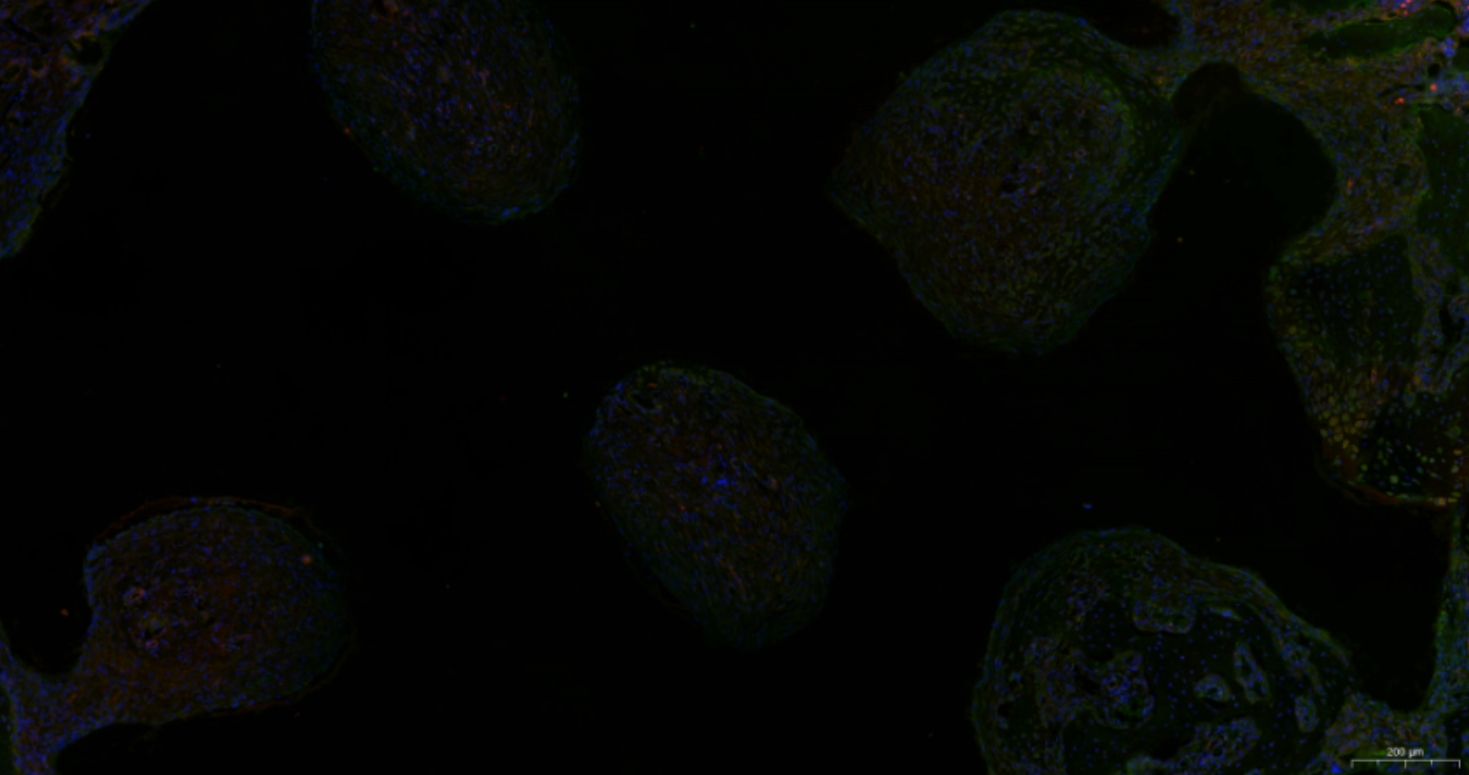

Supplement: Supplementary file 3 [file DataSheet8.zip › raw data_Figure 11B and C/C/TCP-MERGE.png]

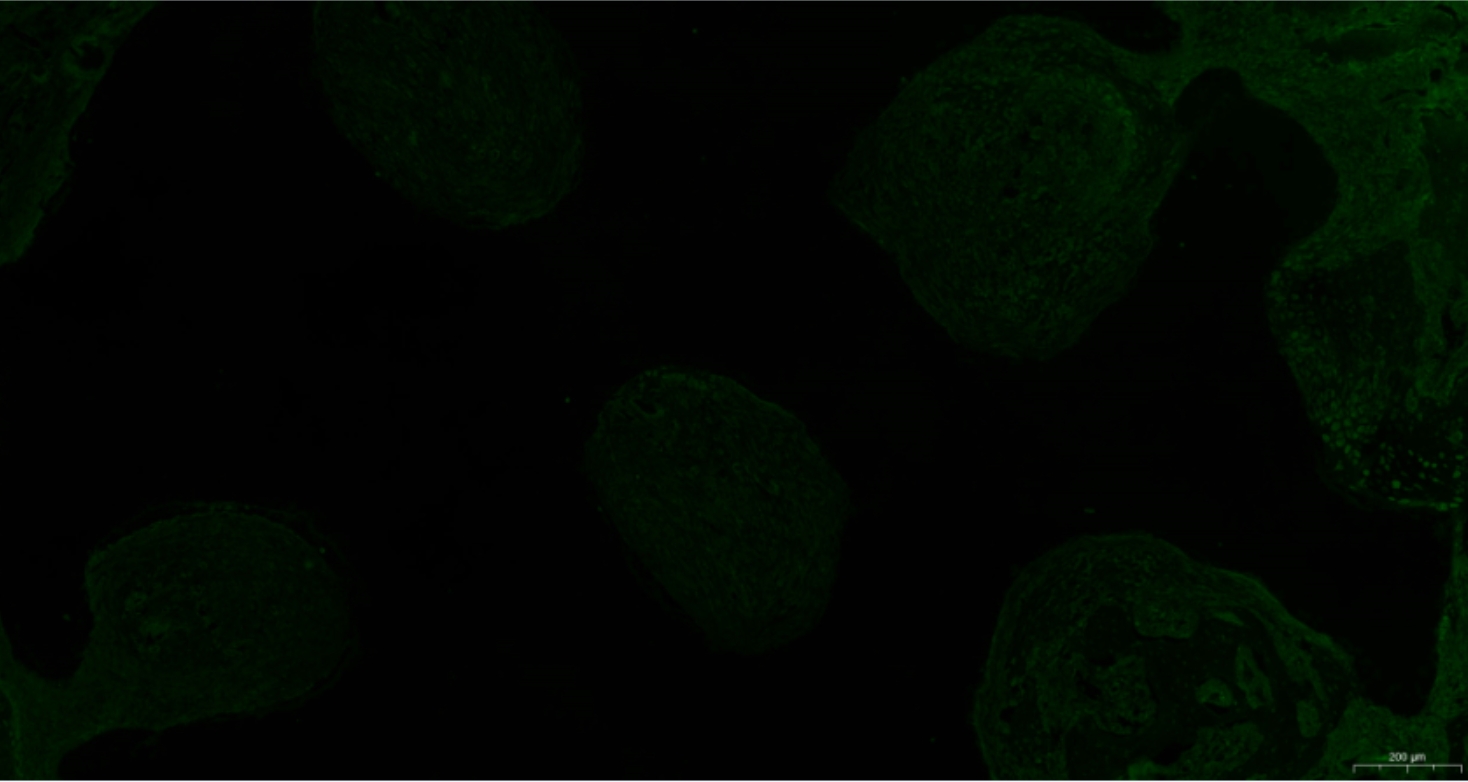

Supplement: Supplementary file 3 [file DataSheet8.zip › raw data_Figure 11B and C/C/TCP-VWF.png]

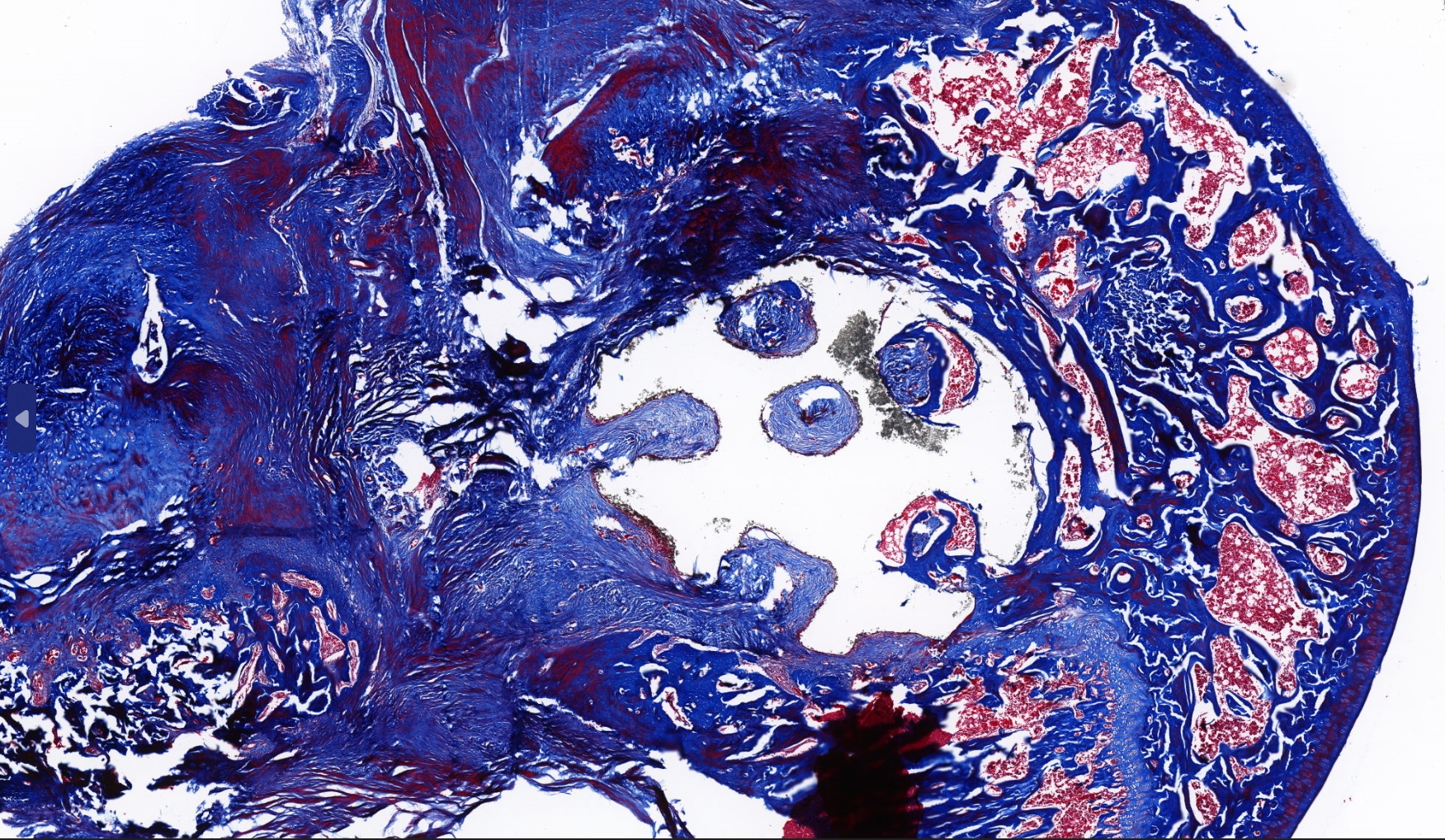

Supplement: Supplementary file 3 [file DataSheet8.zip › raw data_Figure 11B and C/MS 12W.png]

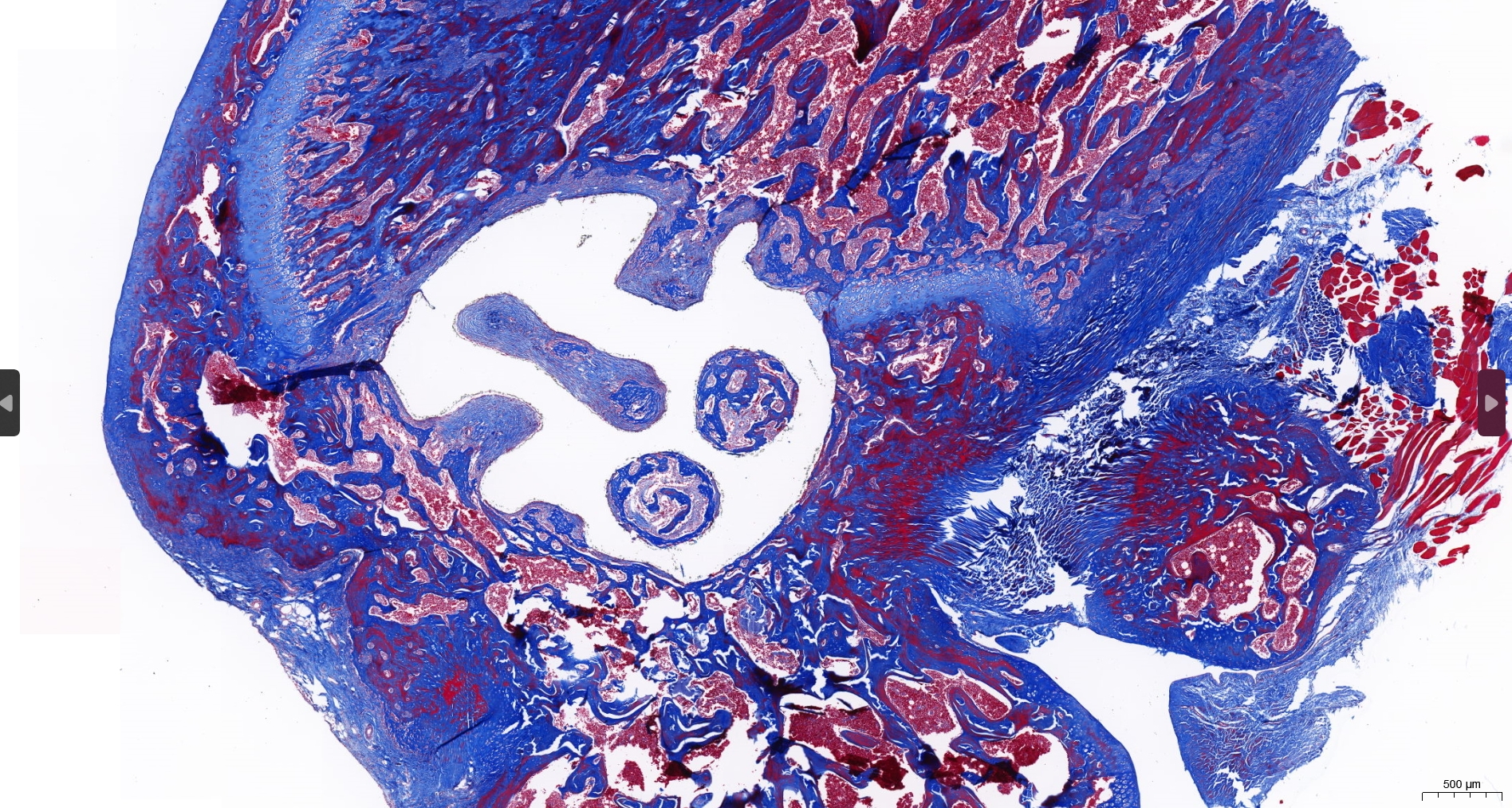

Supplement: Supplementary file 3 [file DataSheet8.zip › raw data_Figure 11B and C/MS 4W.png]

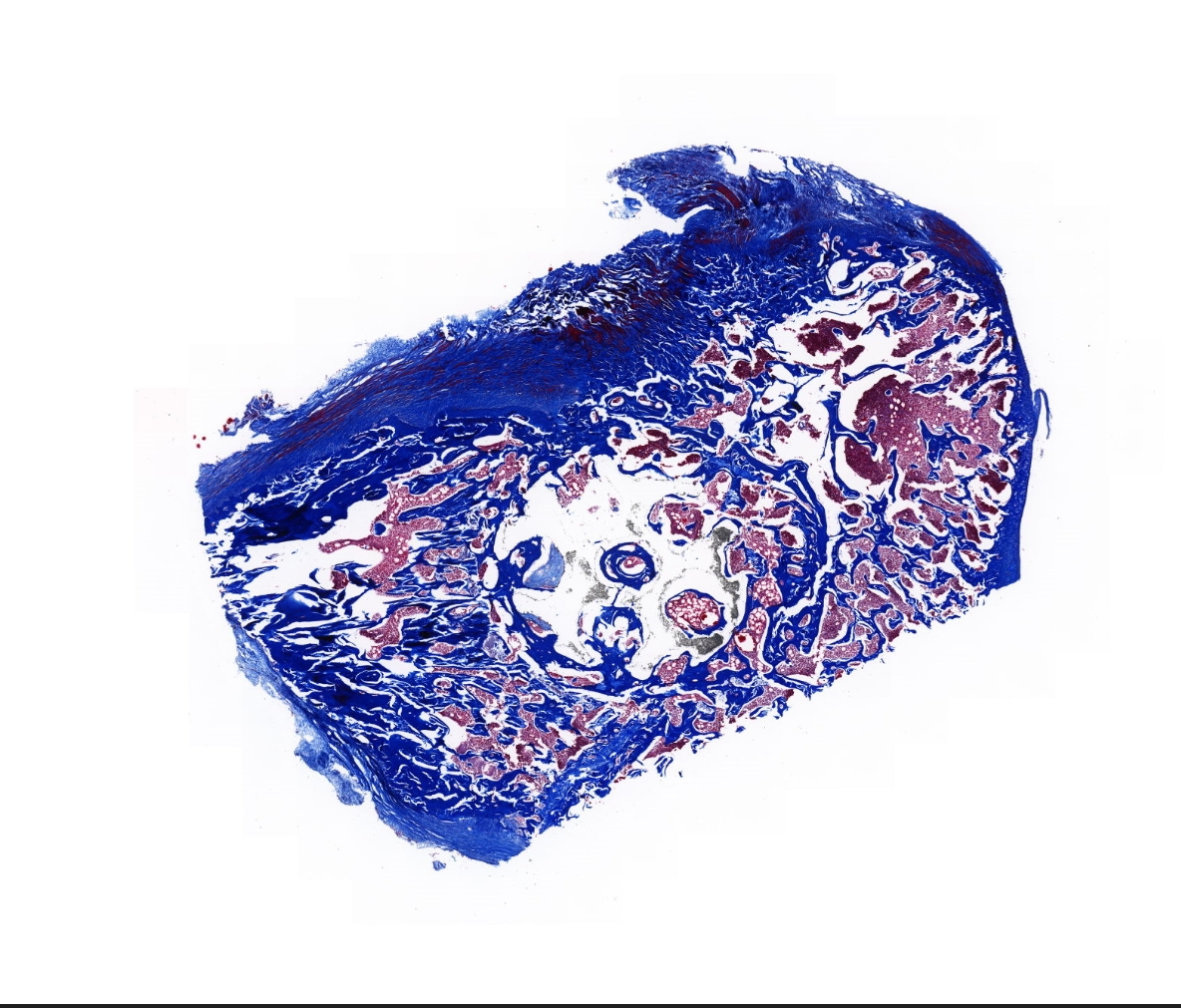

Supplement: Supplementary file 3 [file DataSheet8.zip › raw data_Figure 11B and C/MS 8W.png]

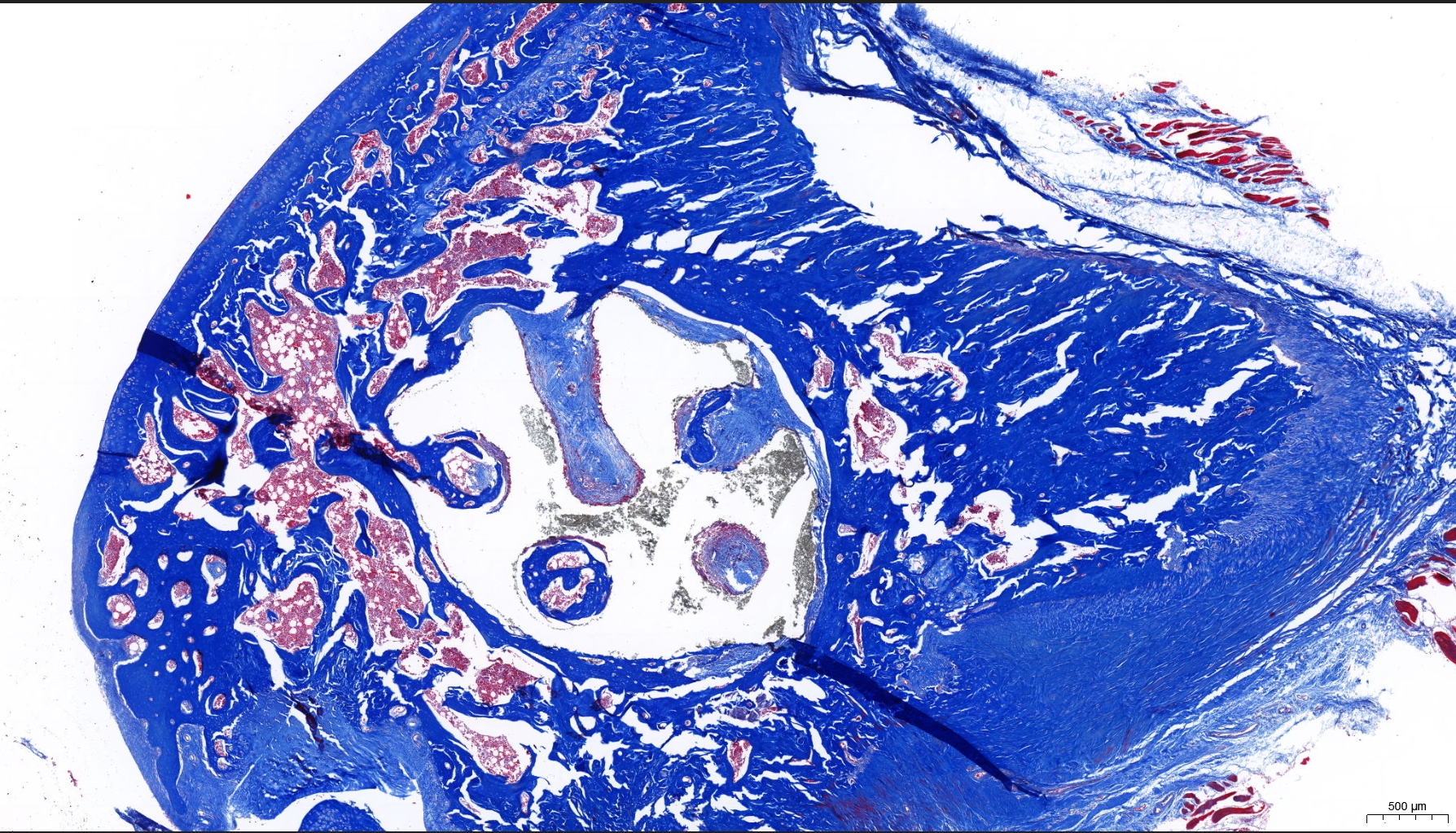

Supplement: Supplementary file 3 [file DataSheet8.zip › raw data_Figure 11B and C/TCP 12W.png]

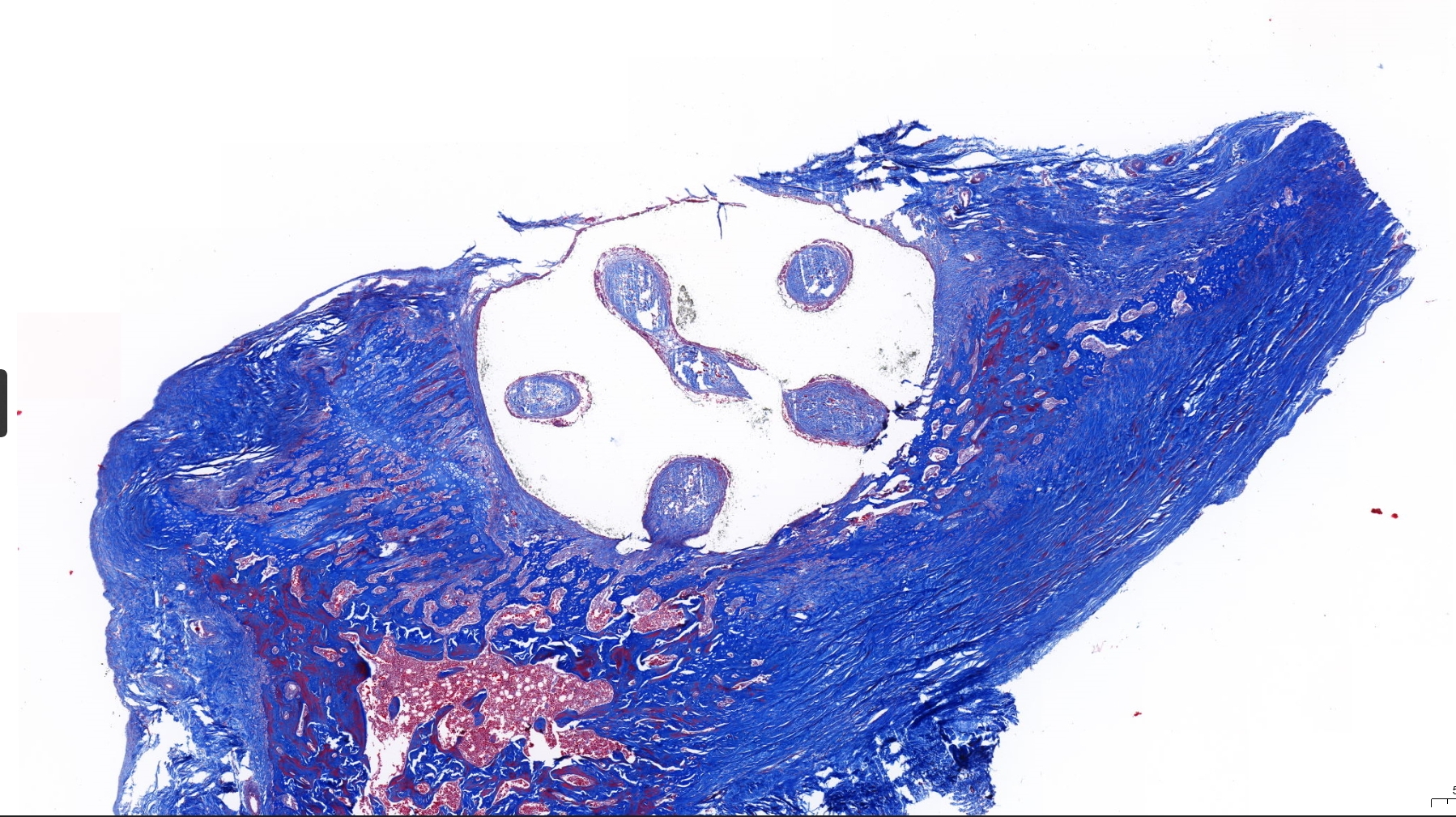

Supplement: Supplementary file 3 [file DataSheet8.zip › raw data_Figure 11B and C/TCP 4W.png]

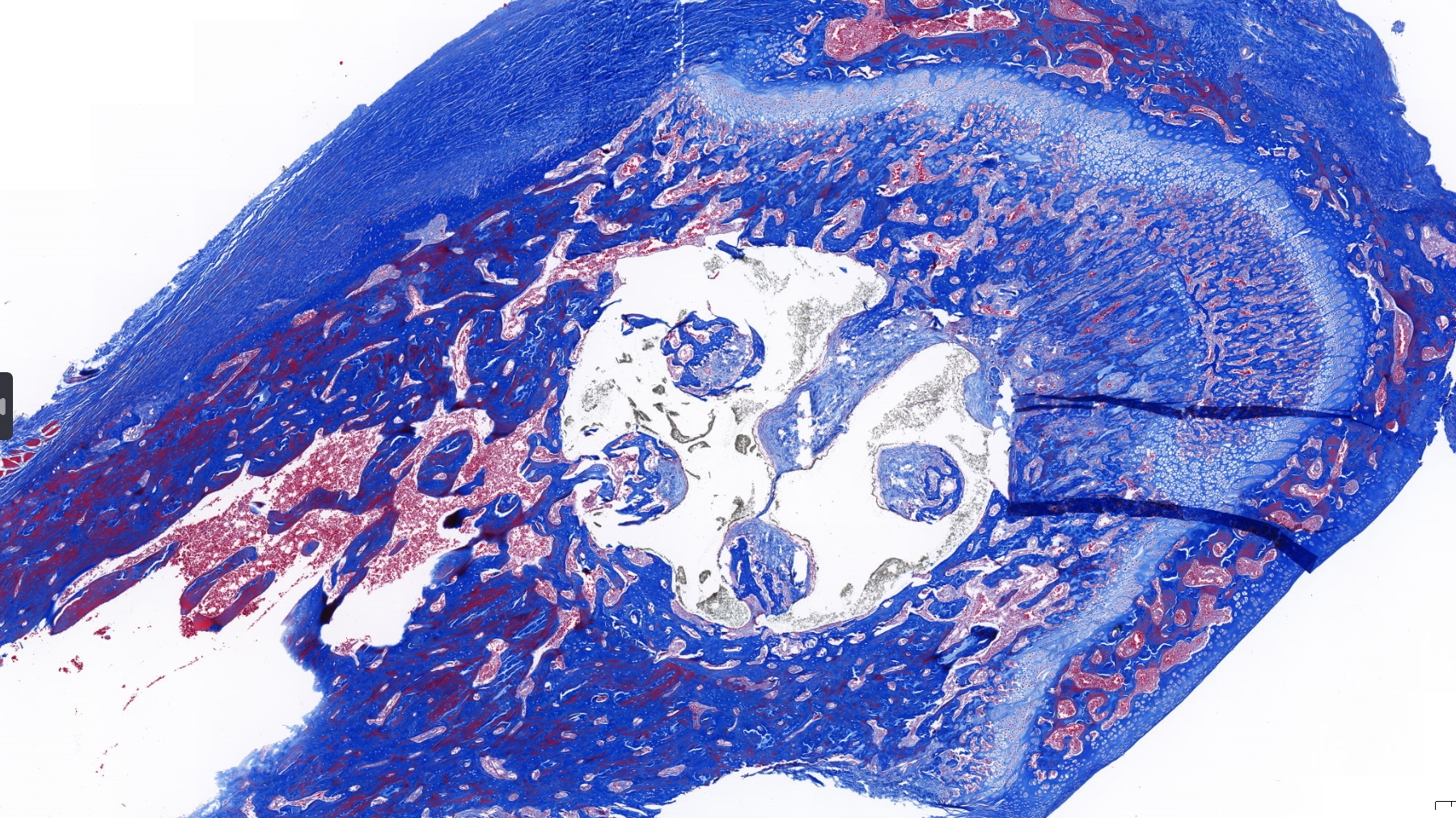

Supplement: Supplementary file 3 [file DataSheet8.zip › raw data_Figure 11B and C/TCP 8W.png]

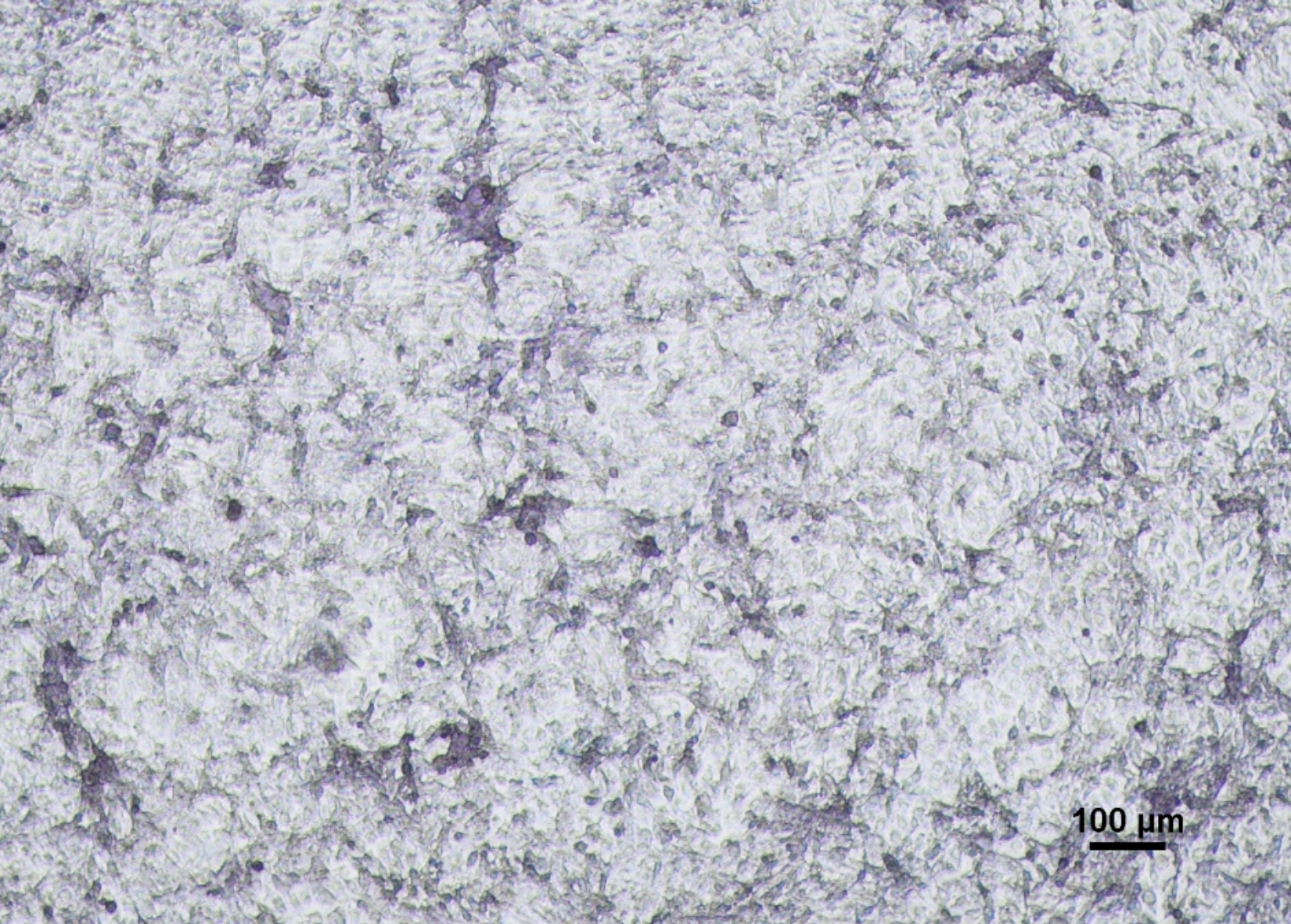

Supplement: Supplementary file 5 [file DataSheet4.zip › raw data_Figure8A C F/A/DAY14 LY294002.png]

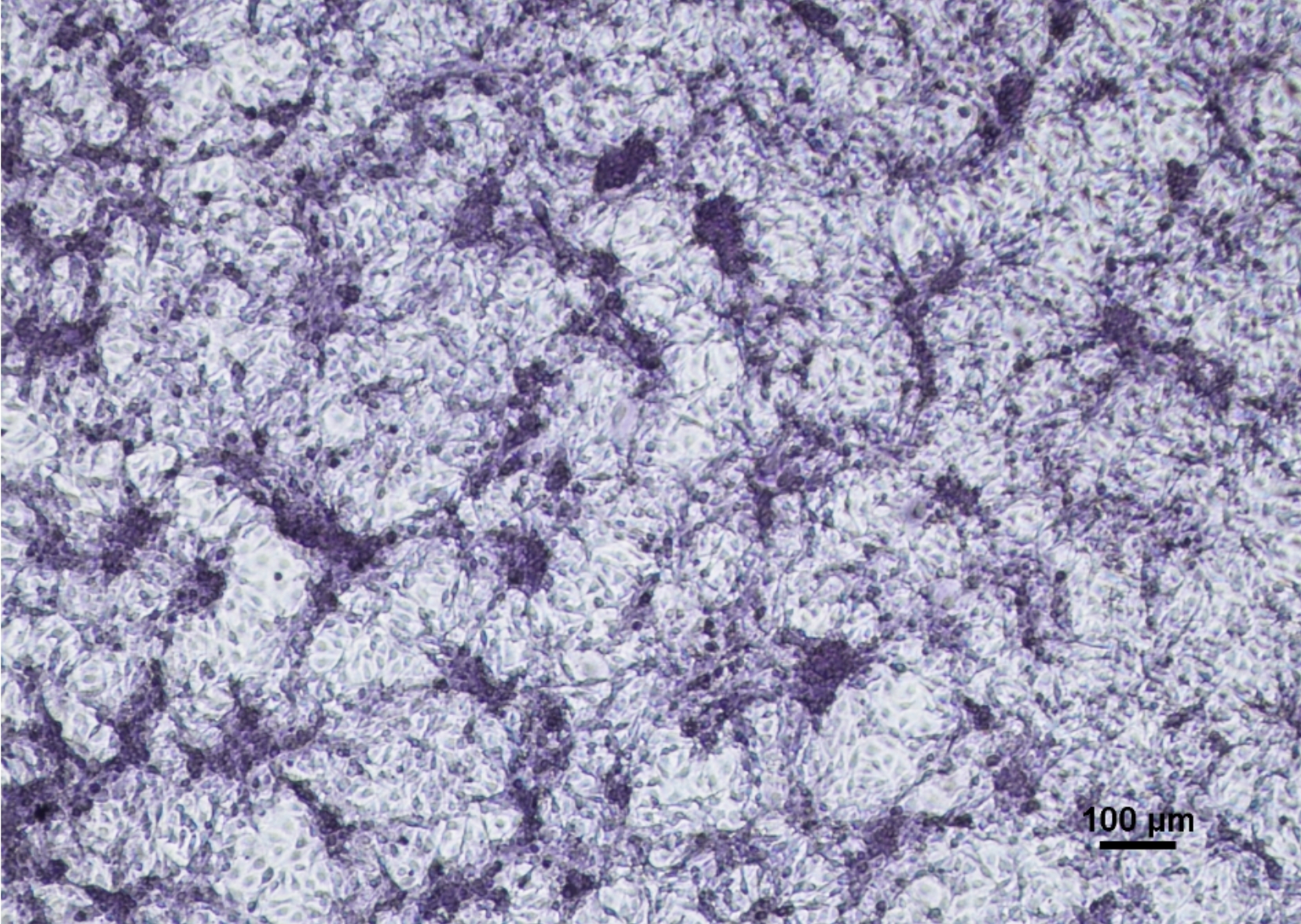

Supplement: Supplementary file 5 [file DataSheet4.zip › raw data_Figure8A C F/A/DAY14 MS.png]

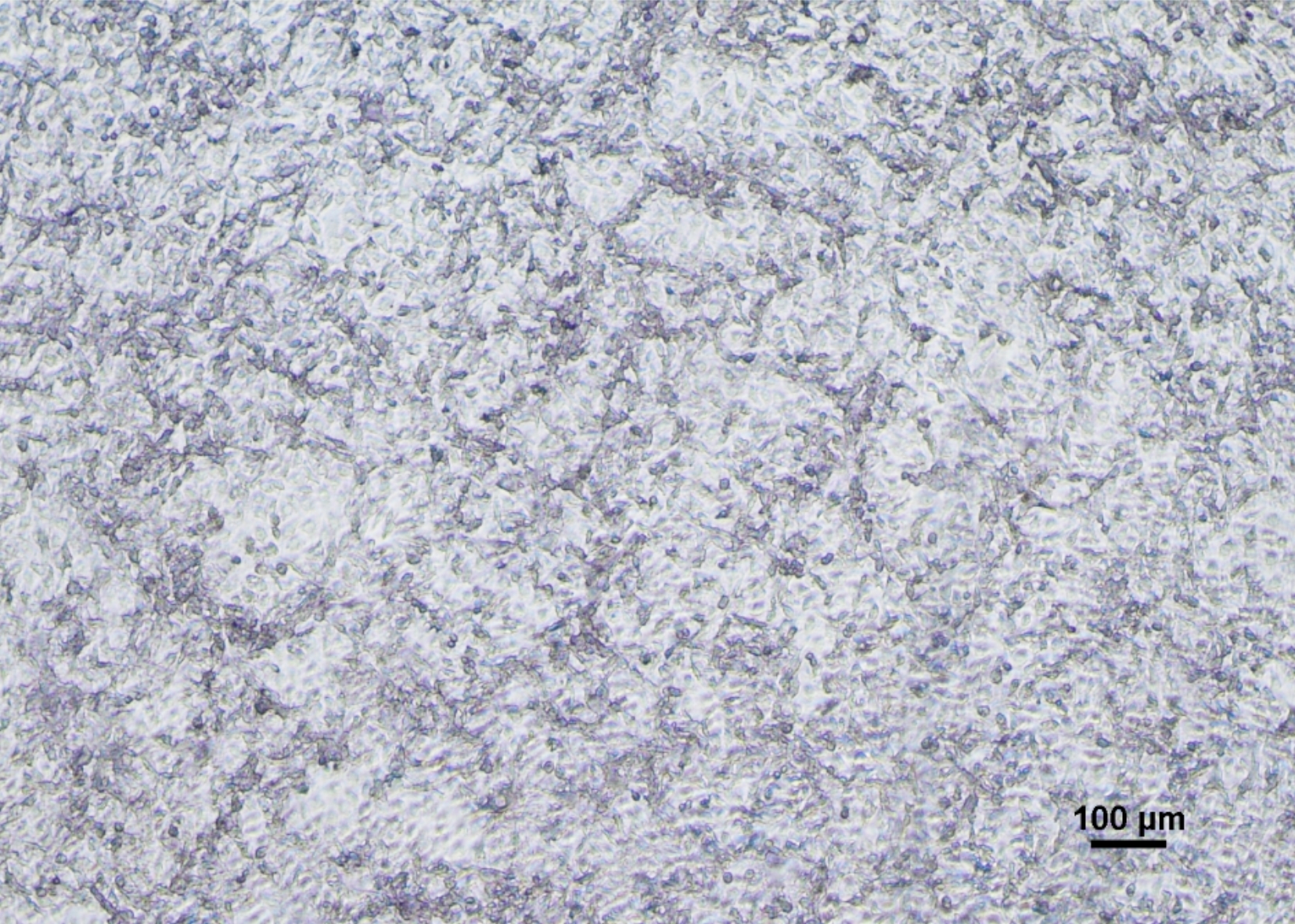

Supplement: Supplementary file 5 [file DataSheet4.zip › raw data_Figure8A C F/A/DAY7 LY294002.png]

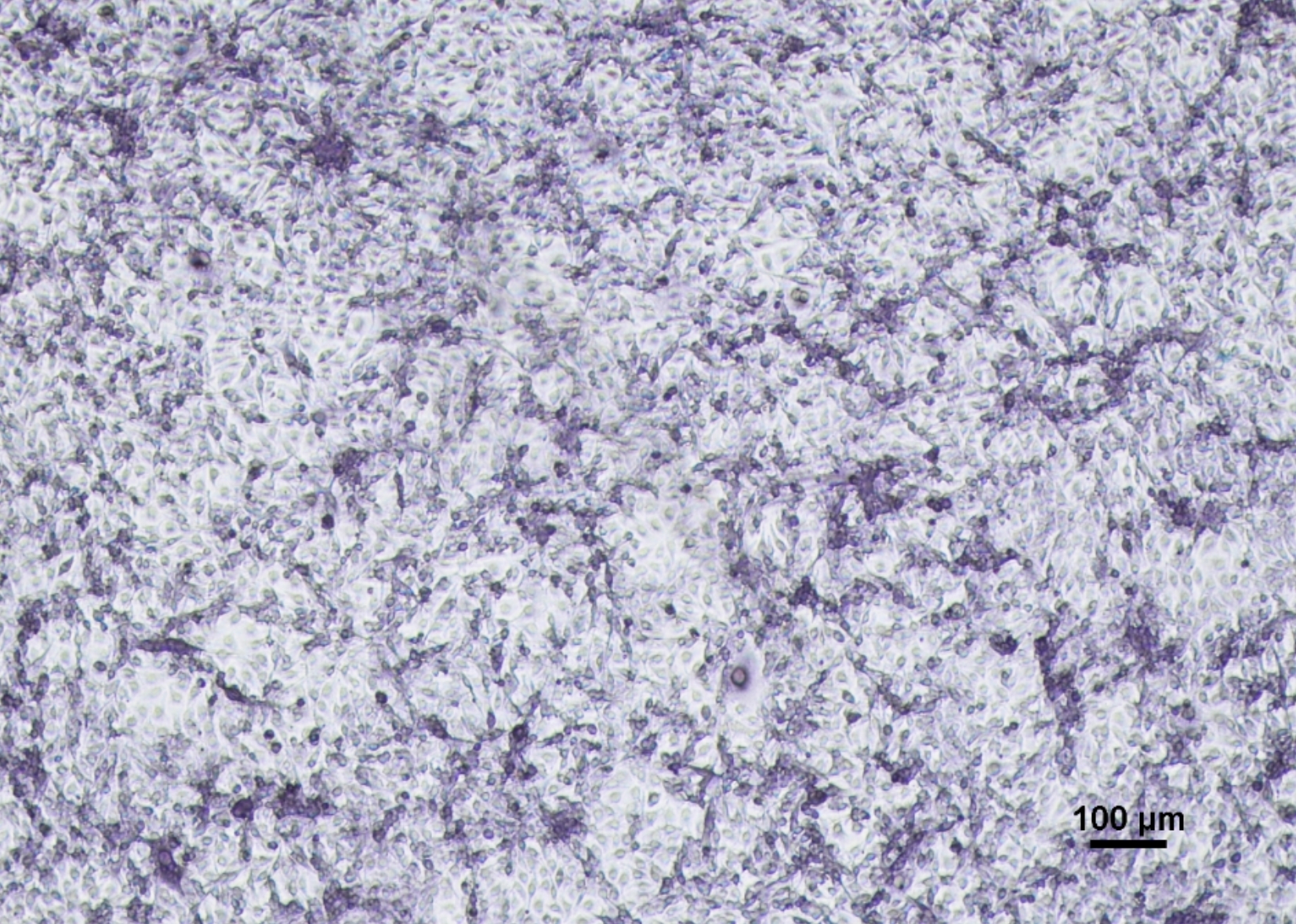

Supplement: Supplementary file 5 [file DataSheet4.zip › raw data_Figure8A C F/A/DAY7 MS.png]

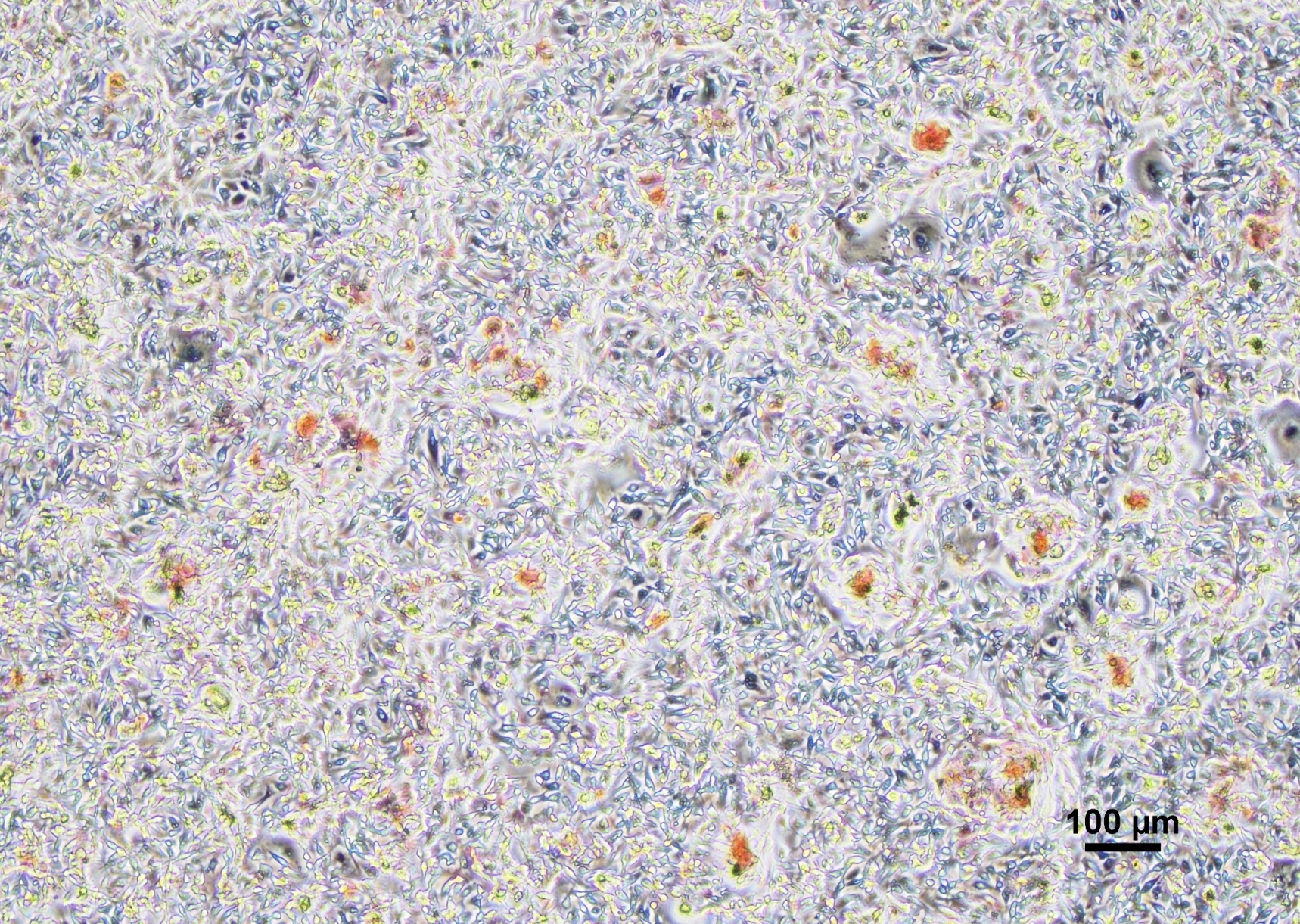

Supplement: Supplementary file 5 [file DataSheet4.zip › raw data_Figure8A C F/C/DAY14 LY294002.png]

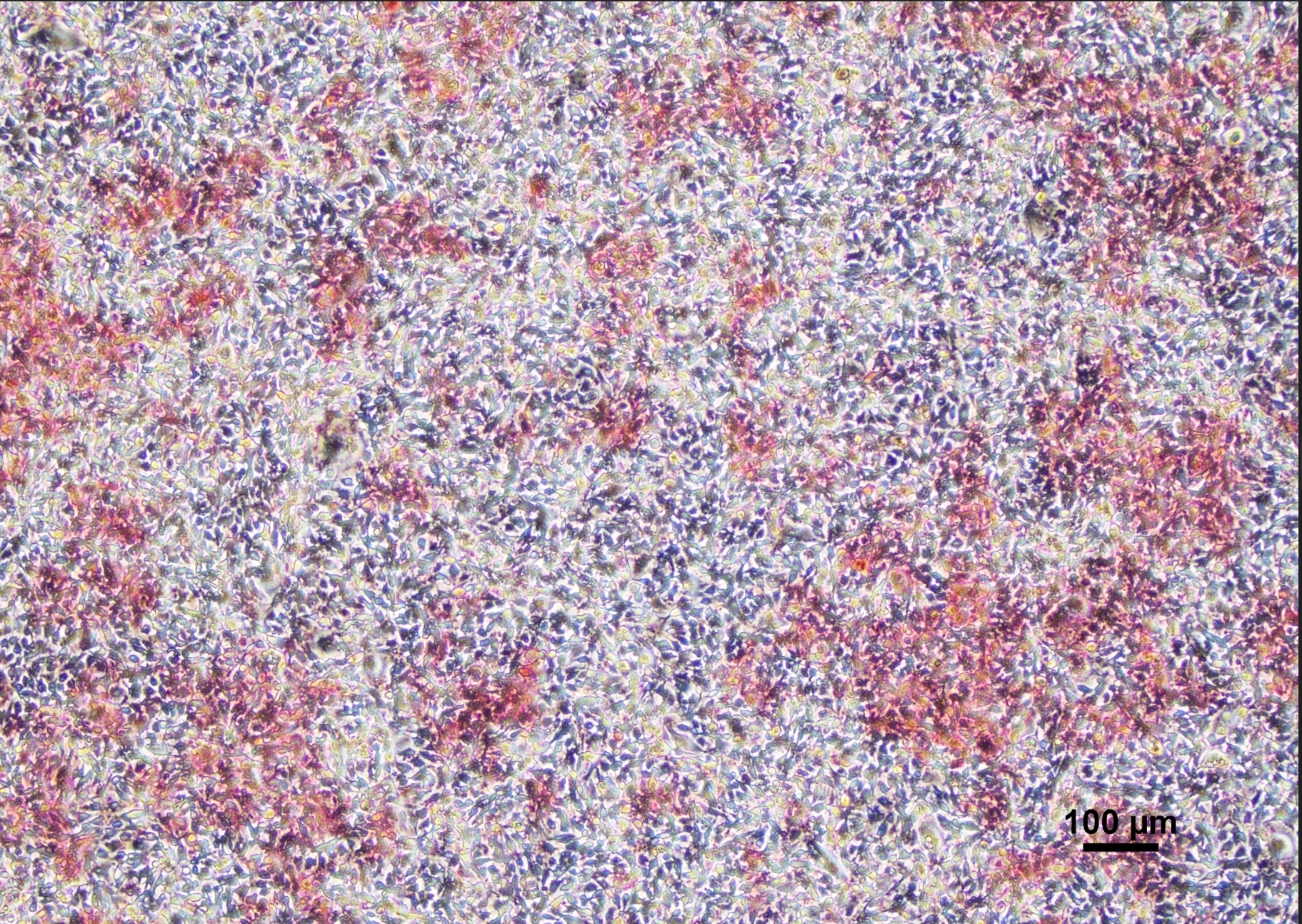

Supplement: Supplementary file 5 [file DataSheet4.zip › raw data_Figure8A C F/C/DAY14 MS.png]

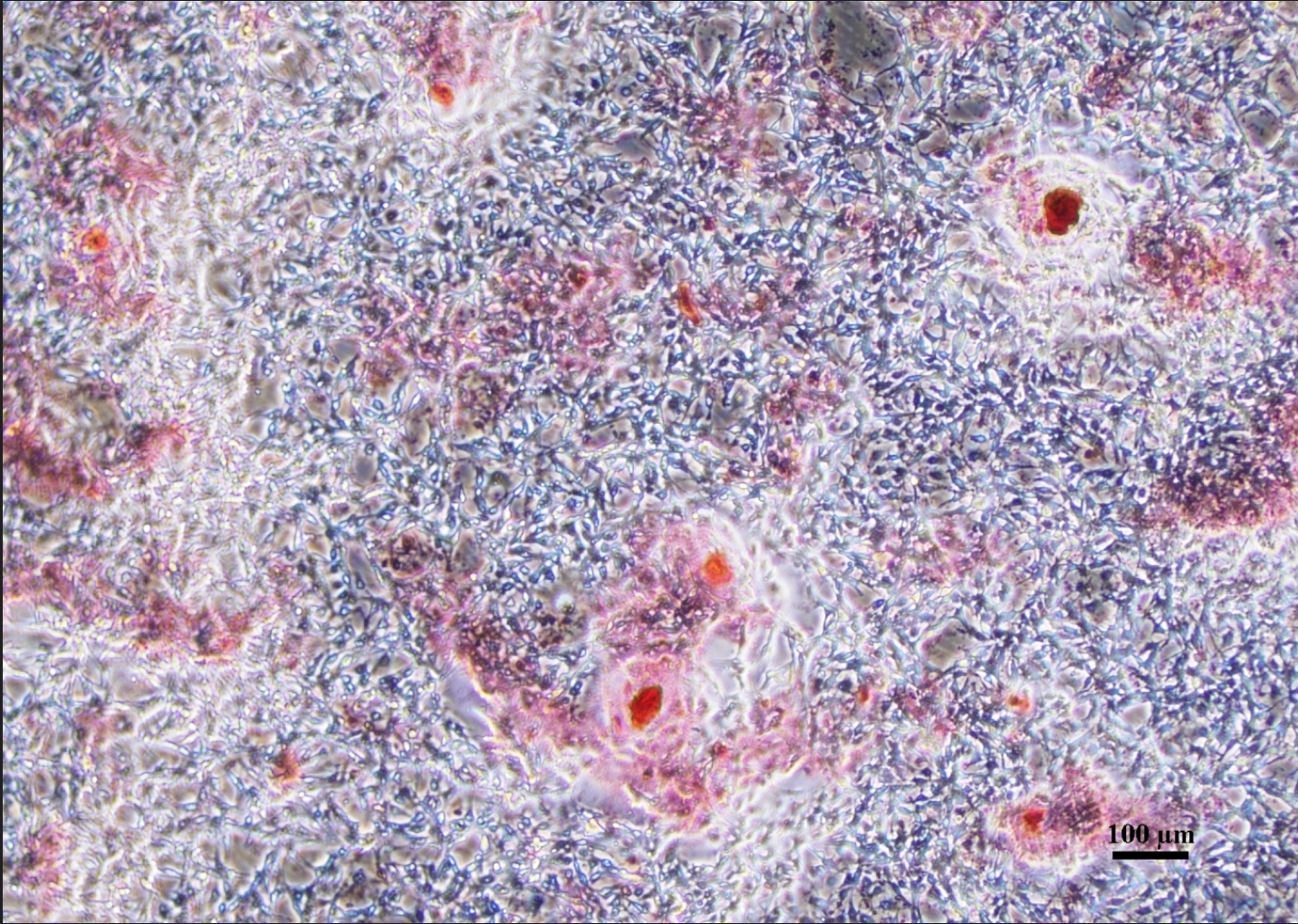

Supplement: Supplementary file 5 [file DataSheet4.zip › raw data_Figure8A C F/C/DAY21 LY294002.png]

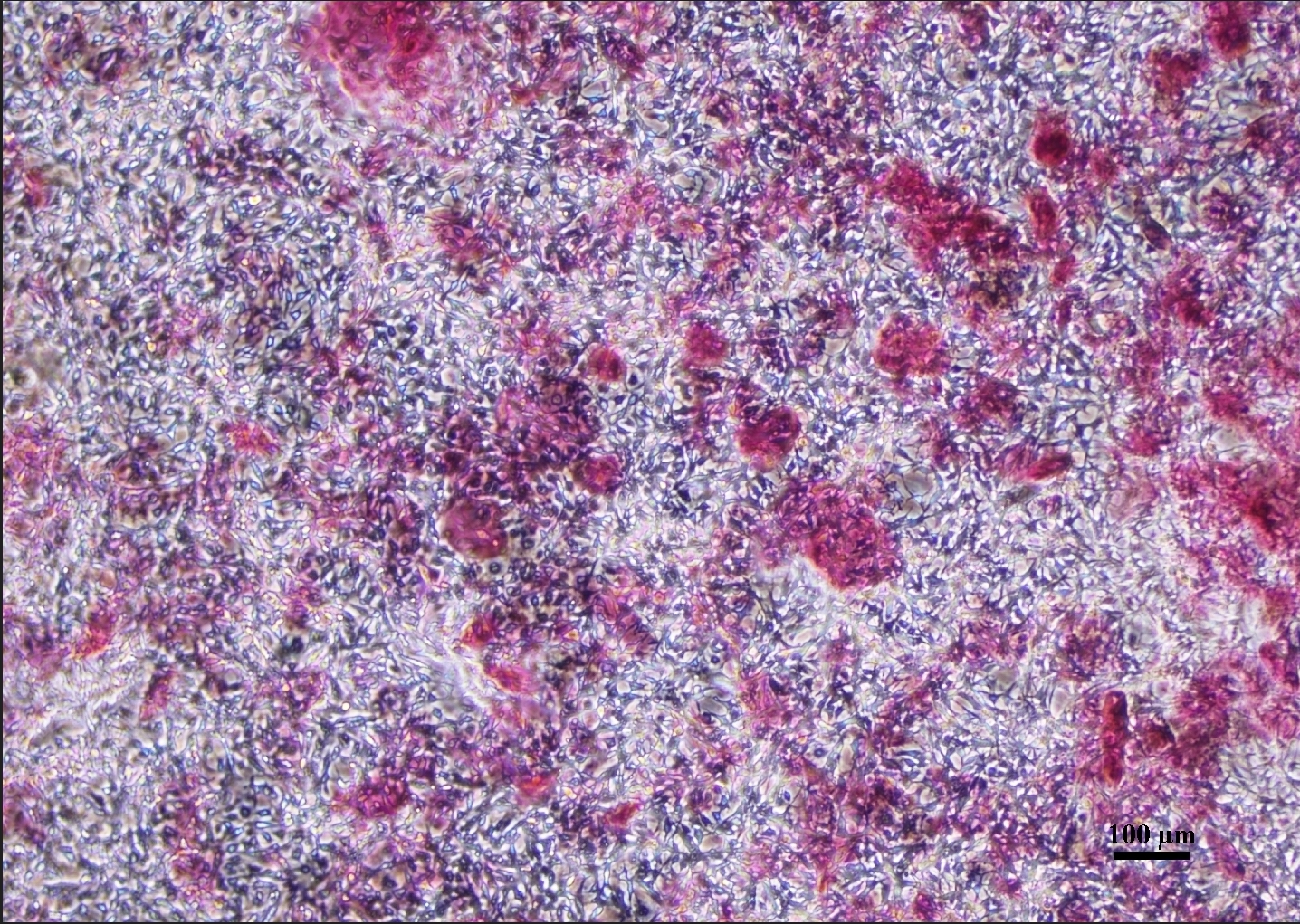

Supplement: Supplementary file 5 [file DataSheet4.zip › raw data_Figure8A C F/C/DAY21 MS.png]

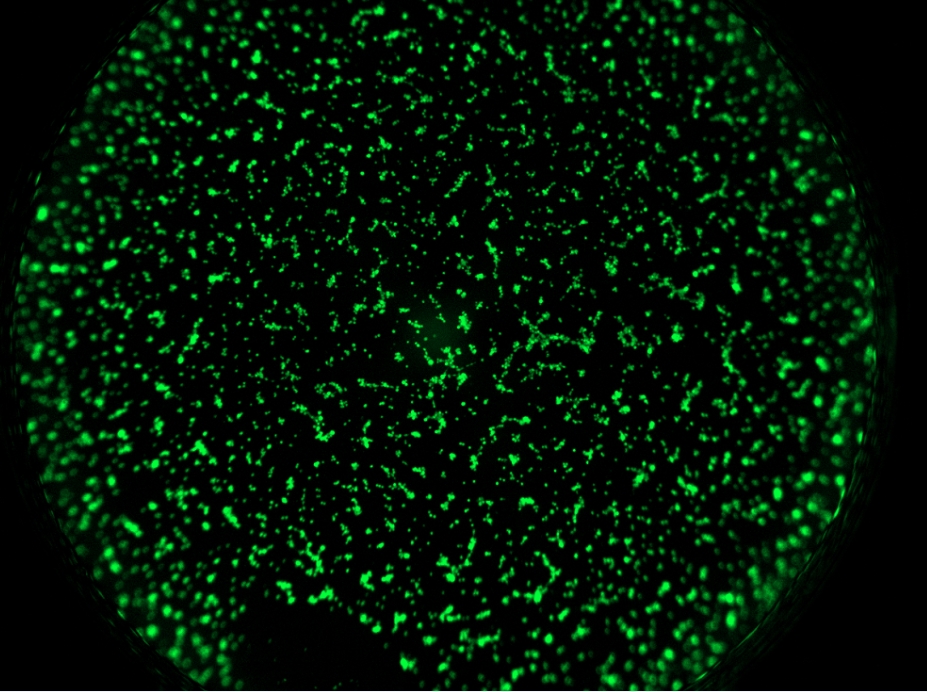

Supplement: Supplementary file 5 [file DataSheet4.zip › raw data_Figure8A C F/F/LY294002-1.png]

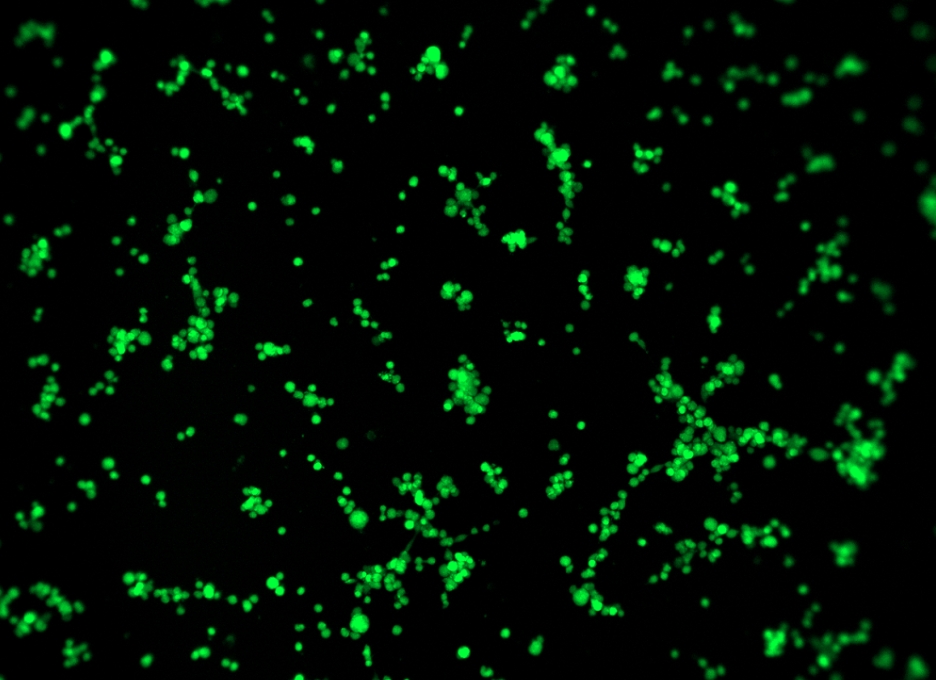

Supplement: Supplementary file 5 [file DataSheet4.zip › raw data_Figure8A C F/F/LY294002-2.png]

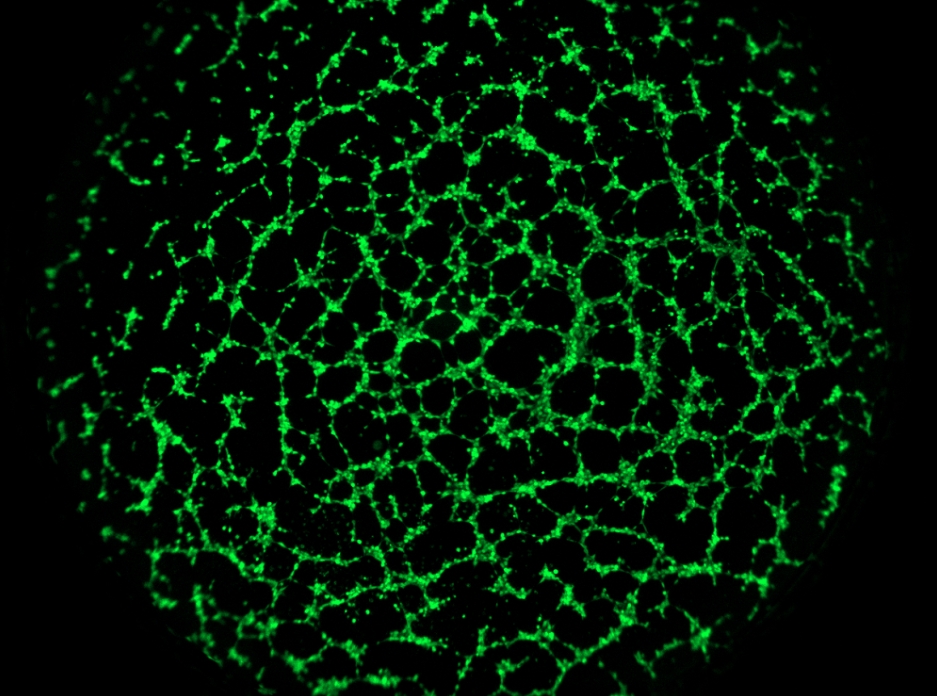

Supplement: Supplementary file 5 [file DataSheet4.zip › raw data_Figure8A C F/F/MS-1.png]

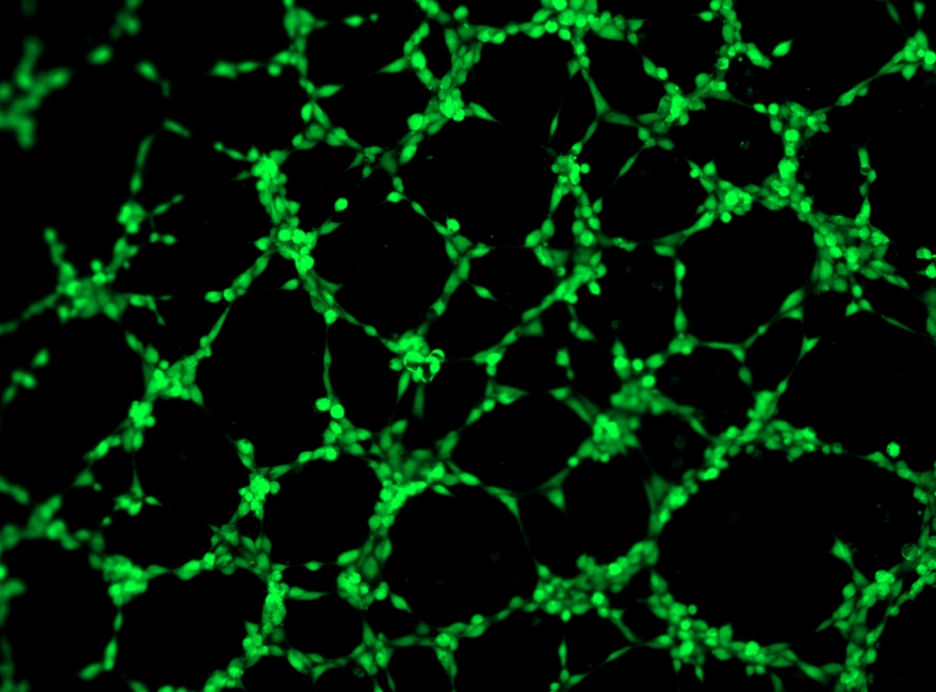

Supplement: Supplementary file 5 [file DataSheet4.zip › raw data_Figure8A C F/F/MS-2.png]

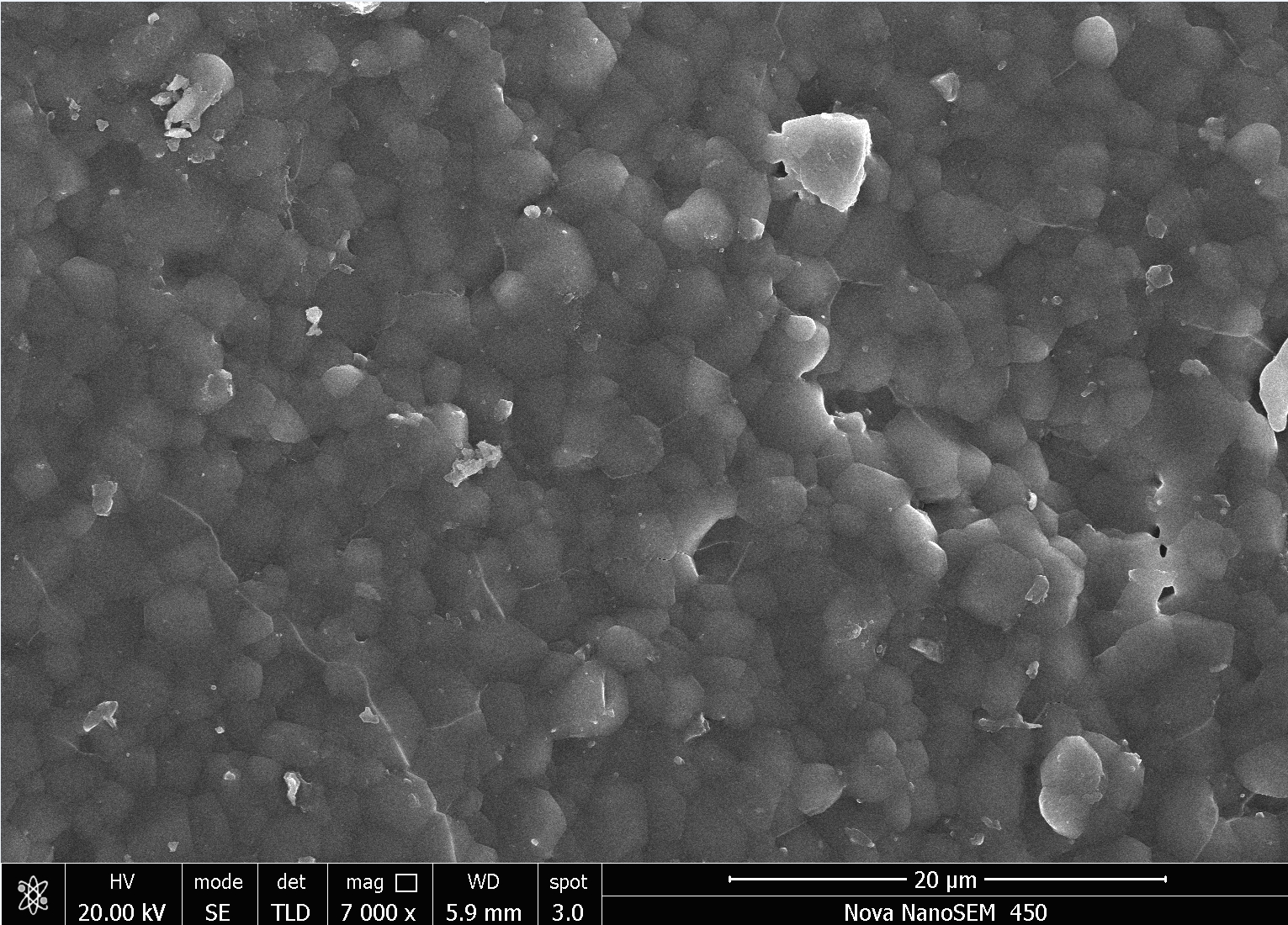

Supplement: Supplementary file 6 [file DataSheet1.zip › Figure 1 and 2/Figure 1A/0MS.png]

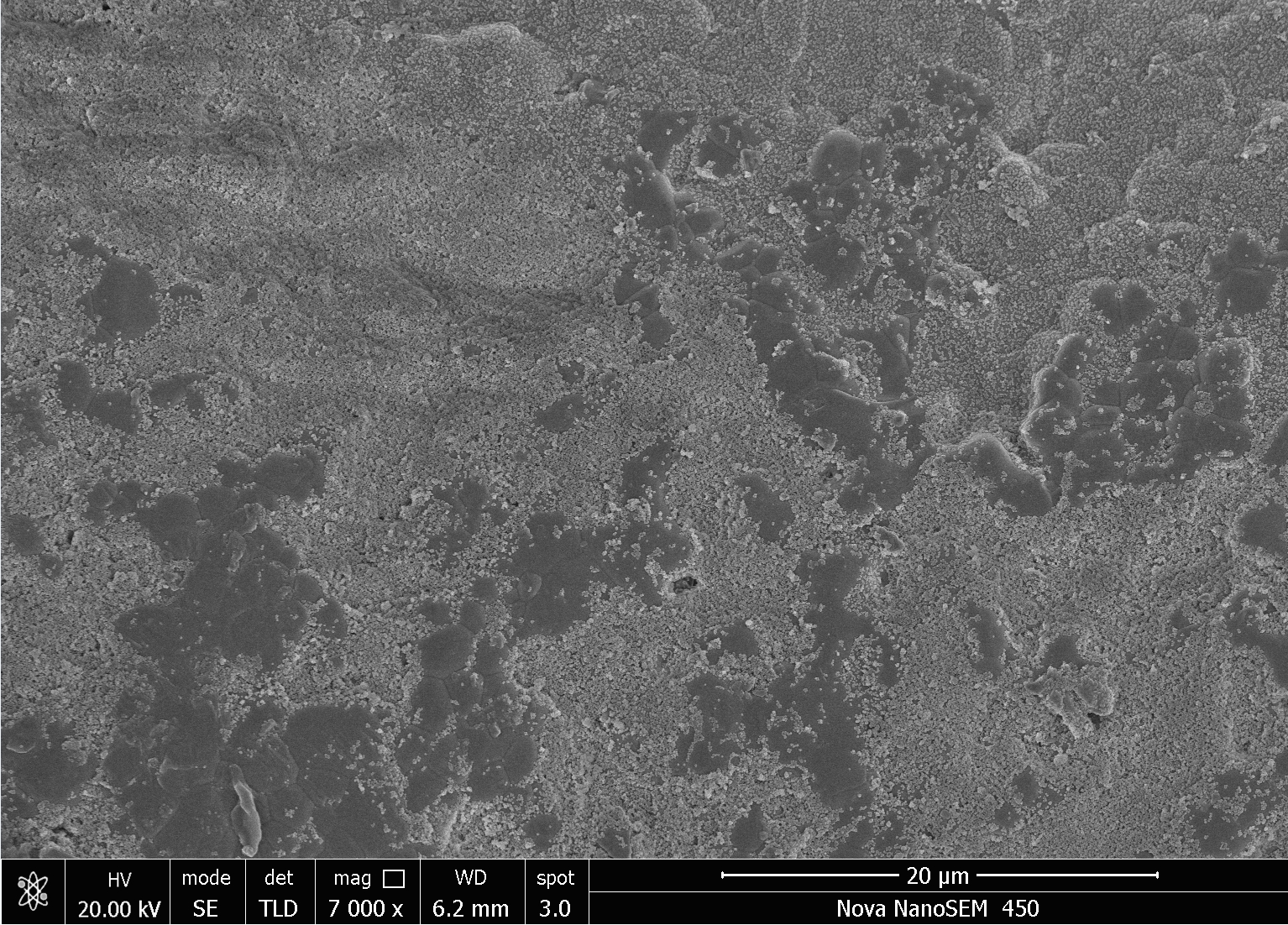

Supplement: Supplementary file 6 [file DataSheet1.zip › Figure 1 and 2/Figure 1A/10MS.png]

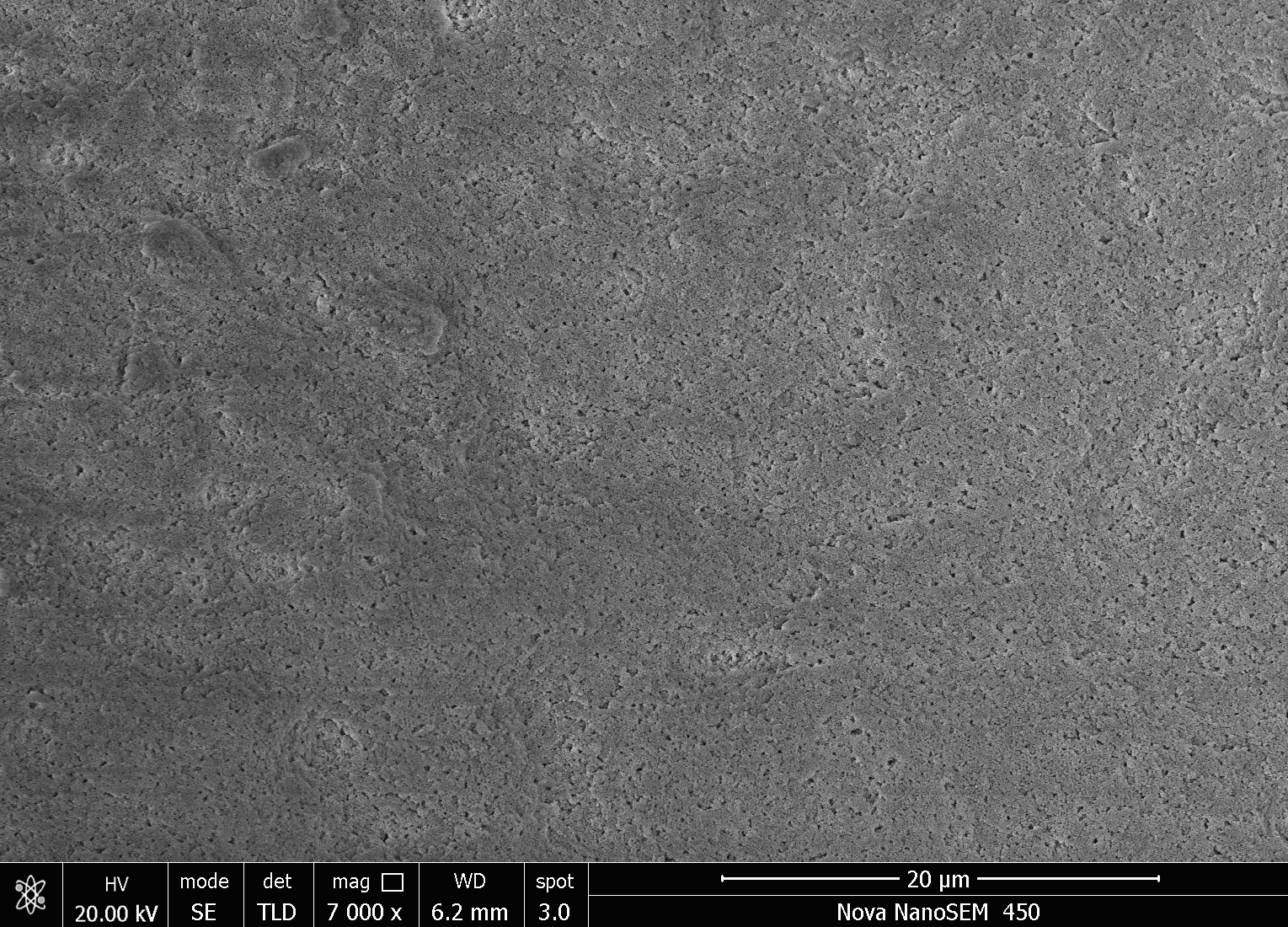

Supplement: Supplementary file 6 [file DataSheet1.zip › Figure 1 and 2/Figure 1A/15MS.png]

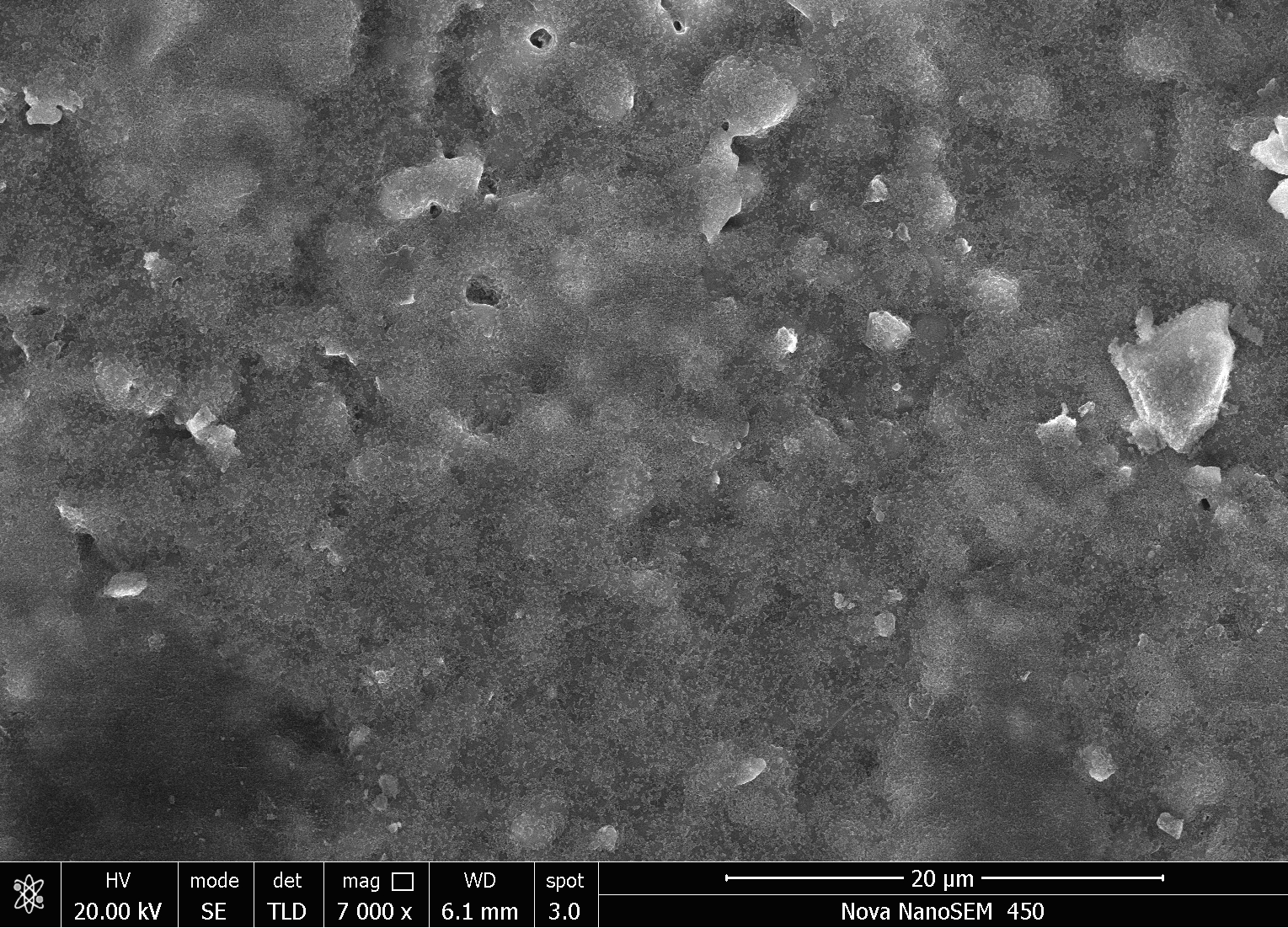

Supplement: Supplementary file 6 [file DataSheet1.zip › Figure 1 and 2/Figure 1A/5MS.png]

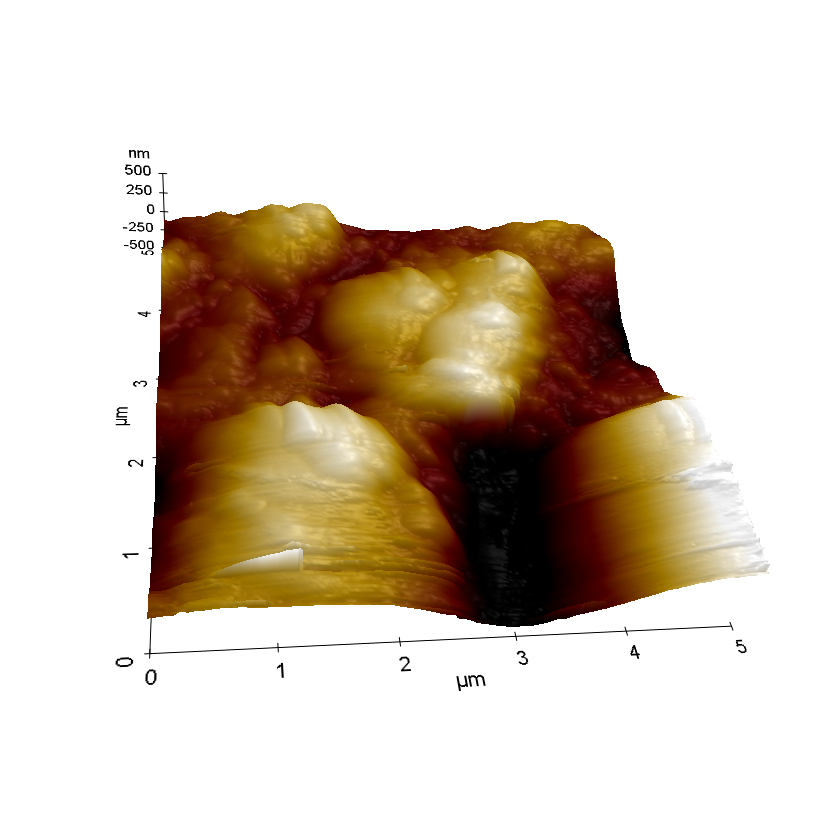

Supplement: Supplementary file 6 [file DataSheet1.zip › Figure 1 and 2/Figure 1B/0MS.png]

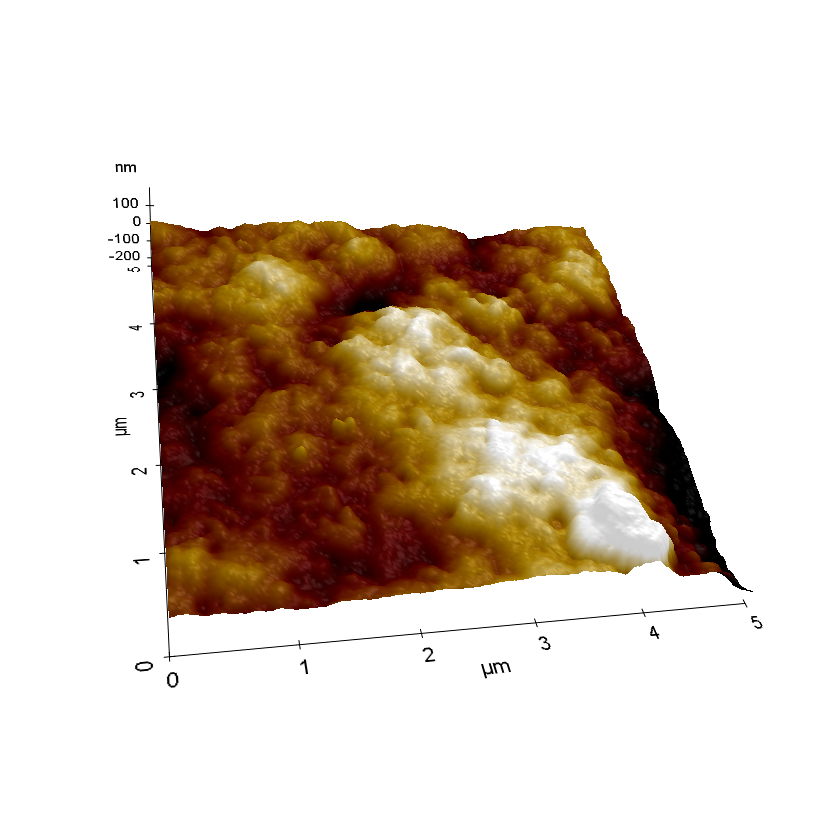

Supplement: Supplementary file 6 [file DataSheet1.zip › Figure 1 and 2/Figure 1B/10MS.png]

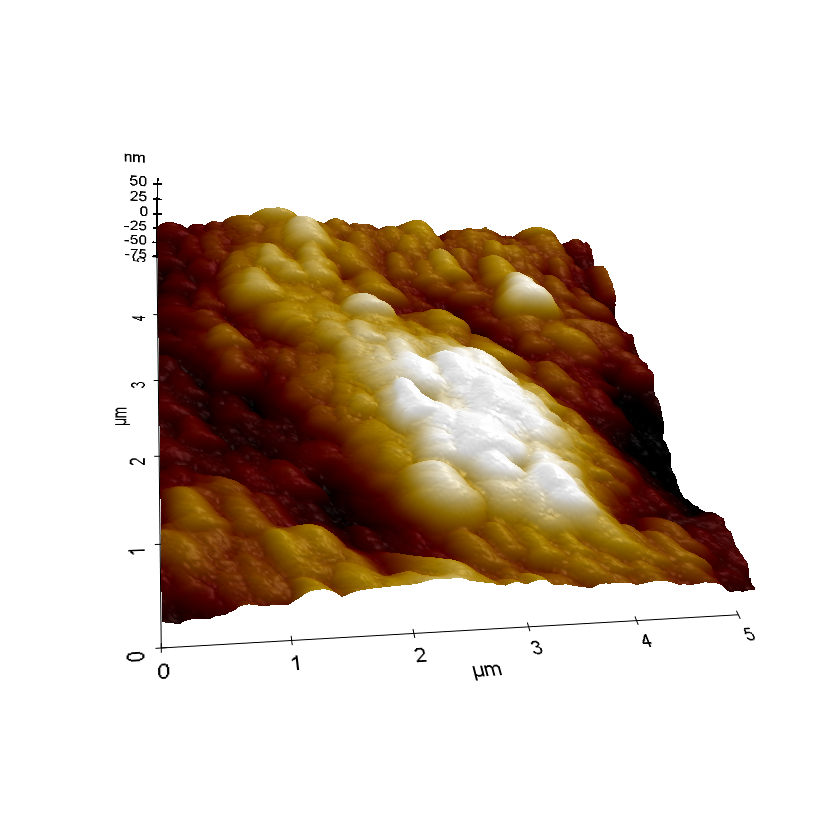

Supplement: Supplementary file 6 [file DataSheet1.zip › Figure 1 and 2/Figure 1B/15MS.png]

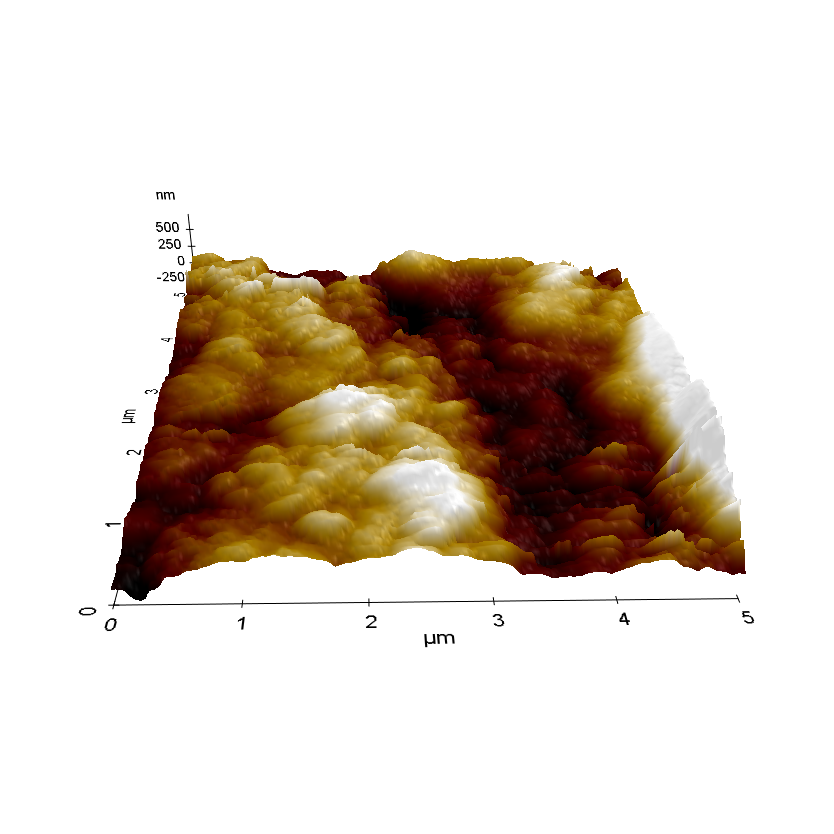

Supplement: Supplementary file 6 [file DataSheet1.zip › Figure 1 and 2/Figure 1B/5MS.png]

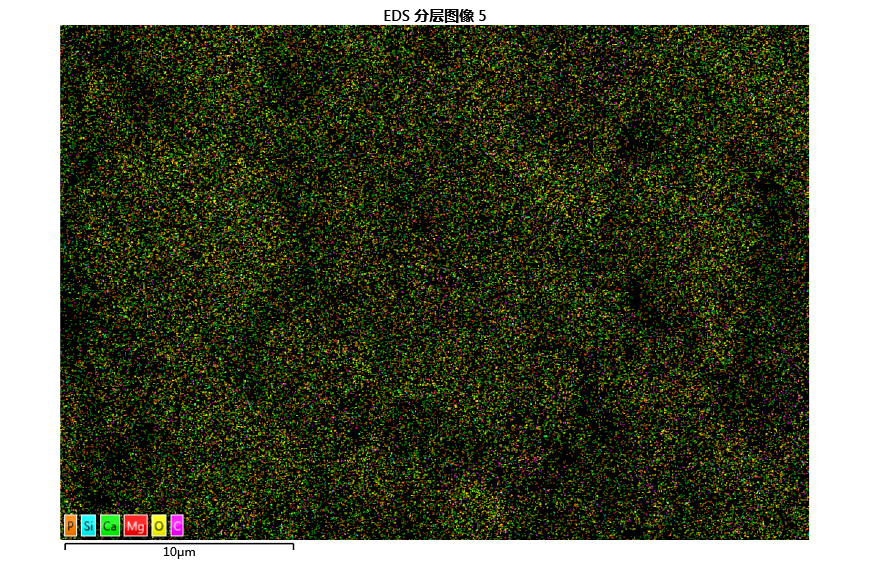

Supplement: Supplementary file 6 [file DataSheet1.zip › Figure 1 and 2/Figure 1C/0MS/0.docx]

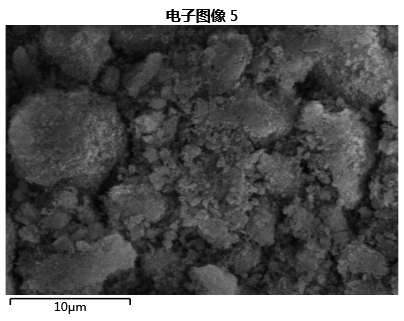

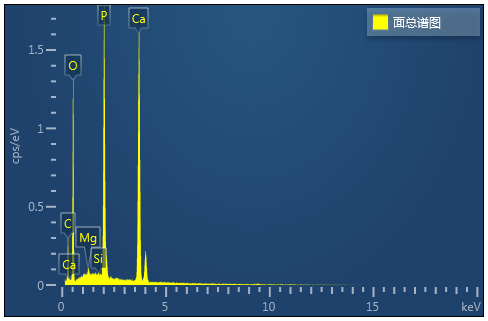


| 元素 | 线类型 | wt% | 原子百分比 |
| --- | --- | --- | --- |
| C | K线系 | 9.61 | 16.89 |
| O | K线系 | 41.54 | 54.79 |
| Mg | K线系 | 0.37 | 0.32 |
| Si | K线系 | 0.00 | 0.00 |
| P | K线系 | 15.97 | 10.88 |
| Ca | K线系 | 32.52 | 17.12 |
| 总量: |  | 100.00 | 100.00 |

Supplement: Supplementary file 6 [file DataSheet1.zip › Figure 1 and 2/Figure 1C/0MS/yuan su.docx]

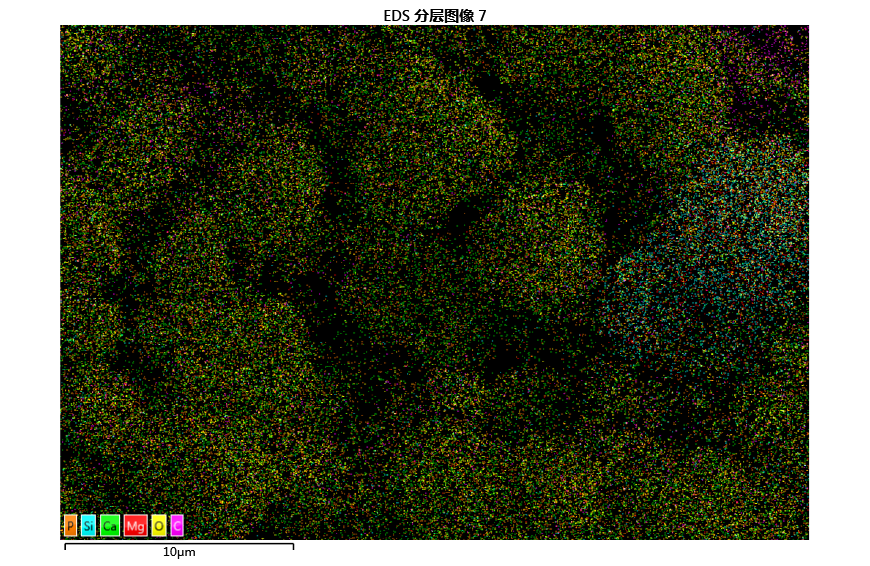

Supplement: Supplementary file 6 [file DataSheet1.zip › Figure 1 and 2/Figure 1C/10MS/10.docx]

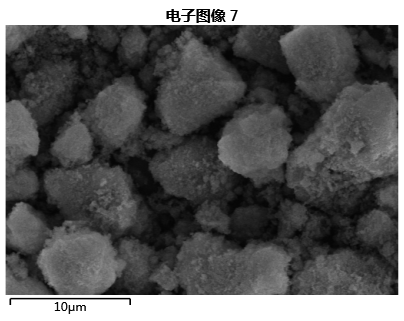

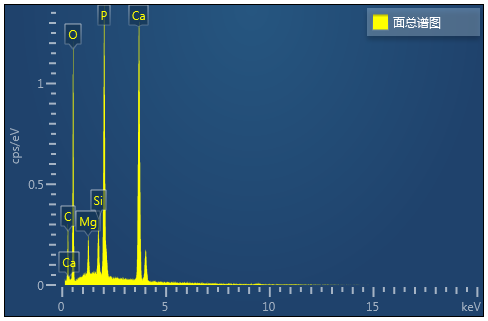


| 元素 | 线类型 | wt% | 原子百分比 |
| --- | --- | --- | --- |
| C | K线系 | 11.14 | 19.22 |
| O | K线系 | 40.45 | 52.41 |
| Mg | K线系 | 1.86 | 1.59 |
| Si | K线系 | 2.39 | 1.76 |
| P | K线系 | 14.33 | 9.59 |
| Ca | K线系 | 29.83 | 15.43 |
| 总量: |  | 100.00 | 100.00 |

Supplement: Supplementary file 6 [file DataSheet1.zip › Figure 1 and 2/Figure 1C/10MS/yuan su.docx]

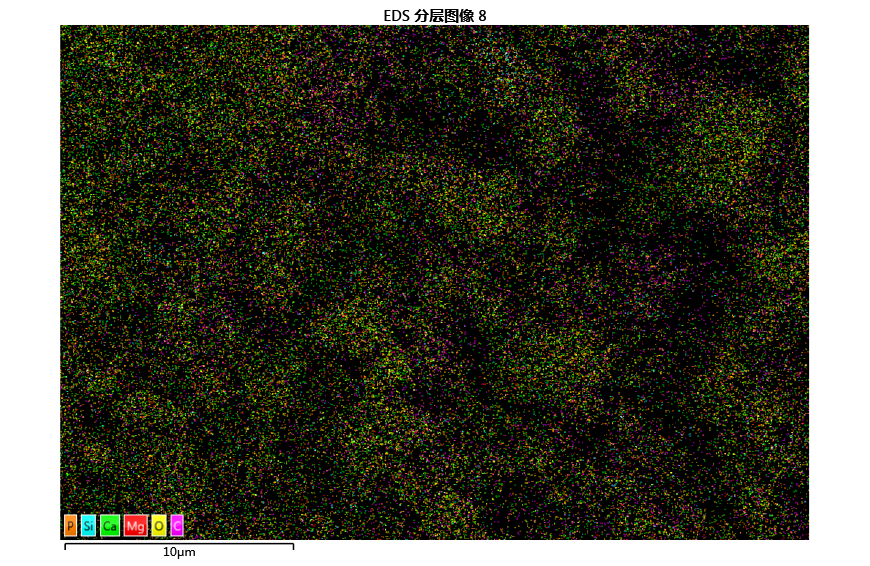

Supplement: Supplementary file 6 [file DataSheet1.zip › Figure 1 and 2/Figure 1C/15MS/15.docx]

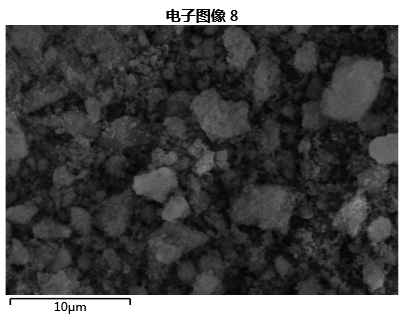

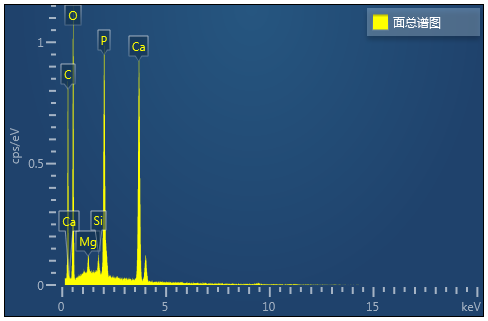


| 元素 | 线类型 | wt% | 原子百分比 |
| --- | --- | --- | --- |
| C | K线系 | 27.82 | 40.78 |
| O | K线系 | 39.13 | 43.06 |
| Mg | K线系 | 0.71 | 0.51 |
| Si | K线系 | 0.64 | 0.40 |
| P | K线系 | 10.15 | 5.77 |
| Ca | K线系 | 21.56 | 9.47 |
| 总量: |  | 100.00 | 100.00 |

Supplement: Supplementary file 6 [file DataSheet1.zip › Figure 1 and 2/Figure 1C/15MS/yuan su.docx]

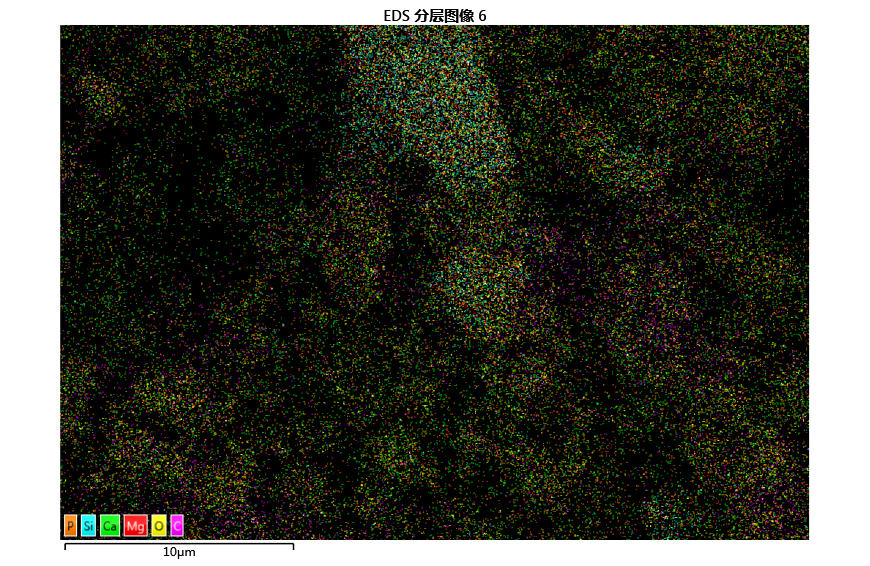

Supplement: Supplementary file 6 [file DataSheet1.zip › Figure 1 and 2/Figure 1C/5MS/5.docx]

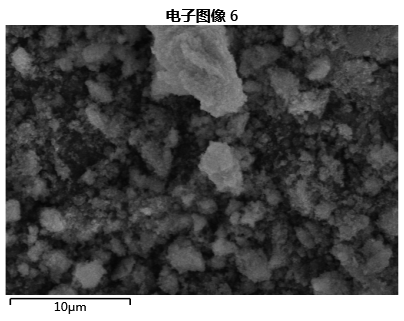

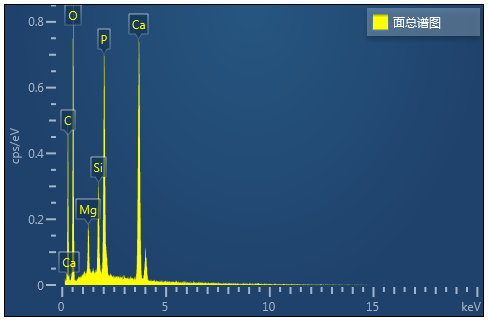


| 元素 | 线类型 | wt% | 原子百分比 |
| --- | --- | --- | --- |
| C | K线系 | 23.83 | 36.35 |
| O | K线系 | 38.18 | 43.73 |
| Mg | K线系 | 1.91 | 1.44 |
| Si | K线系 | 3.29 | 2.15 |
| P | K线系 | 9.91 | 5.87 |
| Ca | K线系 | 22.88 | 10.46 |
| 总量: |  | 100.00 | 100.00 |

Supplement: Supplementary file 6 [file DataSheet1.zip › Figure 1 and 2/Figure 1C/5MS/yuan su.docx]

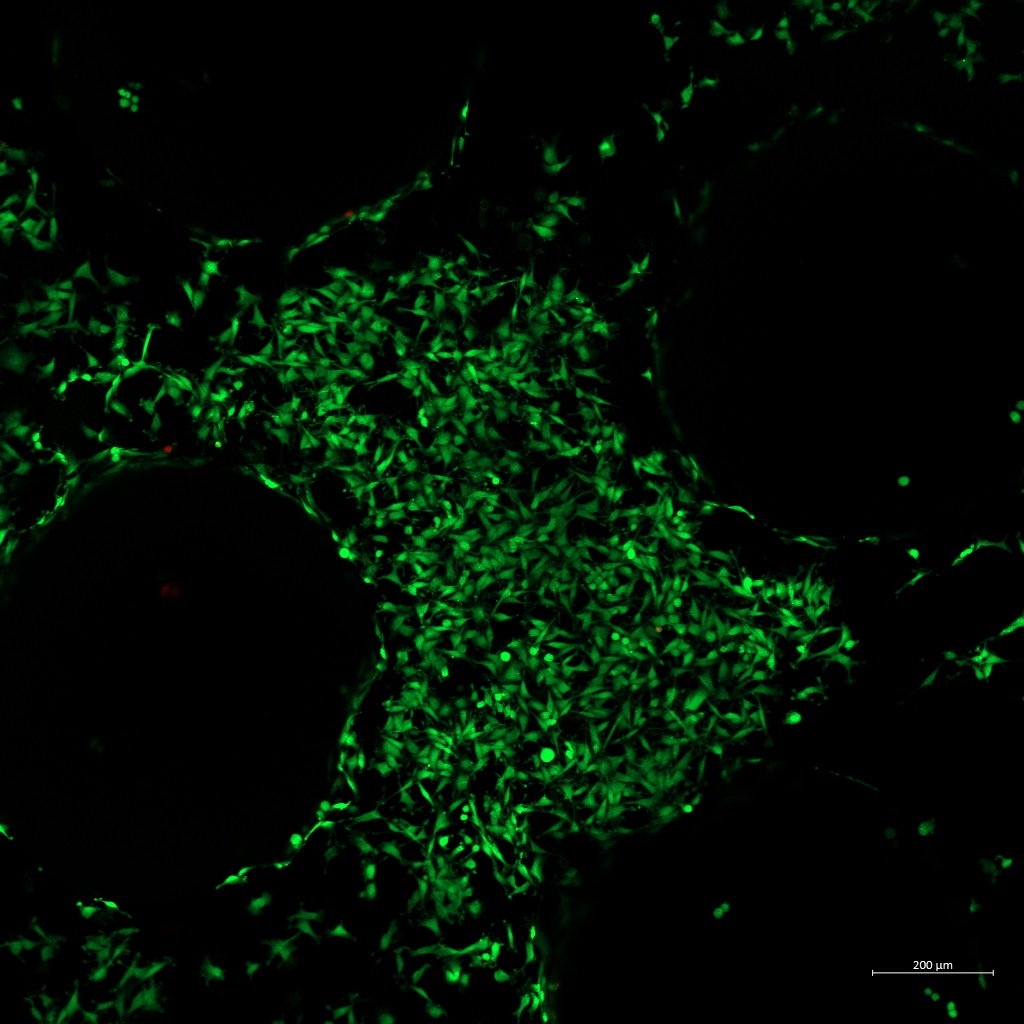

Supplement: Supplementary file 6 [file DataSheet1.zip › Figure 1 and 2/Figure 2/Figure 2A B F/Figure 2A/0MS.jpg]

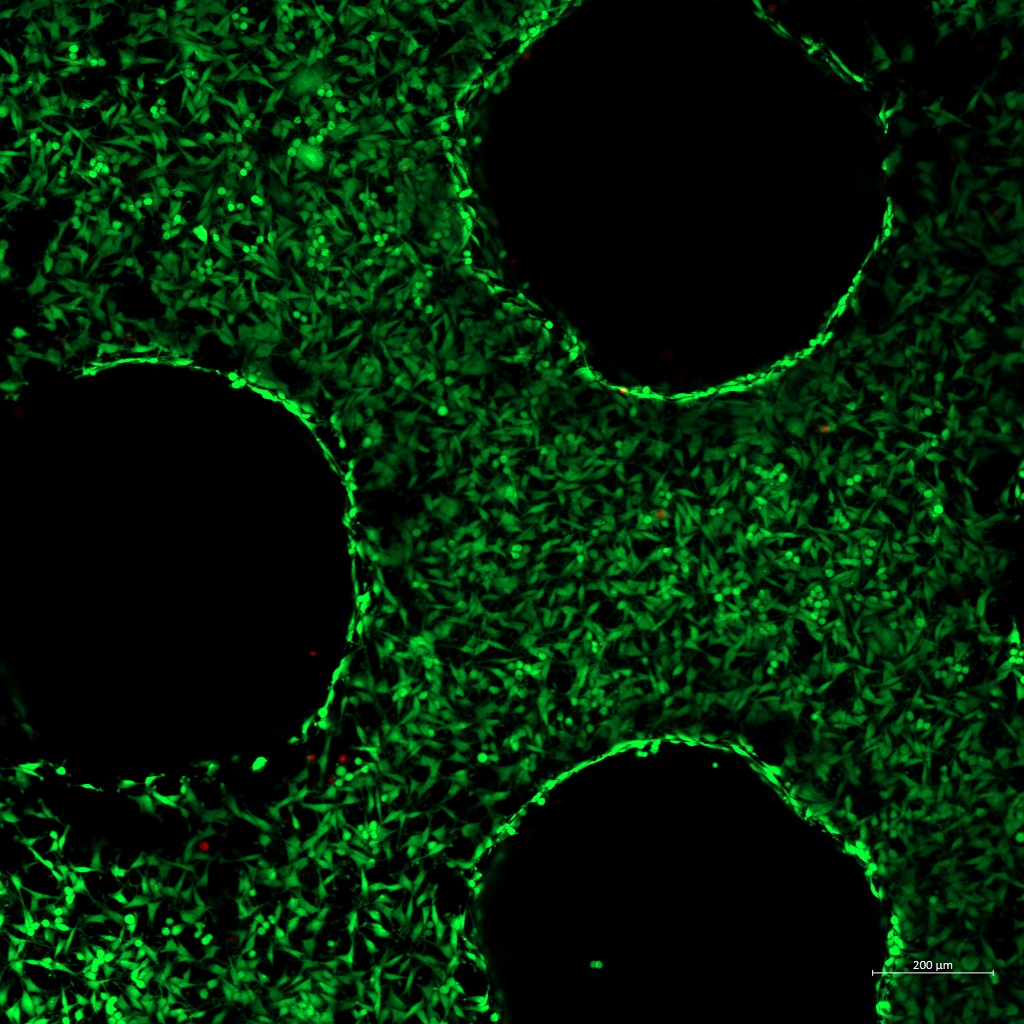

Supplement: Supplementary file 6 [file DataSheet1.zip › Figure 1 and 2/Figure 2/Figure 2A B F/Figure 2A/10MS.jpg]

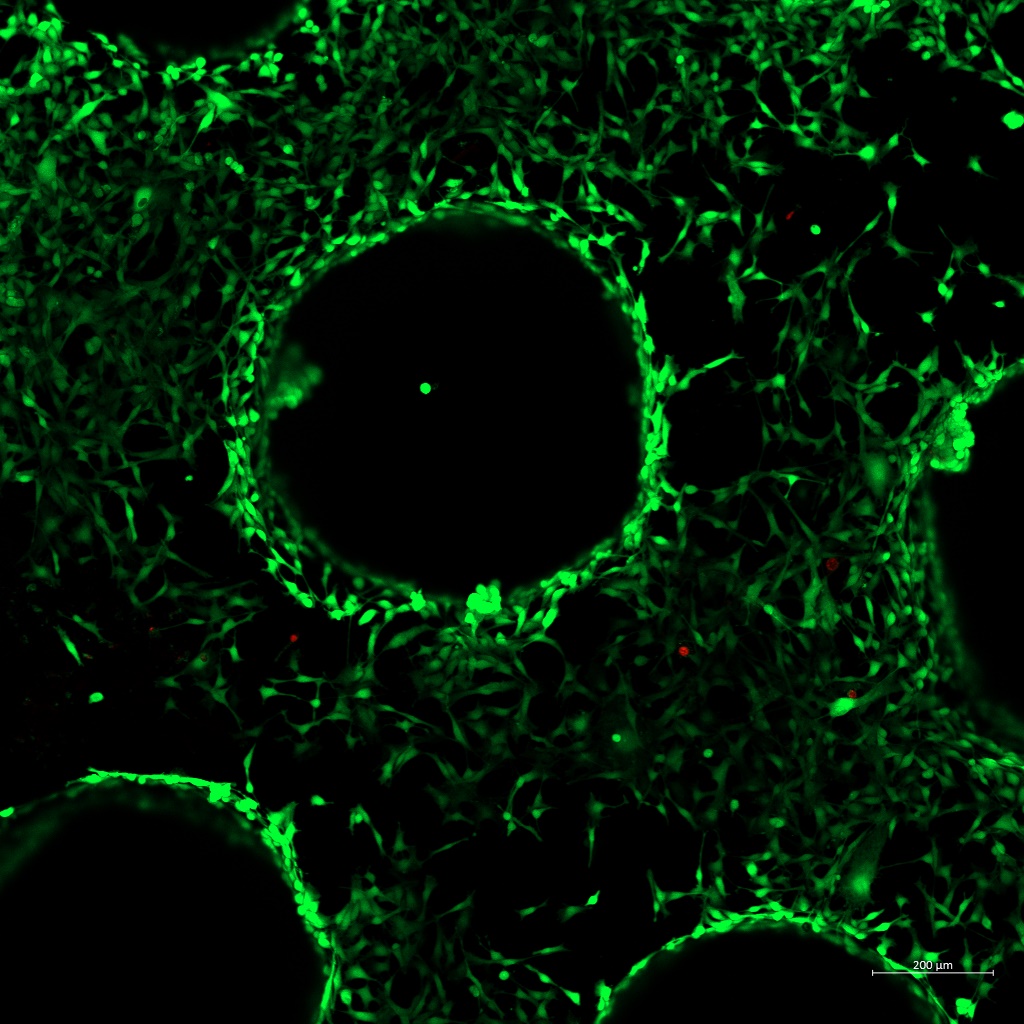

Supplement: Supplementary file 6 [file DataSheet1.zip › Figure 1 and 2/Figure 2/Figure 2A B F/Figure 2A/15MS.jpg]

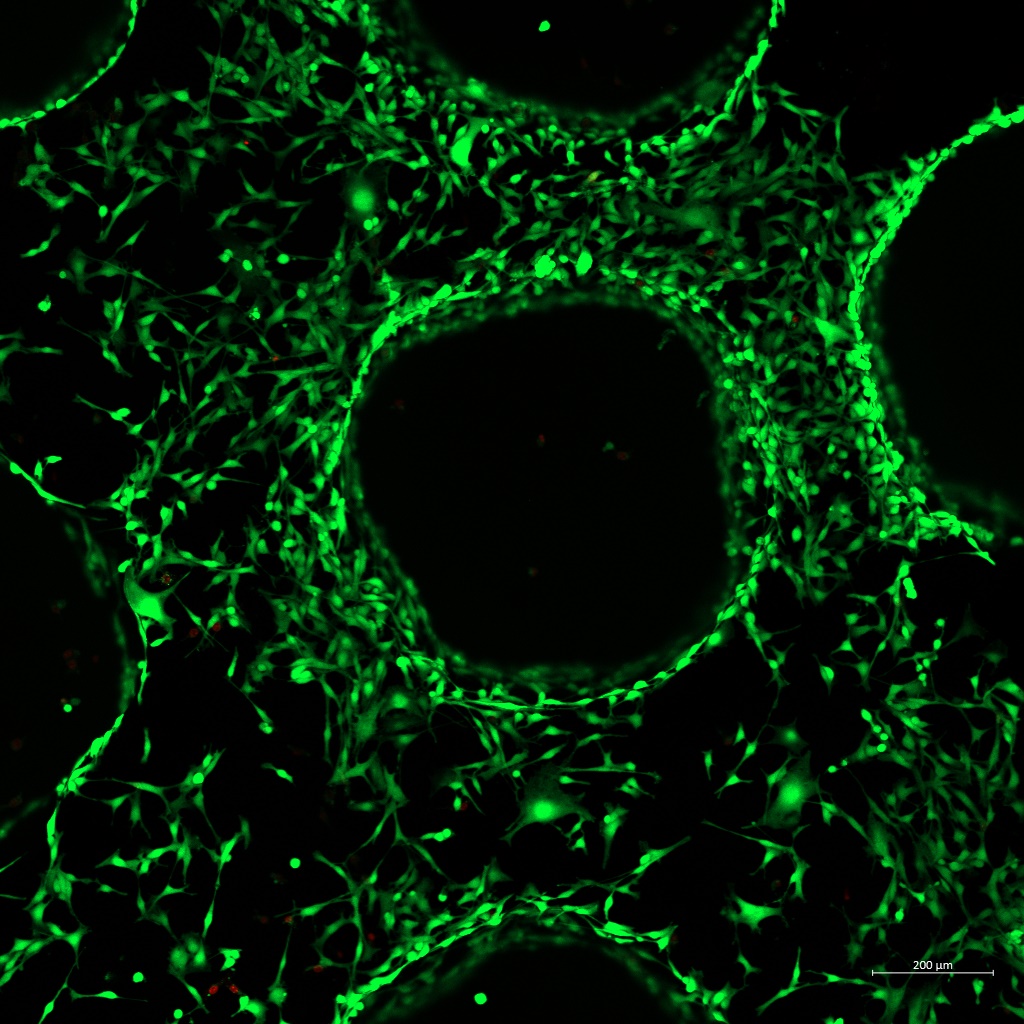

Supplement: Supplementary file 6 [file DataSheet1.zip › Figure 1 and 2/Figure 2/Figure 2A B F/Figure 2A/5MS.jpg]

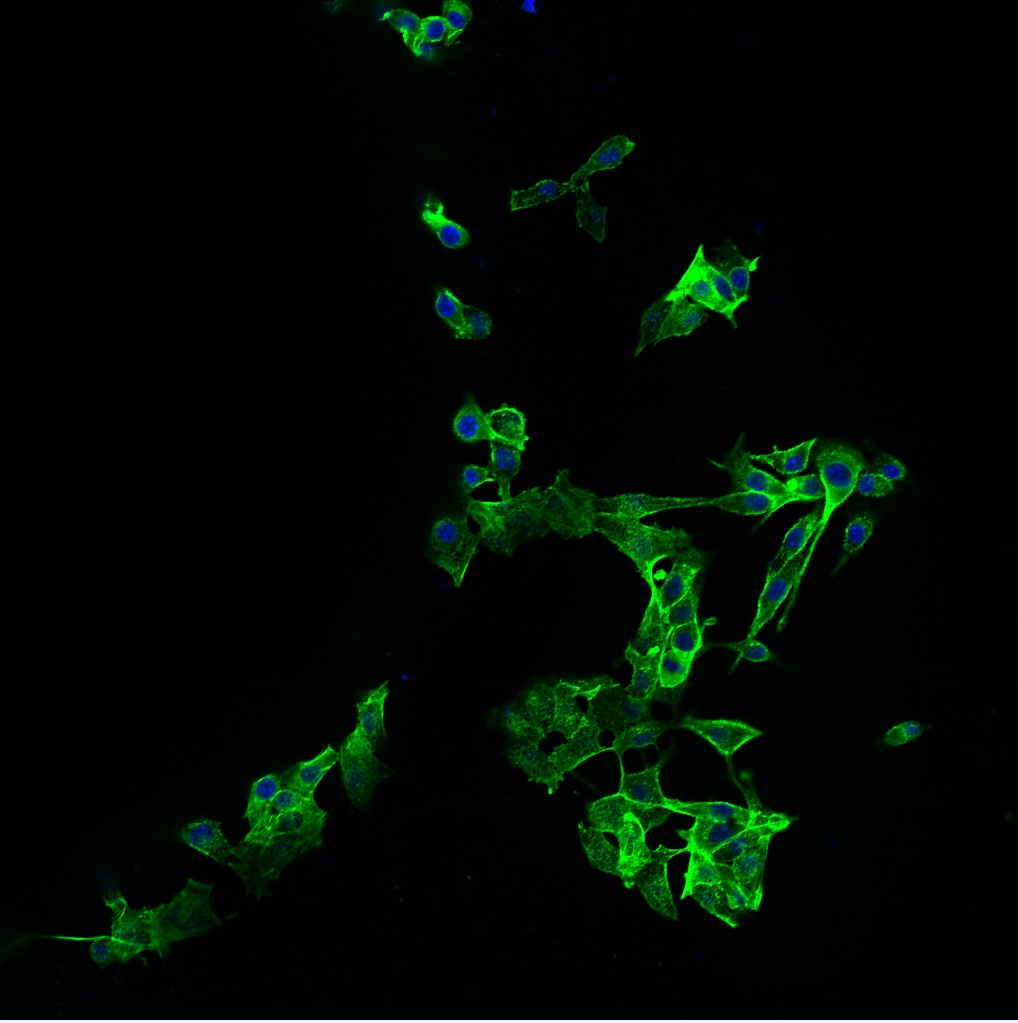

Supplement: Supplementary file 6 [file DataSheet1.zip › Figure 1 and 2/Figure 2/Figure 2A B F/Figure 2B/0MS.png]

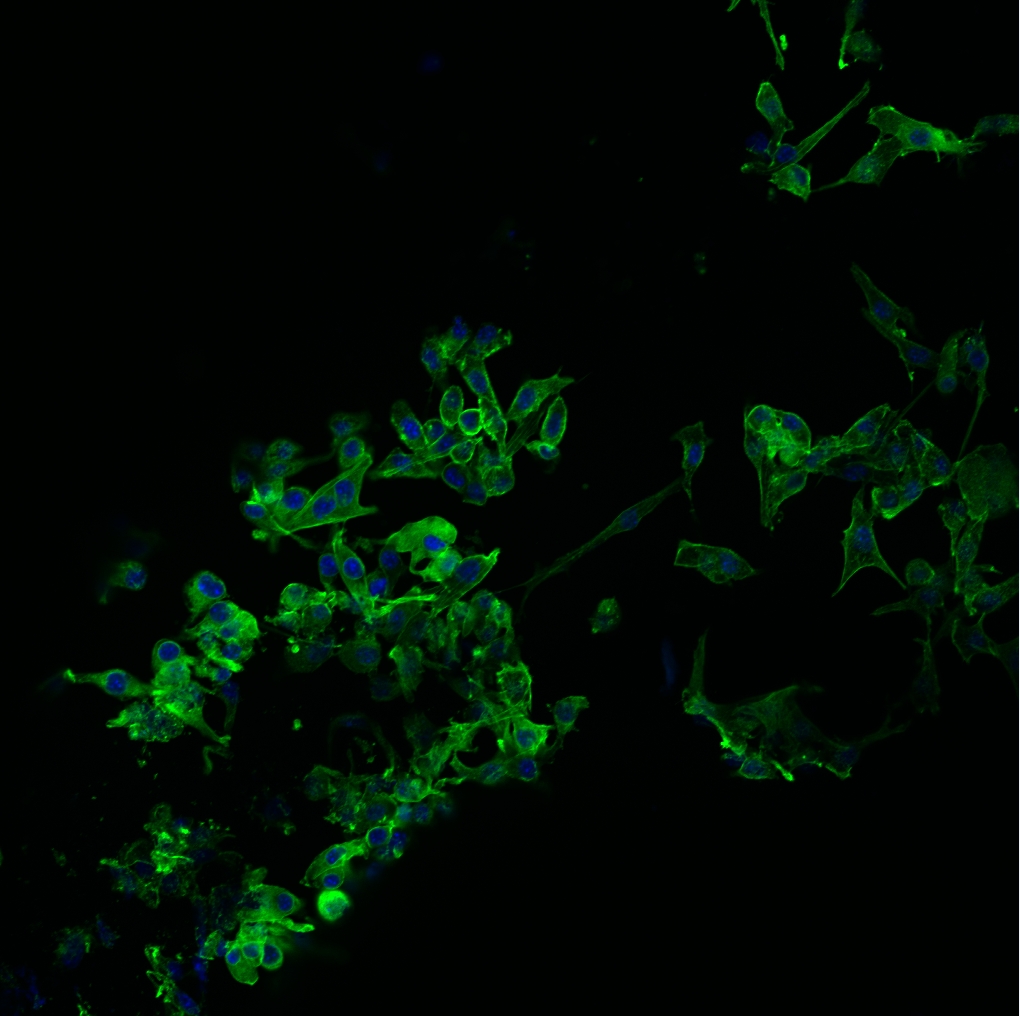

Supplement: Supplementary file 6 [file DataSheet1.zip › Figure 1 and 2/Figure 2/Figure 2A B F/Figure 2B/10MS.png]

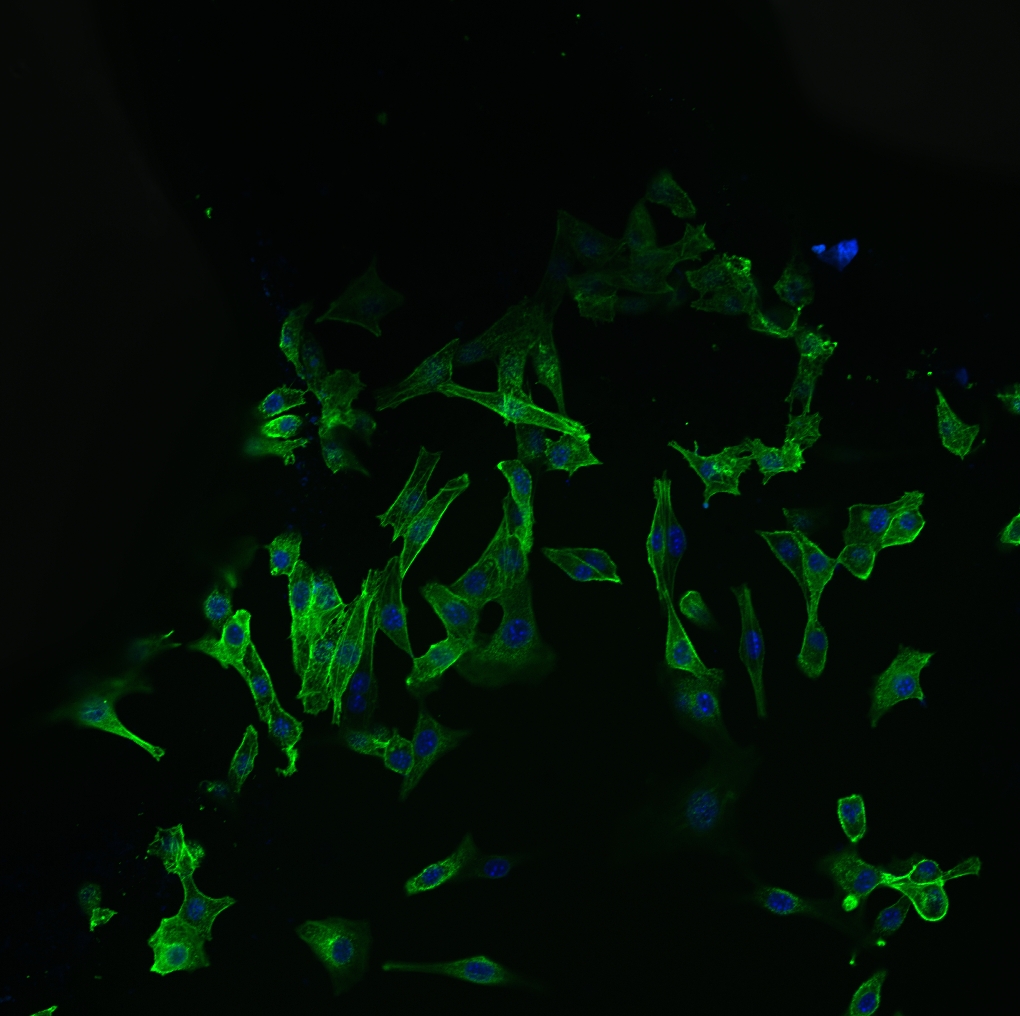

Supplement: Supplementary file 6 [file DataSheet1.zip › Figure 1 and 2/Figure 2/Figure 2A B F/Figure 2B/15MS.png]

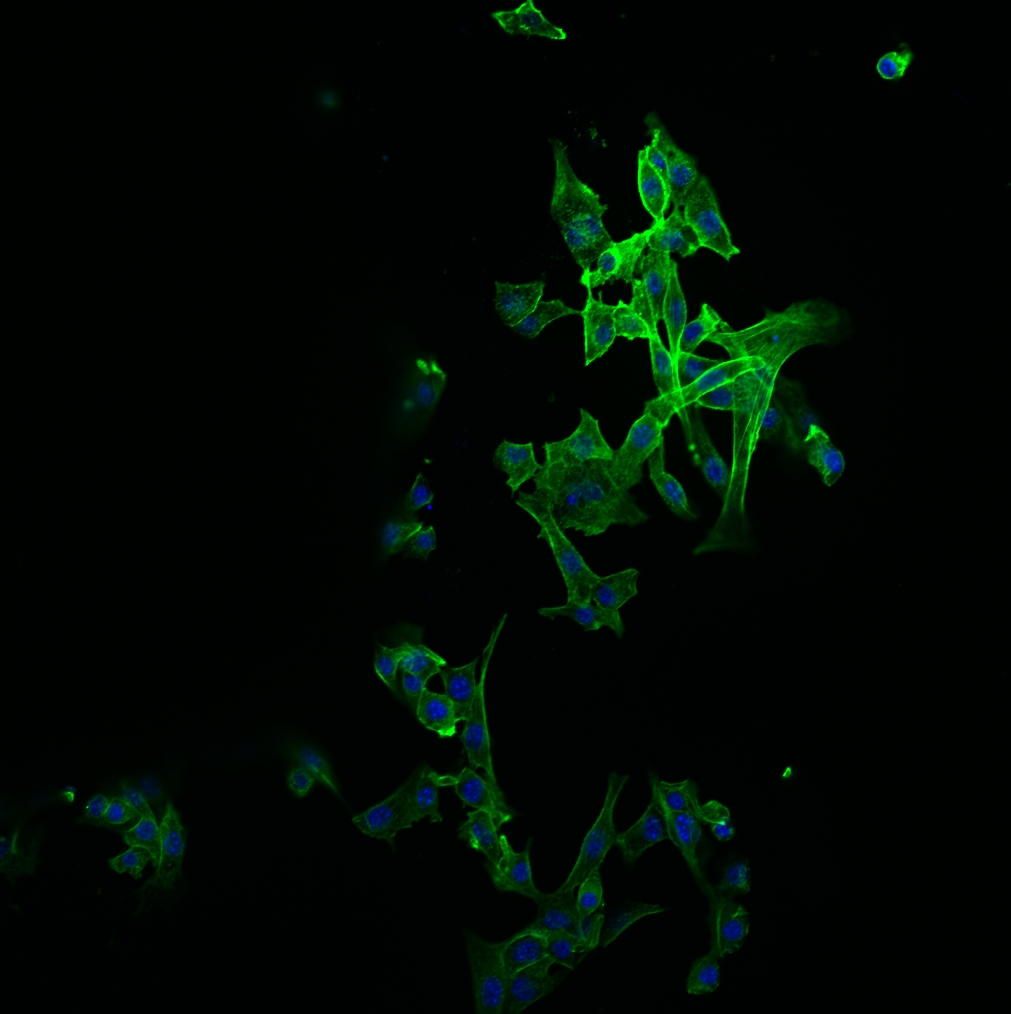

Supplement: Supplementary file 6 [file DataSheet1.zip › Figure 1 and 2/Figure 2/Figure 2A B F/Figure 2B/5MS.png]

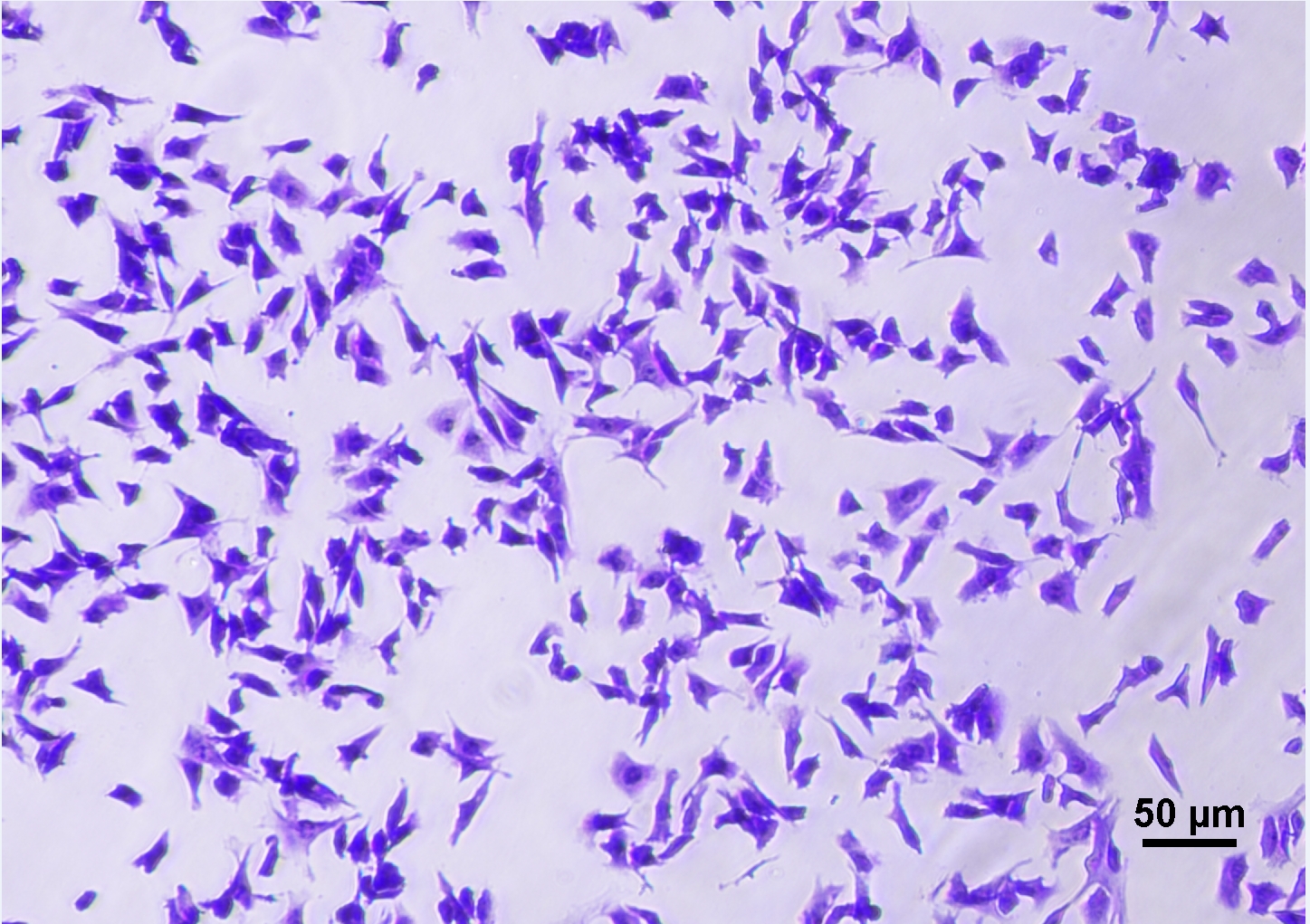

Supplement: Supplementary file 6 [file DataSheet1.zip › Figure 1 and 2/Figure 2/Figure 2A B F/Figure 2F/0MS.png]

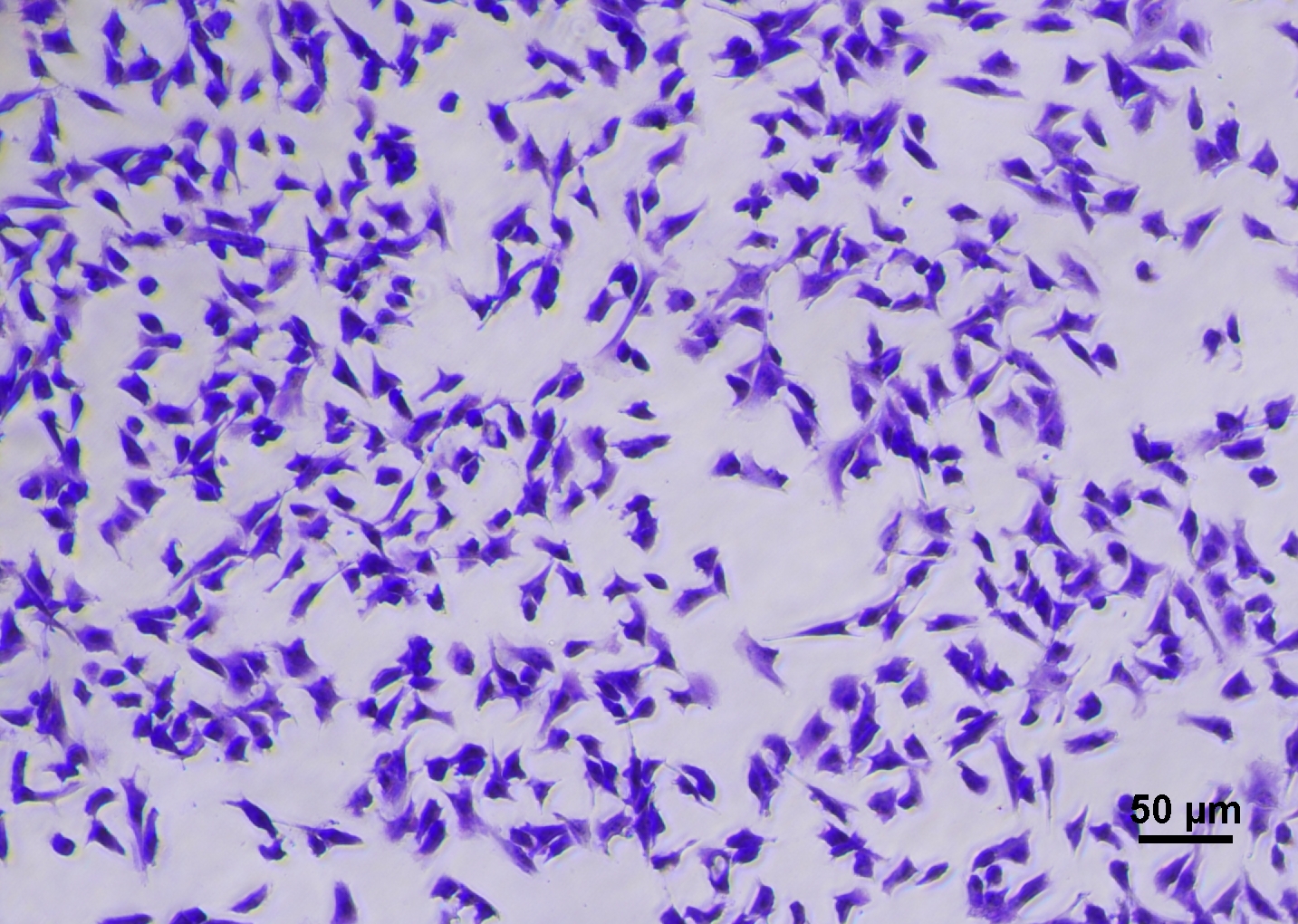

Supplement: Supplementary file 6 [file DataSheet1.zip › Figure 1 and 2/Figure 2/Figure 2A B F/Figure 2F/10MS.png]

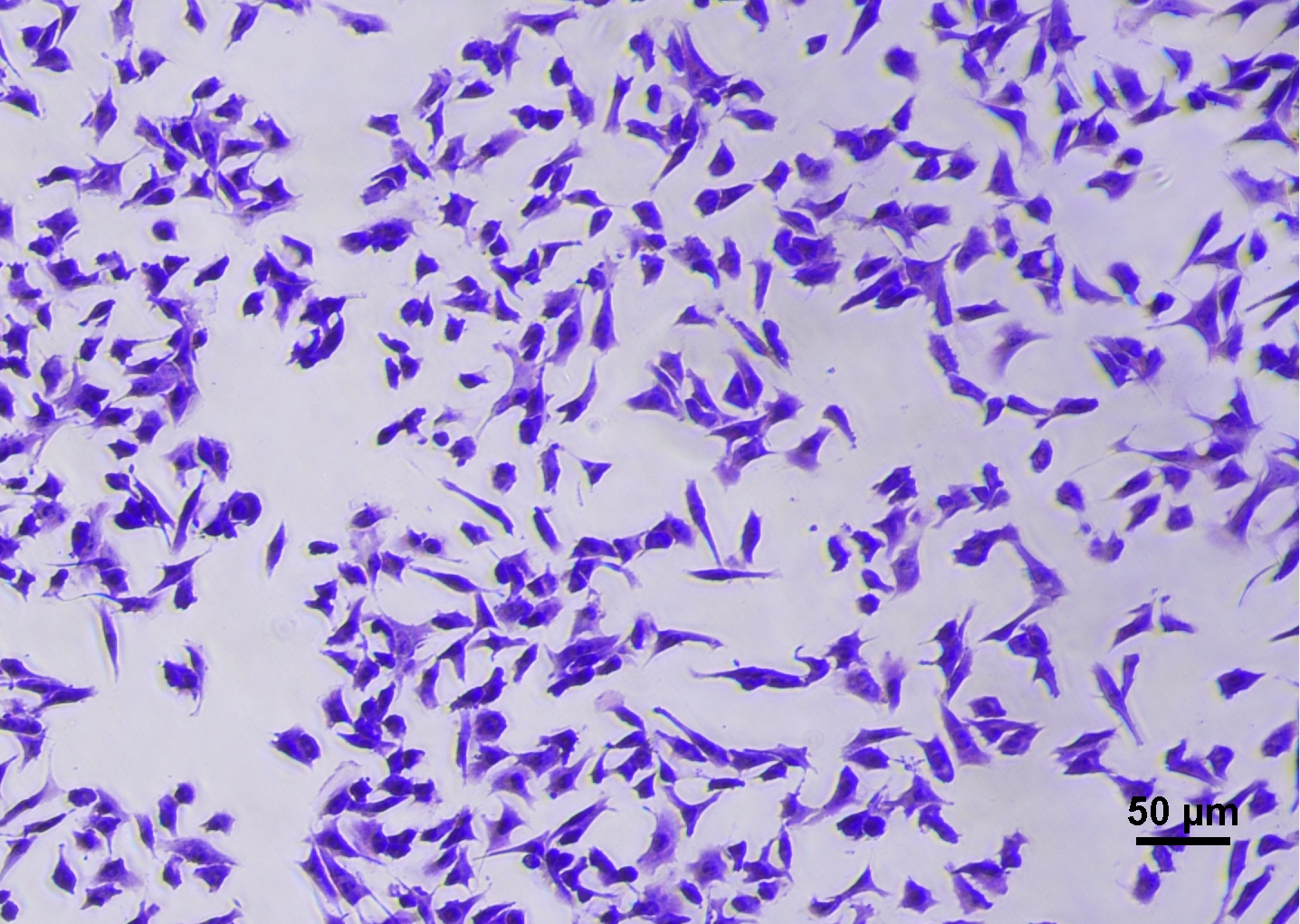

Supplement: Supplementary file 6 [file DataSheet1.zip › Figure 1 and 2/Figure 2/Figure 2A B F/Figure 2F/15MS.png]

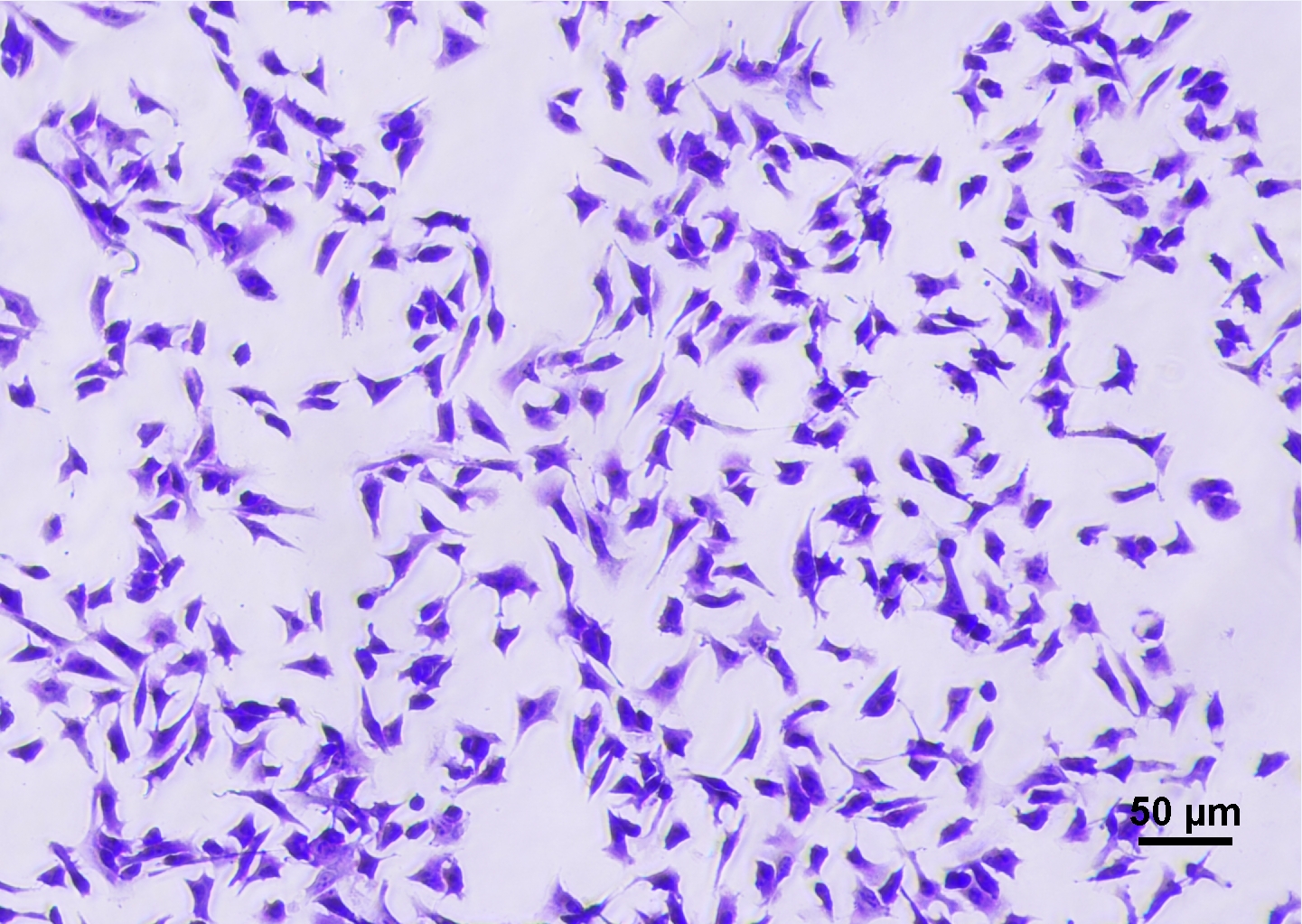

Supplement: Supplementary file 6 [file DataSheet1.zip › Figure 1 and 2/Figure 2/Figure 2A B F/Figure 2F/5MS.png]

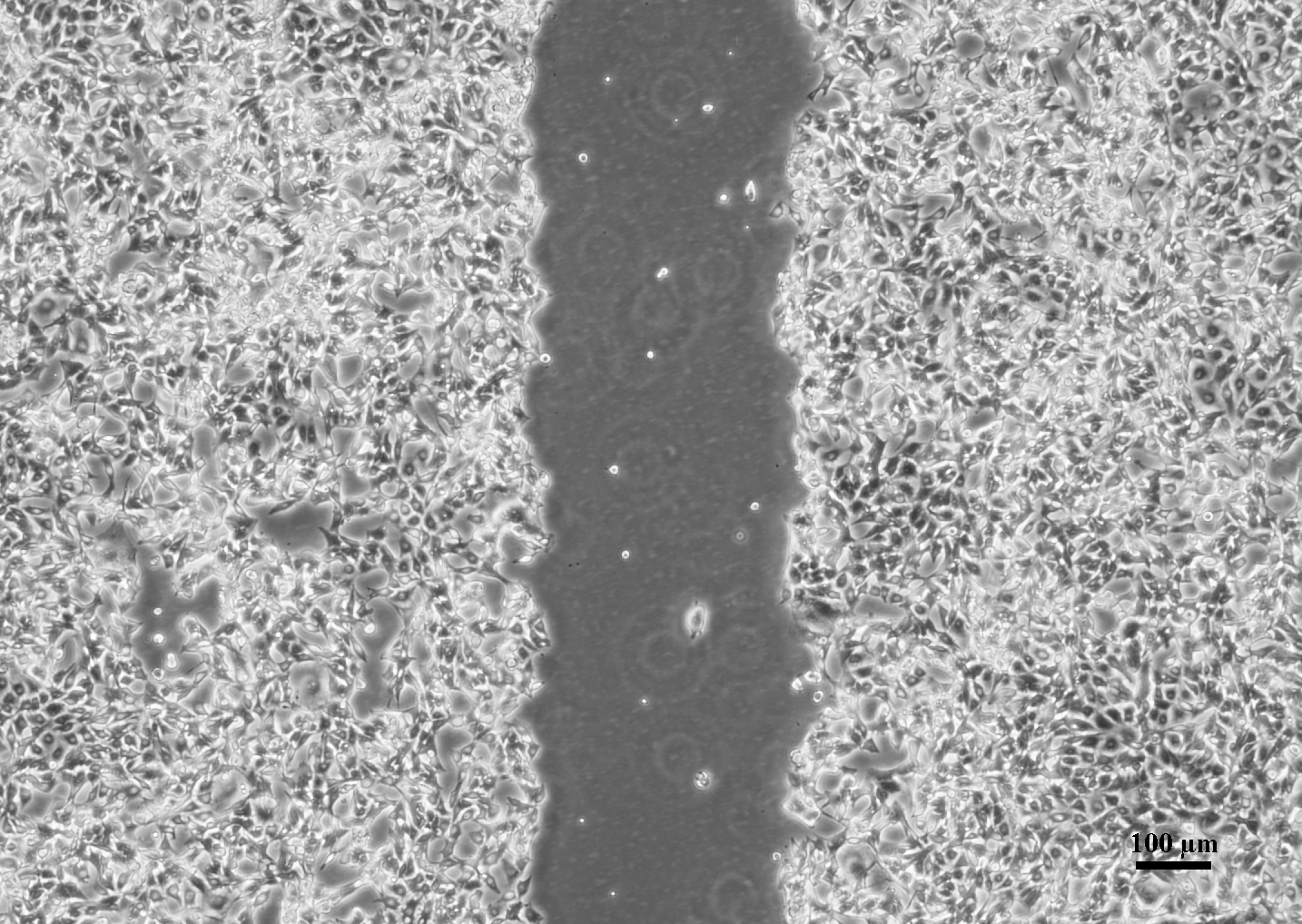

Supplement: Supplementary file 6 [file DataSheet1.zip › Figure 1 and 2/Figure 2/Figure 2C D E G/Figure 2D/0MS 0H.png]

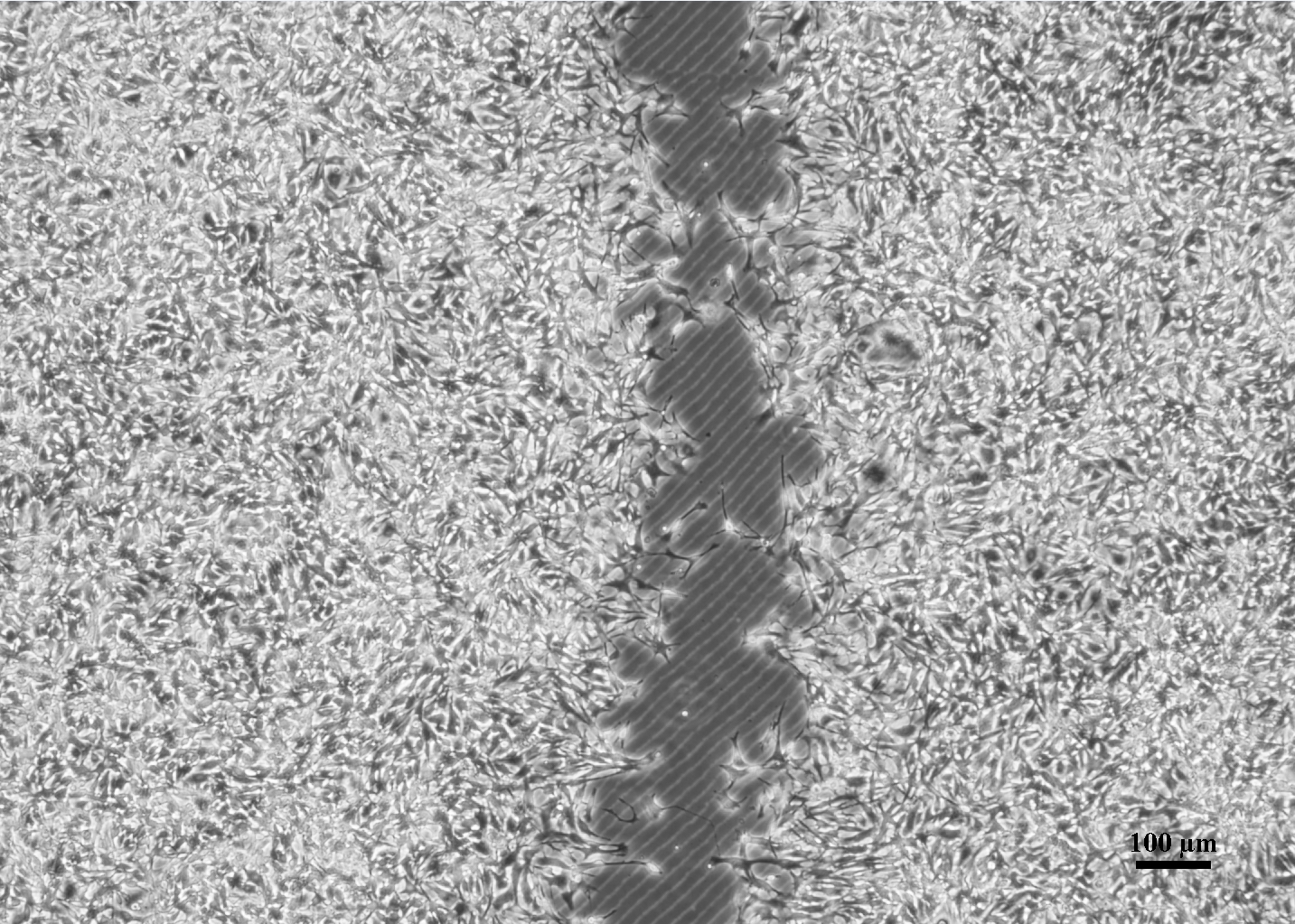

Supplement: Supplementary file 6 [file DataSheet1.zip › Figure 1 and 2/Figure 2/Figure 2C D E G/Figure 2D/0MS 24H.png]

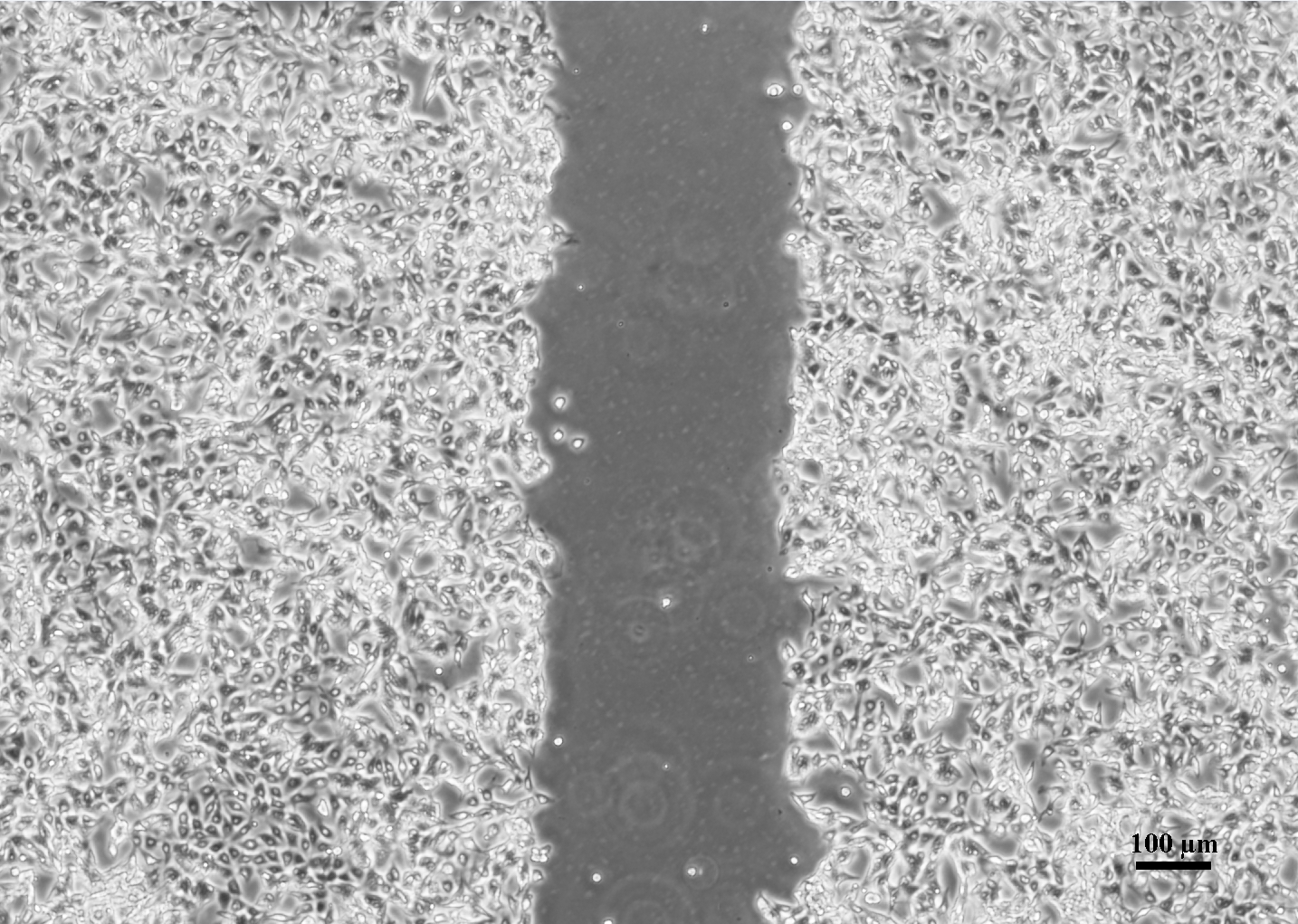

Supplement: Supplementary file 6 [file DataSheet1.zip › Figure 1 and 2/Figure 2/Figure 2C D E G/Figure 2D/10MS 0H.png]

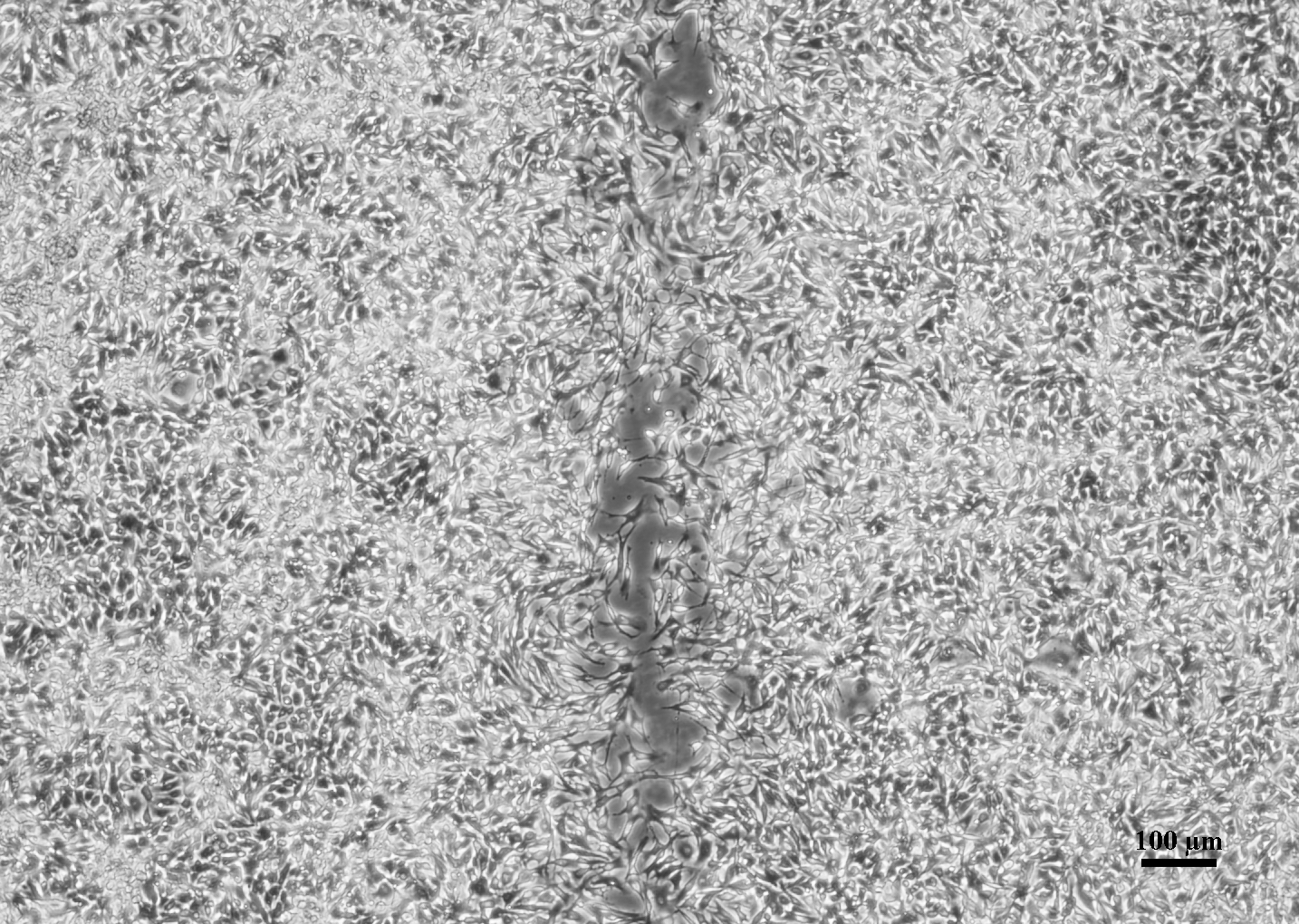

Supplement: Supplementary file 6 [file DataSheet1.zip › Figure 1 and 2/Figure 2/Figure 2C D E G/Figure 2D/10MS 24H.png]

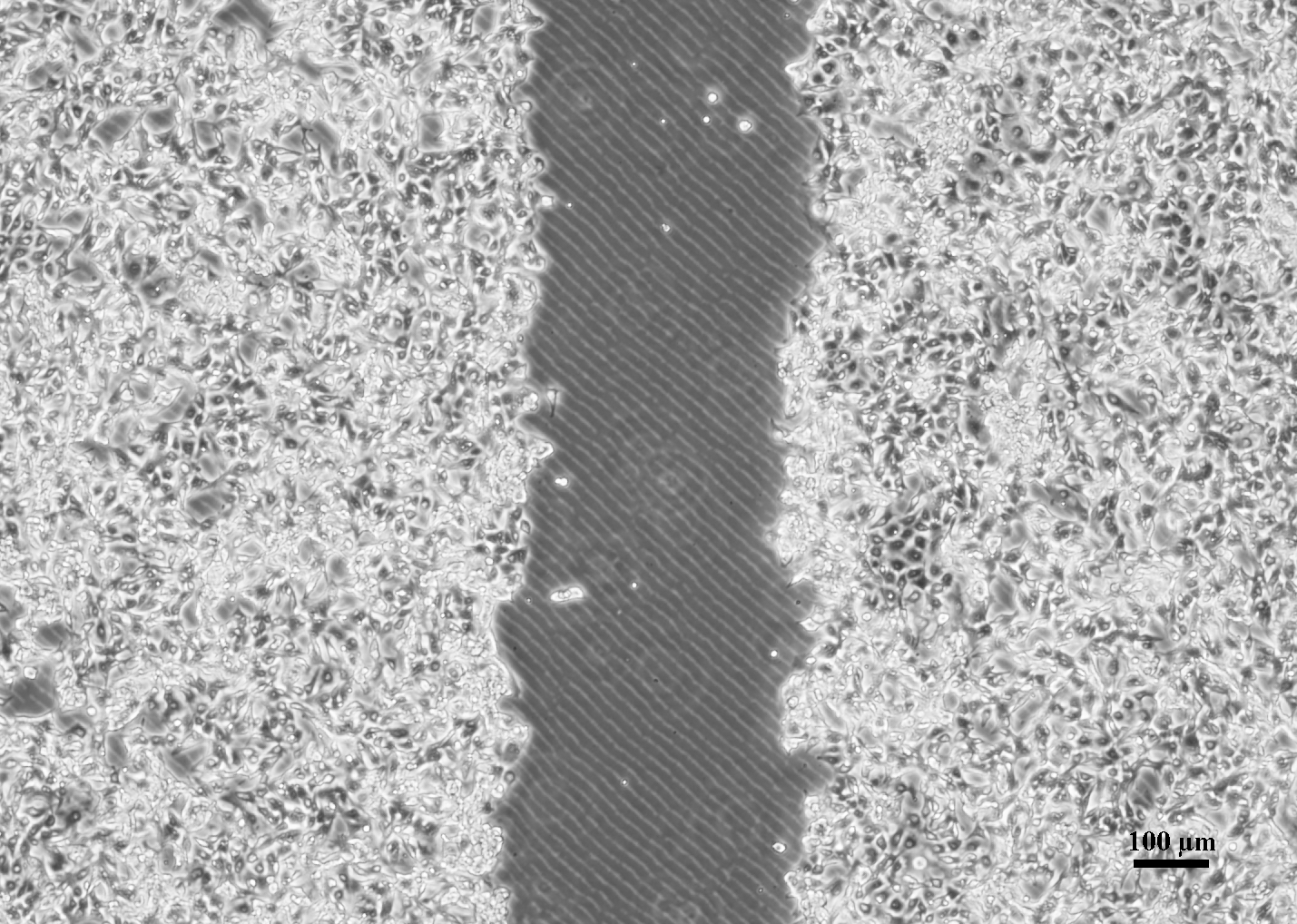

Supplement: Supplementary file 6 [file DataSheet1.zip › Figure 1 and 2/Figure 2/Figure 2C D E G/Figure 2D/15MS 0H.png]

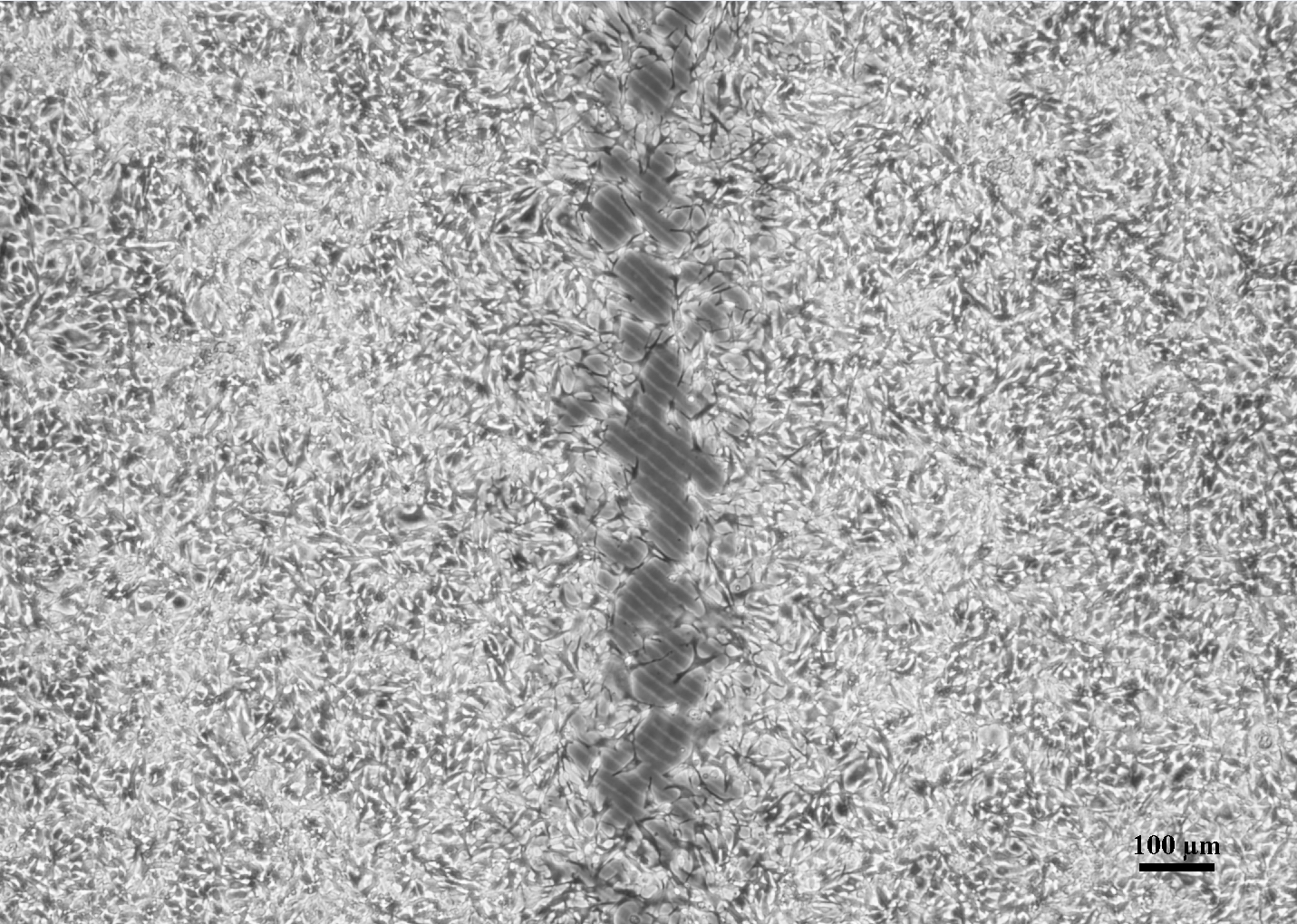

Supplement: Supplementary file 6 [file DataSheet1.zip › Figure 1 and 2/Figure 2/Figure 2C D E G/Figure 2D/15MS 24H.png]

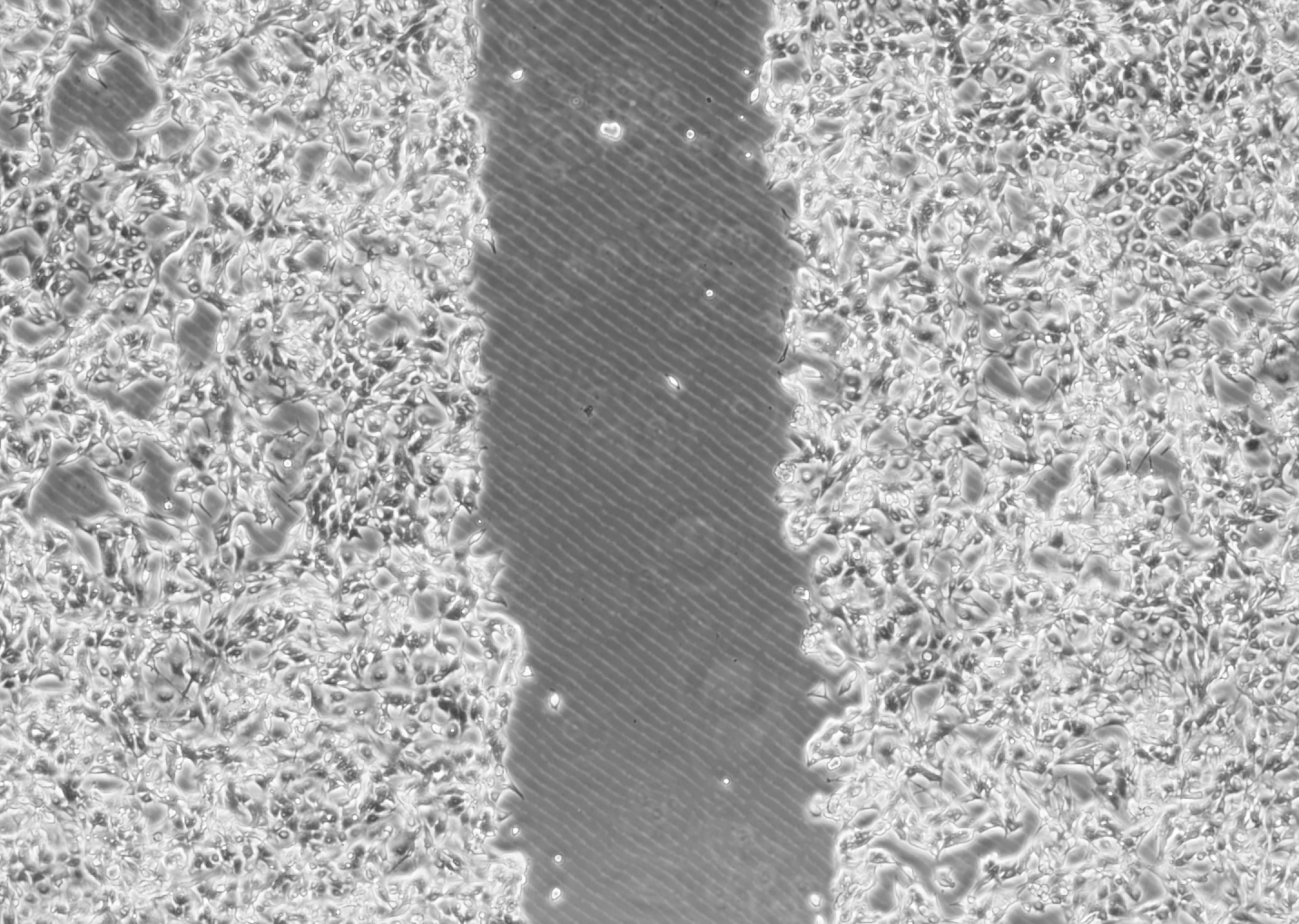

Supplement: Supplementary file 6 [file DataSheet1.zip › Figure 1 and 2/Figure 2/Figure 2C D E G/Figure 2D/5MS 0H.png]

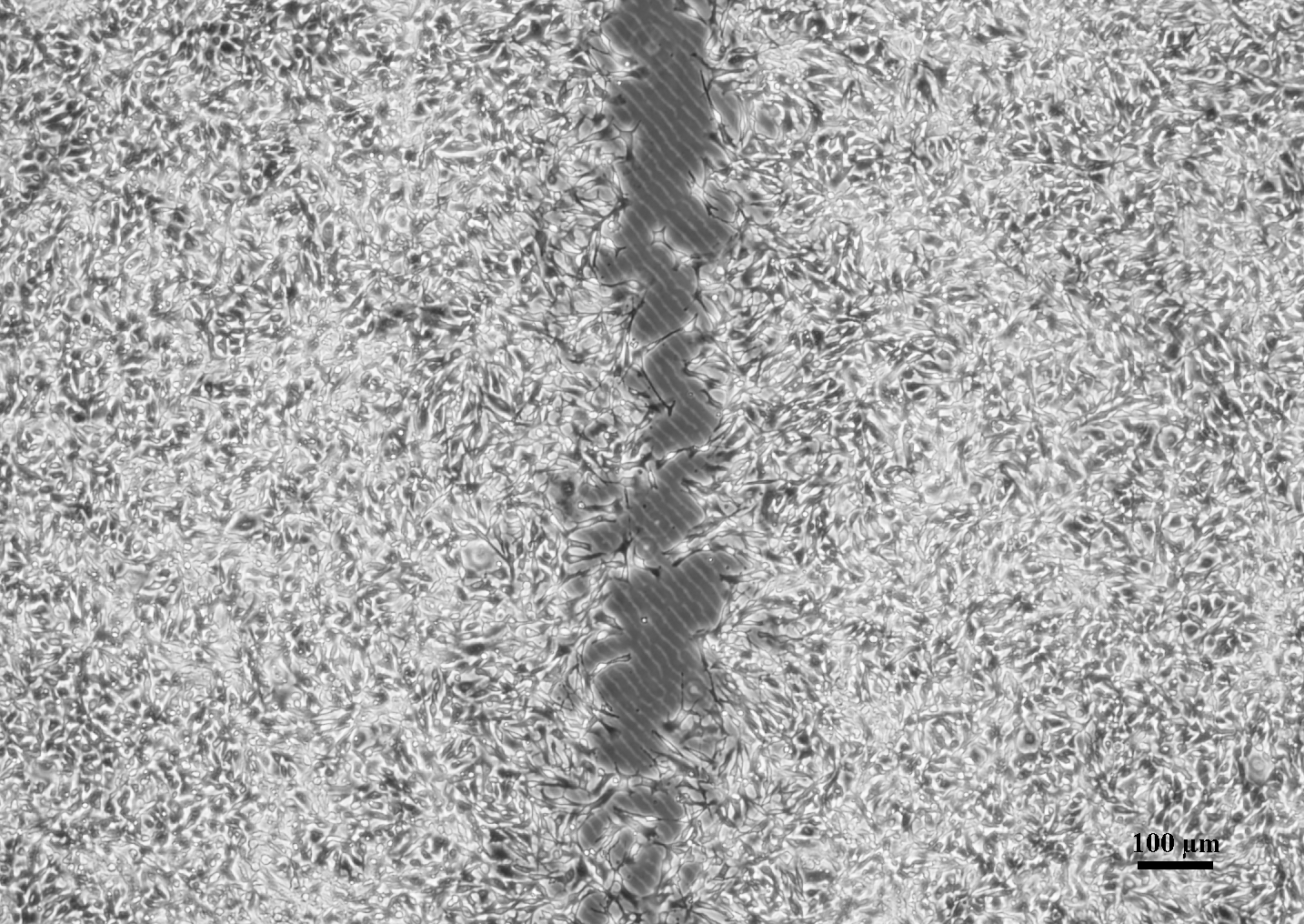

Supplement: Supplementary file 6 [file DataSheet1.zip › Figure 1 and 2/Figure 2/Figure 2C D E G/Figure 2D/5MS 24H.png]

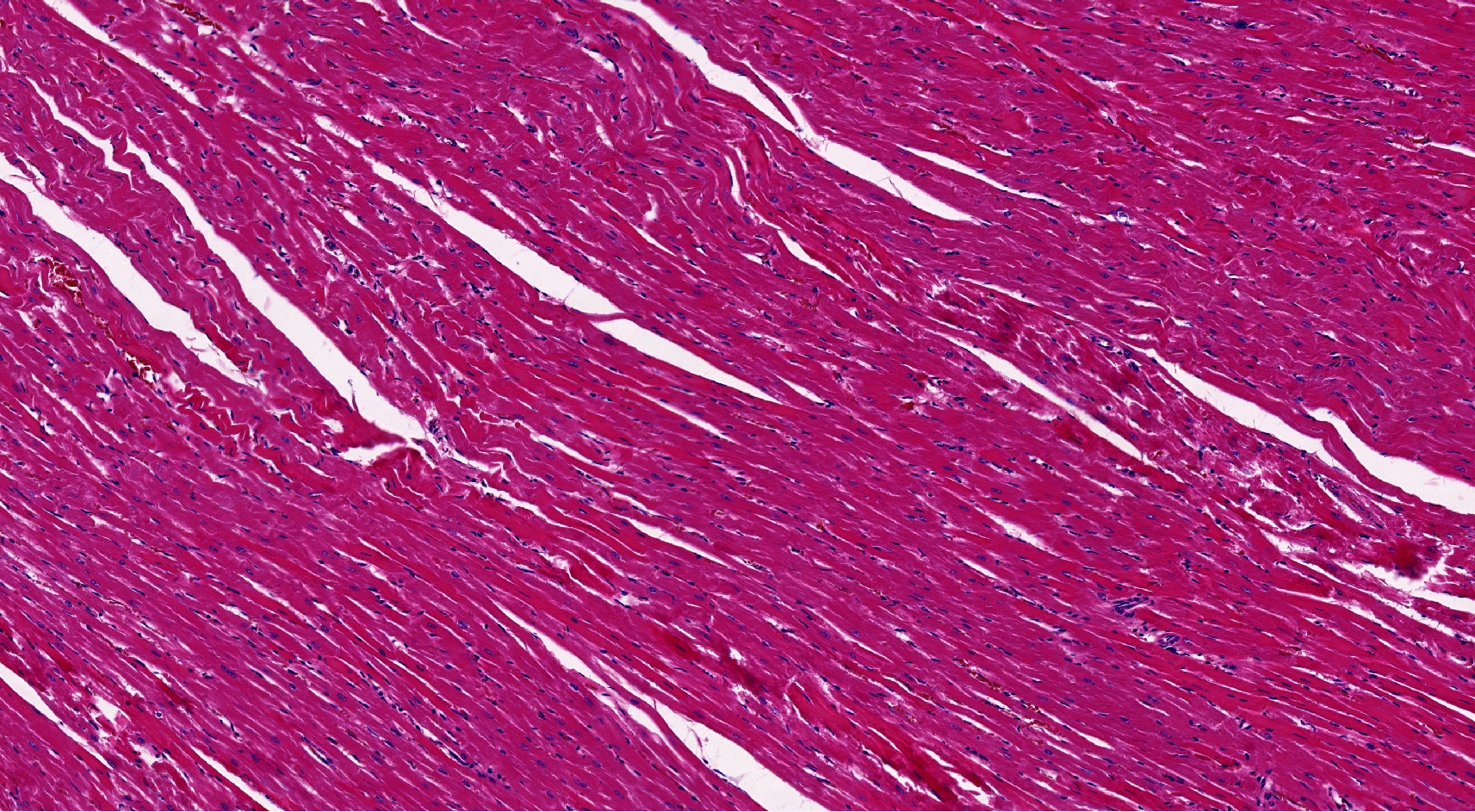

Supplement: Supplementary file 7 [file DataSheet6.zip › Figure 9 and 10/Blank-Heart.png]

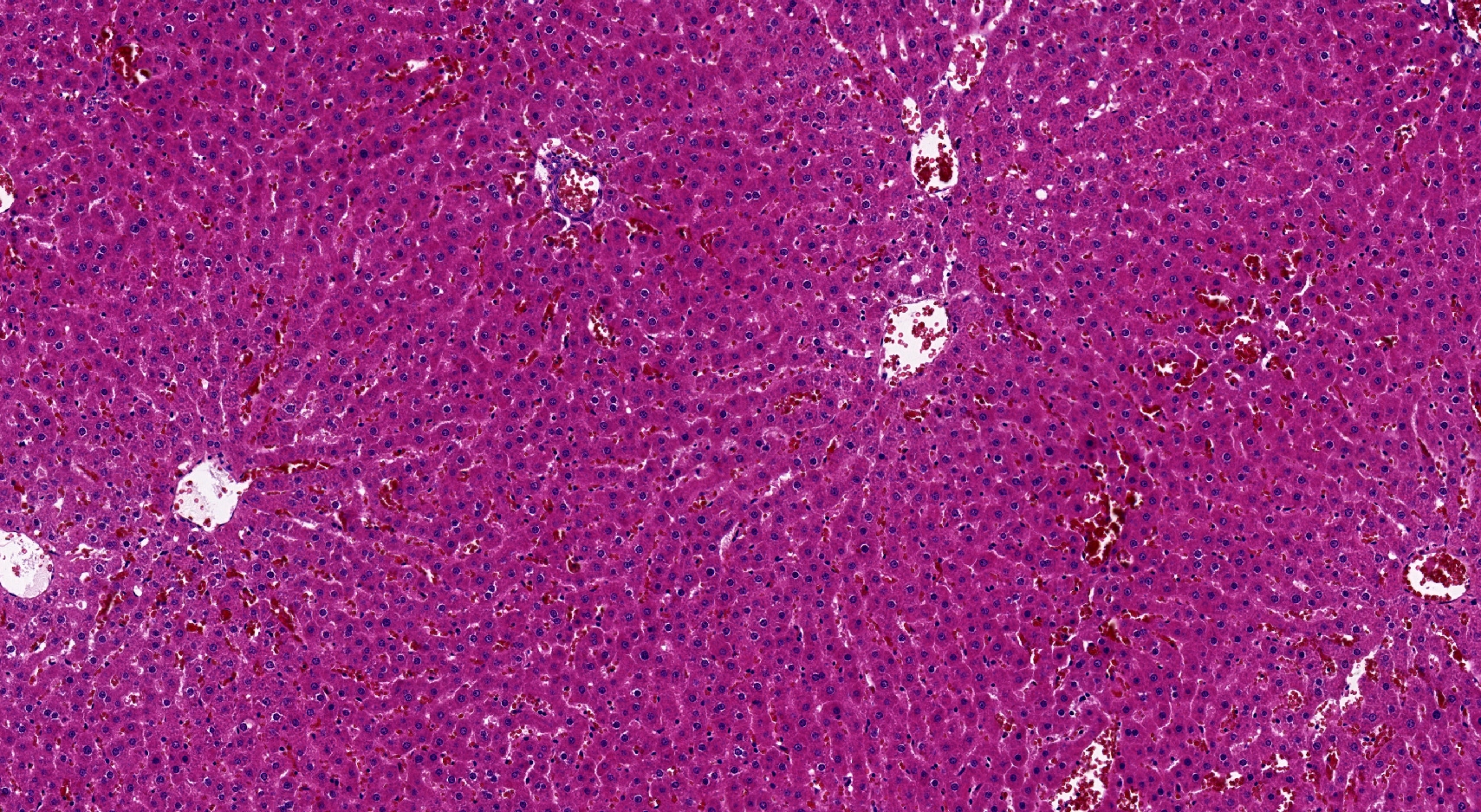

Supplement: Supplementary file 7 [file DataSheet6.zip › Figure 9 and 10/Blank-Liver.png]

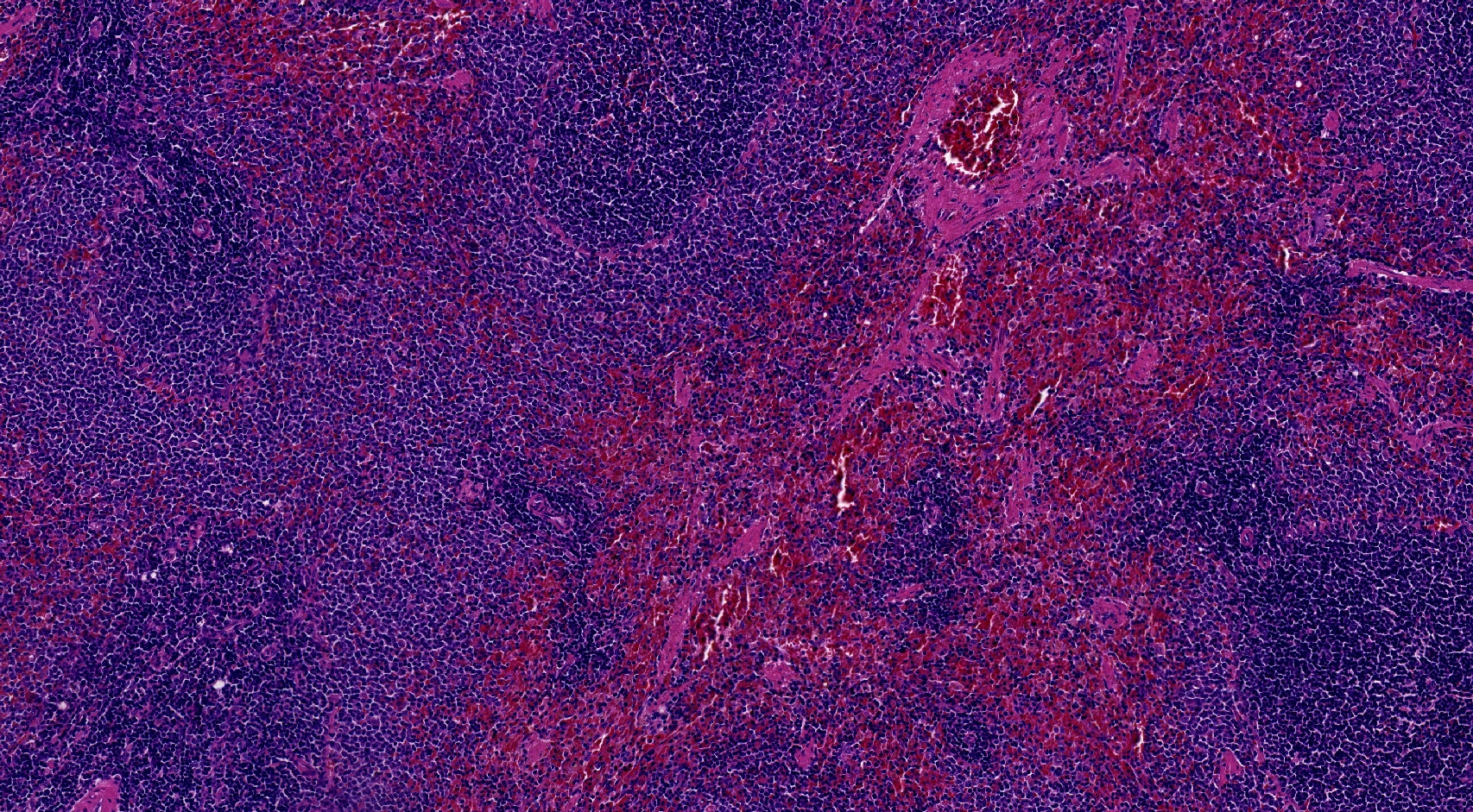

Supplement: Supplementary file 7 [file DataSheet6.zip › Figure 9 and 10/Blank-Spleen.png]

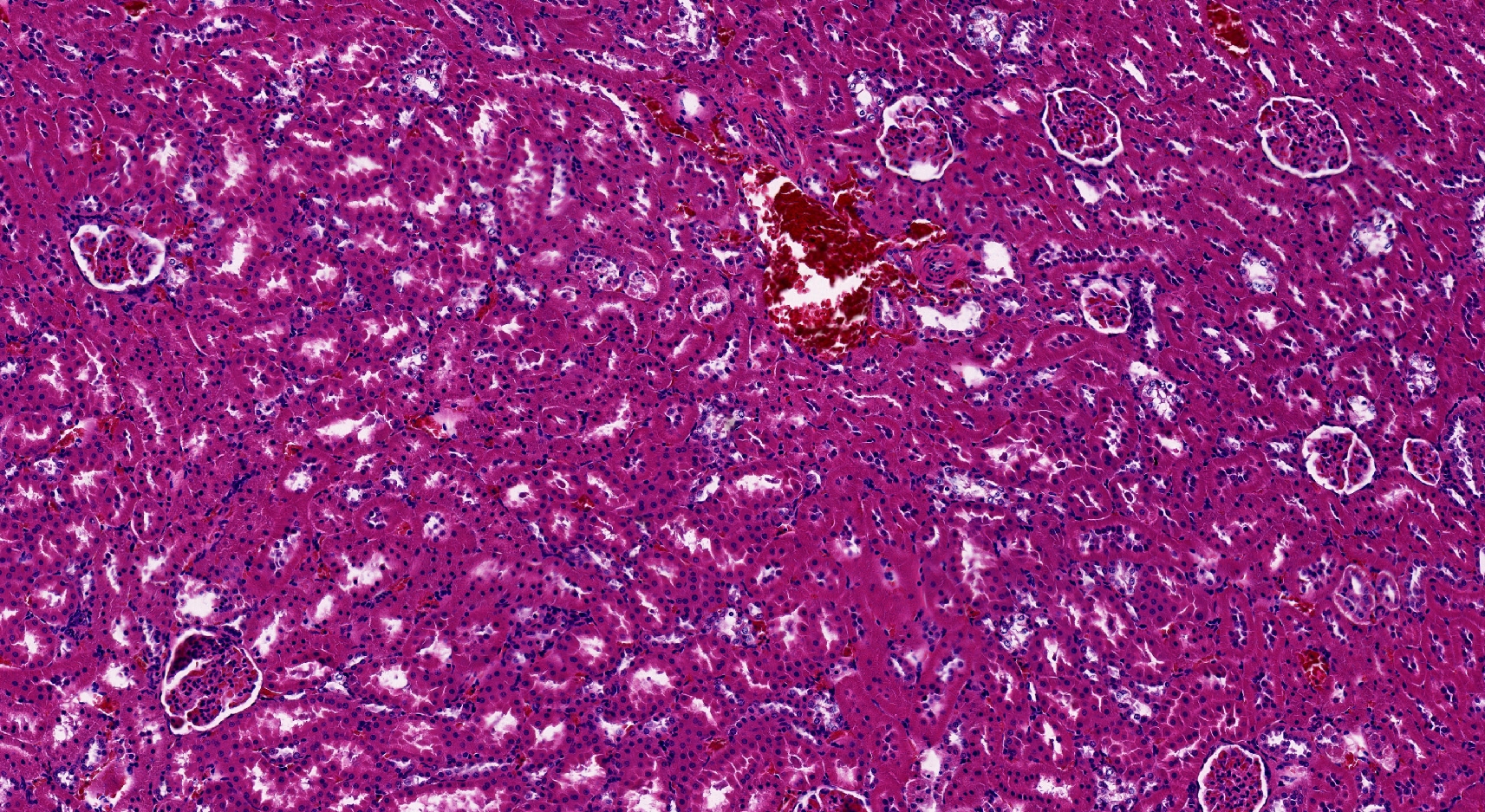

Supplement: Supplementary file 7 [file DataSheet6.zip › Figure 9 and 10/Blank-kidney.png]

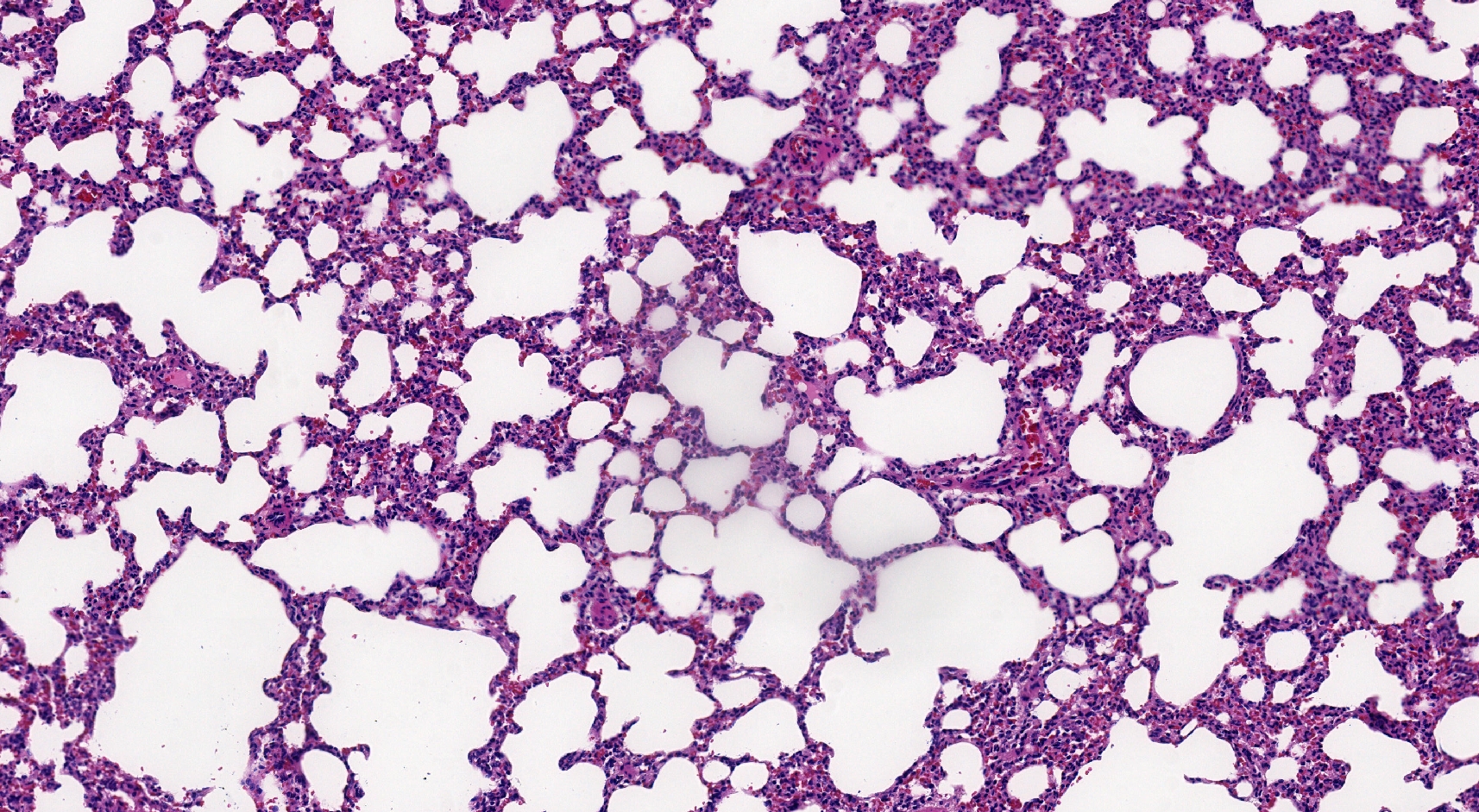

Supplement: Supplementary file 7 [file DataSheet6.zip › Figure 9 and 10/Blank-lung.png]

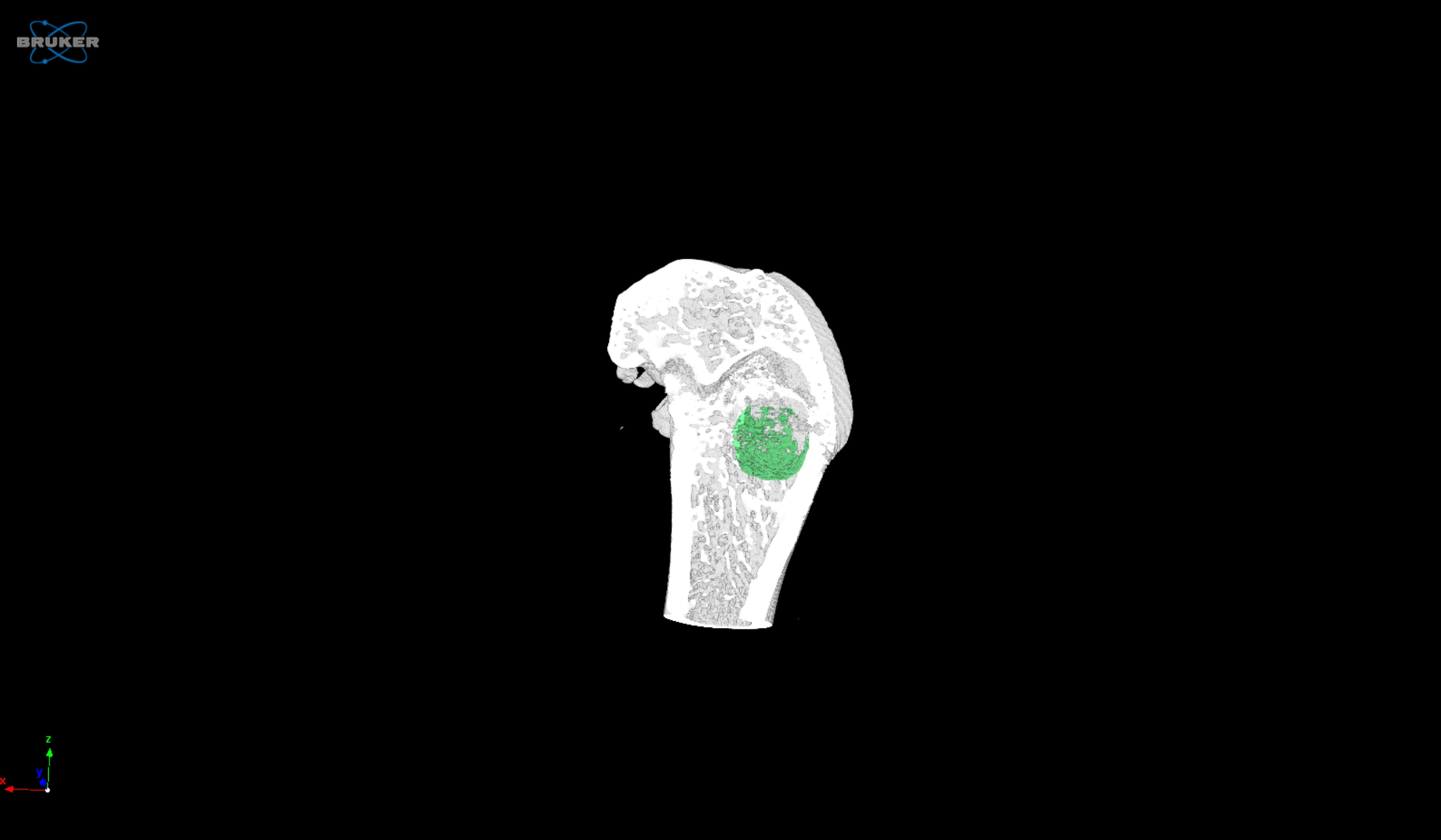

Supplement: Supplementary file 7 [file DataSheet6.zip › Figure 9 and 10/Figure 10/A/12W Blank.png]

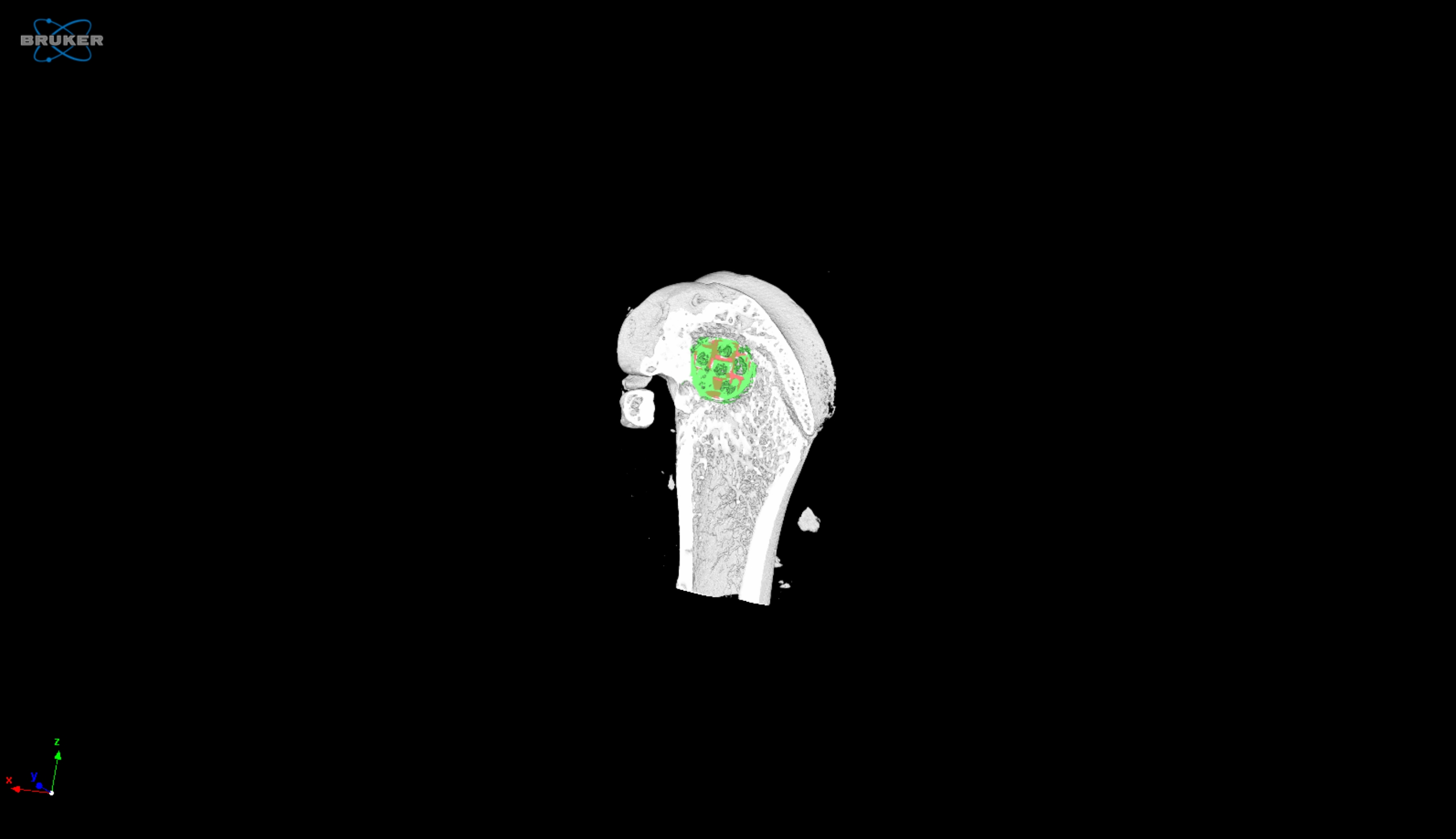

Supplement: Supplementary file 7 [file DataSheet6.zip › Figure 9 and 10/Figure 10/A/12W MS-TCP.png]

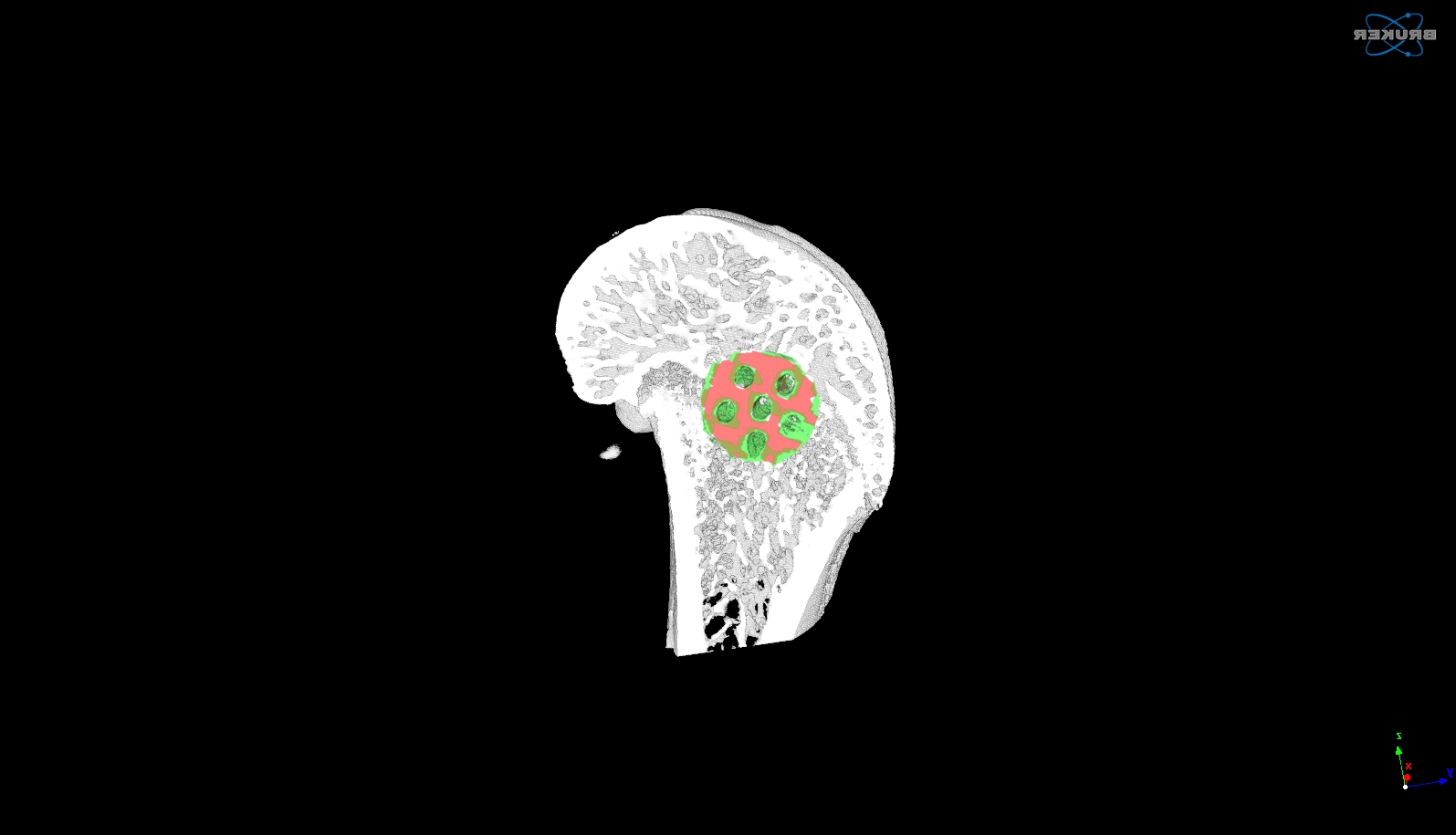

Supplement: Supplementary file 7 [file DataSheet6.zip › Figure 9 and 10/Figure 10/A/12W TCP.png]

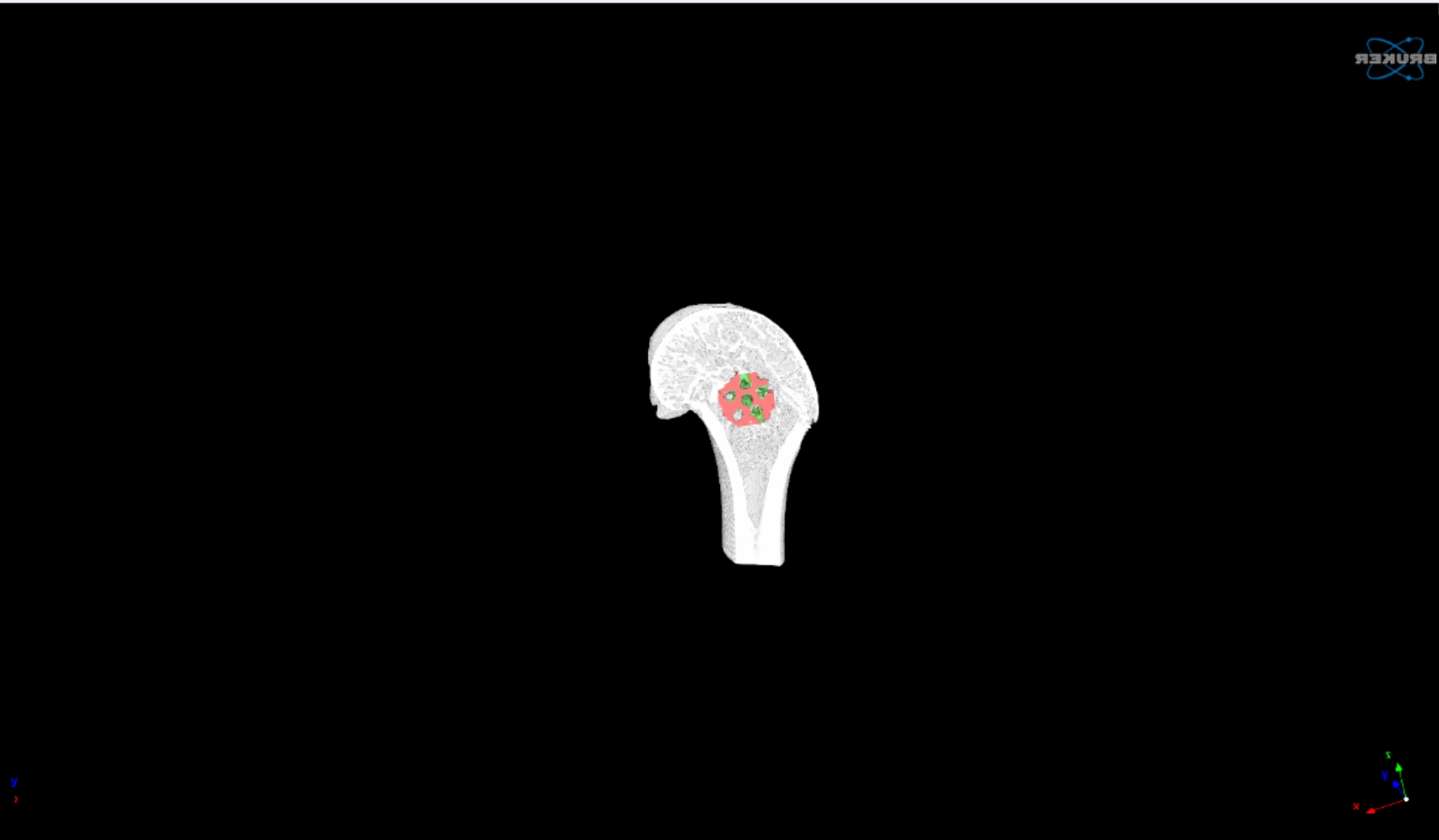

Supplement: Supplementary file 7 [file DataSheet6.zip › Figure 9 and 10/Figure 10/A/4W MS-TCP.png]

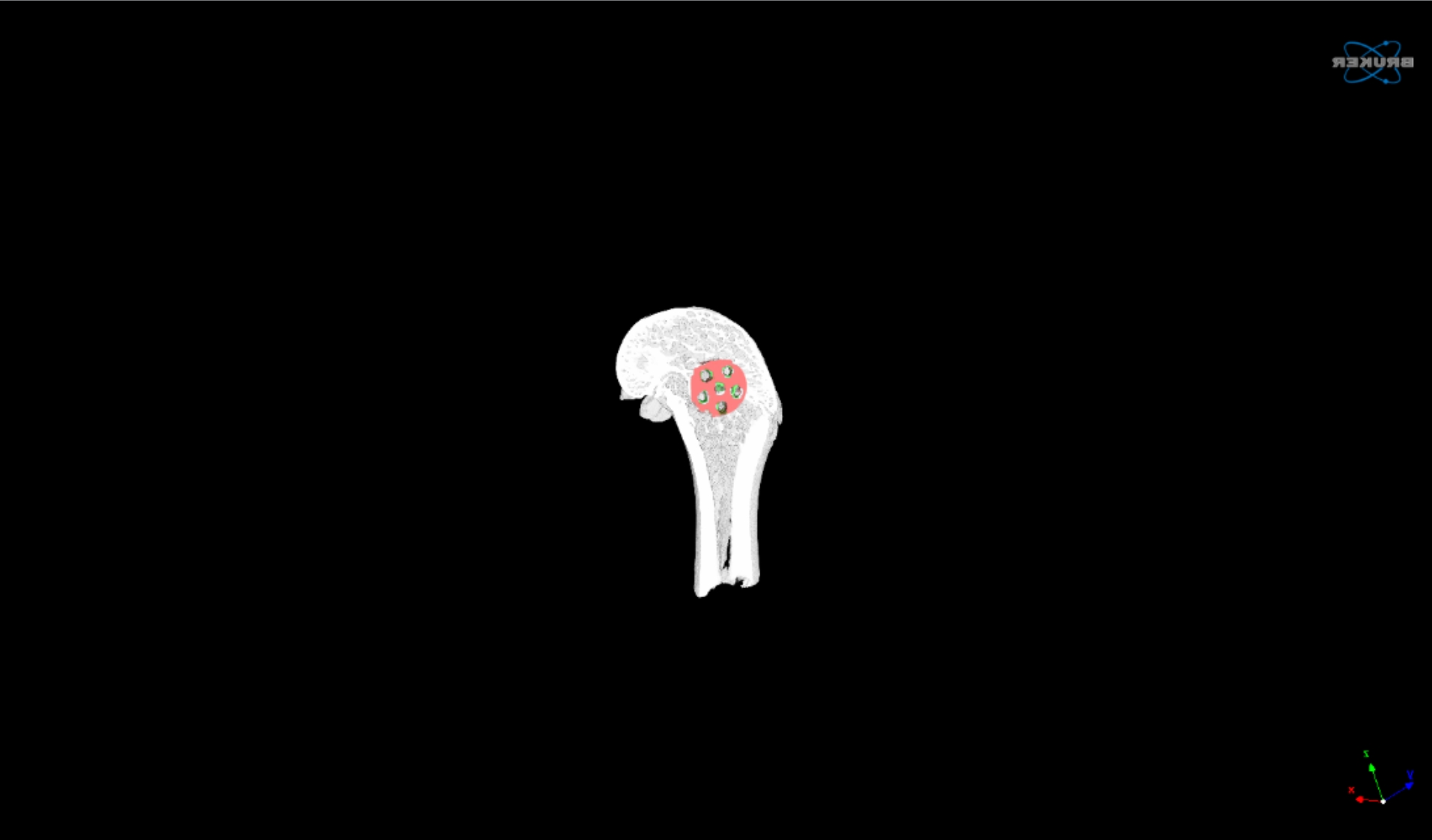

Supplement: Supplementary file 7 [file DataSheet6.zip › Figure 9 and 10/Figure 10/A/4W TCP.png]

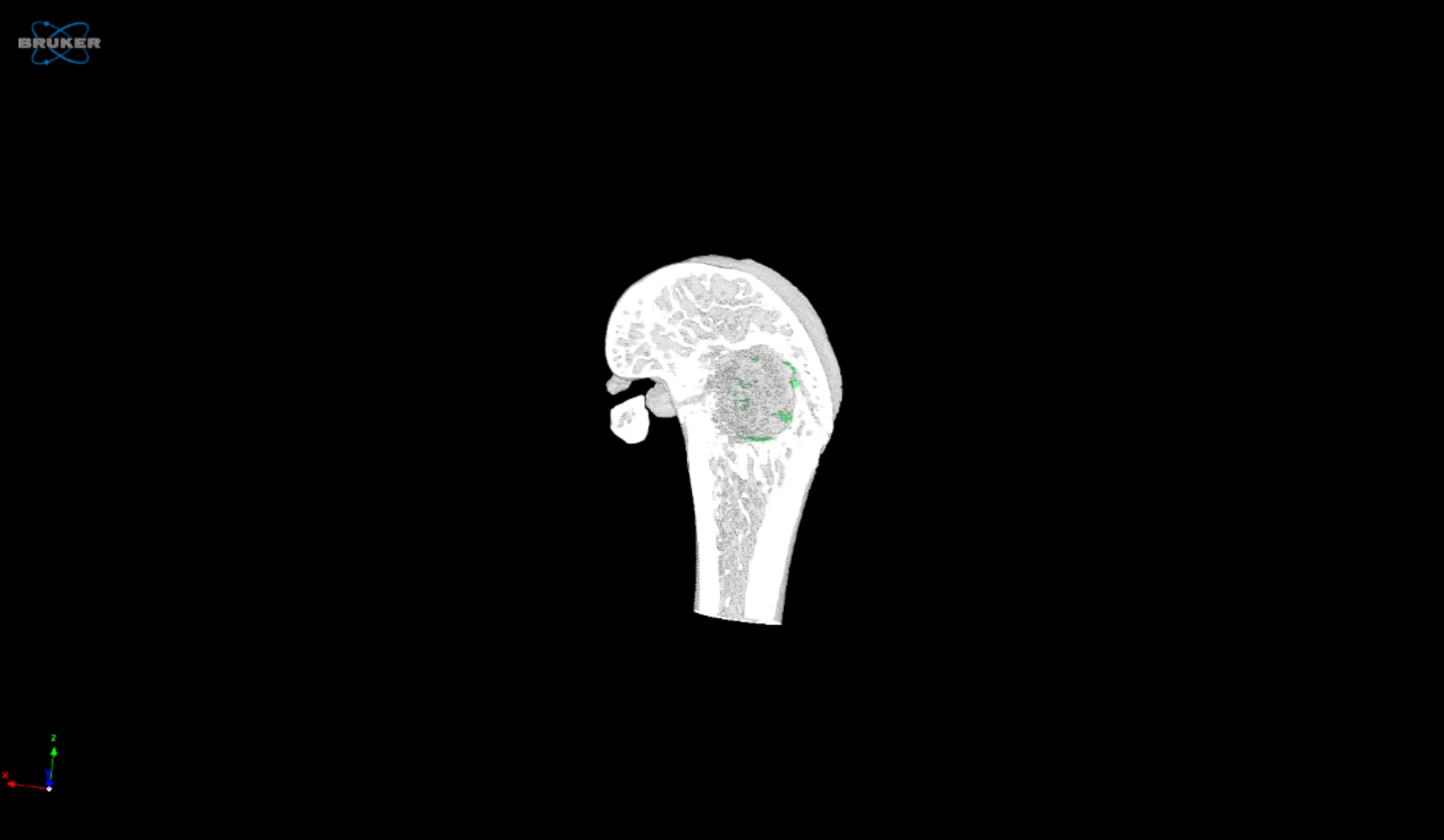

Supplement: Supplementary file 7 [file DataSheet6.zip › Figure 9 and 10/Figure 10/A/4w Blank.png]
