# Supplementary material for: 3D printed magnesium silicate/β-tricalcium phosphate scaffolds promote coupled osteogenesis and angiogenesis
Source: Front Bioeng Biotechnol. 2025 Jan 31;12:1518145. doi: 10.3389/fbioe.2024.1518145 (PMC11841418; doi:10.3389/fbioe.2024.1518145)
Supplement: Supplementary file 4 [file Table1.docx]

**Supplemental material**

**
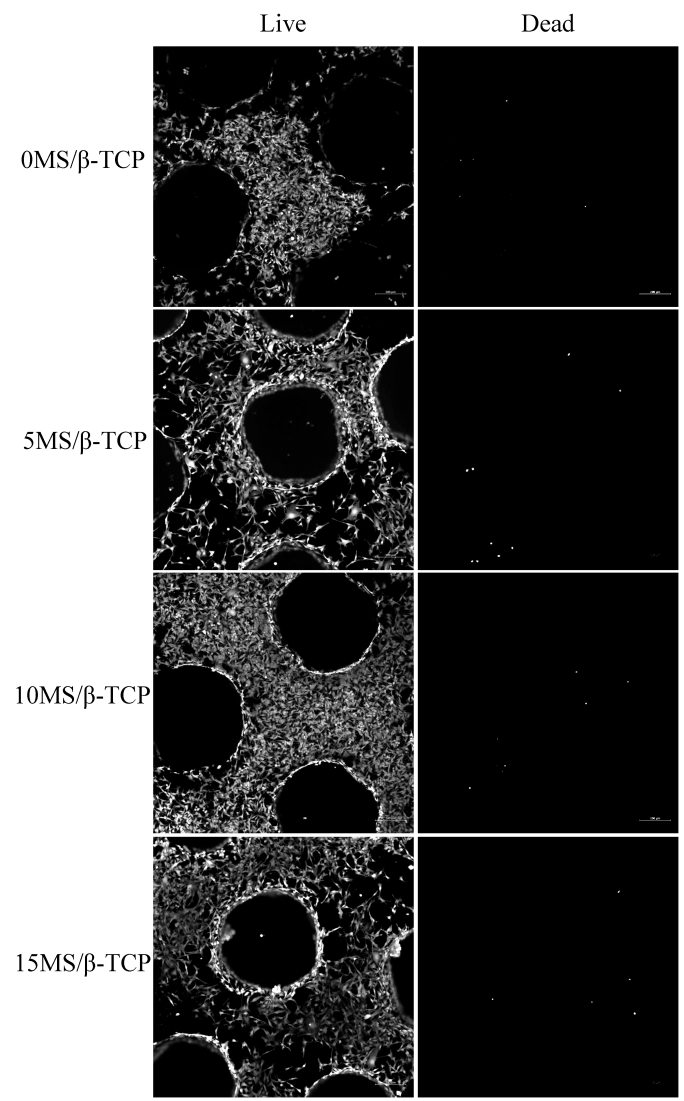
**

**Figure 2A** Representative greyscale images of the live and dead channel of MC3T3-E1 cells cultured on scaffolds.

**
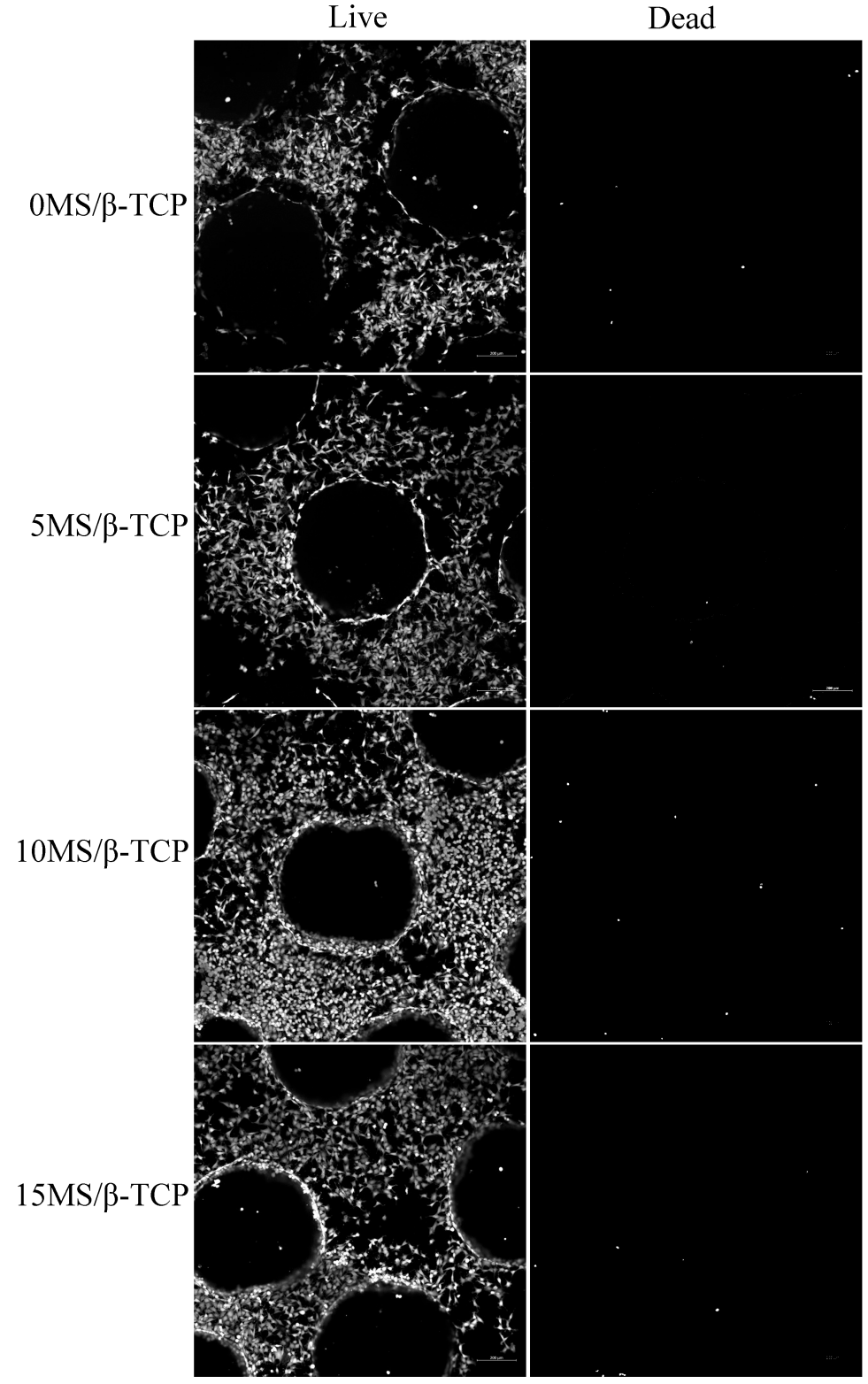
**

**Figure 3A** Representative greyscale images of the live and dead channel of HUVECs cells cultured on scaffolds.
