# Supplementary figures and images for: 3D printed magnesium silicate/β-tricalcium phosphate scaffolds promote coupled osteogenesis and angiogenesis (part 2 of 2)
Source: Front Bioeng Biotechnol. 2025 Jan 31;12:1518145. doi: 10.3389/fbioe.2024.1518145 (PMC11841418; doi:10.3389/fbioe.2024.1518145)

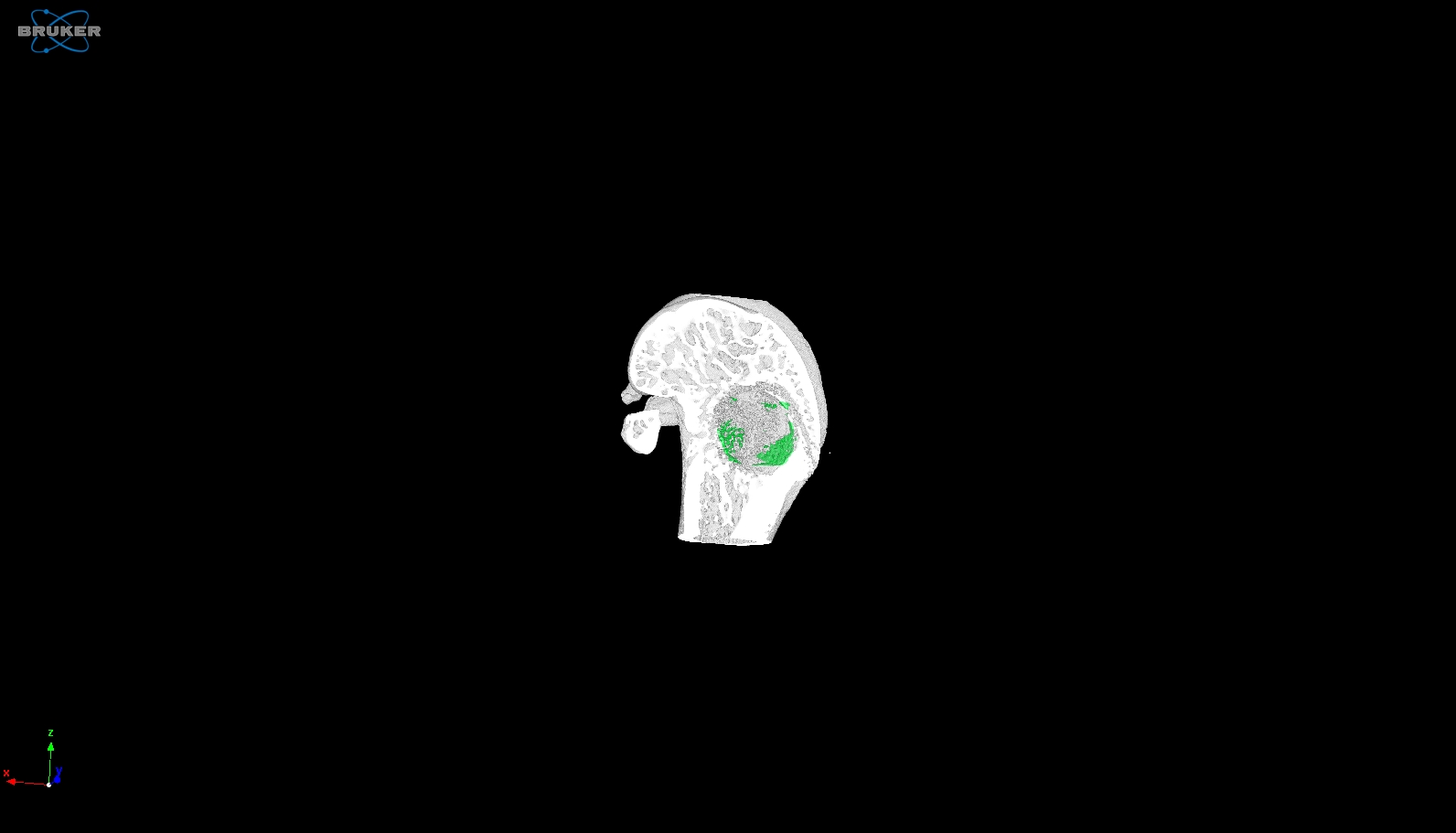

Supplement: Supplementary file 7 [file DataSheet6.zip › Figure 9 and 10/Figure 10/A/8W Blank.png]

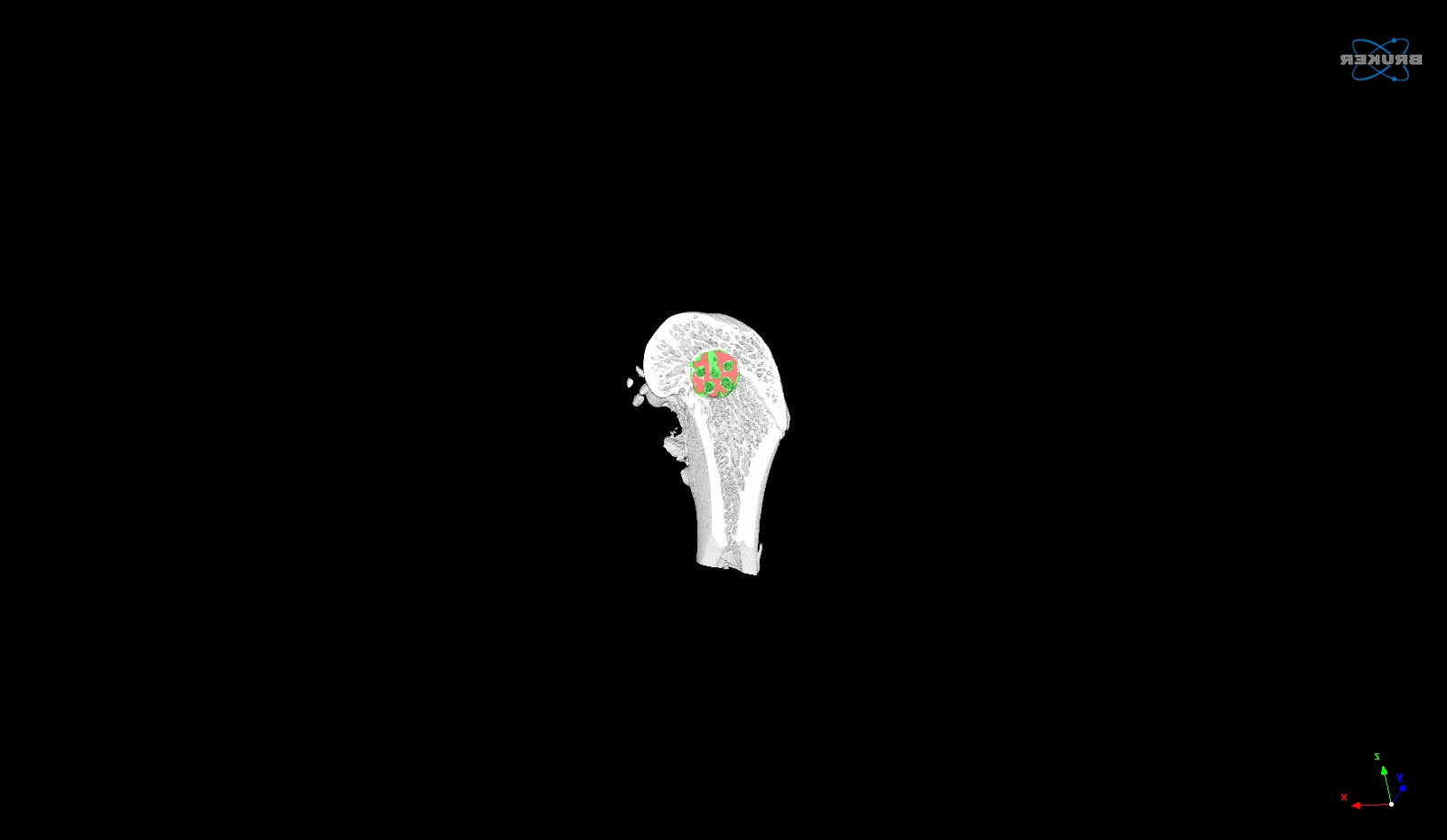

Supplement: Supplementary file 7 [file DataSheet6.zip › Figure 9 and 10/Figure 10/A/8W MS-TCP.png]

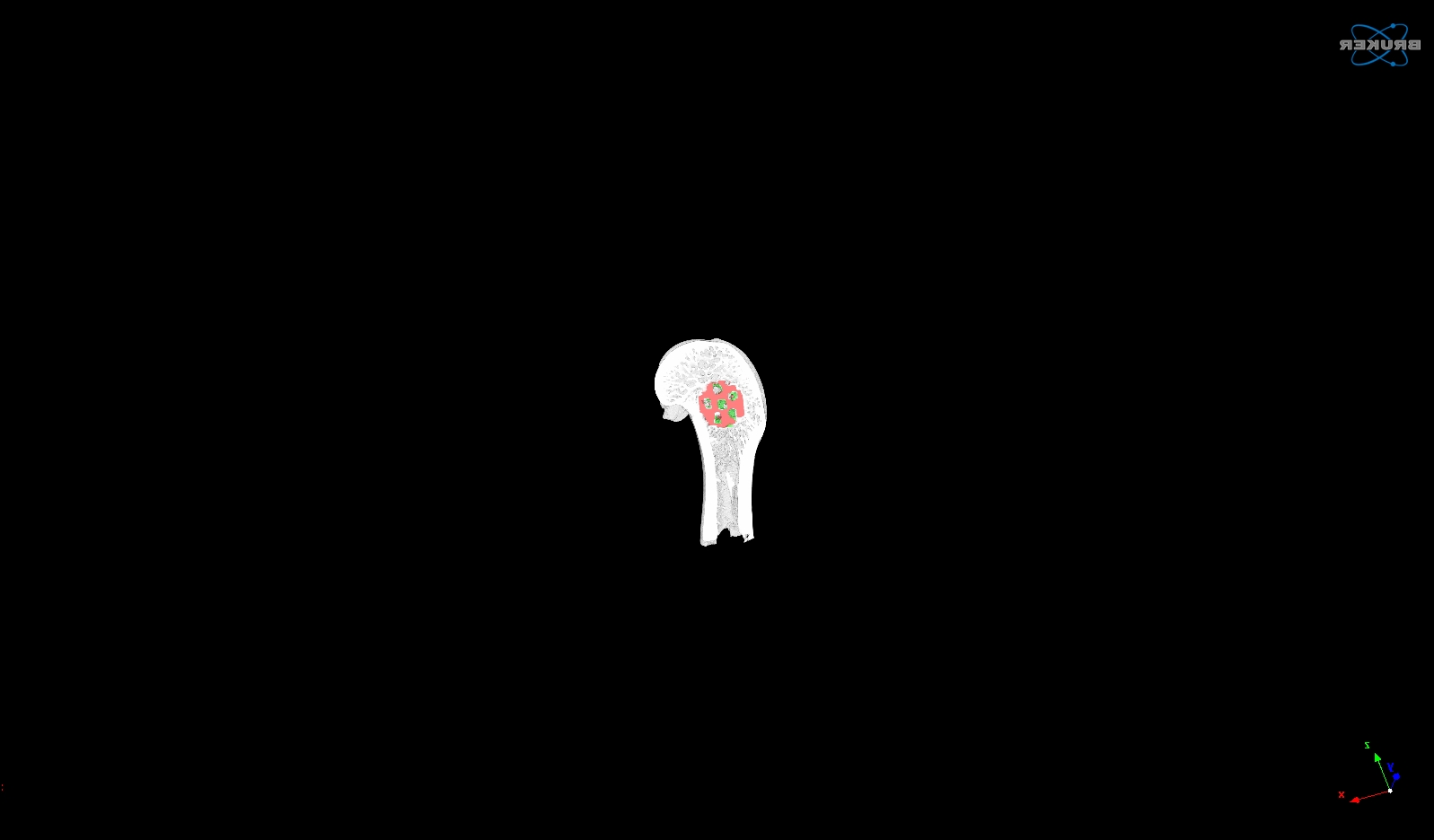

Supplement: Supplementary file 7 [file DataSheet6.zip › Figure 9 and 10/Figure 10/A/8W TCP.png]

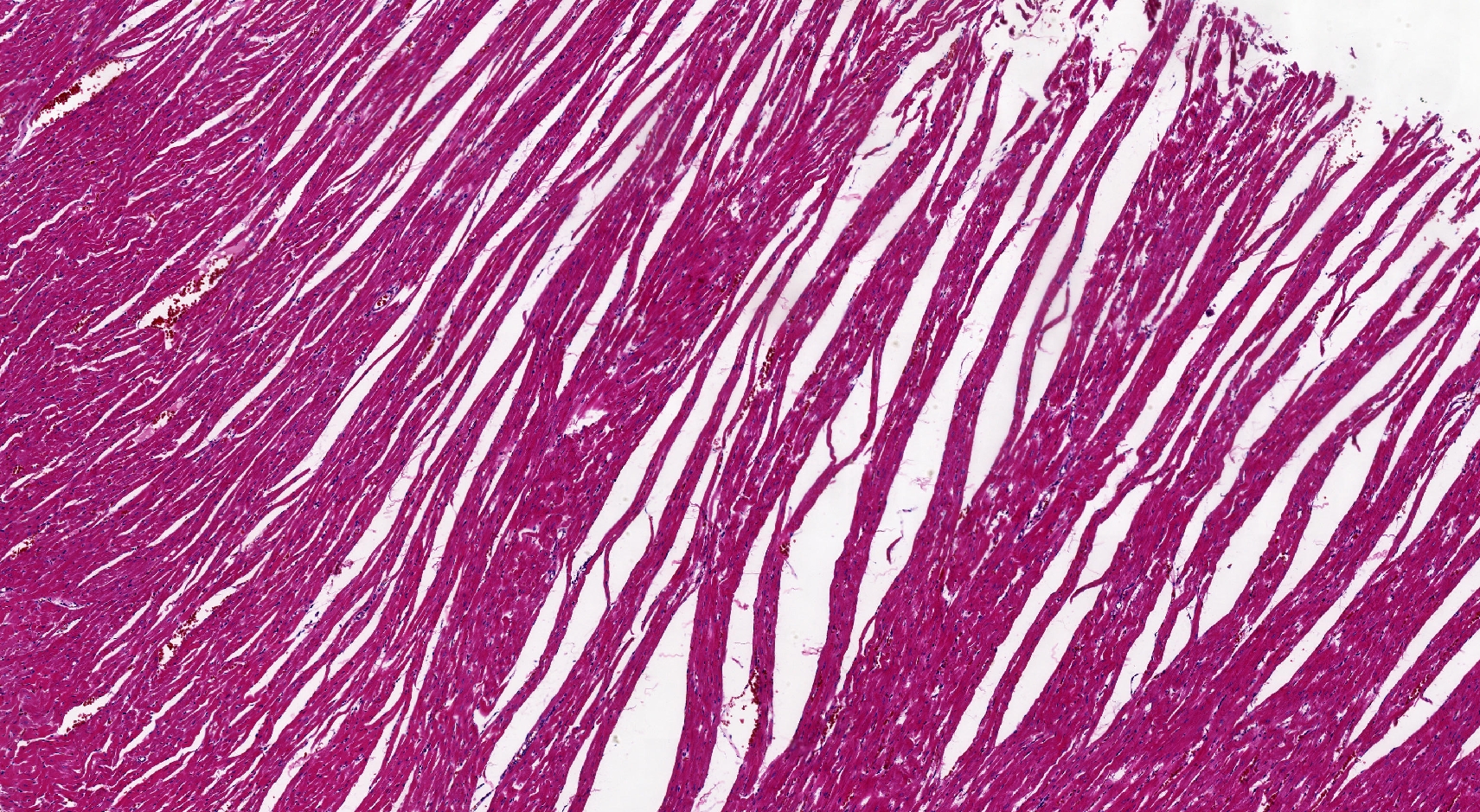

Supplement: Supplementary file 7 [file DataSheet6.zip › Figure 9 and 10/MS-Heart.png]

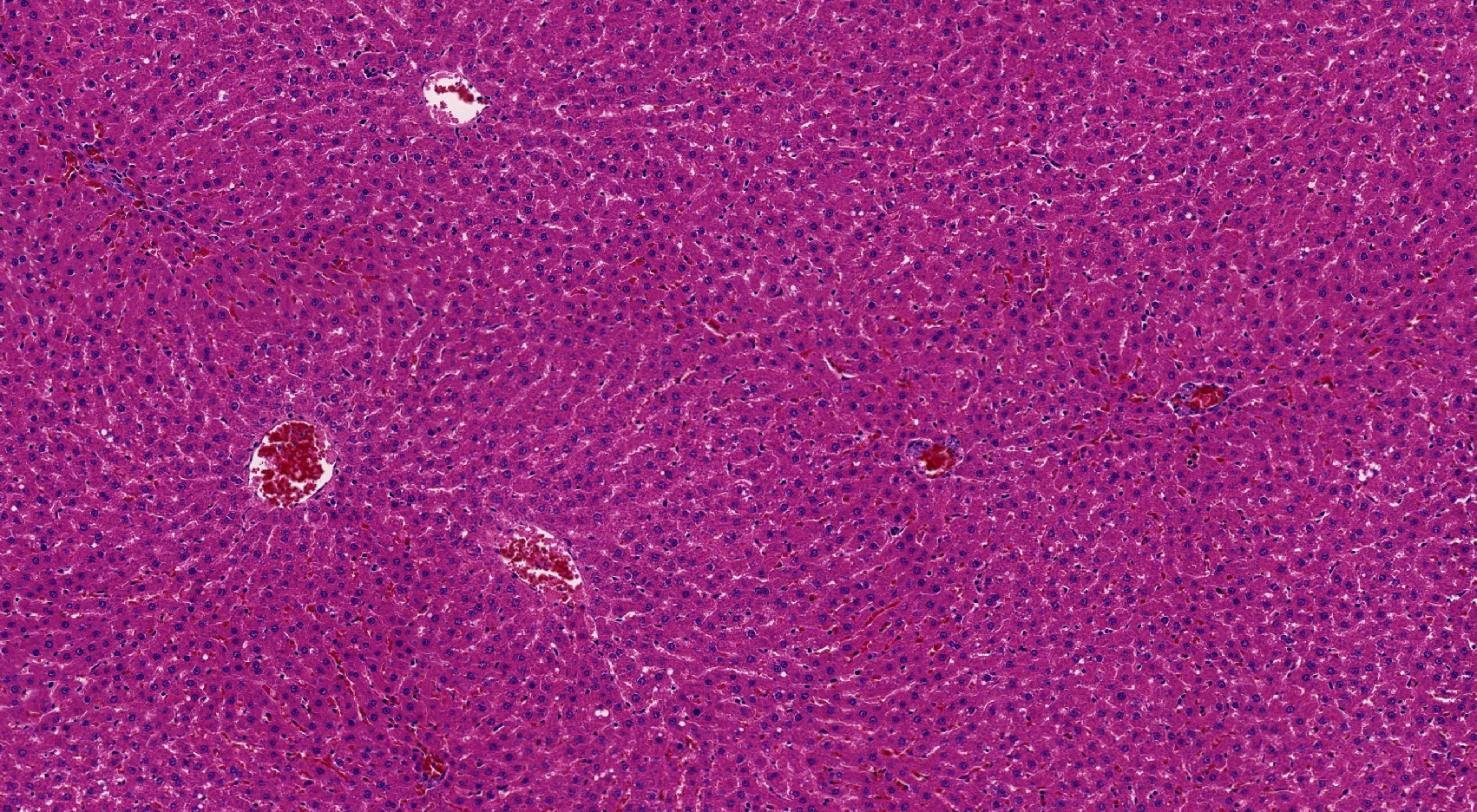

Supplement: Supplementary file 7 [file DataSheet6.zip › Figure 9 and 10/MS-Liver.png]

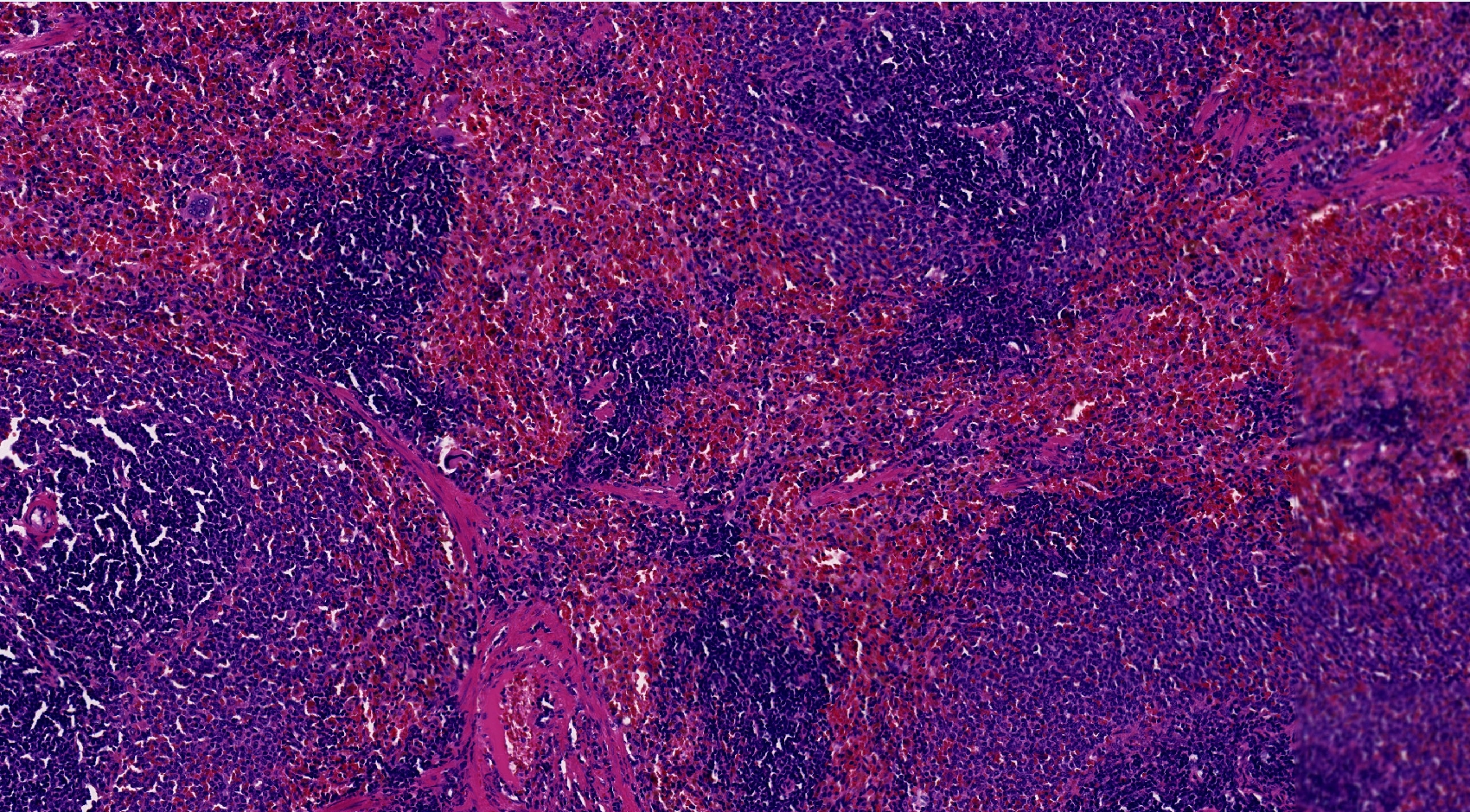

Supplement: Supplementary file 7 [file DataSheet6.zip › Figure 9 and 10/MS-Spleen.png]

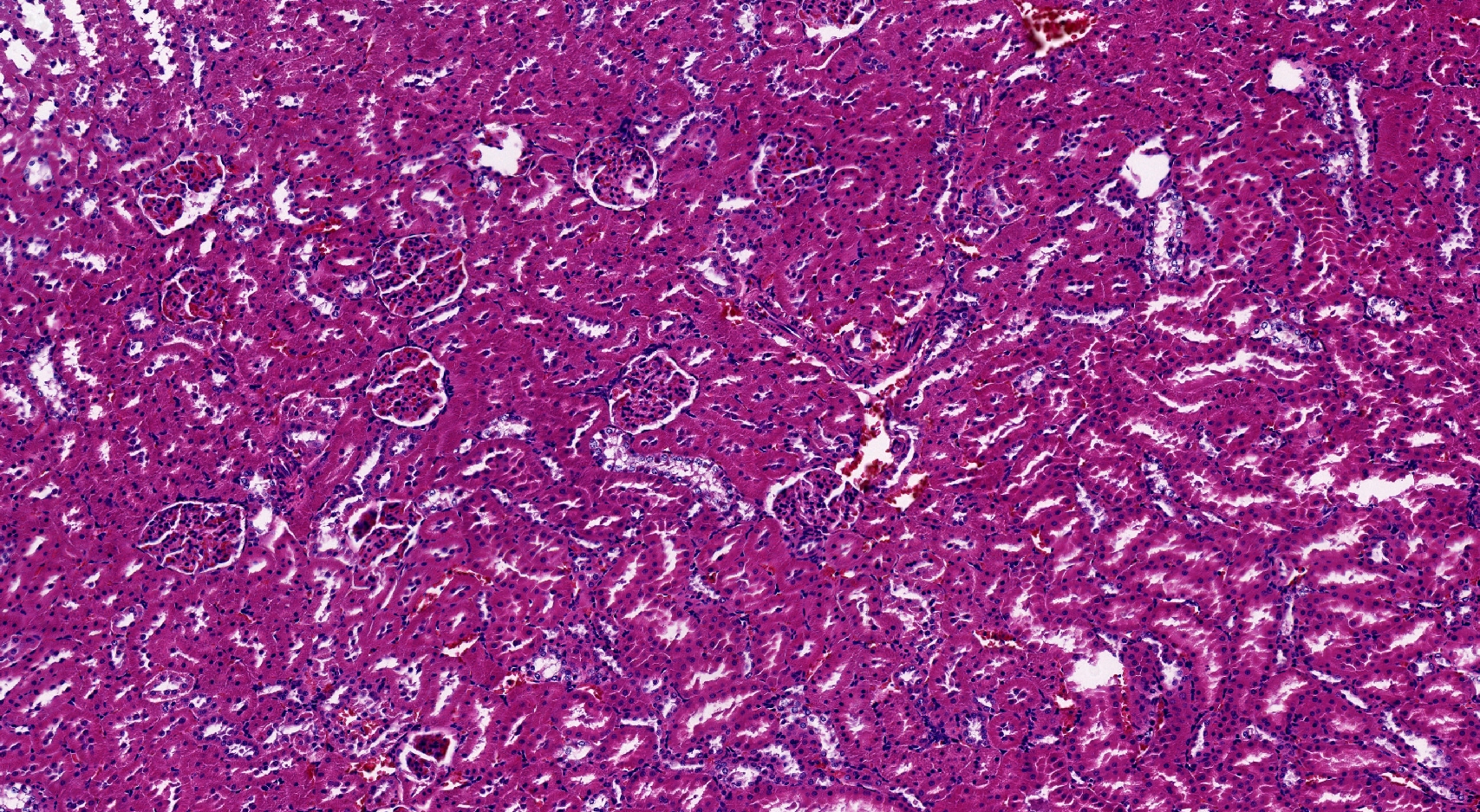

Supplement: Supplementary file 7 [file DataSheet6.zip › Figure 9 and 10/MS-kidney.png]

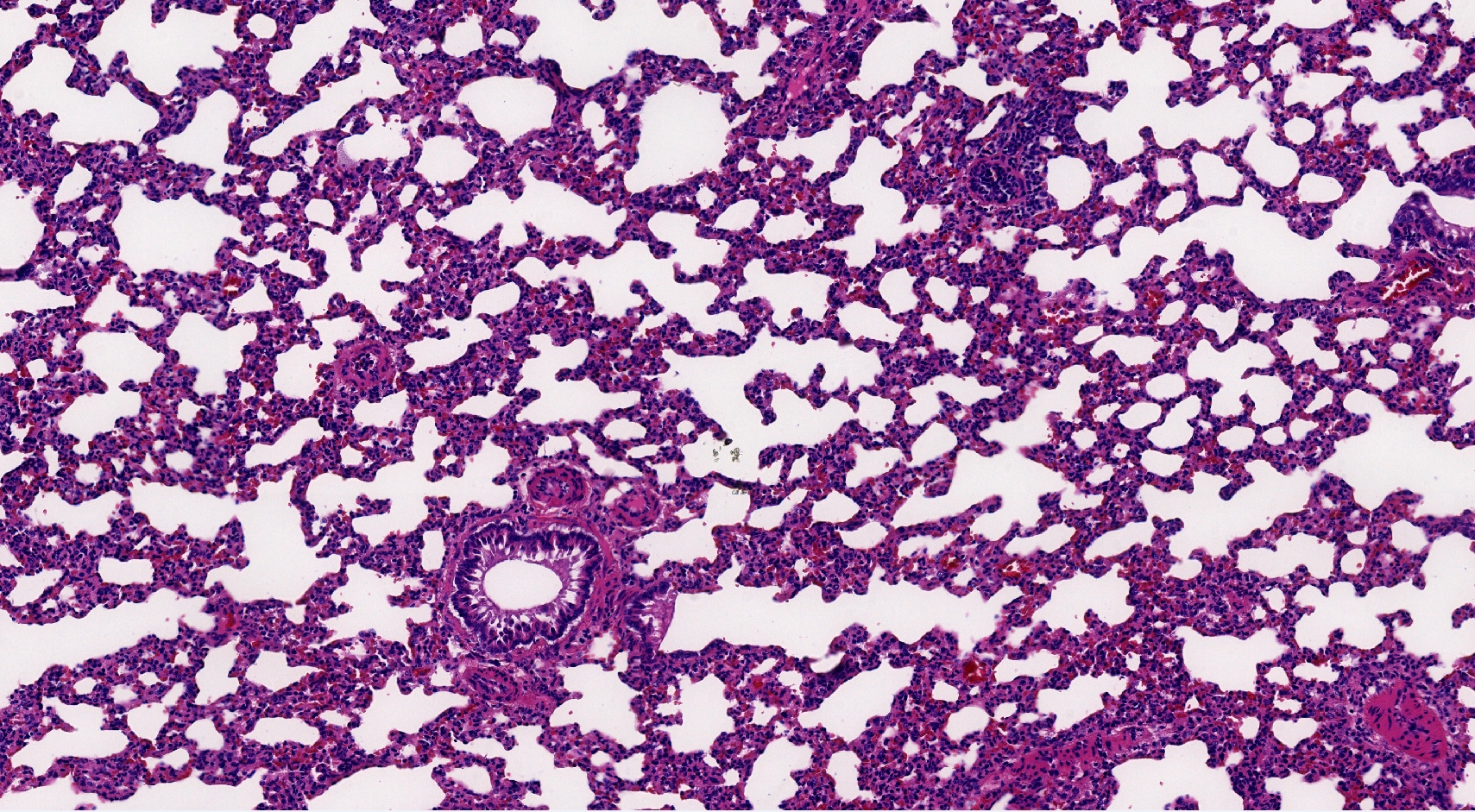

Supplement: Supplementary file 7 [file DataSheet6.zip › Figure 9 and 10/MS-lung.png]

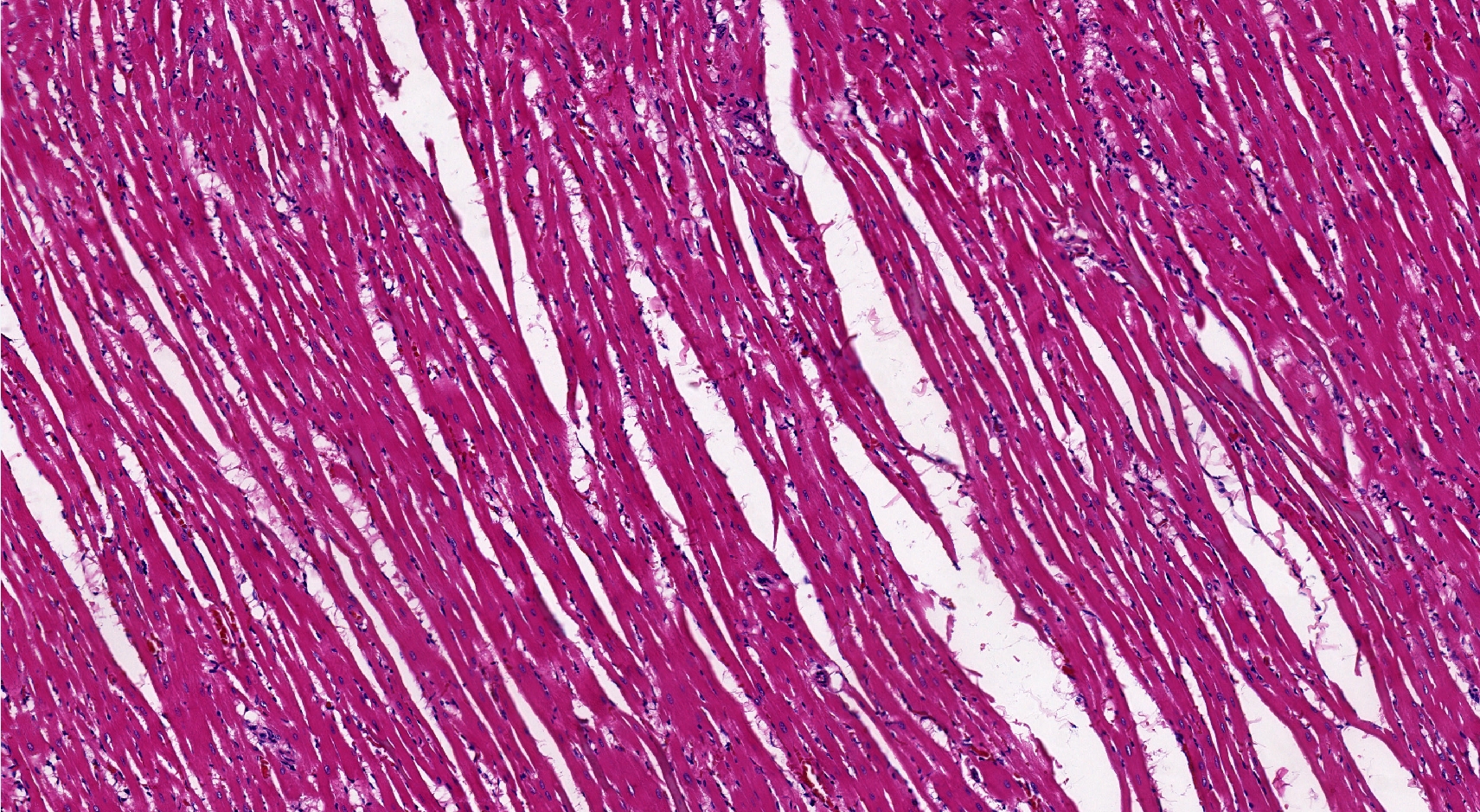

Supplement: Supplementary file 7 [file DataSheet6.zip › Figure 9 and 10/TCP-Heart.png]

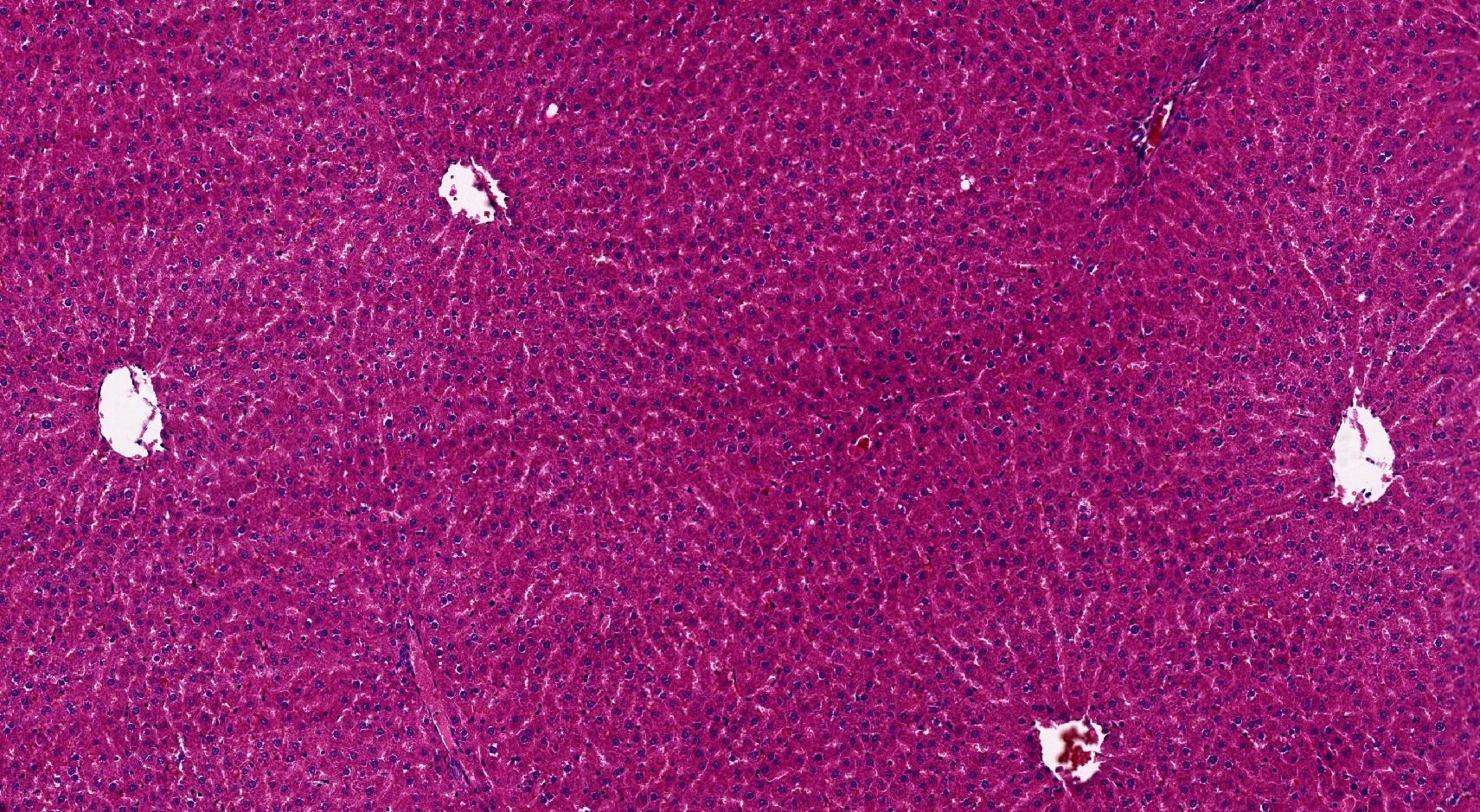

Supplement: Supplementary file 7 [file DataSheet6.zip › Figure 9 and 10/TCP-Liver.png]

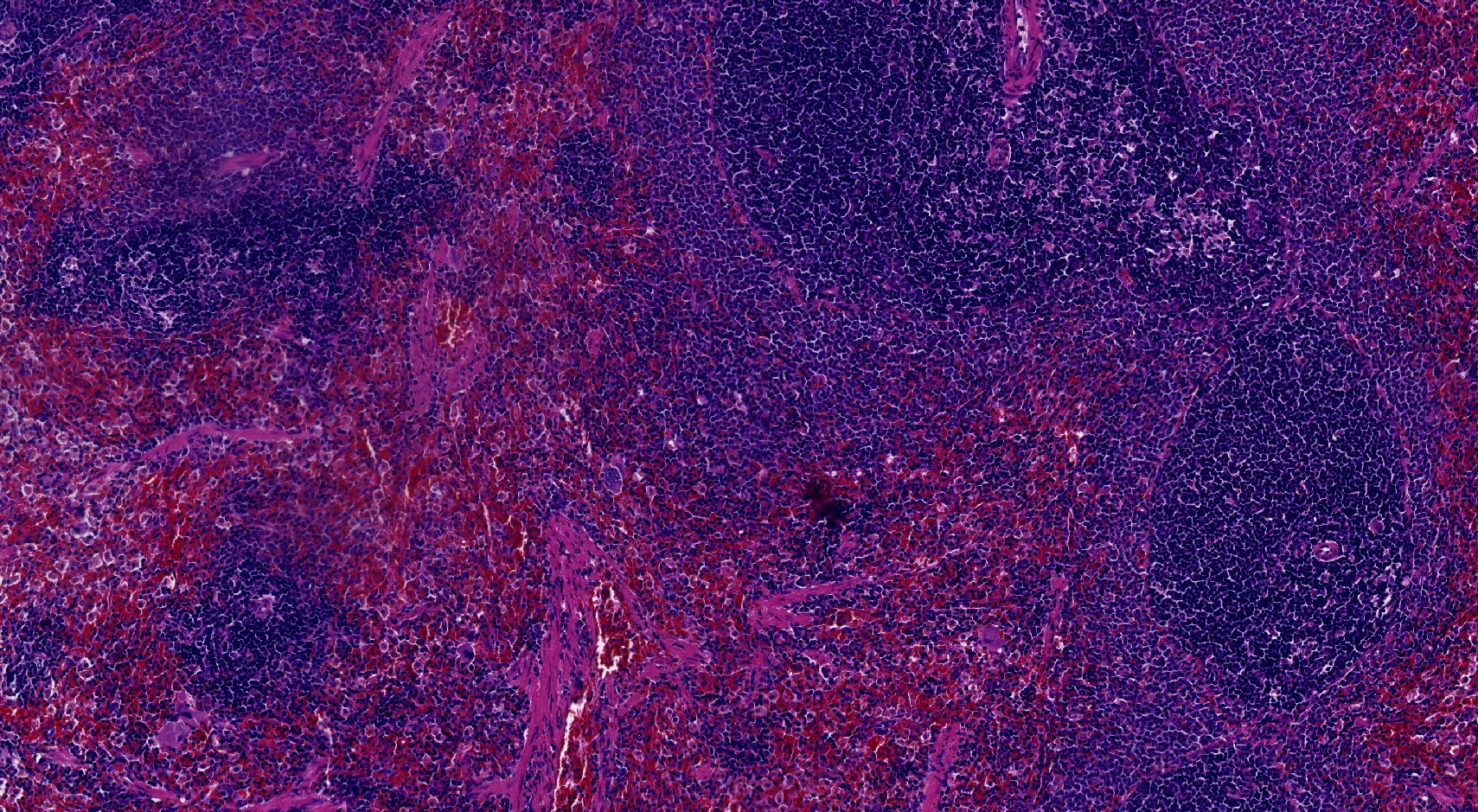

Supplement: Supplementary file 7 [file DataSheet6.zip › Figure 9 and 10/TCP-Spleen.png]

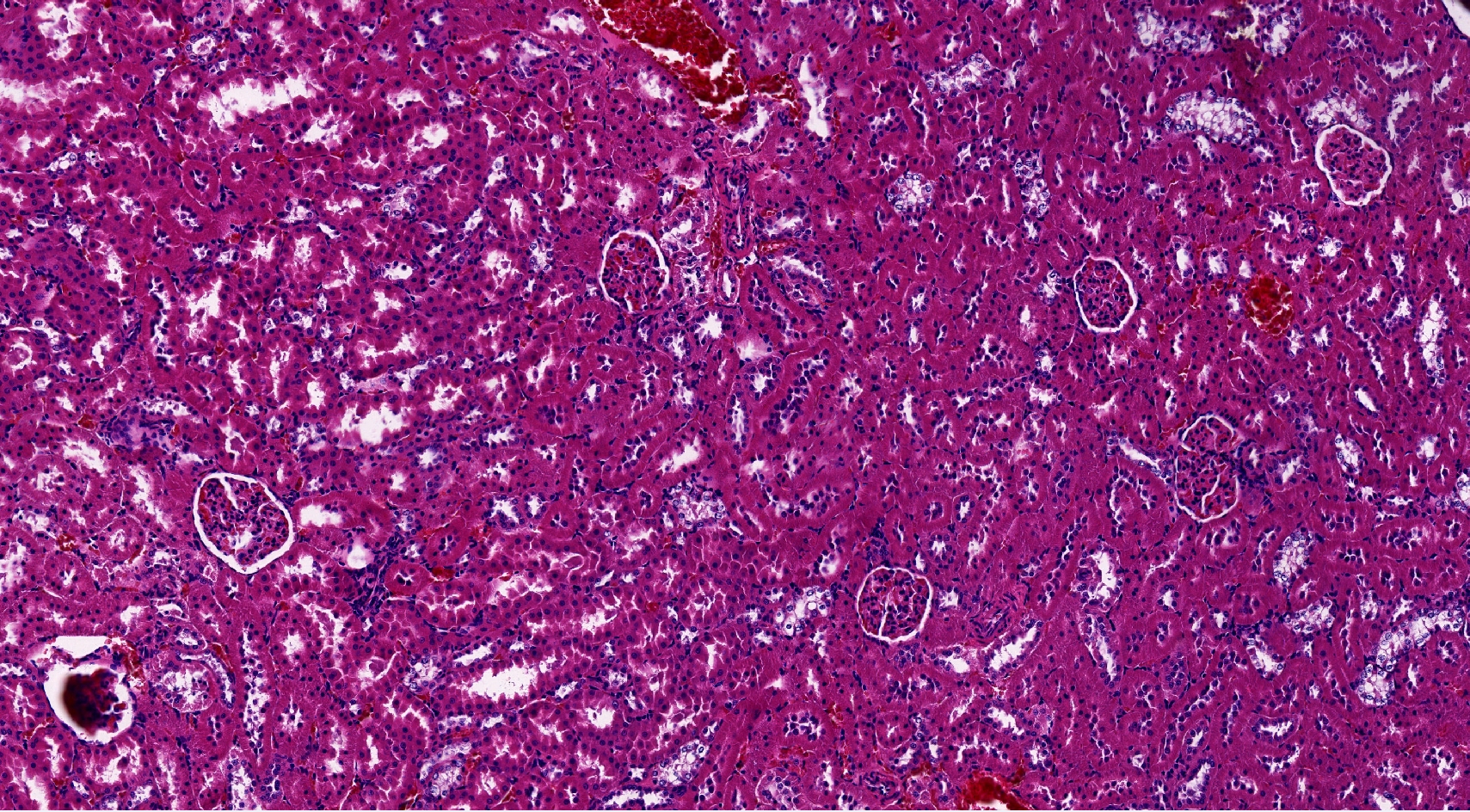

Supplement: Supplementary file 7 [file DataSheet6.zip › Figure 9 and 10/TCP-kidney.png]

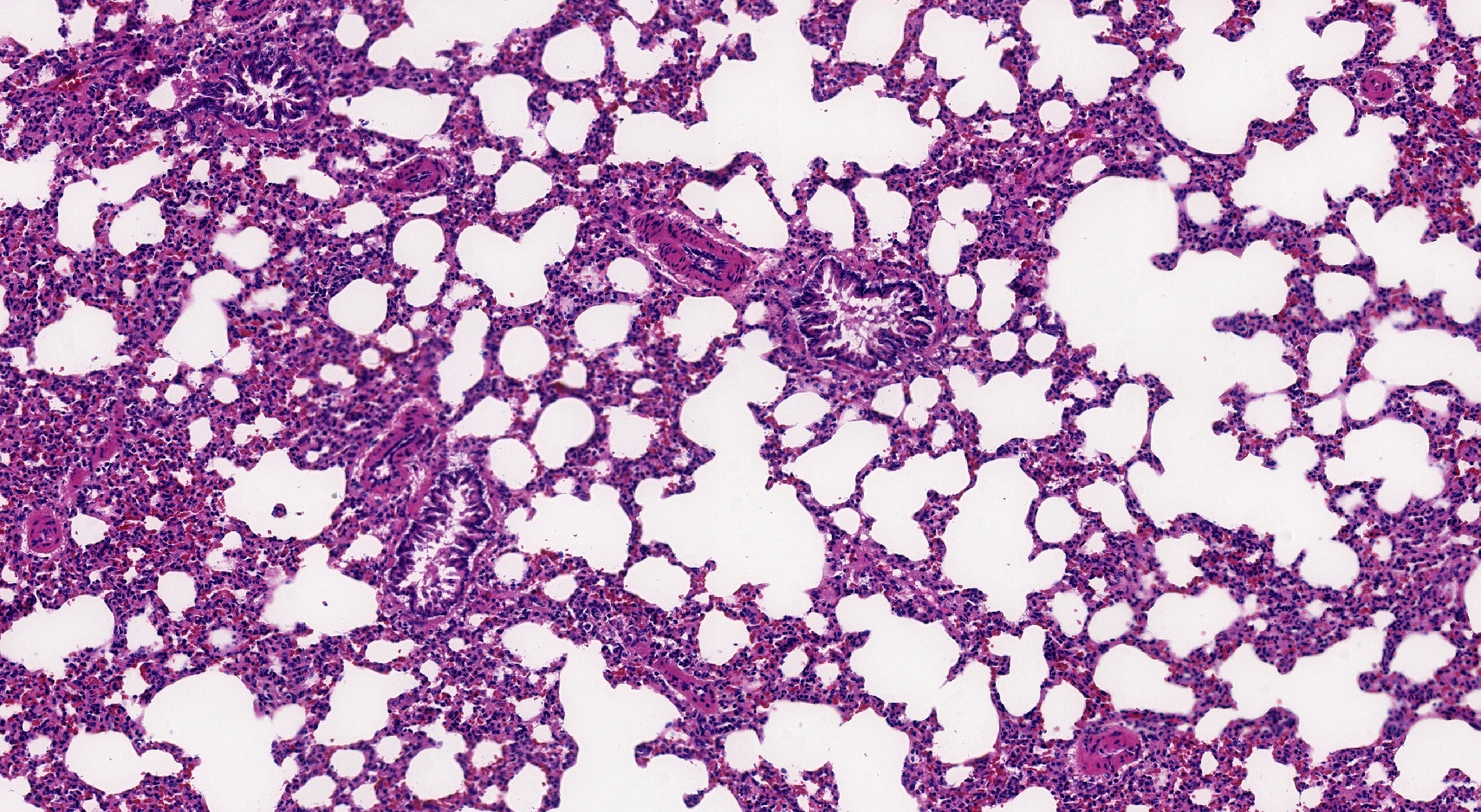

Supplement: Supplementary file 7 [file DataSheet6.zip › Figure 9 and 10/TCP-lung.png]

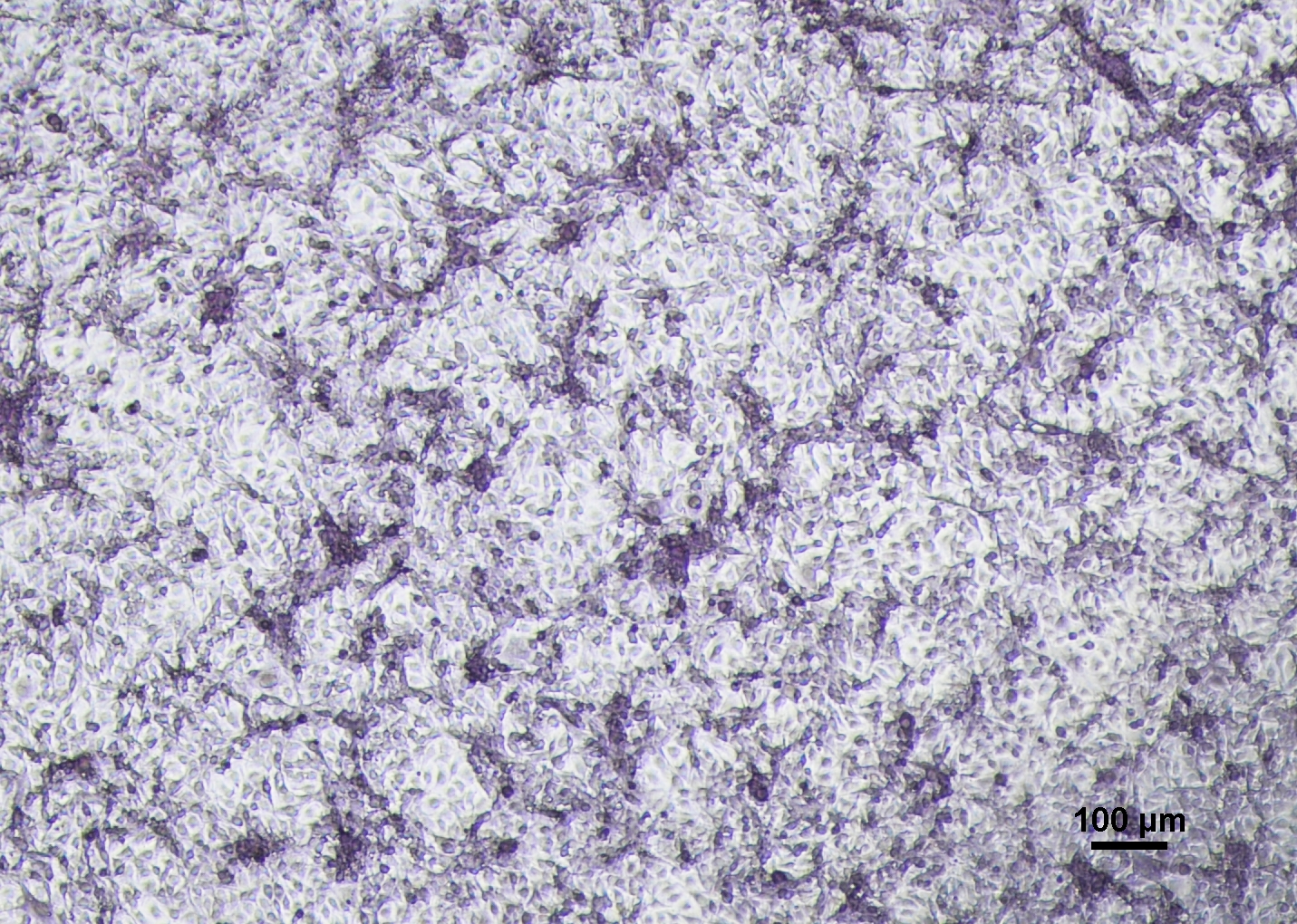

Supplement: Supplementary file 8 [file DataSheet2.zip › Figure 3 and 4/Figure 4/raw data_Figure 4 ALP staining/Day14 0MS.png]

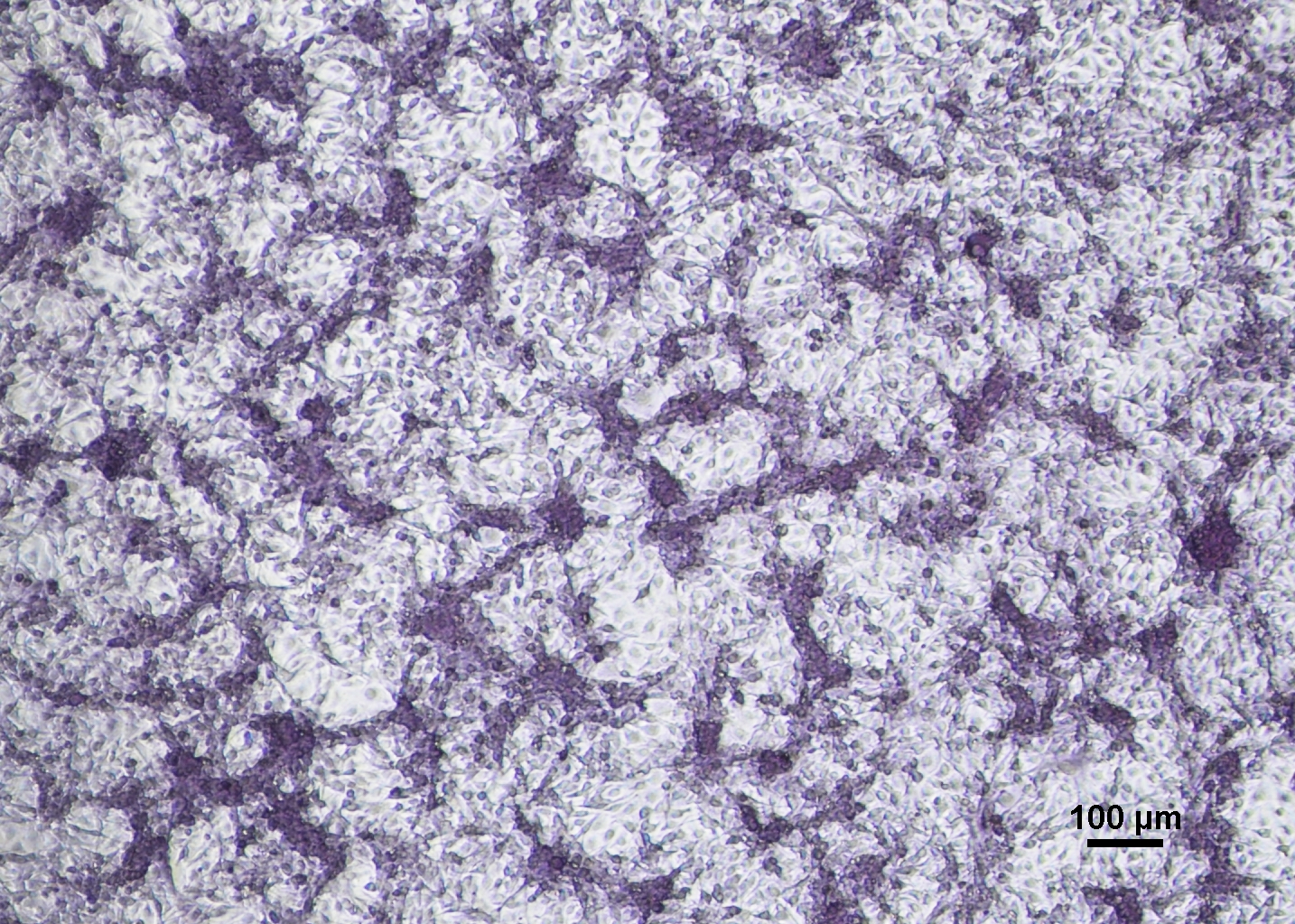

Supplement: Supplementary file 8 [file DataSheet2.zip › Figure 3 and 4/Figure 4/raw data_Figure 4 ALP staining/Day14 10MS.png]

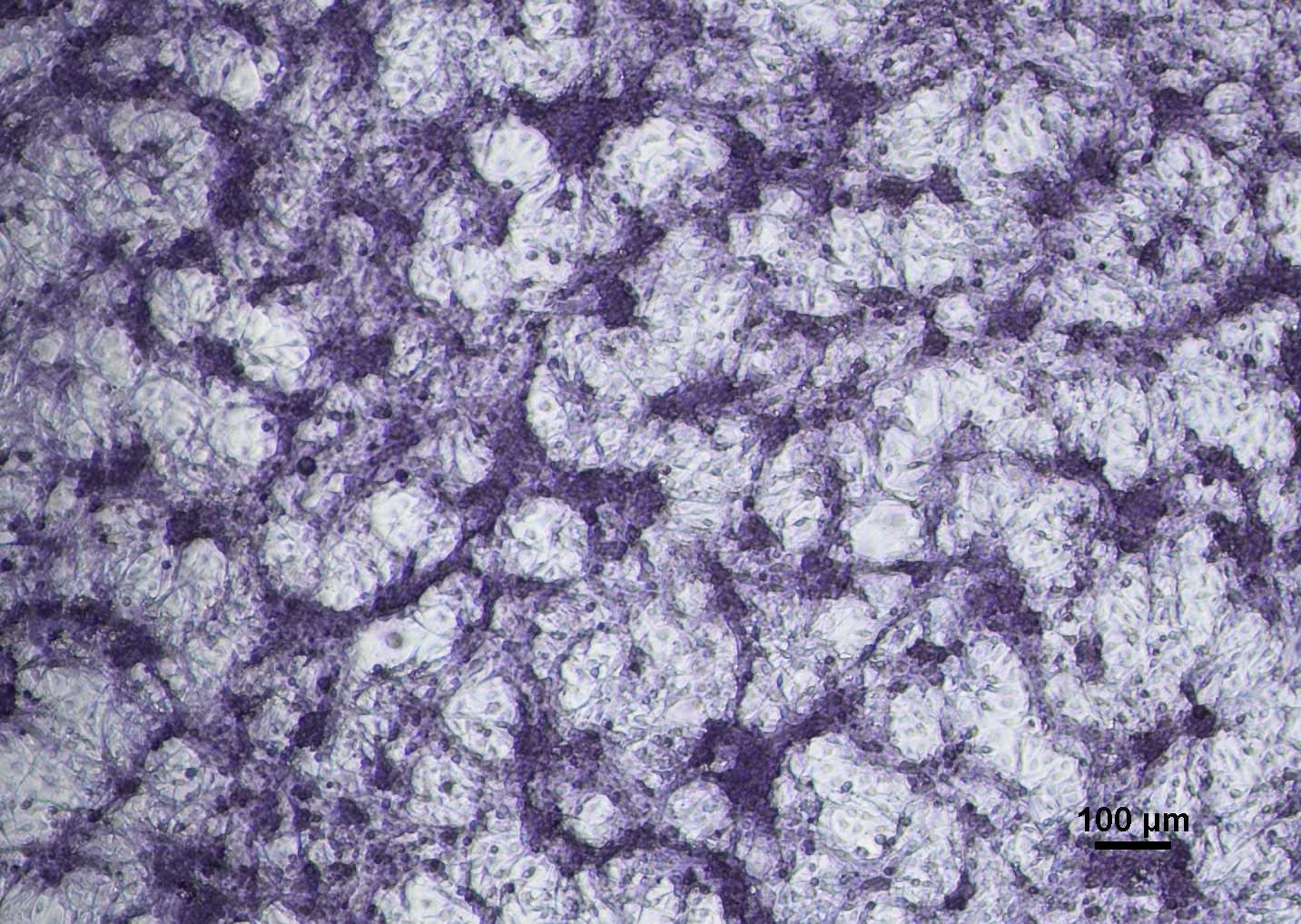

Supplement: Supplementary file 8 [file DataSheet2.zip › Figure 3 and 4/Figure 4/raw data_Figure 4 ALP staining/Day14 15MS.png]

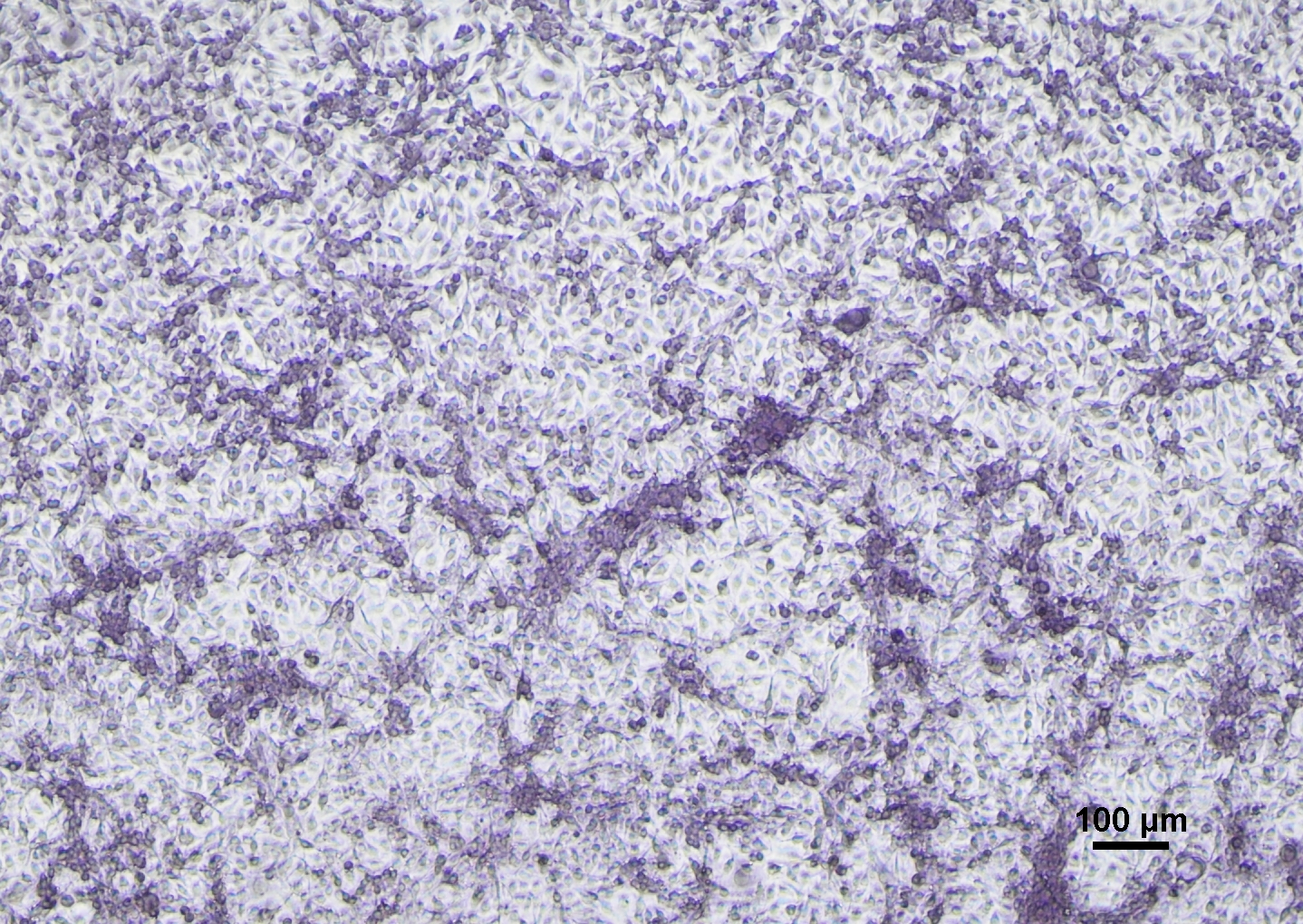

Supplement: Supplementary file 8 [file DataSheet2.zip › Figure 3 and 4/Figure 4/raw data_Figure 4 ALP staining/Day14 5MS.png]

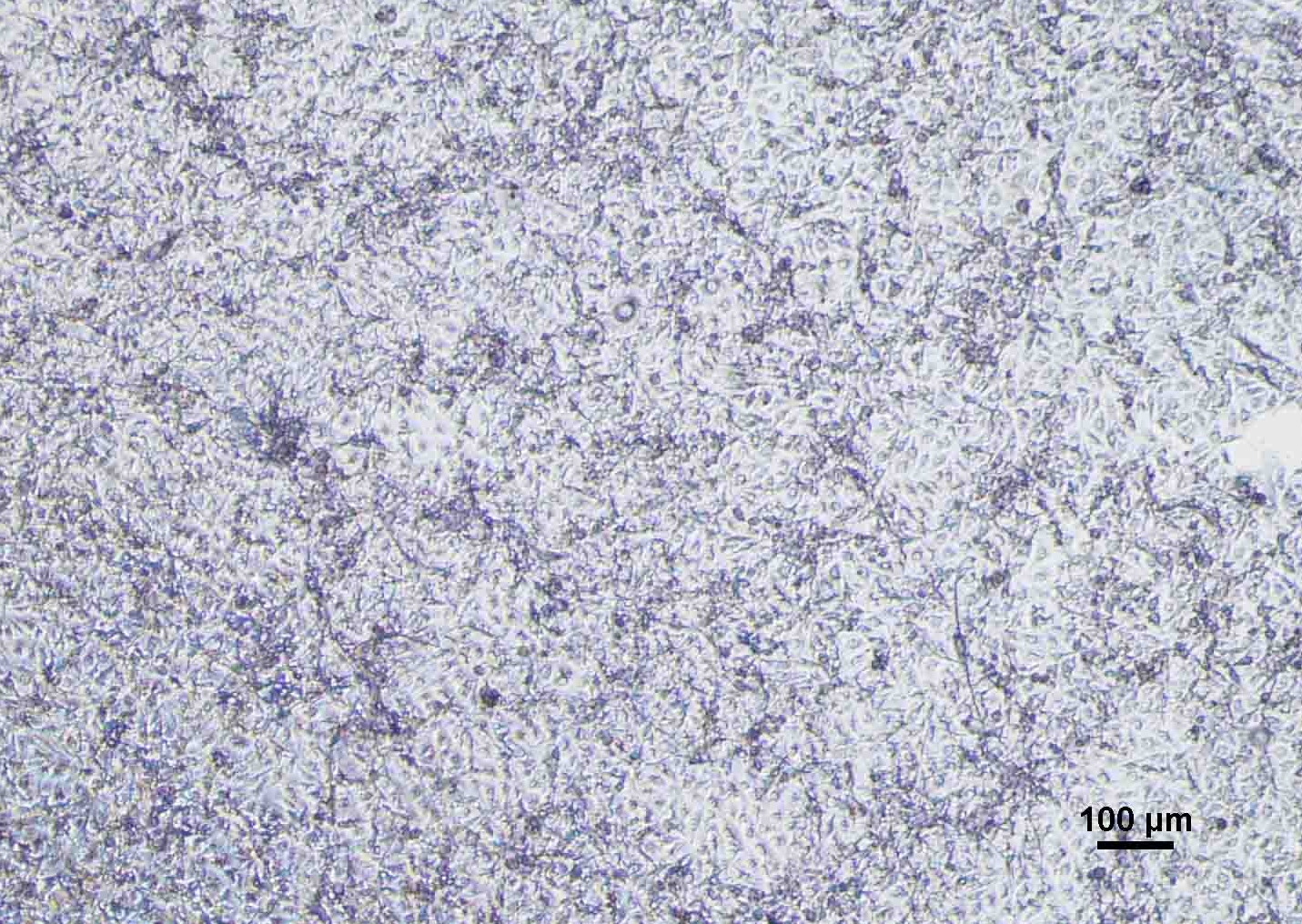

Supplement: Supplementary file 8 [file DataSheet2.zip › Figure 3 and 4/Figure 4/raw data_Figure 4 ALP staining/Day7 0MS.png]

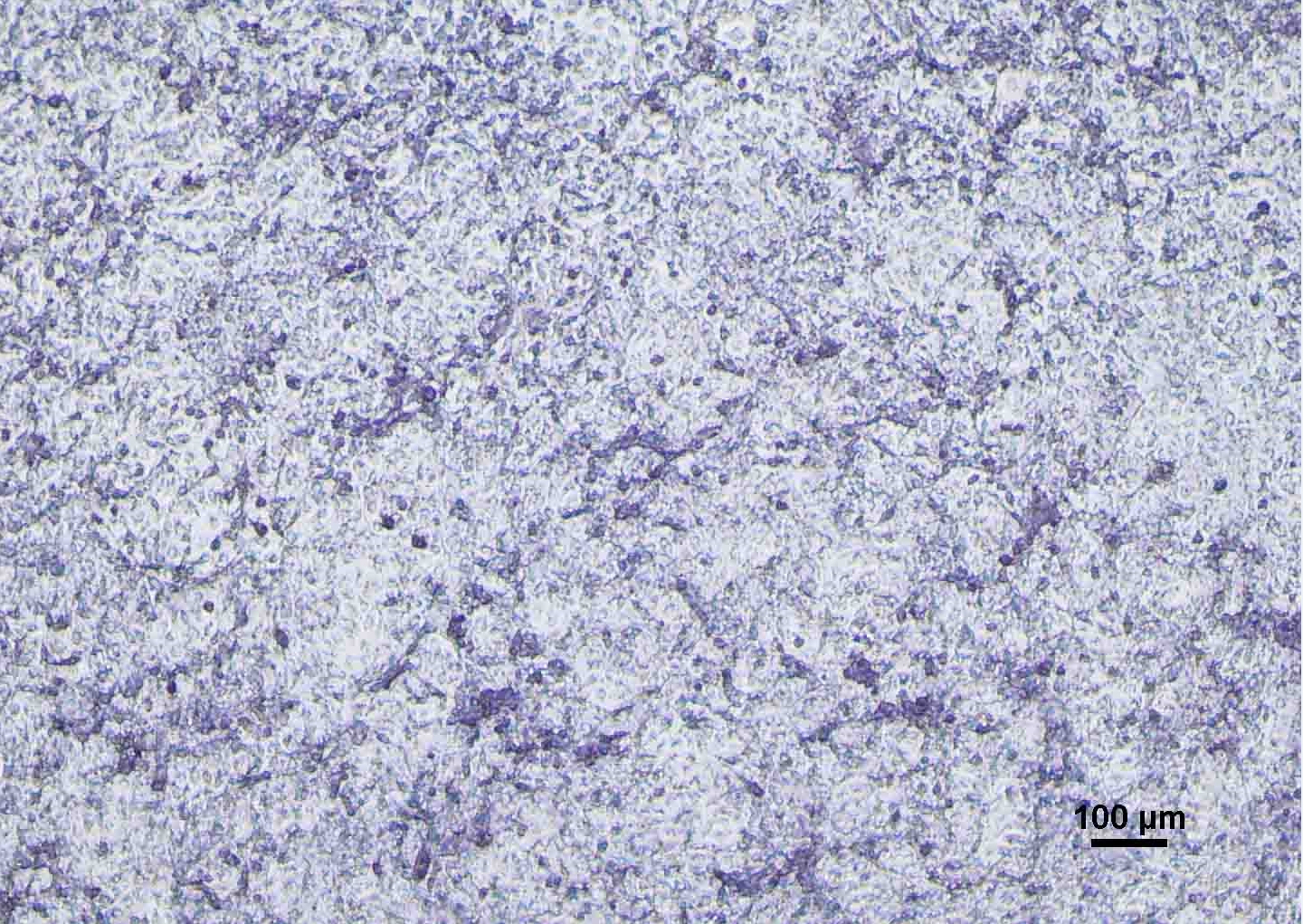

Supplement: Supplementary file 8 [file DataSheet2.zip › Figure 3 and 4/Figure 4/raw data_Figure 4 ALP staining/Day7 10MS.png]

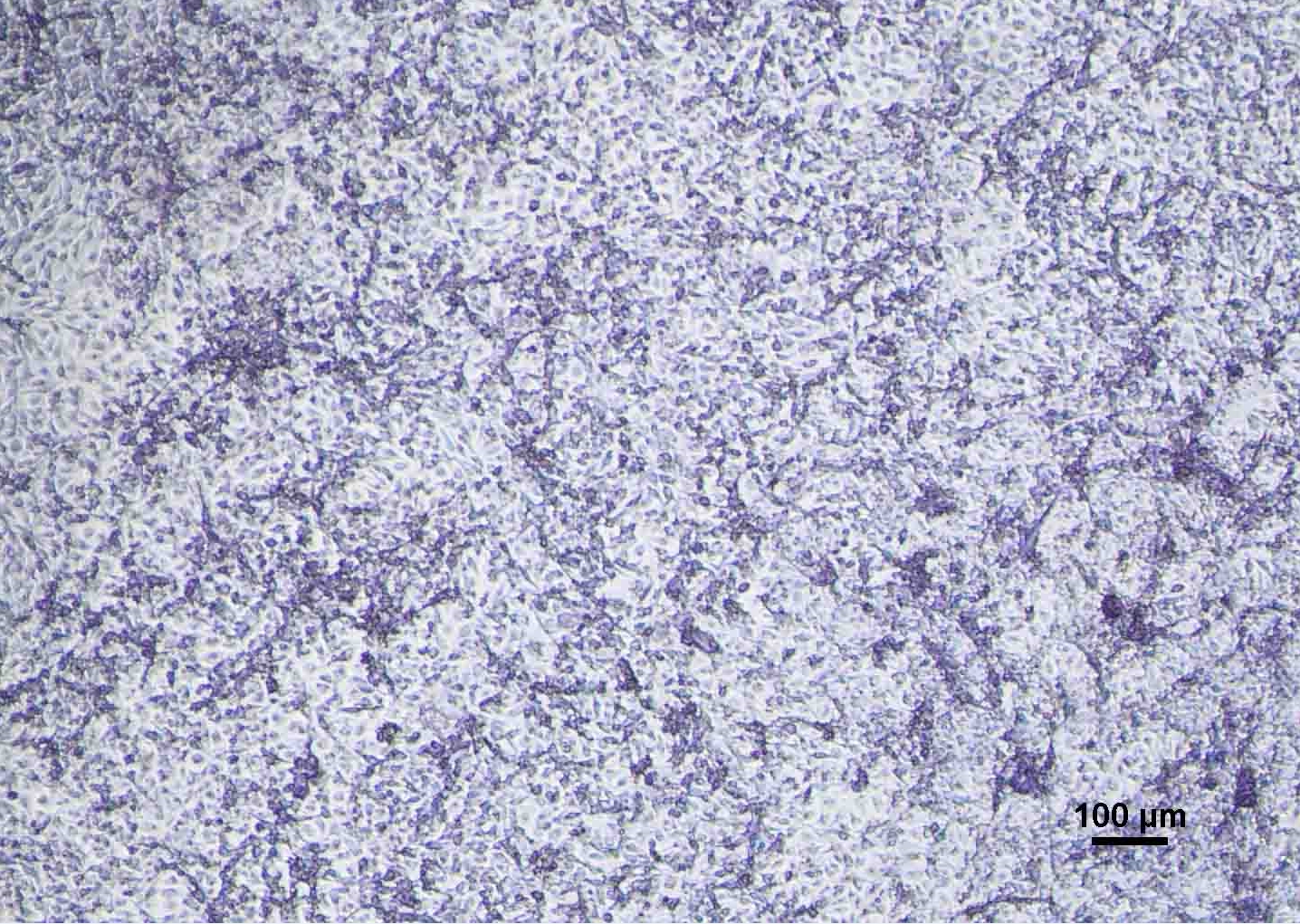

Supplement: Supplementary file 8 [file DataSheet2.zip › Figure 3 and 4/Figure 4/raw data_Figure 4 ALP staining/Day7 15MS.png]

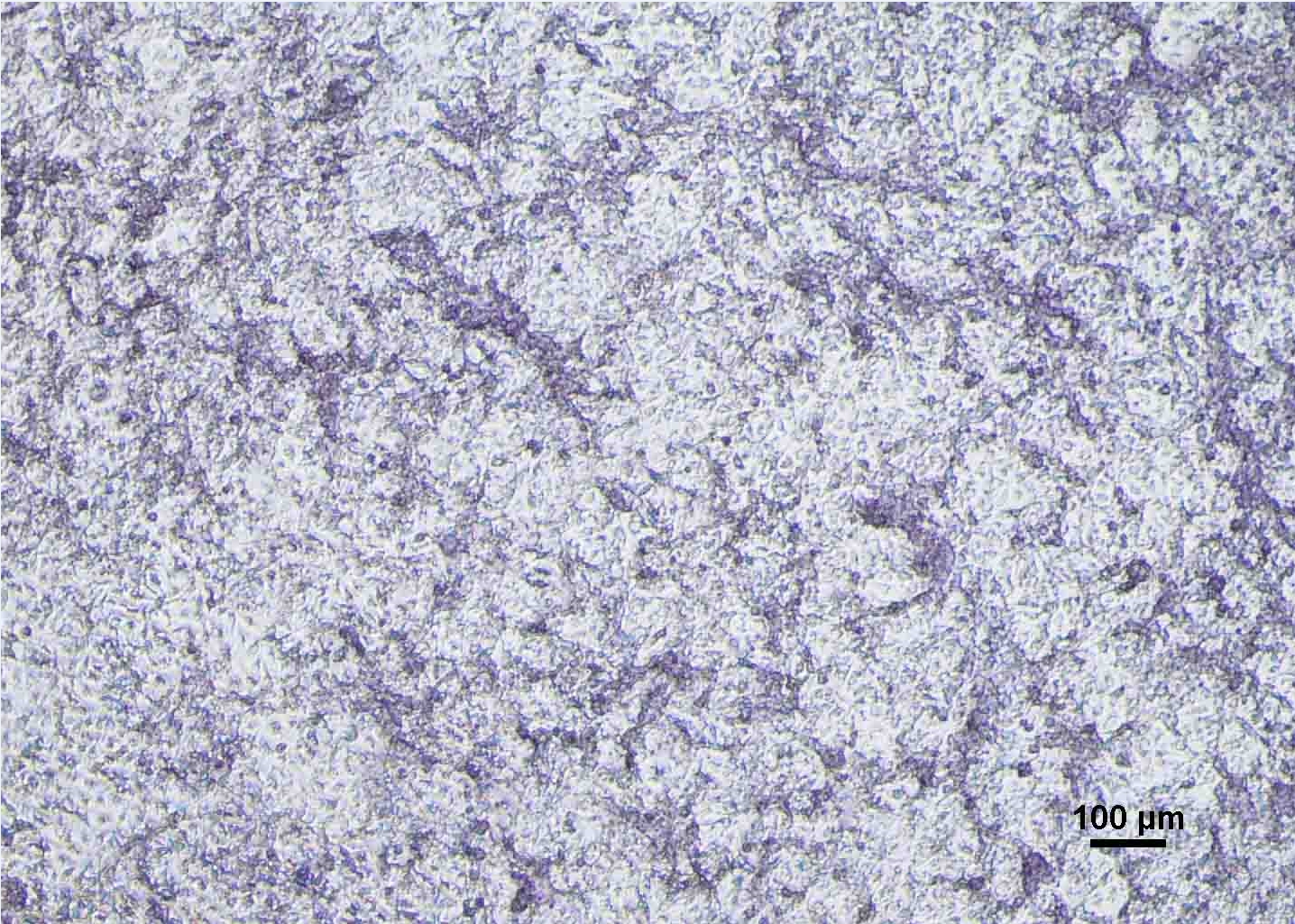

Supplement: Supplementary file 8 [file DataSheet2.zip › Figure 3 and 4/Figure 4/raw data_Figure 4 ALP staining/Day7 5MS.png]

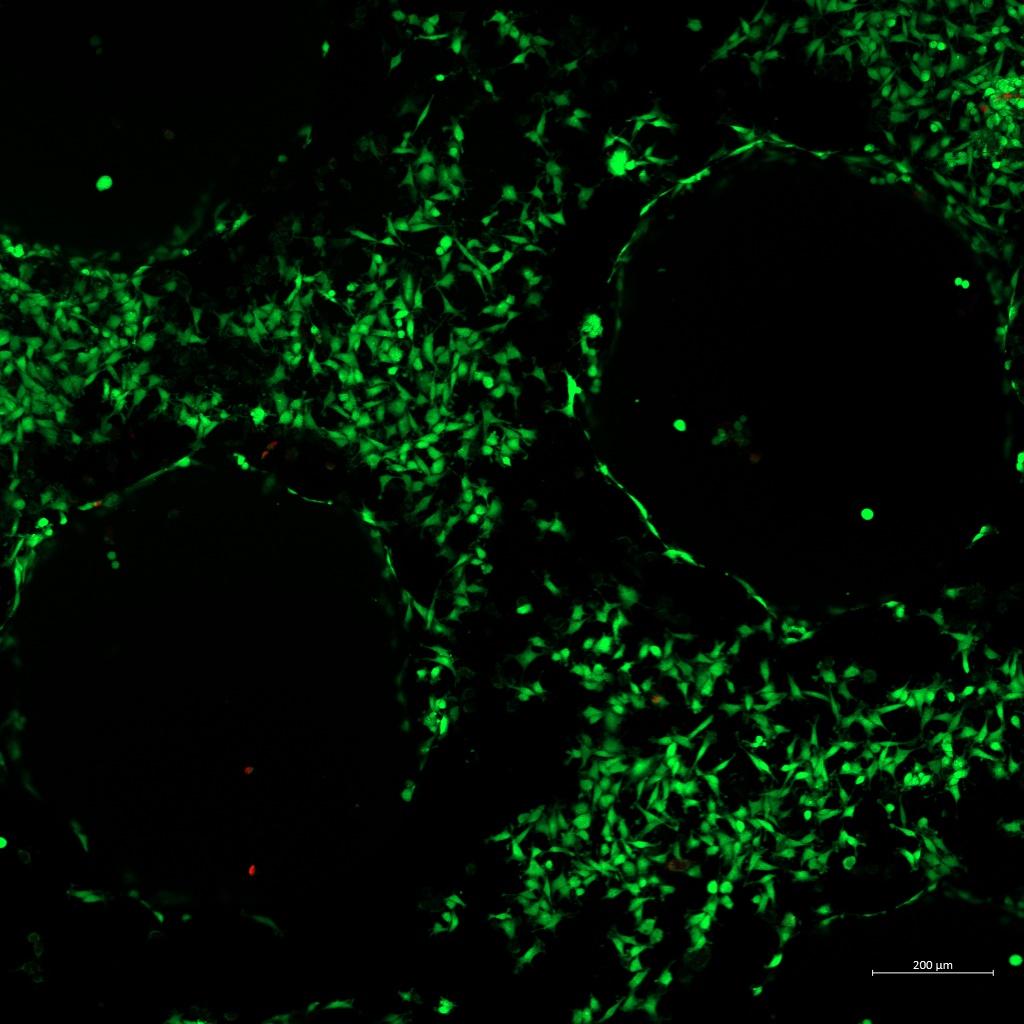

Supplement: Supplementary file 8 [file DataSheet2.zip › Figure 3 and 4/figure 3a/0MS.jpg]

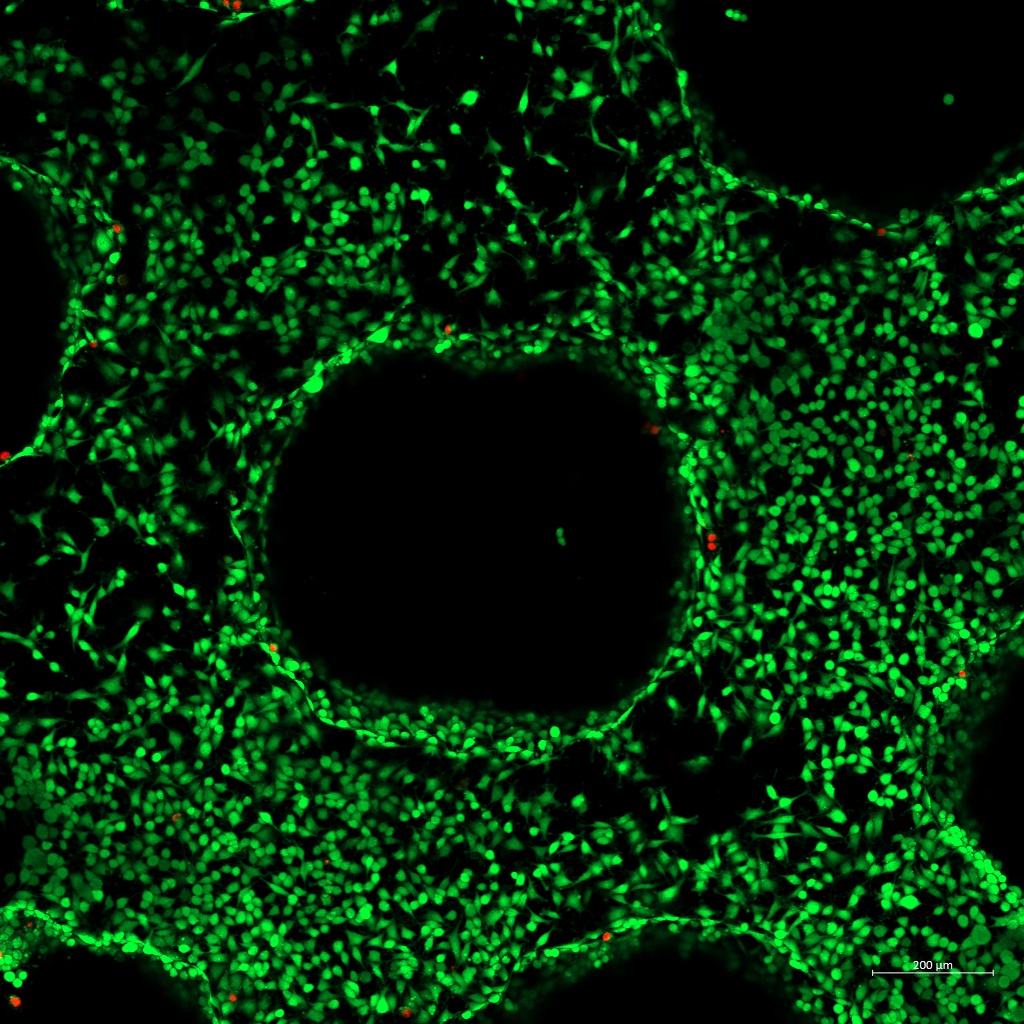

Supplement: Supplementary file 8 [file DataSheet2.zip › Figure 3 and 4/figure 3a/10MS.jpg]

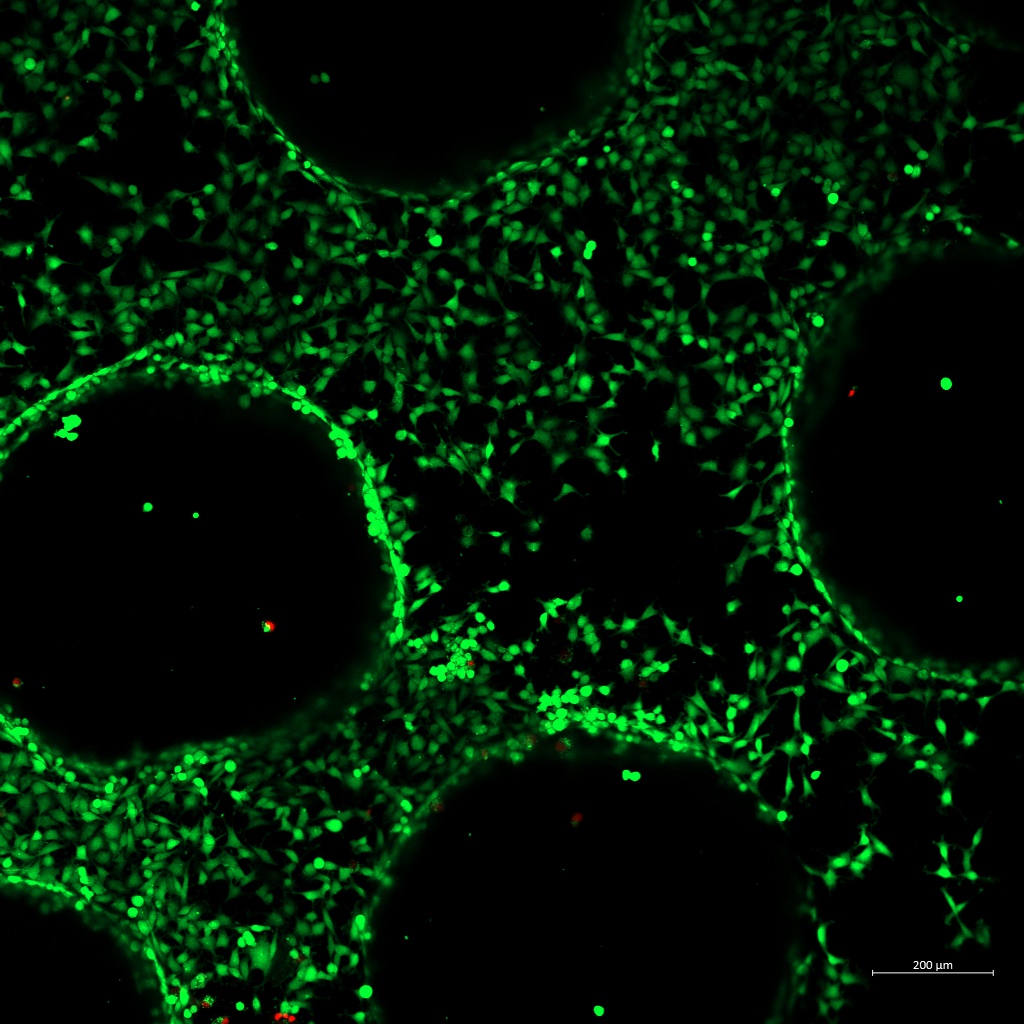

Supplement: Supplementary file 8 [file DataSheet2.zip › Figure 3 and 4/figure 3a/15MS.jpg]

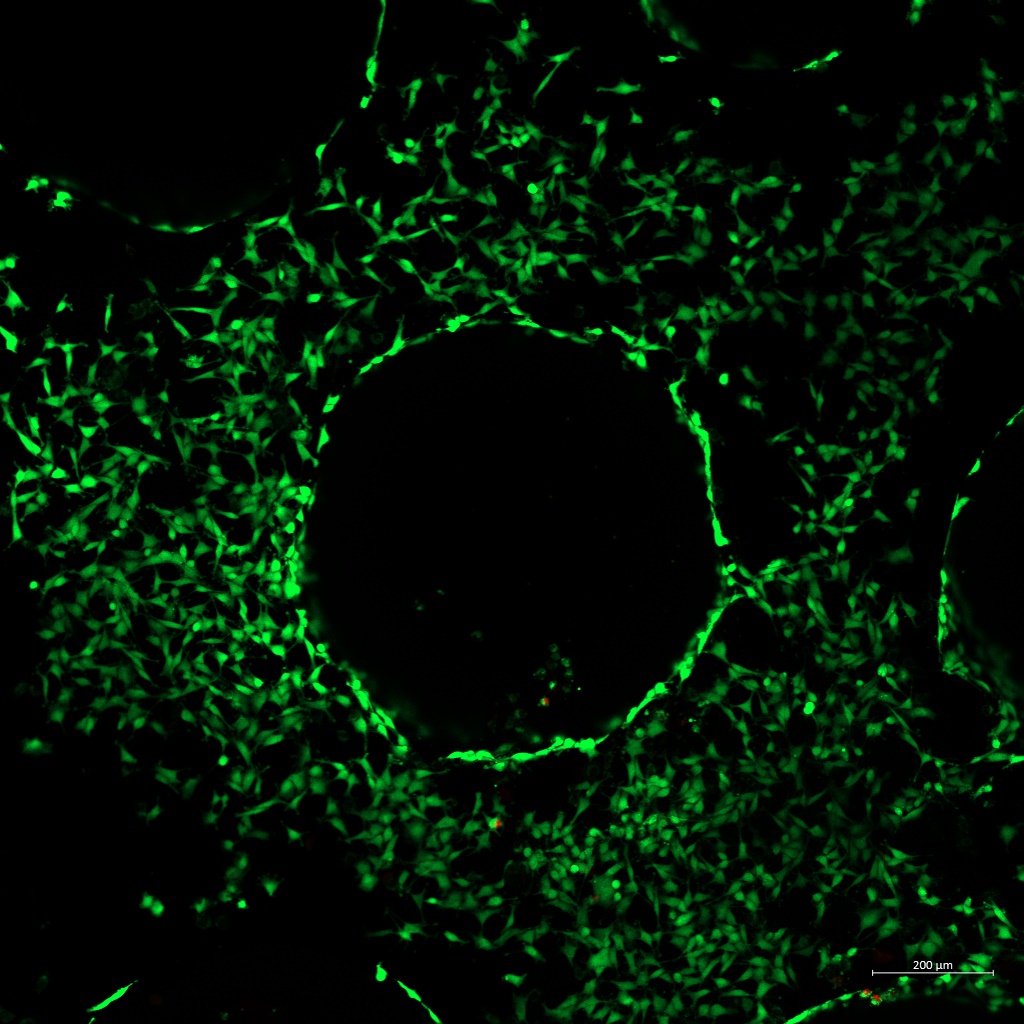

Supplement: Supplementary file 8 [file DataSheet2.zip › Figure 3 and 4/figure 3a/5MS.jpg]

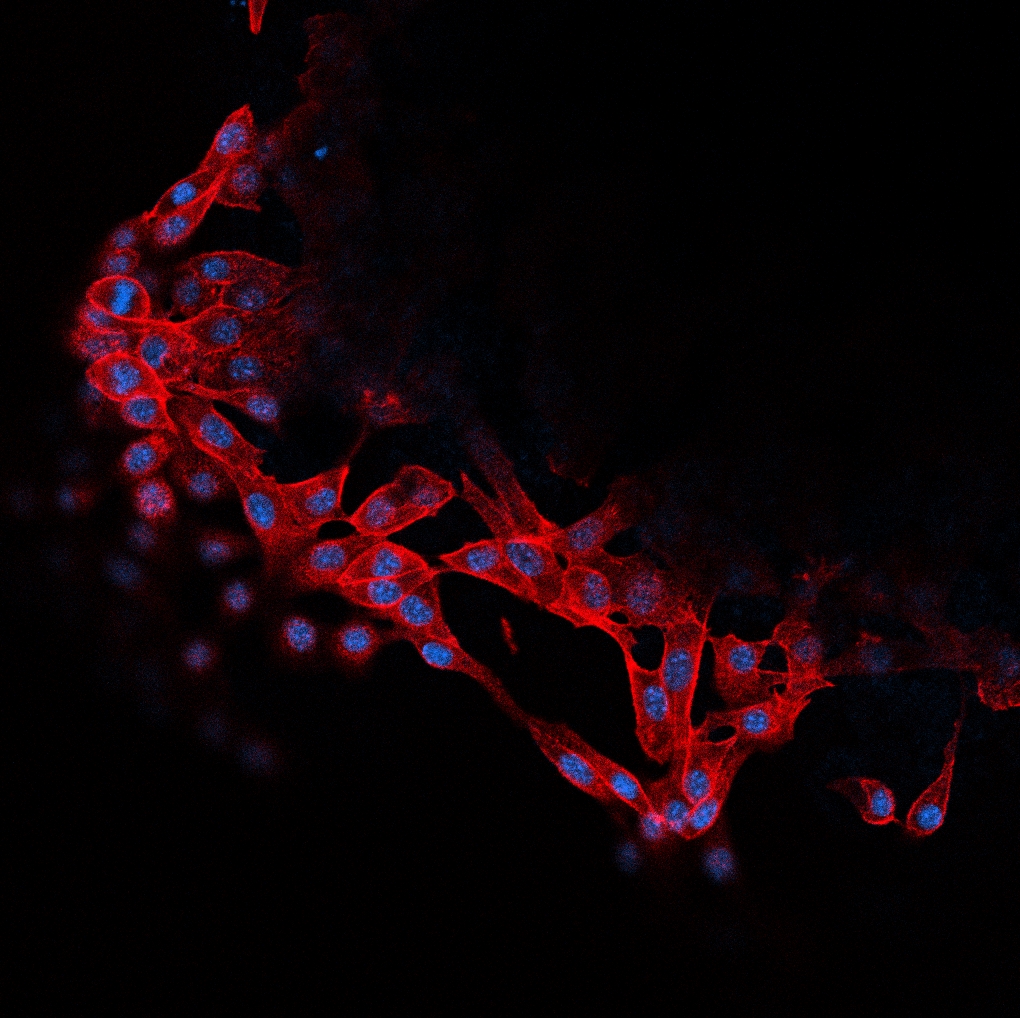

Supplement: Supplementary file 8 [file DataSheet2.zip › Figure 3 and 4/figure 3b/0MS.png]

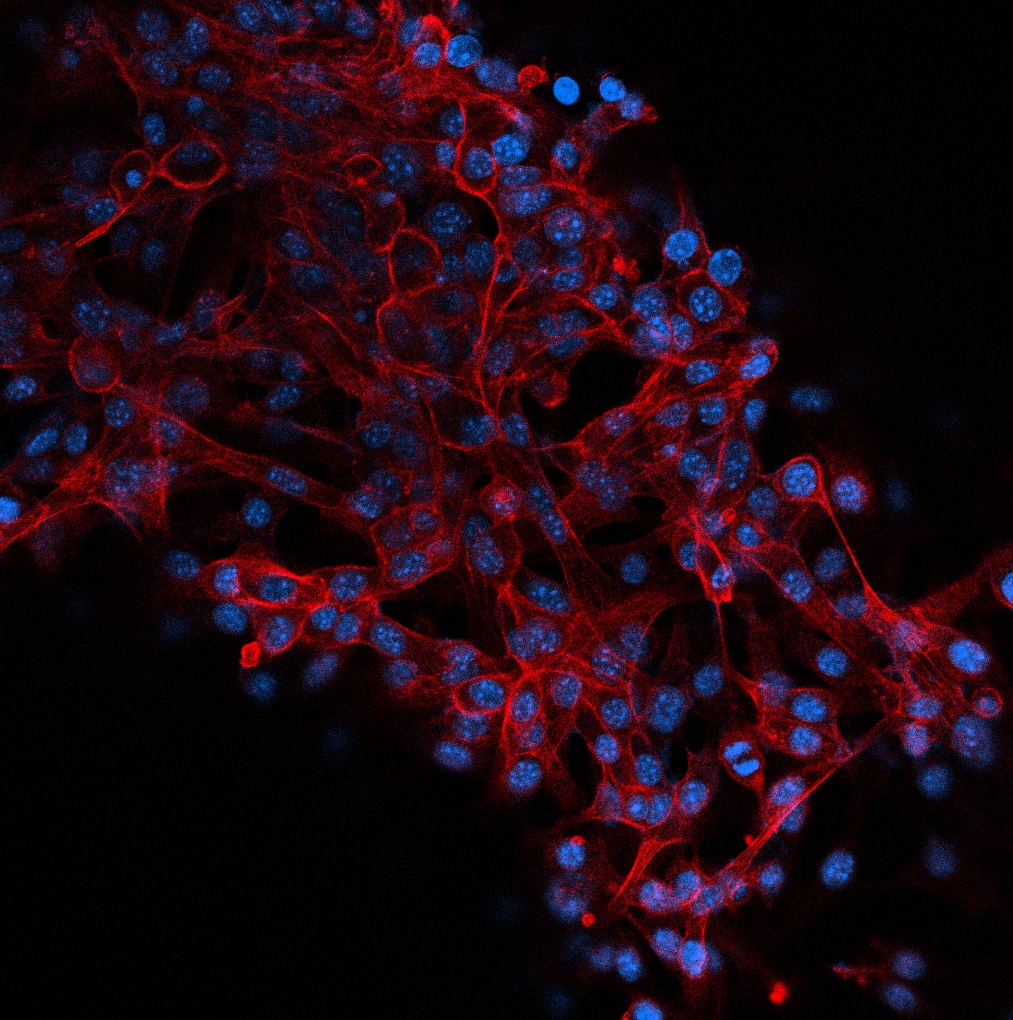

Supplement: Supplementary file 8 [file DataSheet2.zip › Figure 3 and 4/figure 3b/10MS.png]

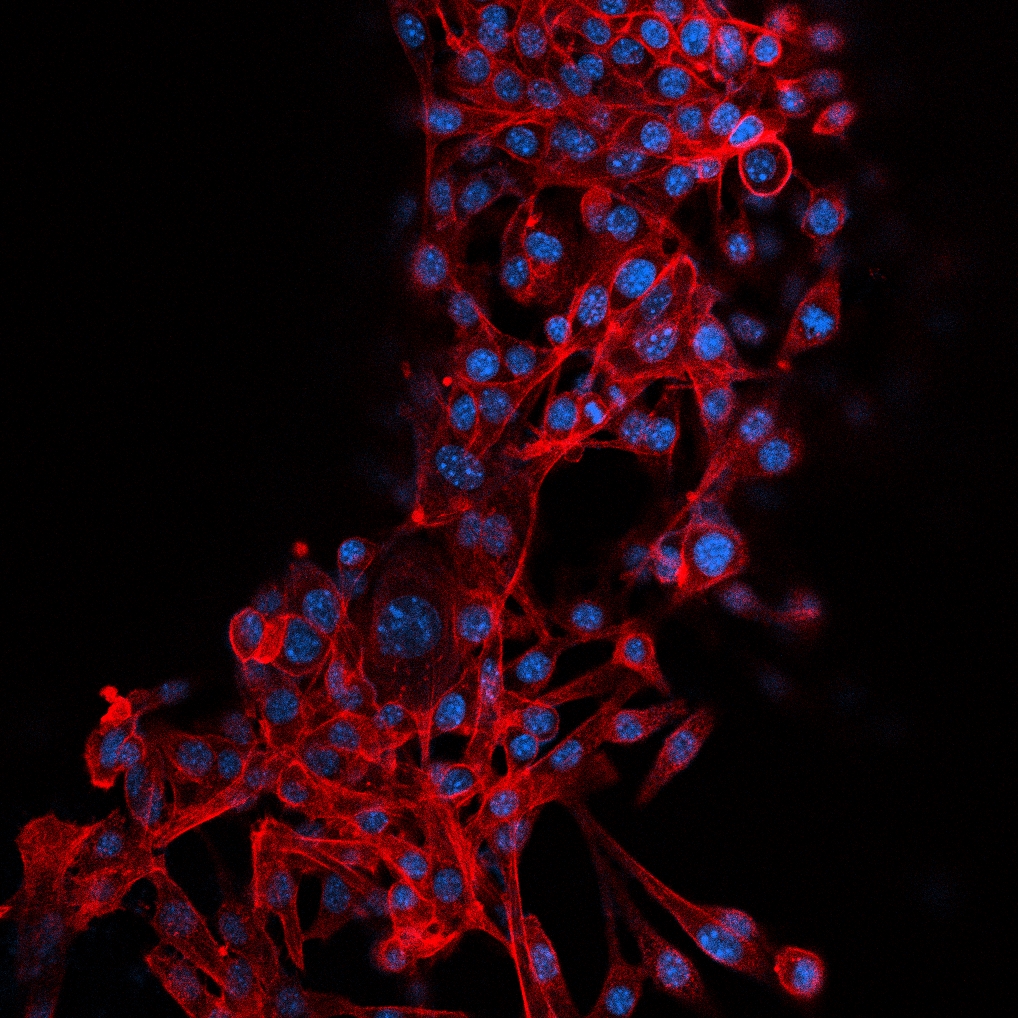

Supplement: Supplementary file 8 [file DataSheet2.zip › Figure 3 and 4/figure 3b/15MS.png]

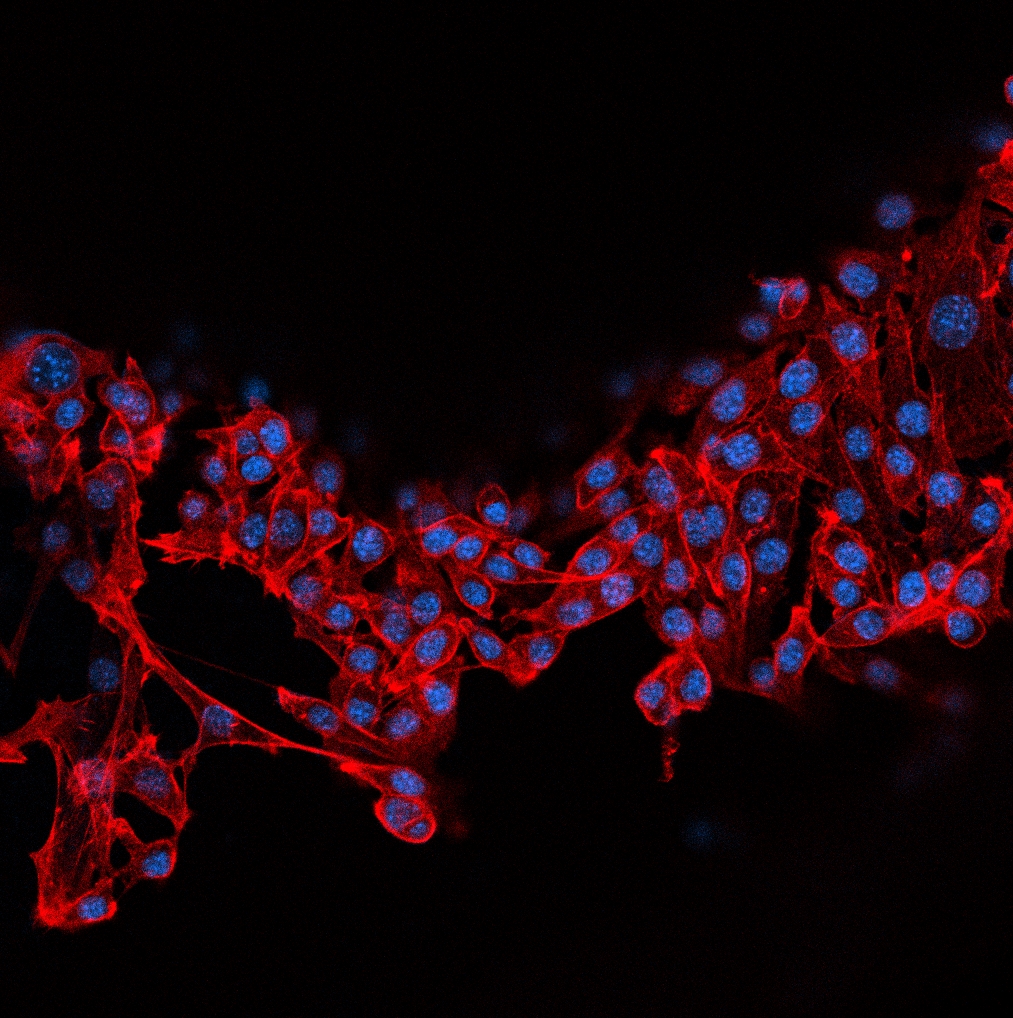

Supplement: Supplementary file 8 [file DataSheet2.zip › Figure 3 and 4/figure 3b/5MS.png]

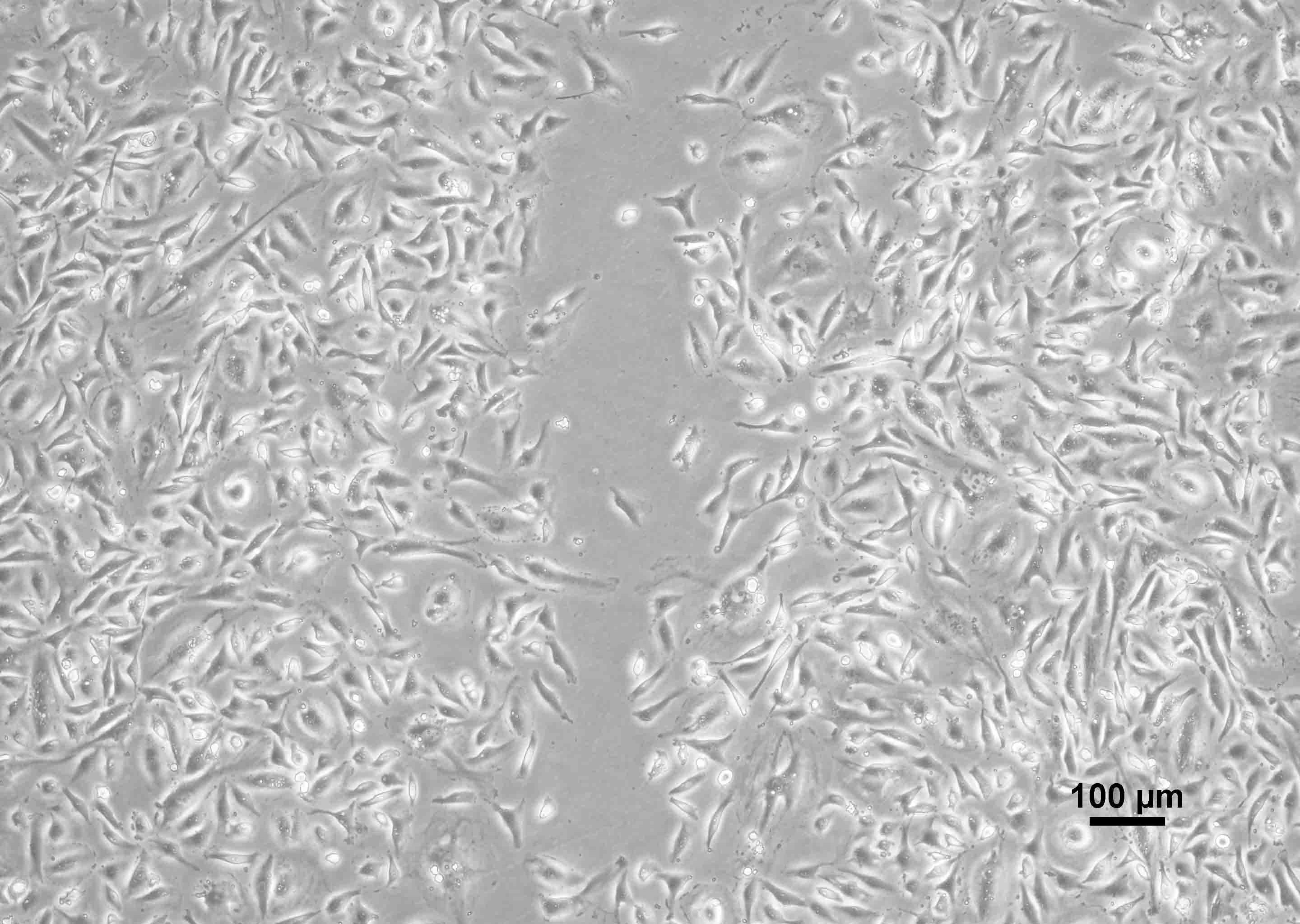

Supplement: Supplementary file 8 [file DataSheet2.zip › Figure 3 and 4/figure 3d/0MS 48h Image.tif]

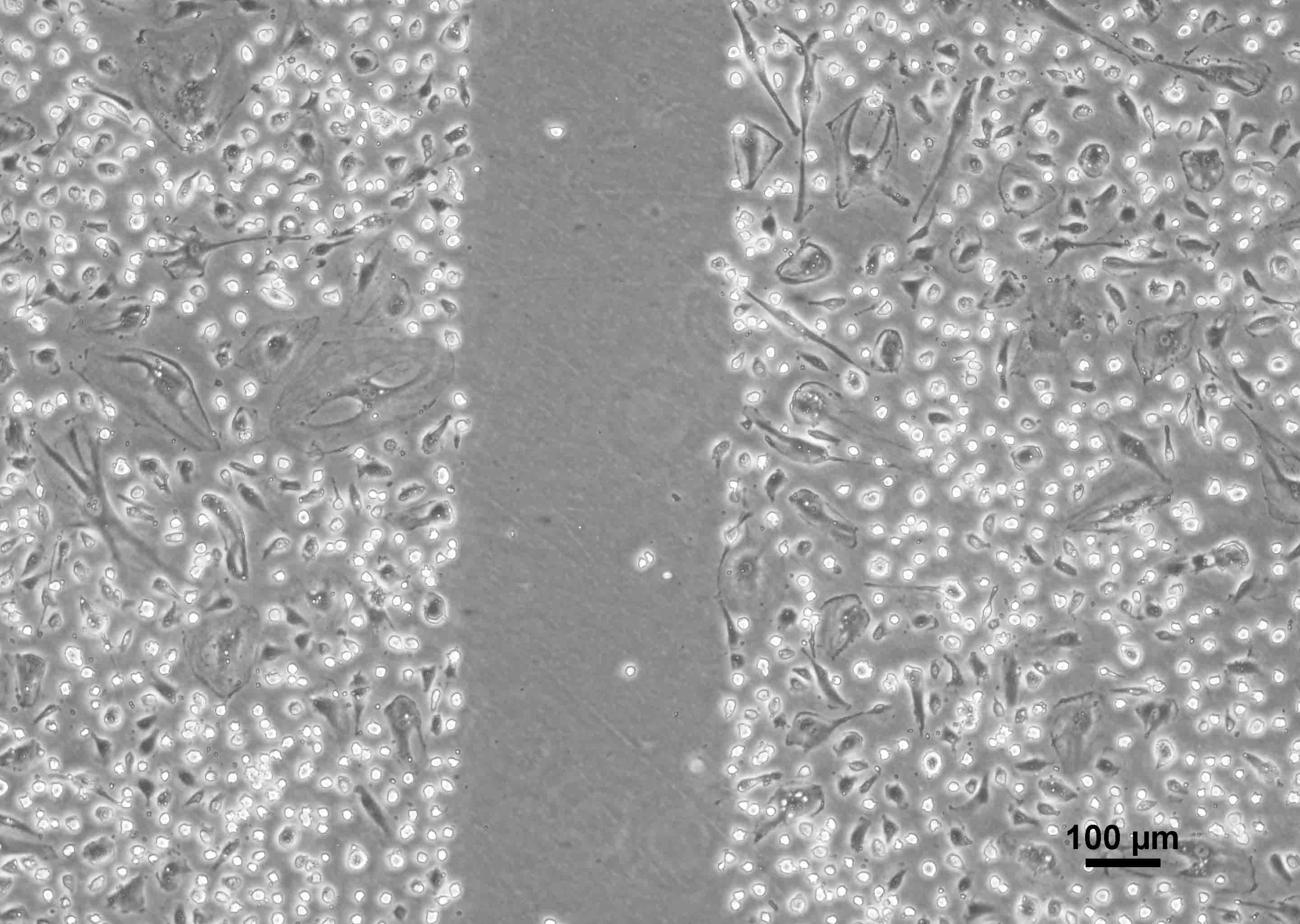

Supplement: Supplementary file 8 [file DataSheet2.zip › Figure 3 and 4/figure 3d/0ms 0h Image.tif]

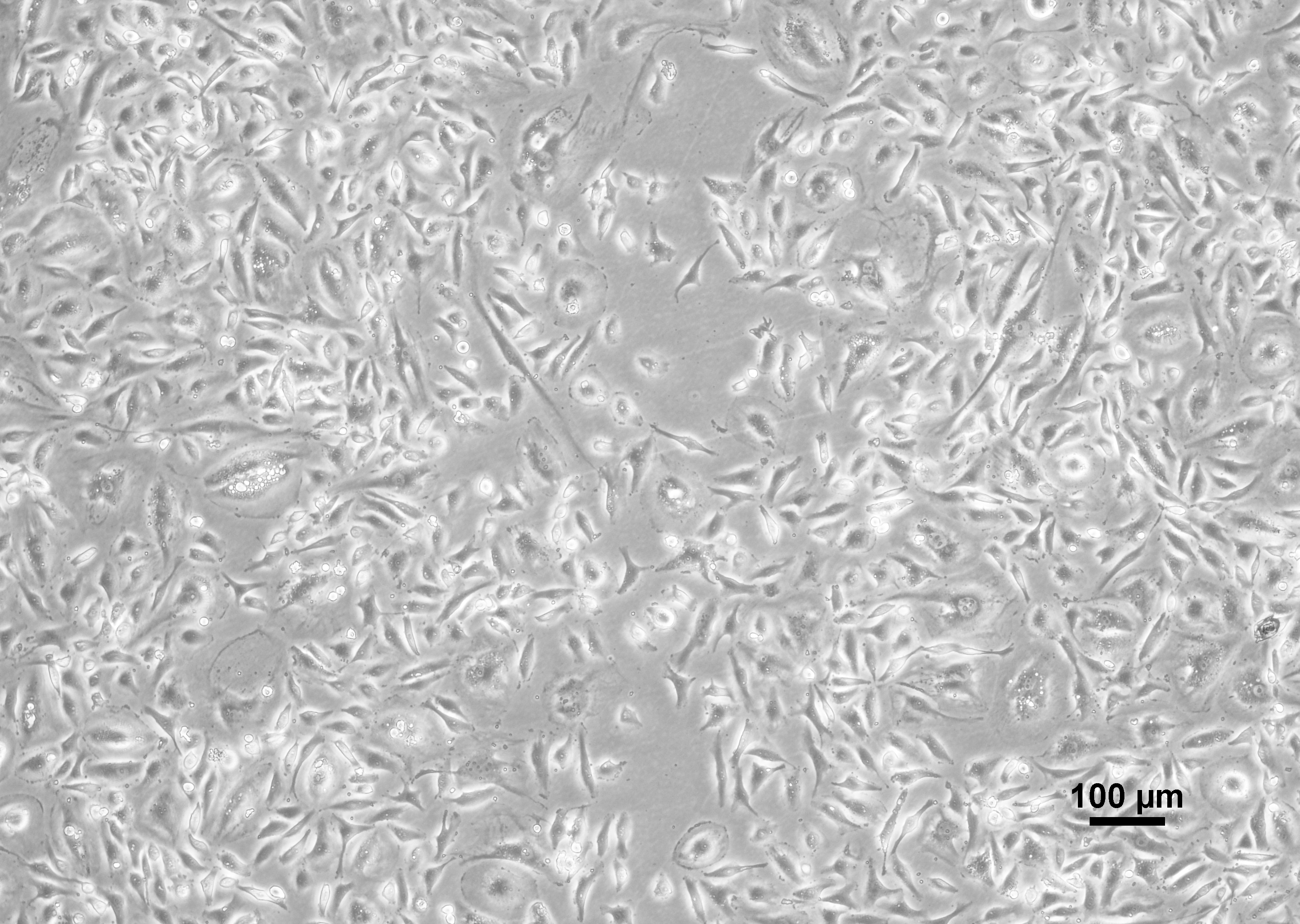

Supplement: Supplementary file 8 [file DataSheet2.zip › Figure 3 and 4/figure 3d/10MS 48h Image.tif]

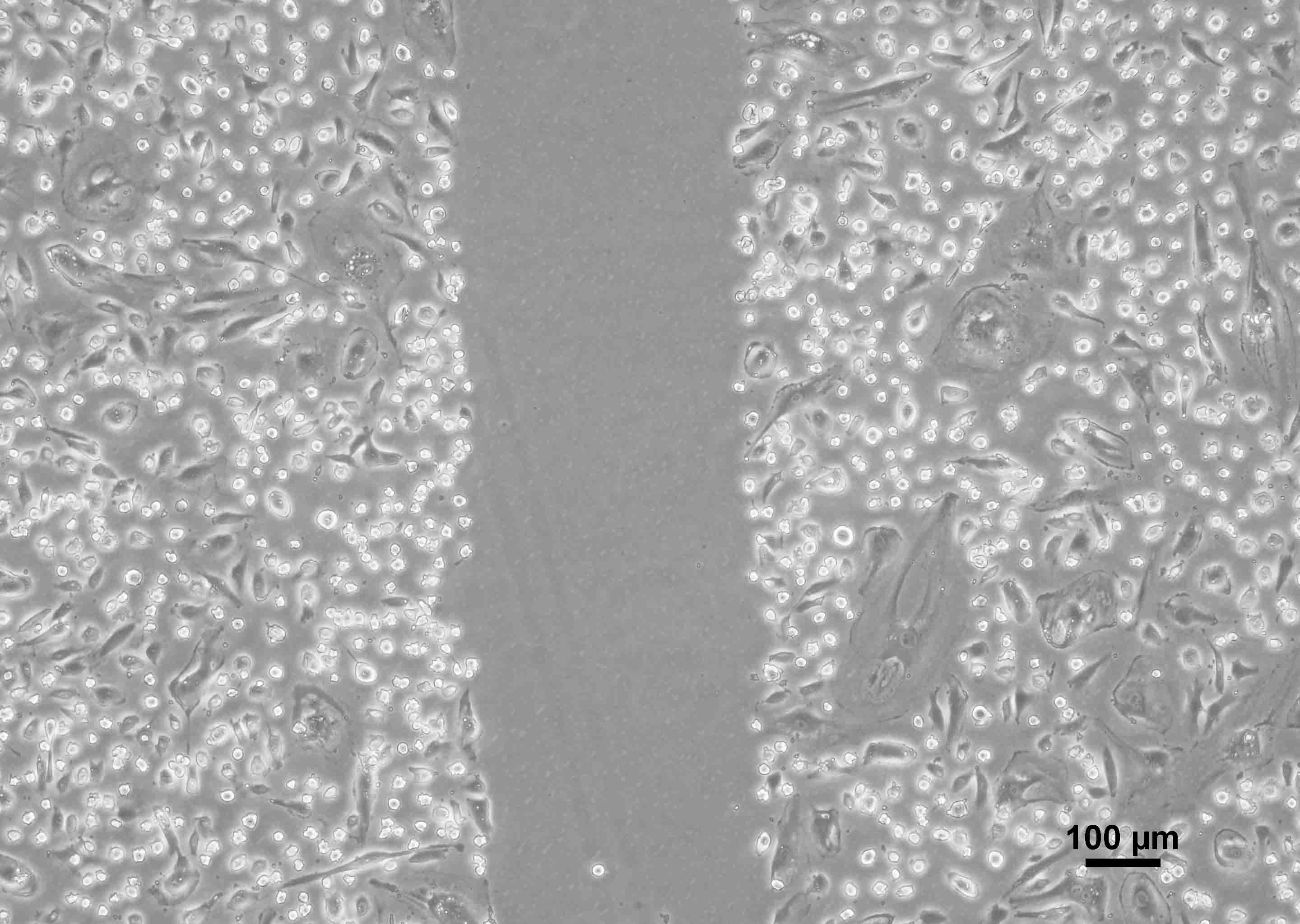

Supplement: Supplementary file 8 [file DataSheet2.zip › Figure 3 and 4/figure 3d/10ms 0h Image.tif]

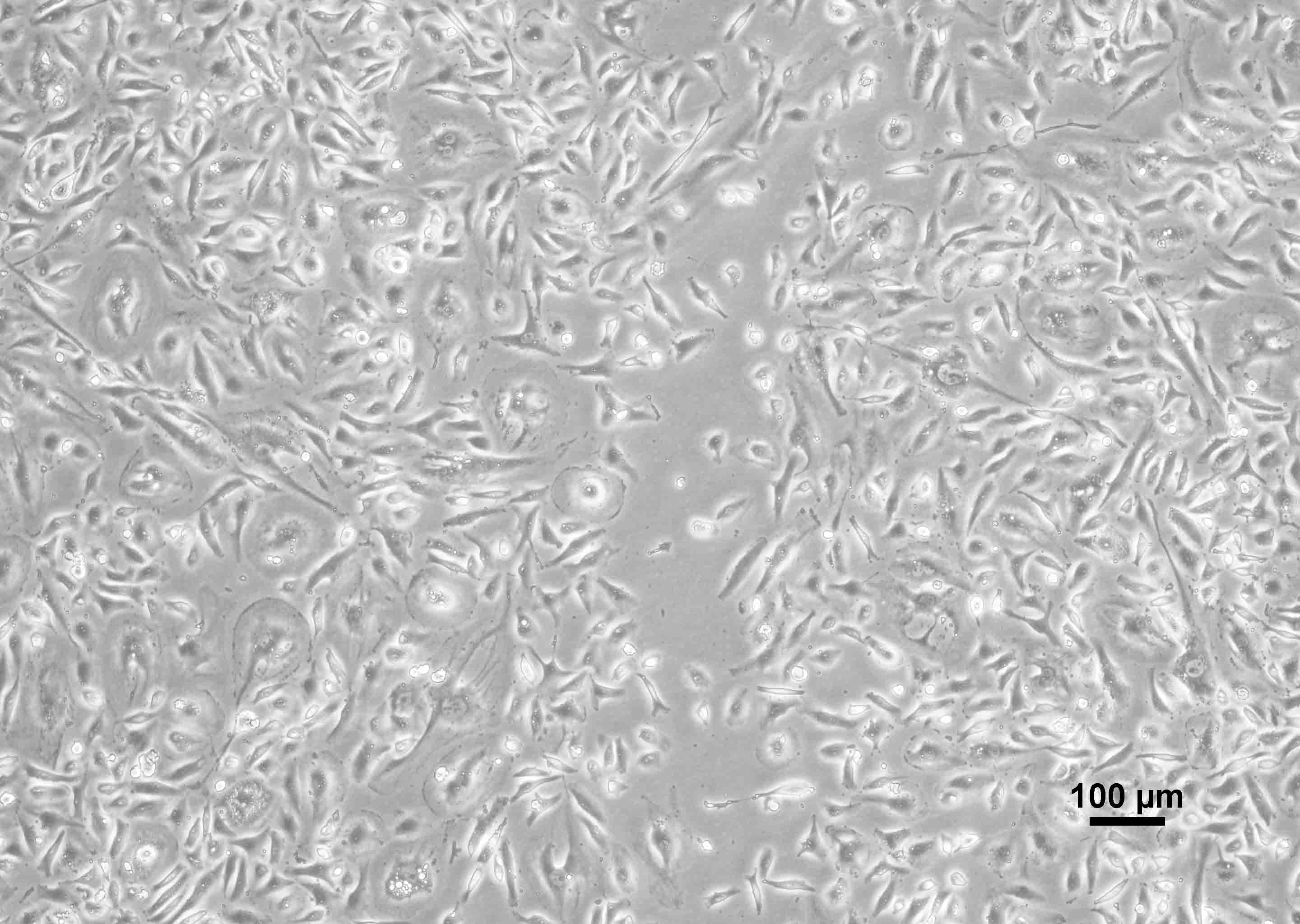

Supplement: Supplementary file 8 [file DataSheet2.zip › Figure 3 and 4/figure 3d/15MS 48h Image.tif]

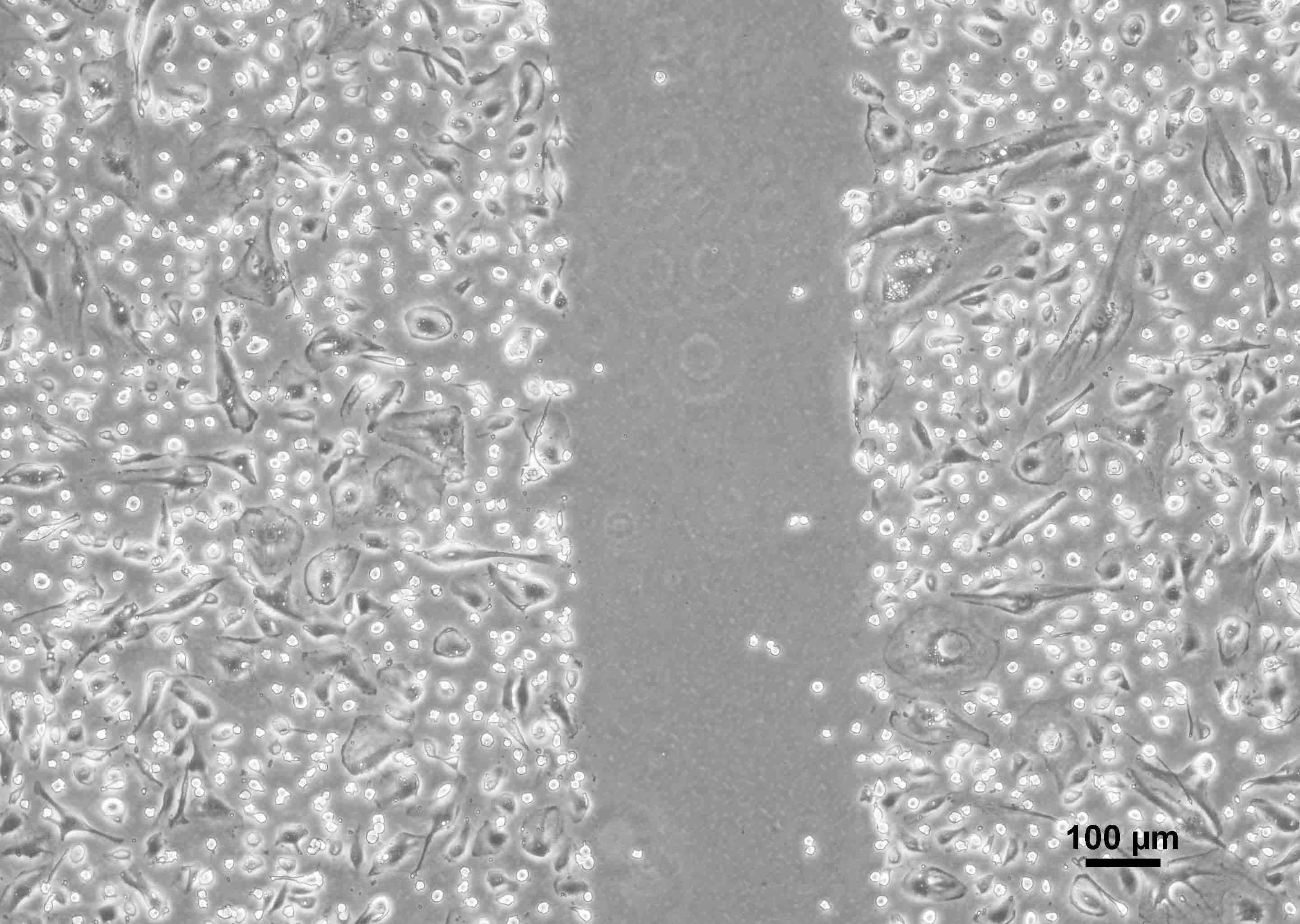

Supplement: Supplementary file 8 [file DataSheet2.zip › Figure 3 and 4/figure 3d/15ms 0h Image.tif]

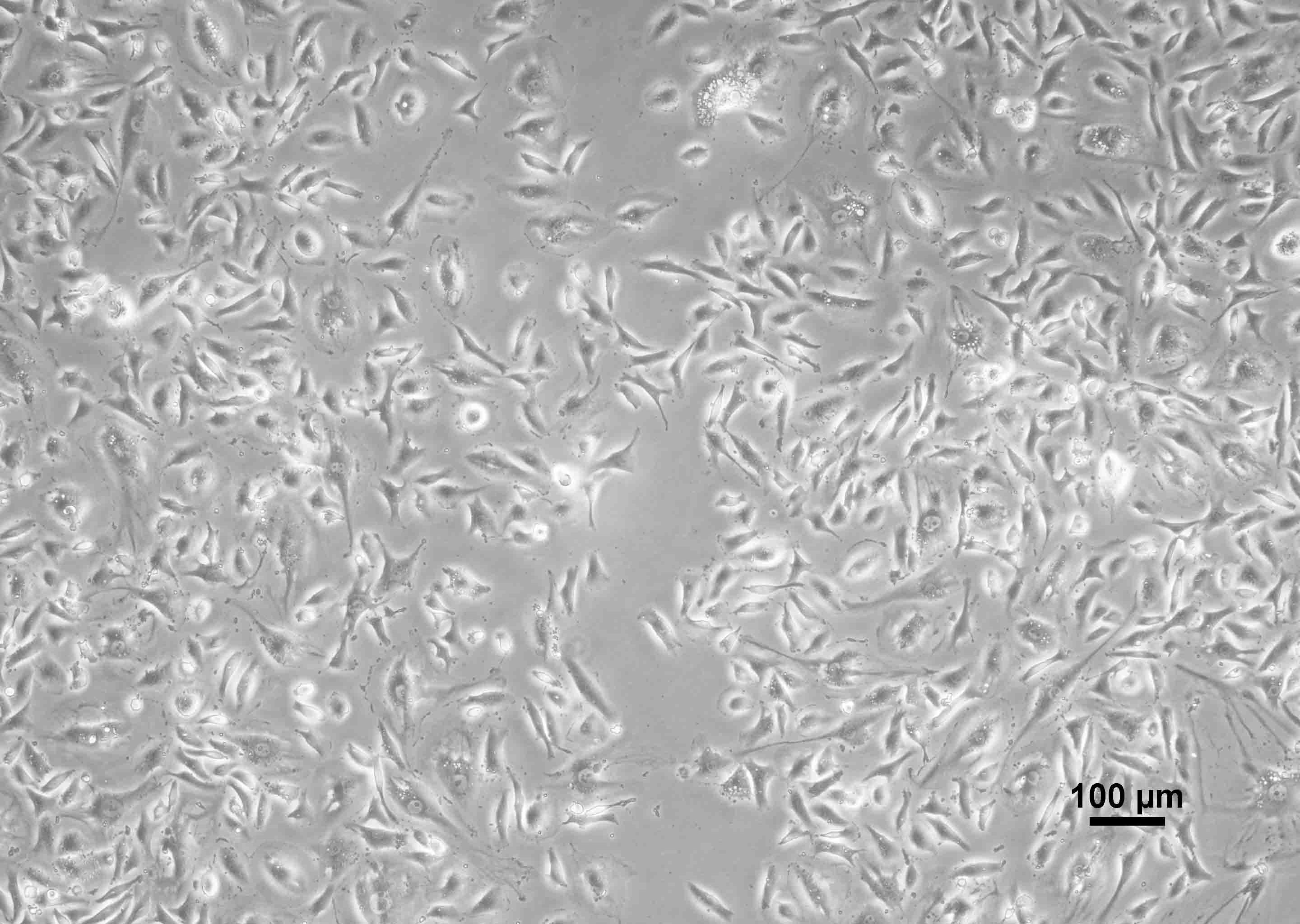

Supplement: Supplementary file 8 [file DataSheet2.zip › Figure 3 and 4/figure 3d/5MS 48h Image.tif]

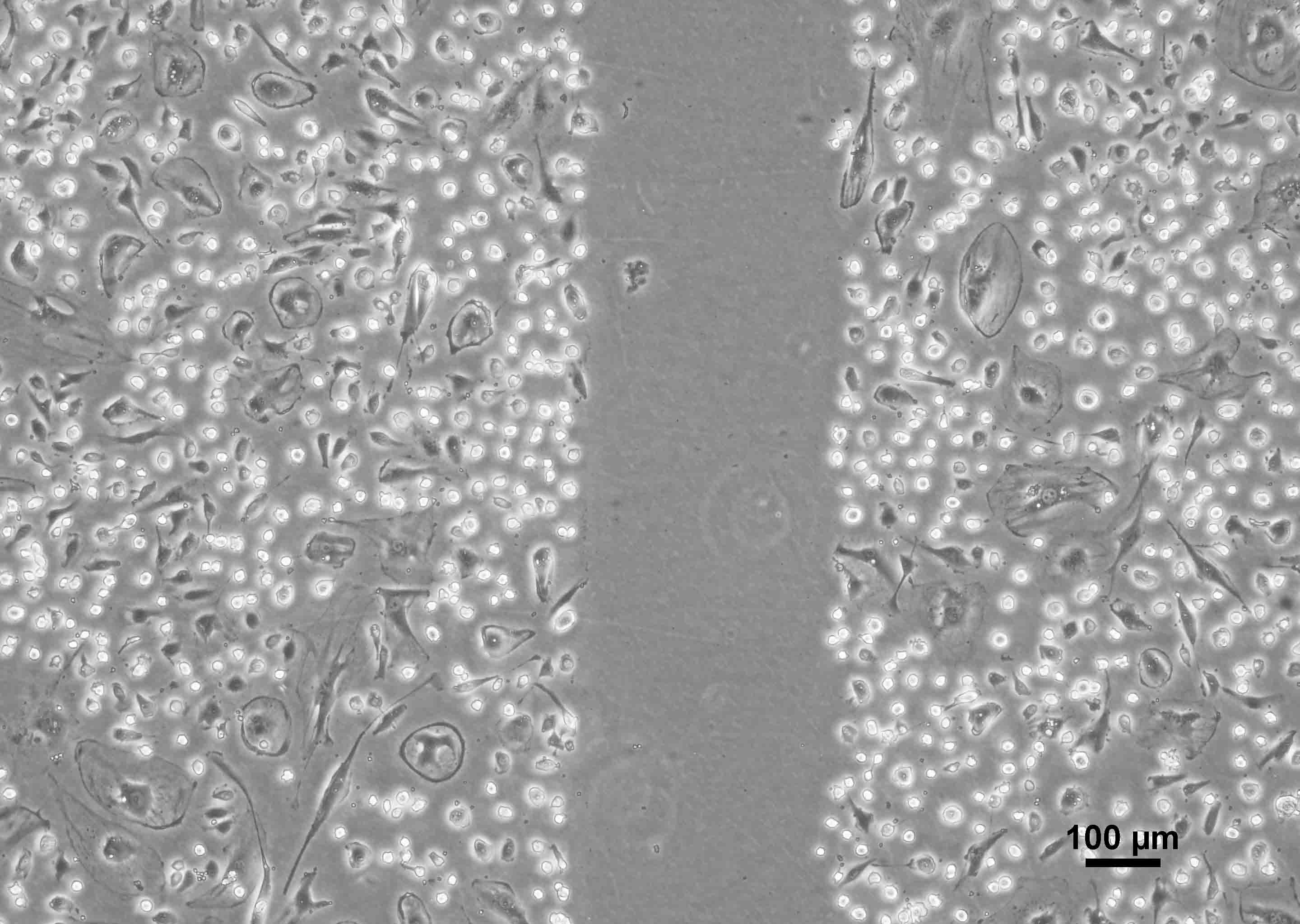

Supplement: Supplementary file 8 [file DataSheet2.zip › Figure 3 and 4/figure 3d/5ms 0h Image.tif]

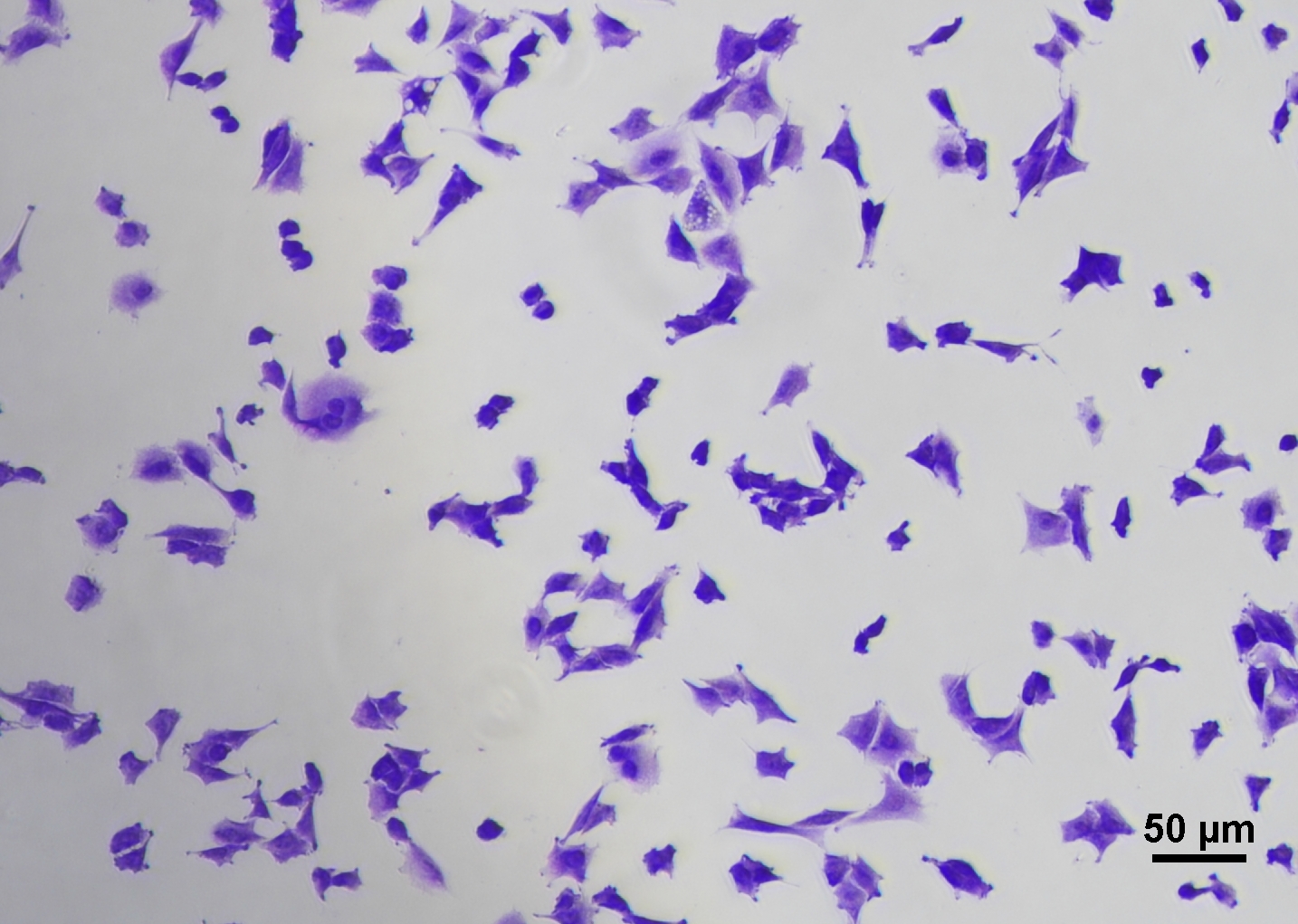

Supplement: Supplementary file 8 [file DataSheet2.zip › Figure 3 and 4/figure 3f/0MS.png]

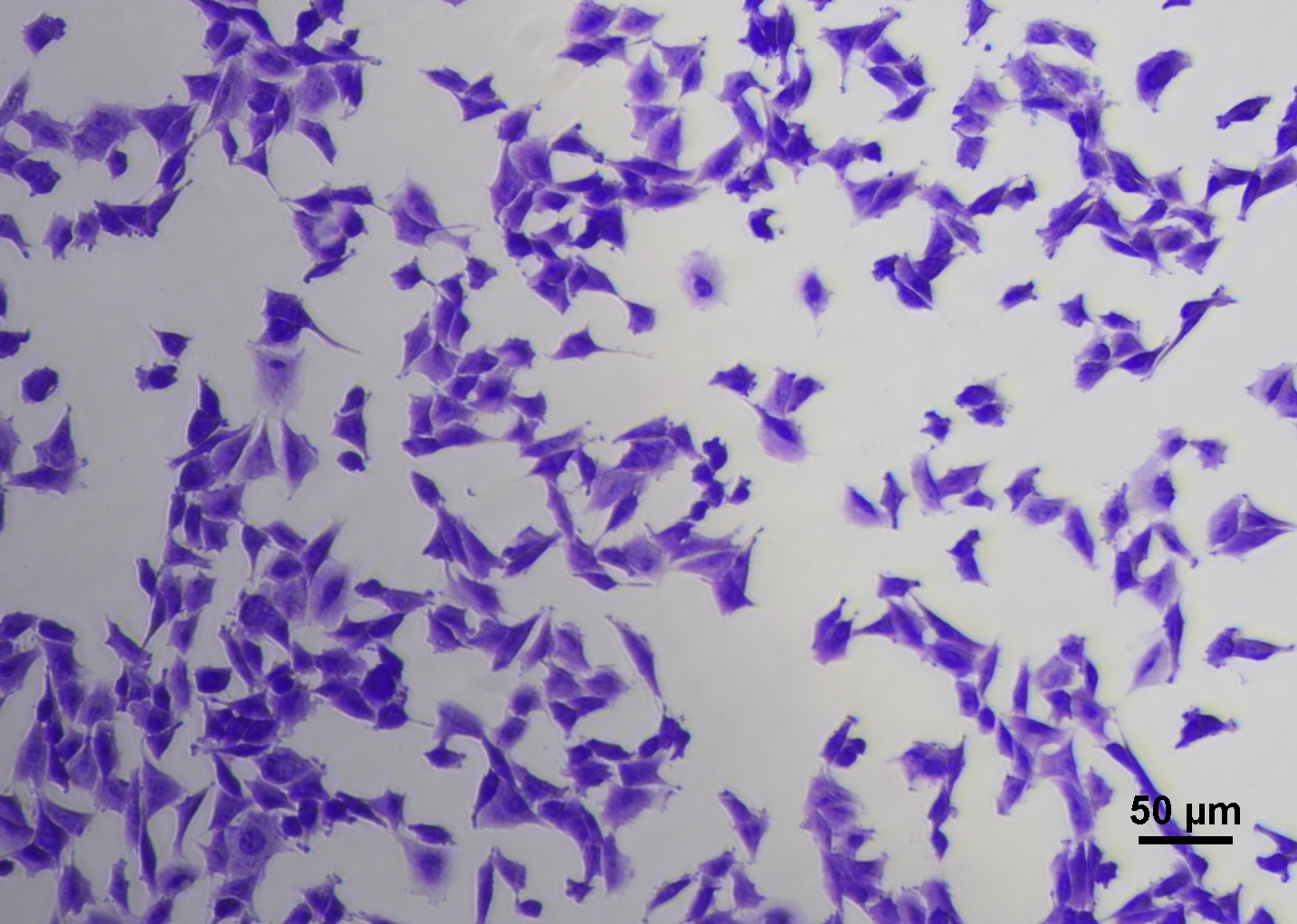

Supplement: Supplementary file 8 [file DataSheet2.zip › Figure 3 and 4/figure 3f/10MS.png]

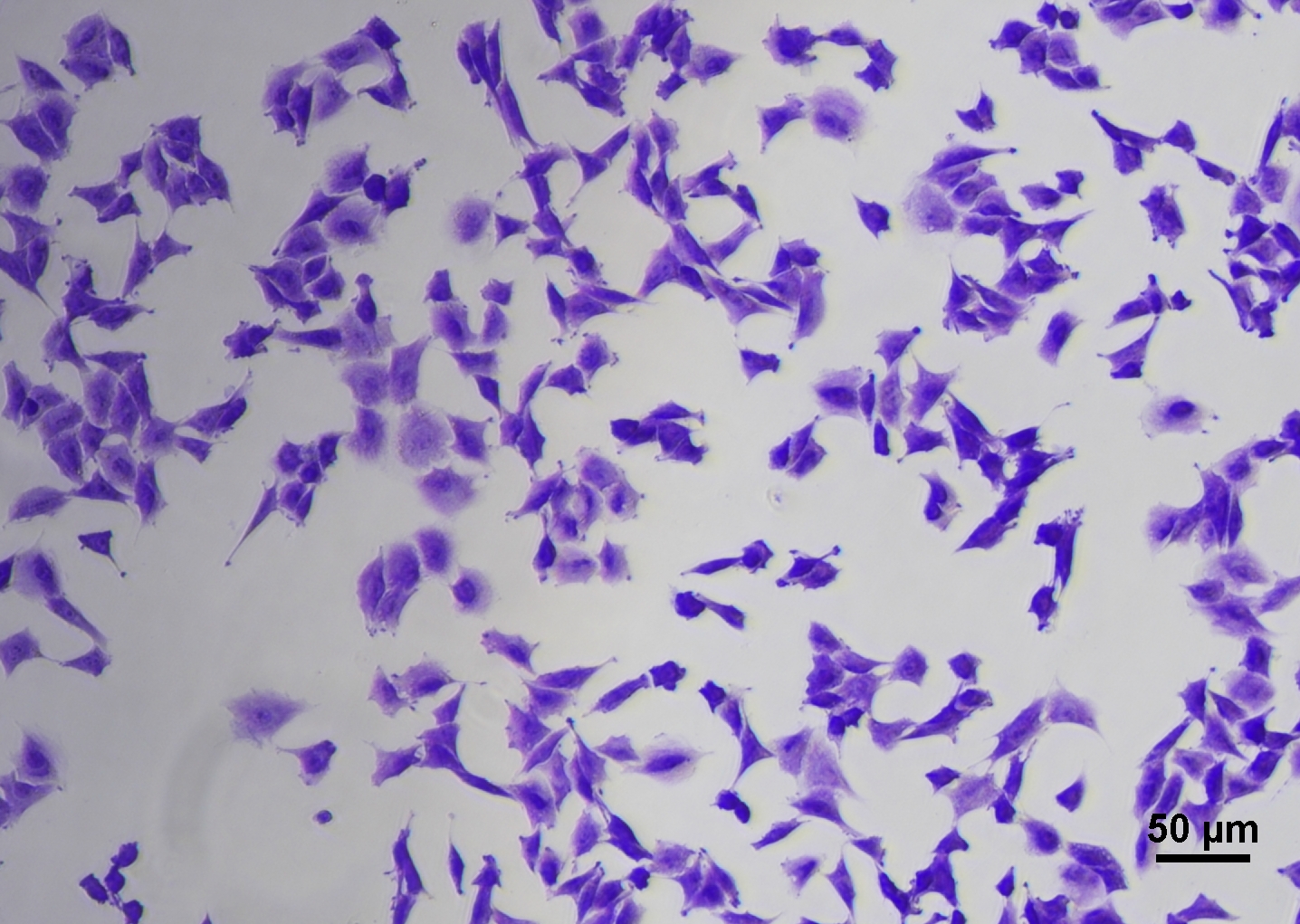

Supplement: Supplementary file 8 [file DataSheet2.zip › Figure 3 and 4/figure 3f/15MS.png]

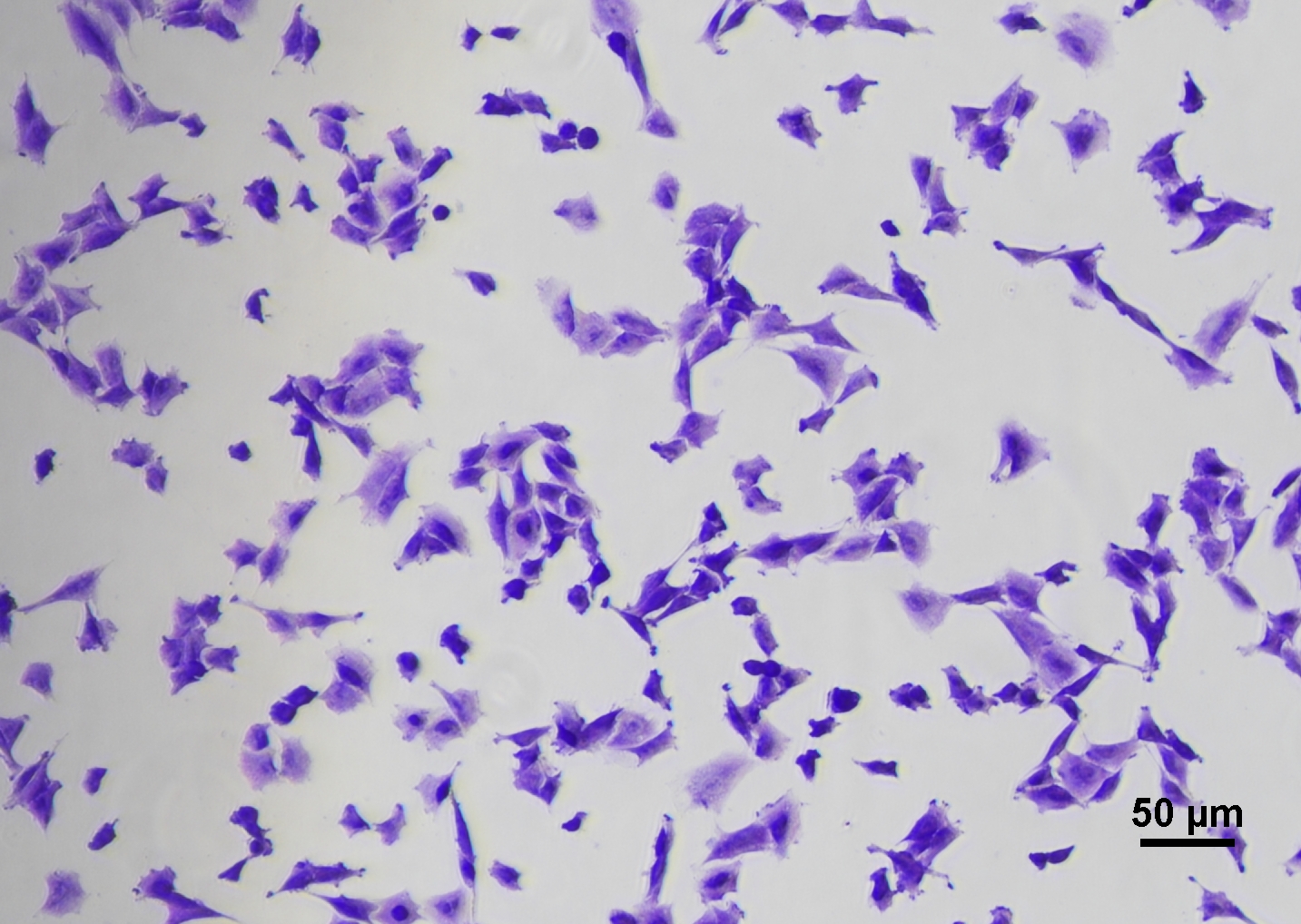

Supplement: Supplementary file 8 [file DataSheet2.zip › Figure 3 and 4/figure 3f/5MS.png]

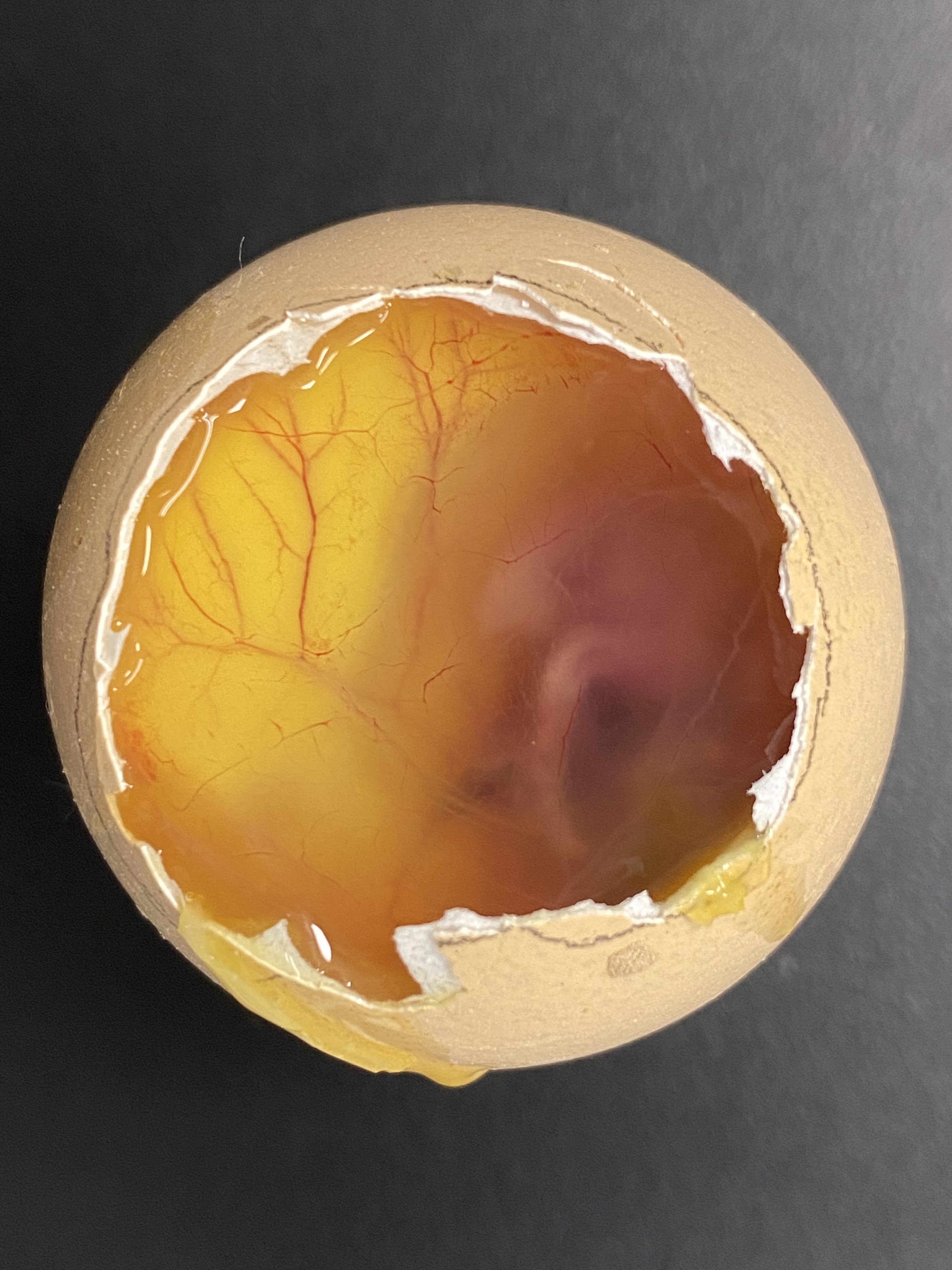

Supplement: Supplementary file 9 [file DataSheet5.zip › raw data_Figure8B D E L G H I J k/E LY294002.jpg]

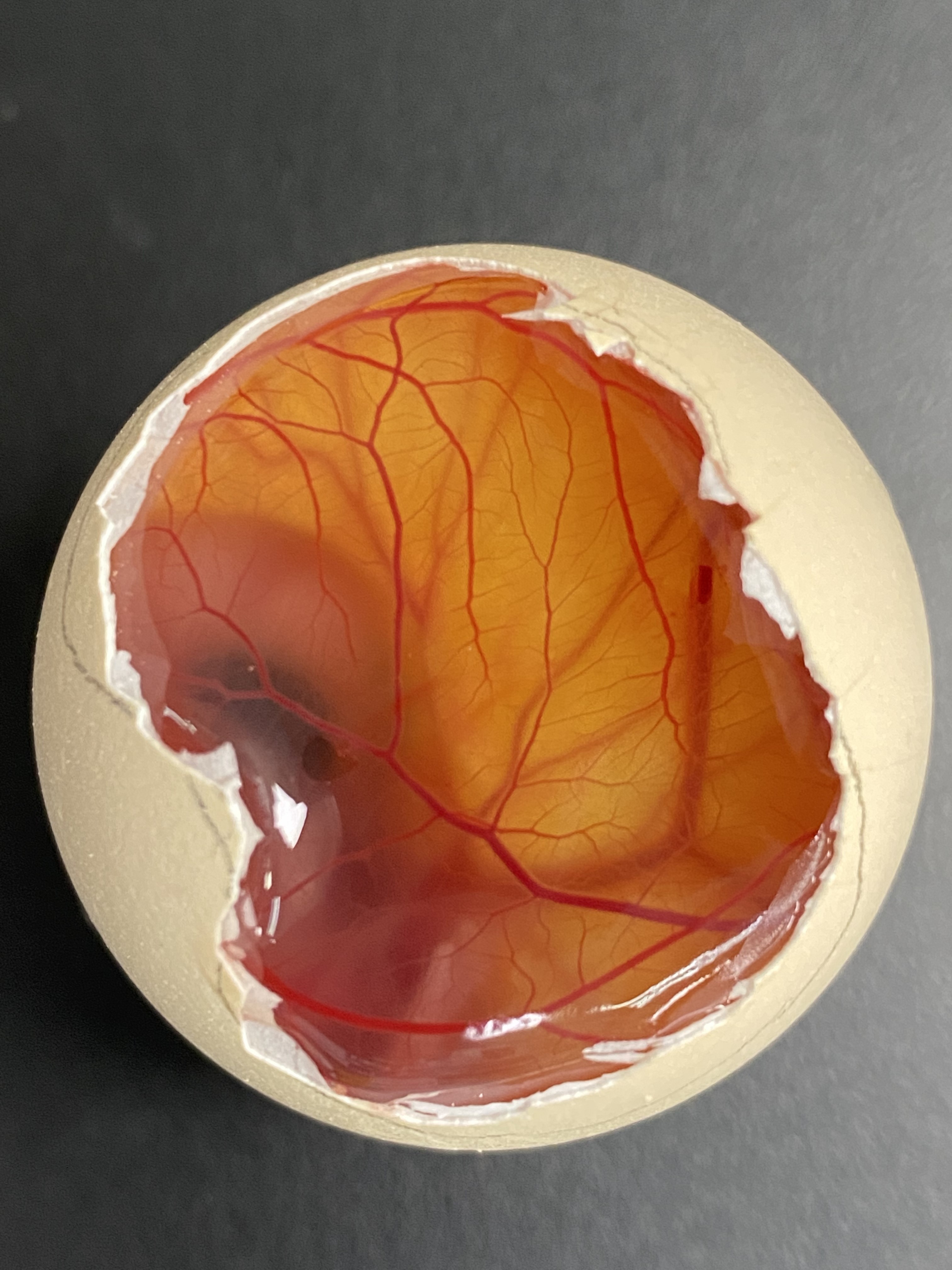

Supplement: Supplementary file 9 [file DataSheet5.zip › raw data_Figure8B D E L G H I J k/E MS.jpg]

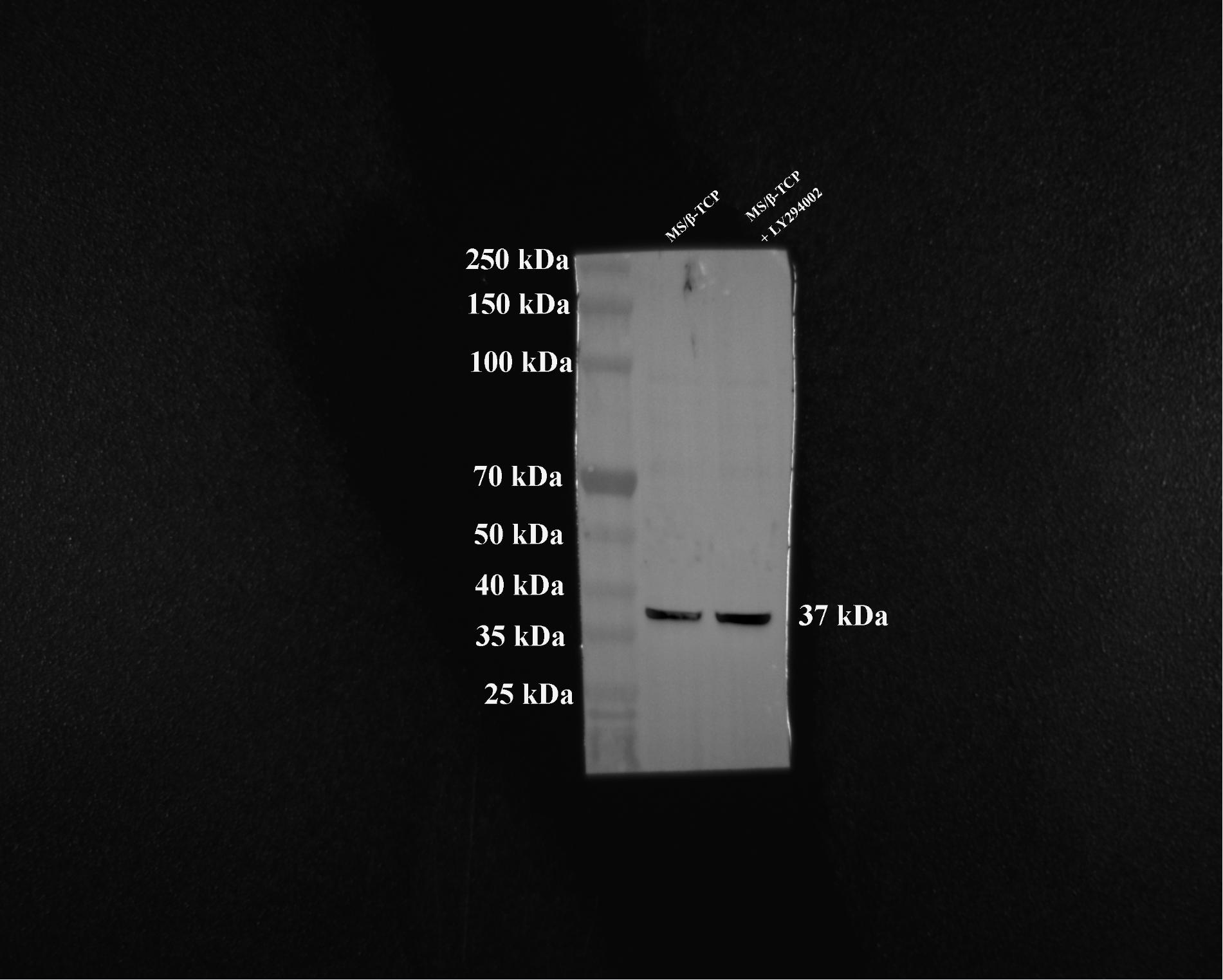

Supplement: Supplementary file 9 [file DataSheet5.zip › raw data_Figure8B D E L G H I J k/L/Figure 8L GAPDH.tif]

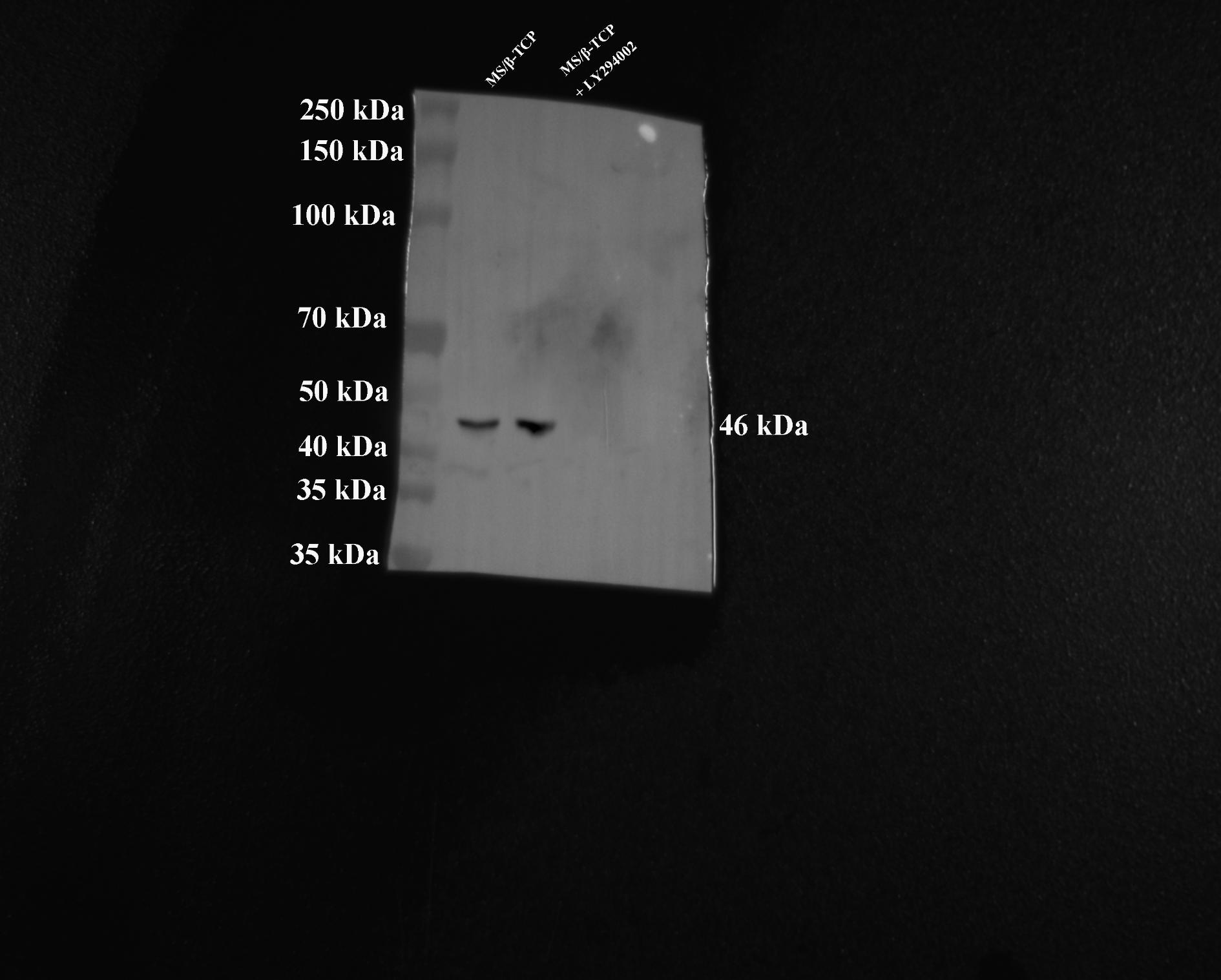

Supplement: Supplementary file 9 [file DataSheet5.zip › raw data_Figure8B D E L G H I J k/L/Figure 8L GSK-3b HUVEC.tif]

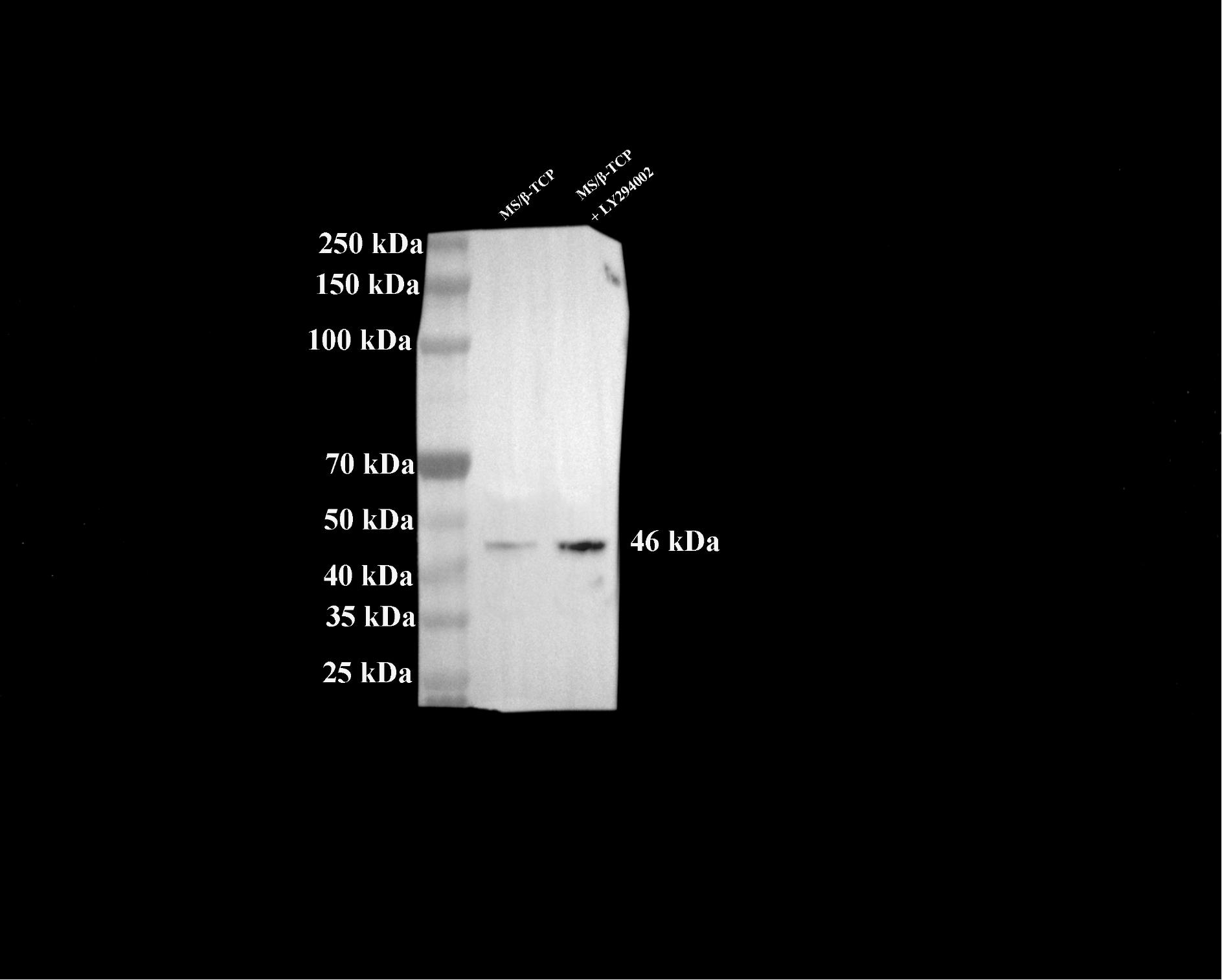

Supplement: Supplementary file 9 [file DataSheet5.zip › raw data_Figure8B D E L G H I J k/L/Figure 8L GSK-3b MC3T3.tif]

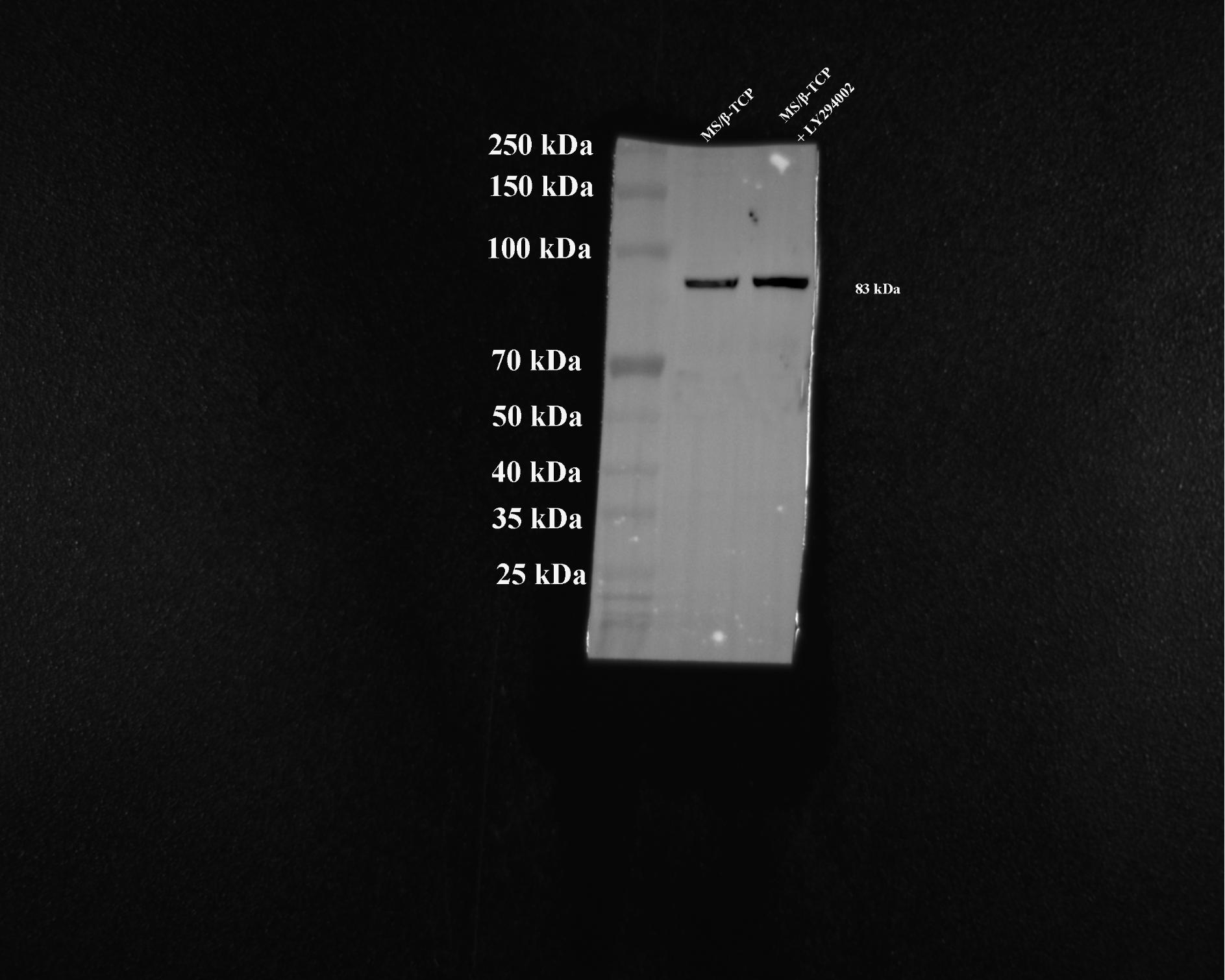

Supplement: Supplementary file 9 [file DataSheet5.zip › raw data_Figure8B D E L G H I J k/L/Figure 8L PI3K MC3T3-E1.tif]

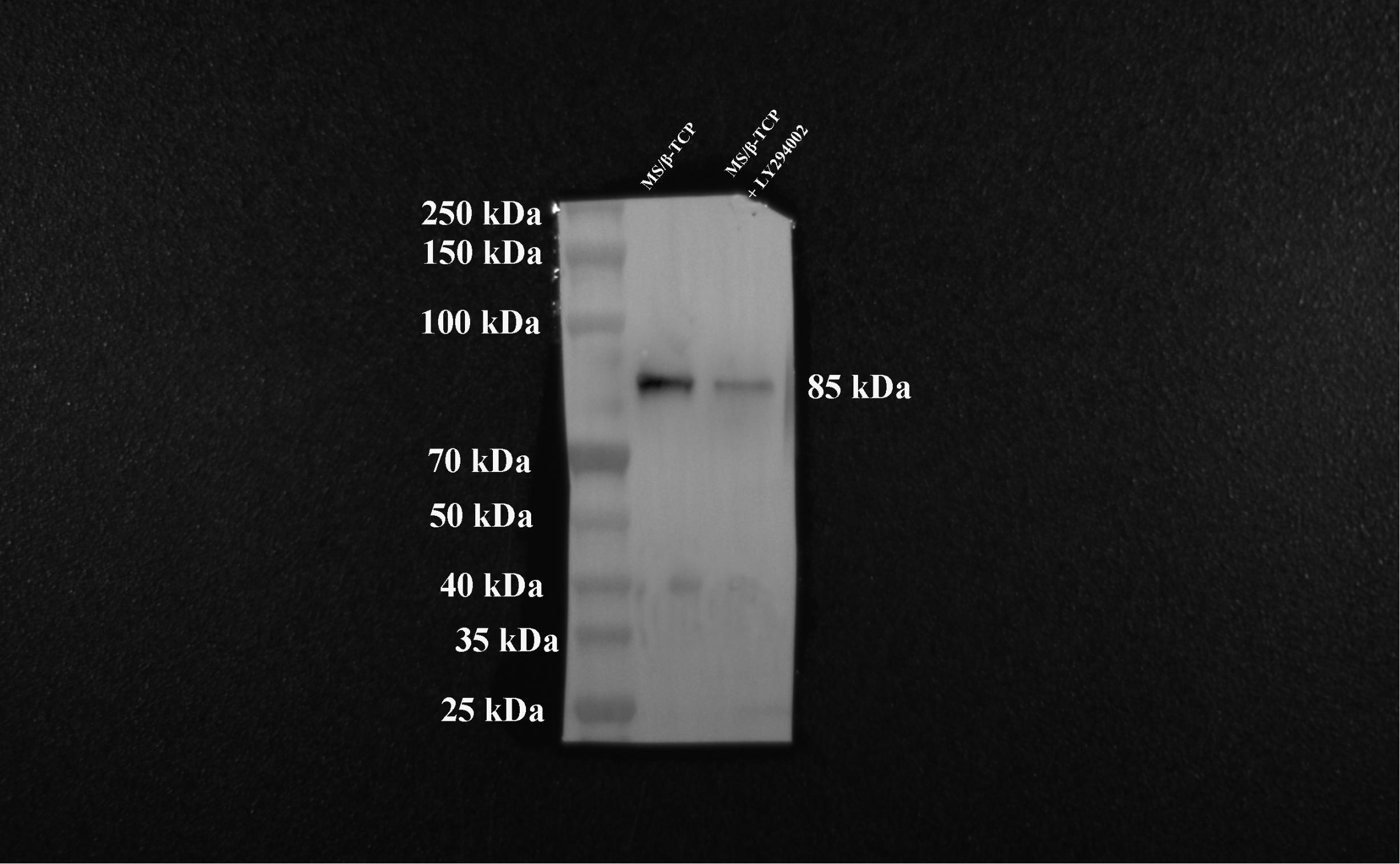

Supplement: Supplementary file 9 [file DataSheet5.zip › raw data_Figure8B D E L G H I J k/L/Figure 8L b Catenin HUVEC.tif]

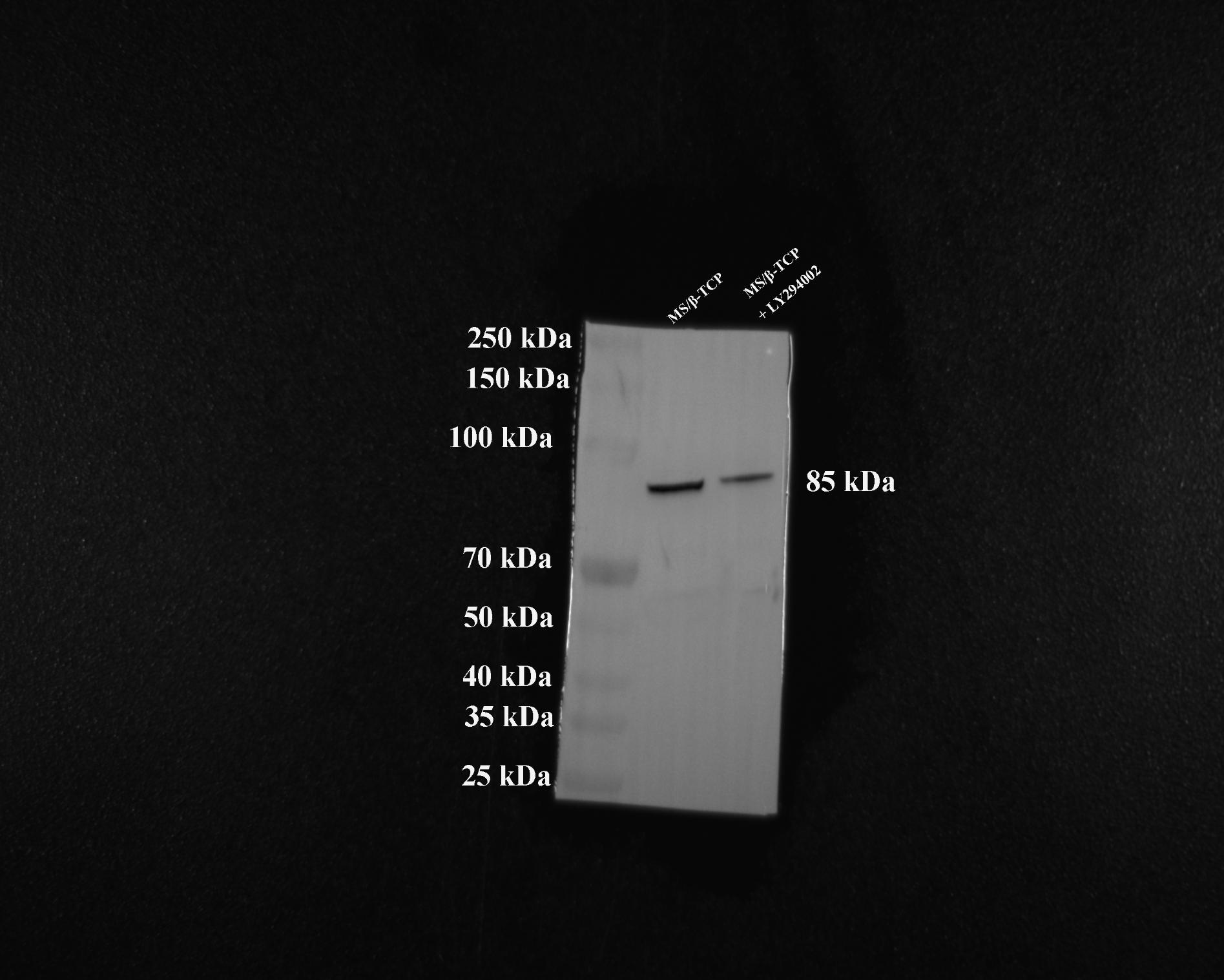

Supplement: Supplementary file 9 [file DataSheet5.zip › raw data_Figure8B D E L G H I J k/L/Figure 8L b catenin MC3T3.tif]

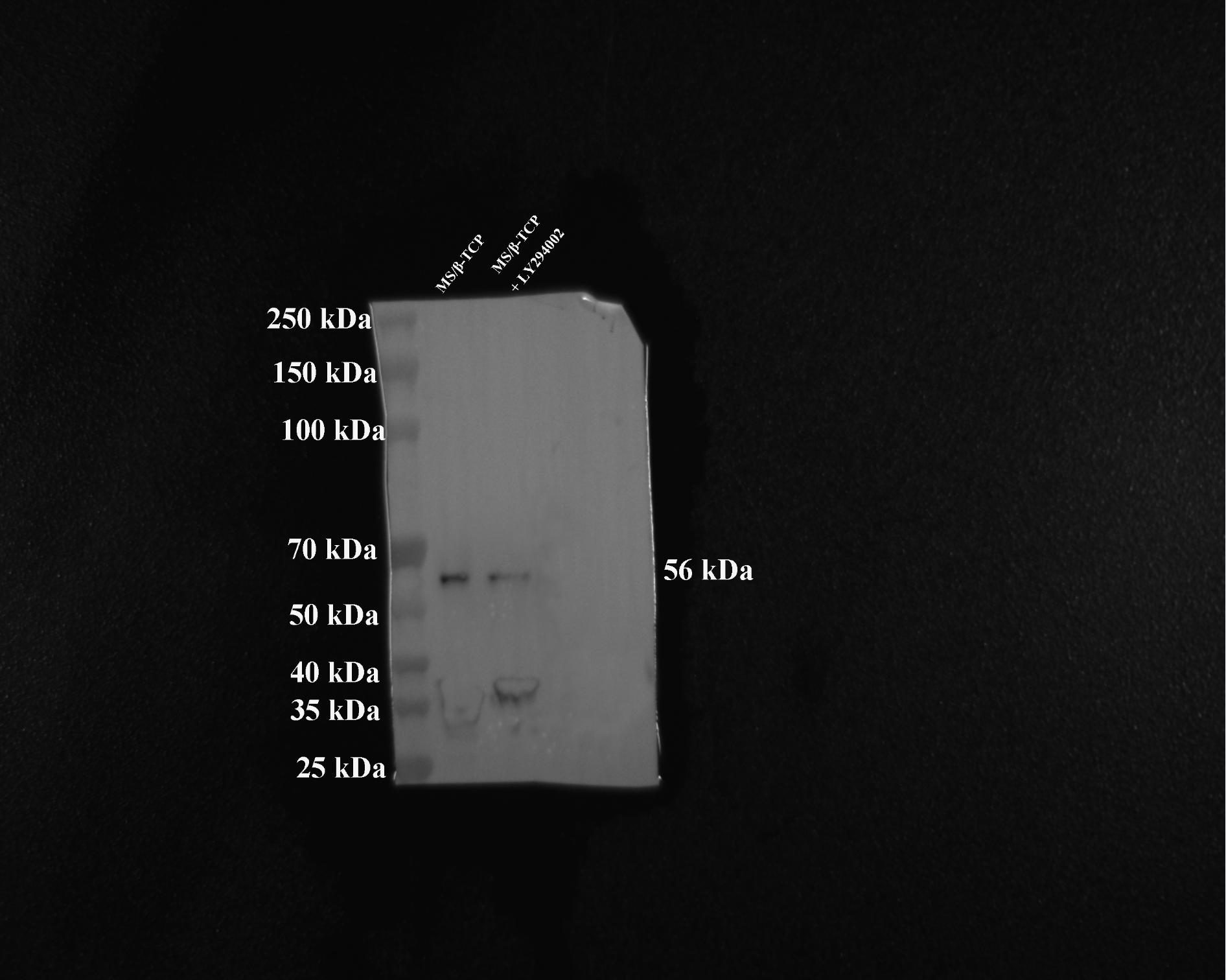

Supplement: Supplementary file 9 [file DataSheet5.zip › raw data_Figure8B D E L G H I J k/L/Figure 8L p-AKT HUVEC.tif]

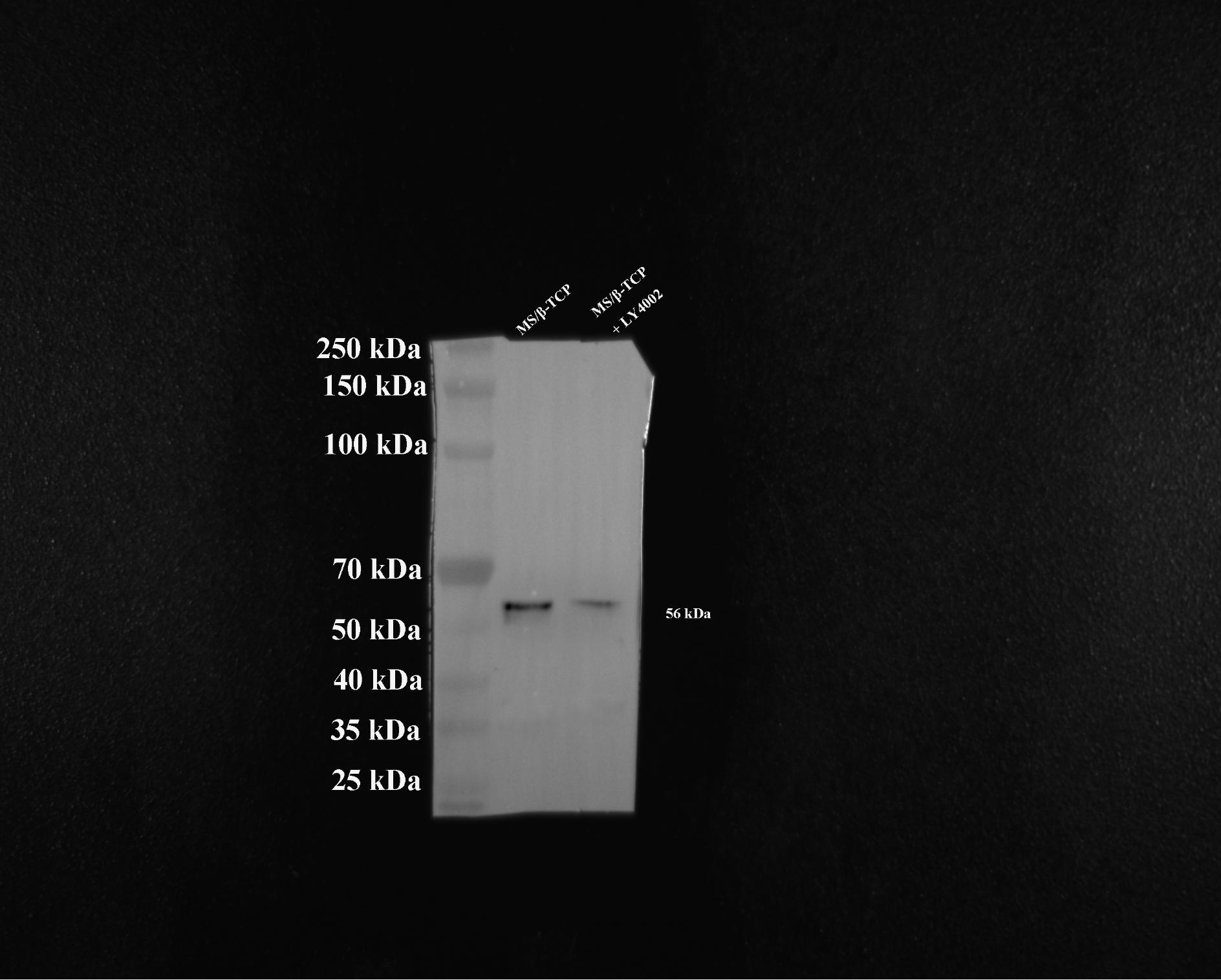

Supplement: Supplementary file 9 [file DataSheet5.zip › raw data_Figure8B D E L G H I J k/L/Figure 8L p-AKT MC3T3.tif]

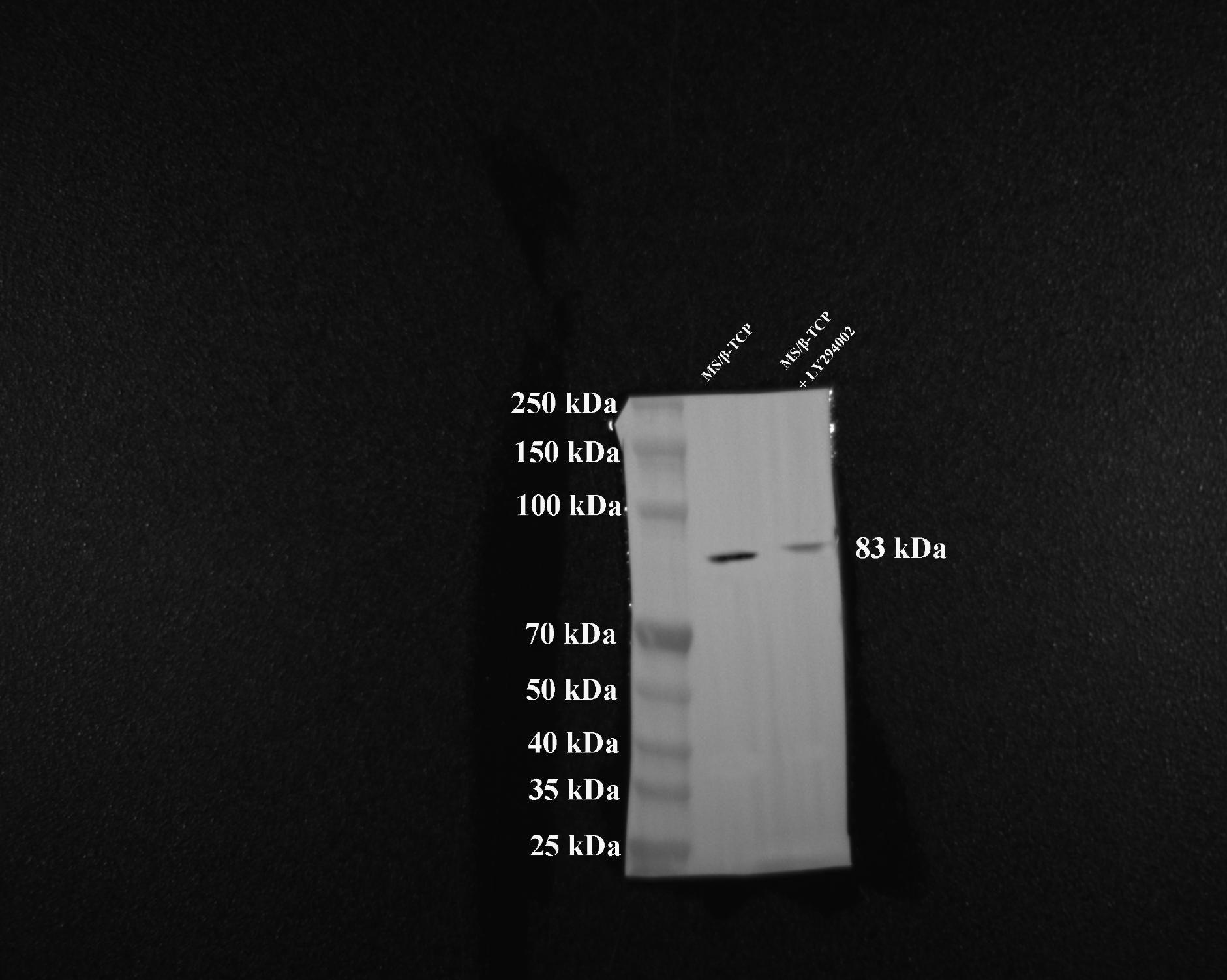

Supplement: Supplementary file 9 [file DataSheet5.zip › raw data_Figure8B D E L G H I J k/L/Figure 8L p-PI3K HUVEC.tif]

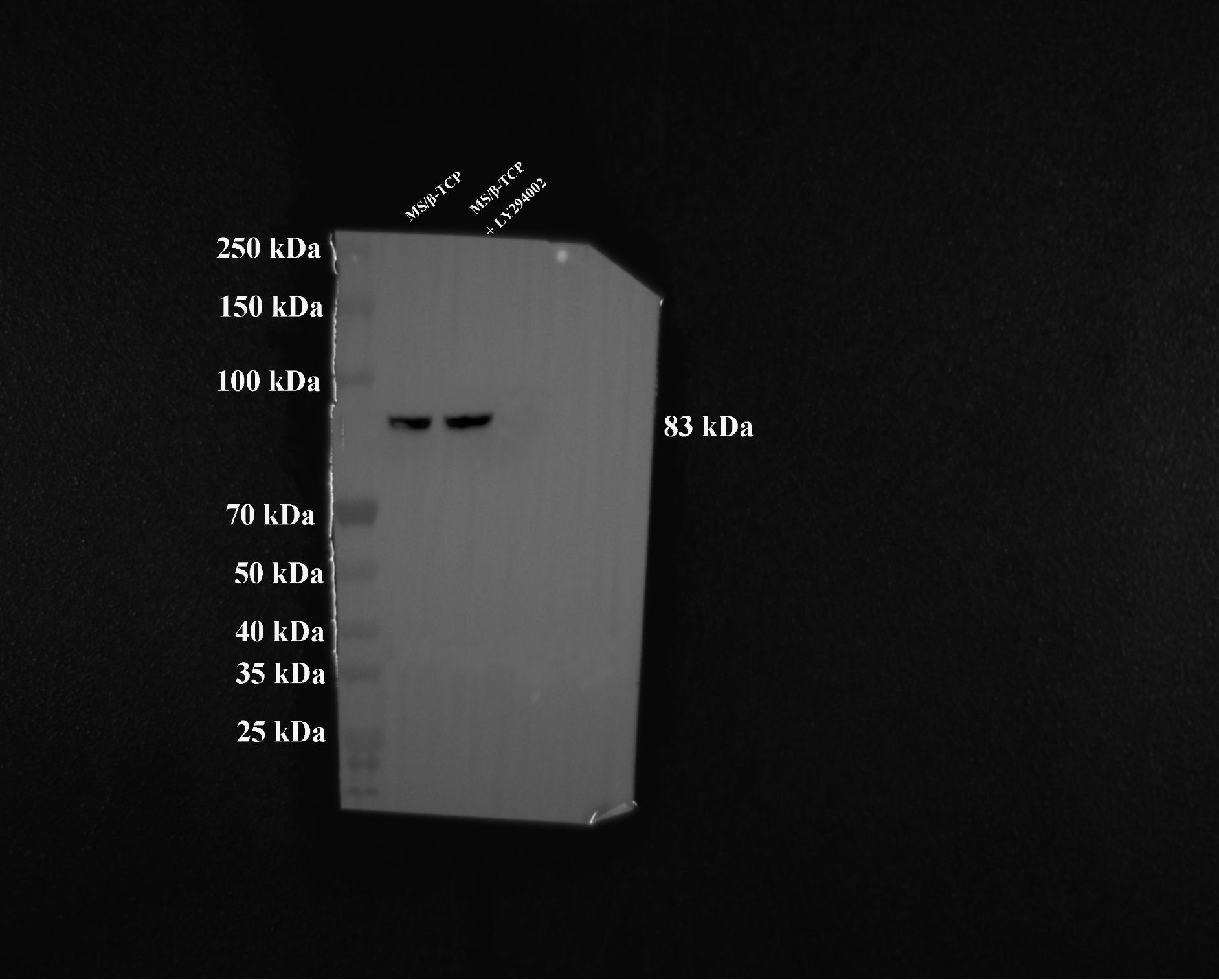

Supplement: Supplementary file 9 [file DataSheet5.zip › raw data_Figure8B D E L G H I J k/L/Figure 8L pi3k HUVEC.tif]

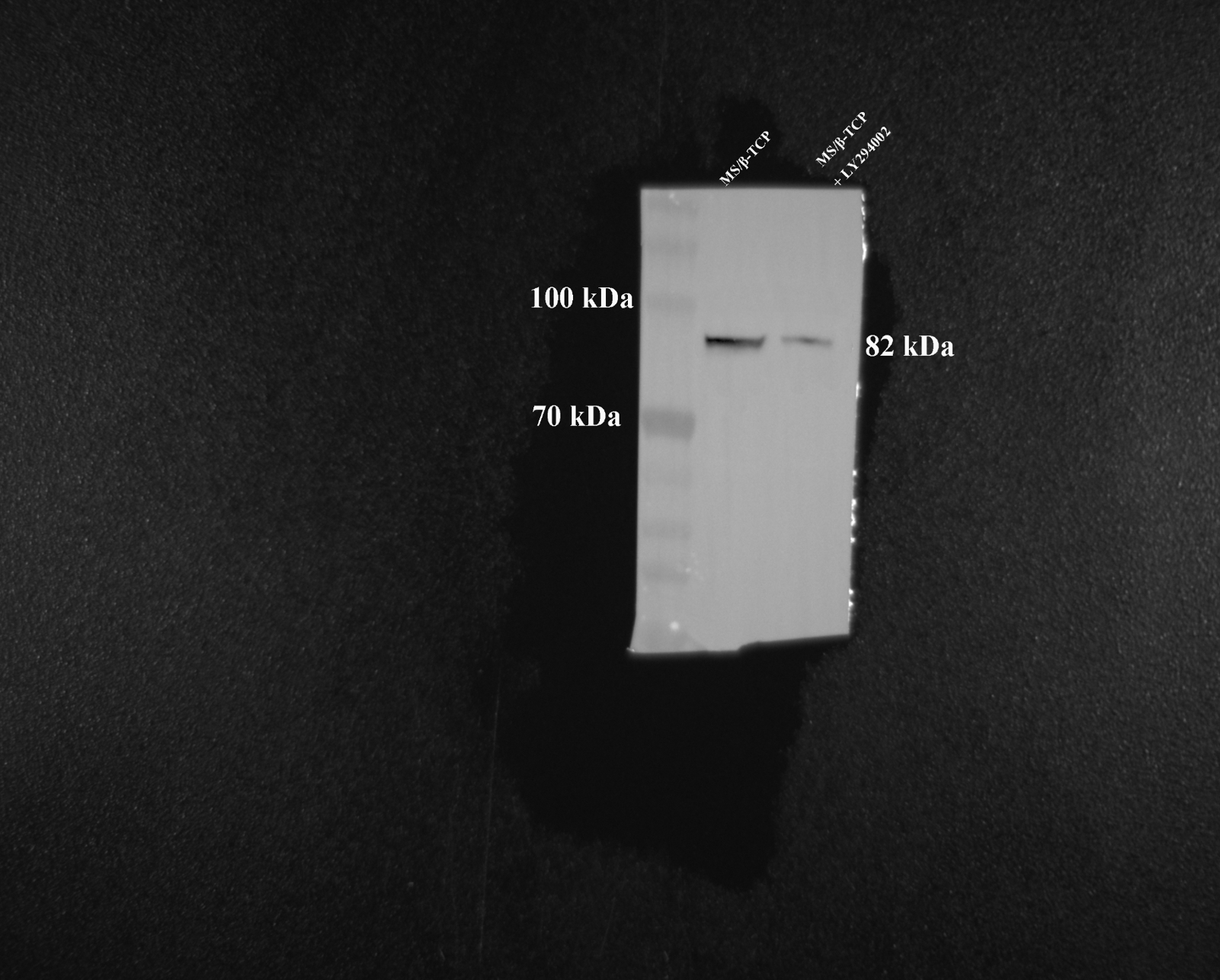

Supplement: Supplementary file 9 [file DataSheet5.zip › raw data_Figure8B D E L G H I J k/k/CD31.png]

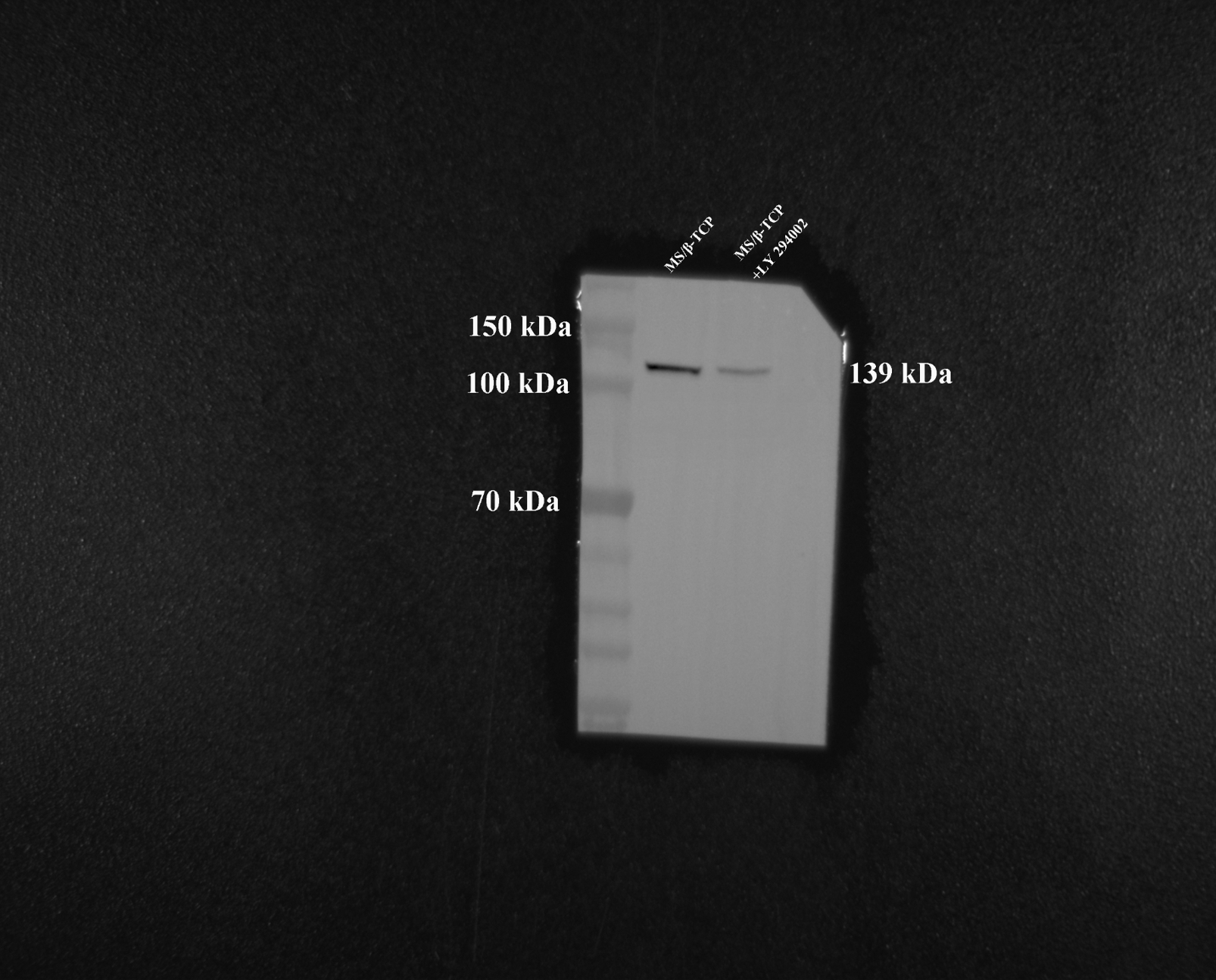

Supplement: Supplementary file 9 [file DataSheet5.zip › raw data_Figure8B D E L G H I J k/k/COL 1A1.png]

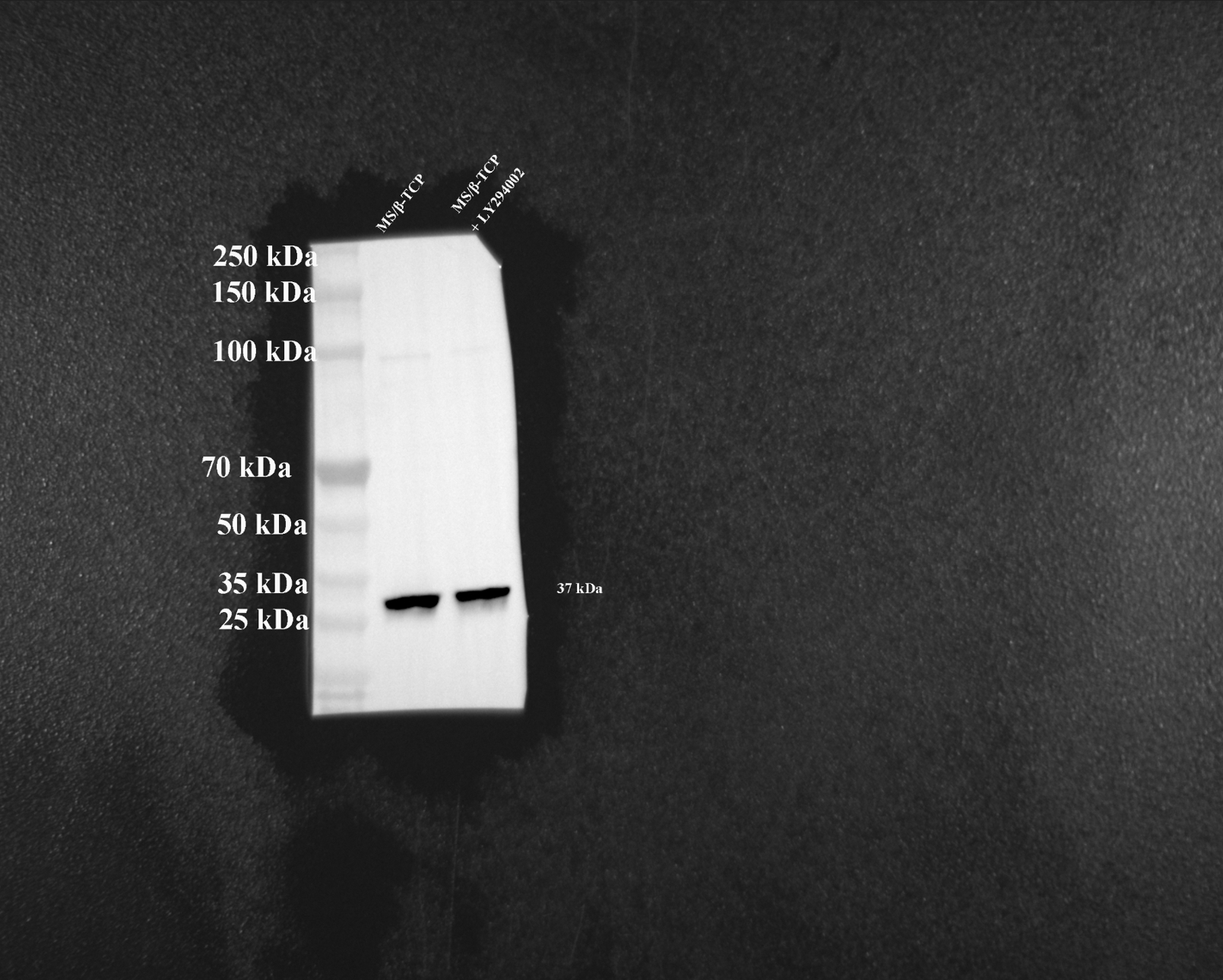

Supplement: Supplementary file 9 [file DataSheet5.zip › raw data_Figure8B D E L G H I J k/k/GAPDH.png]

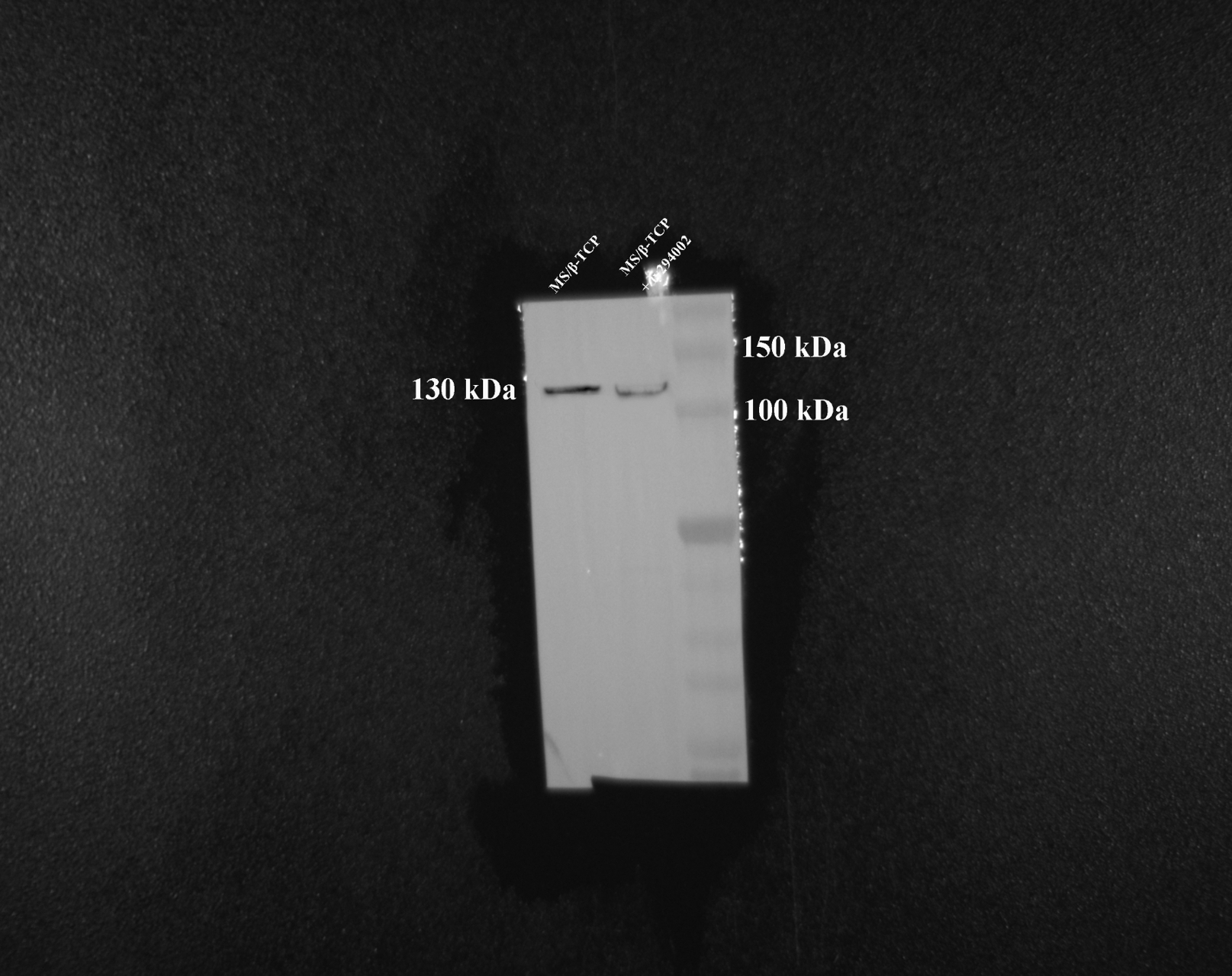

Supplement: Supplementary file 9 [file DataSheet5.zip › raw data_Figure8B D E L G H I J k/k/HIF 1a.png]

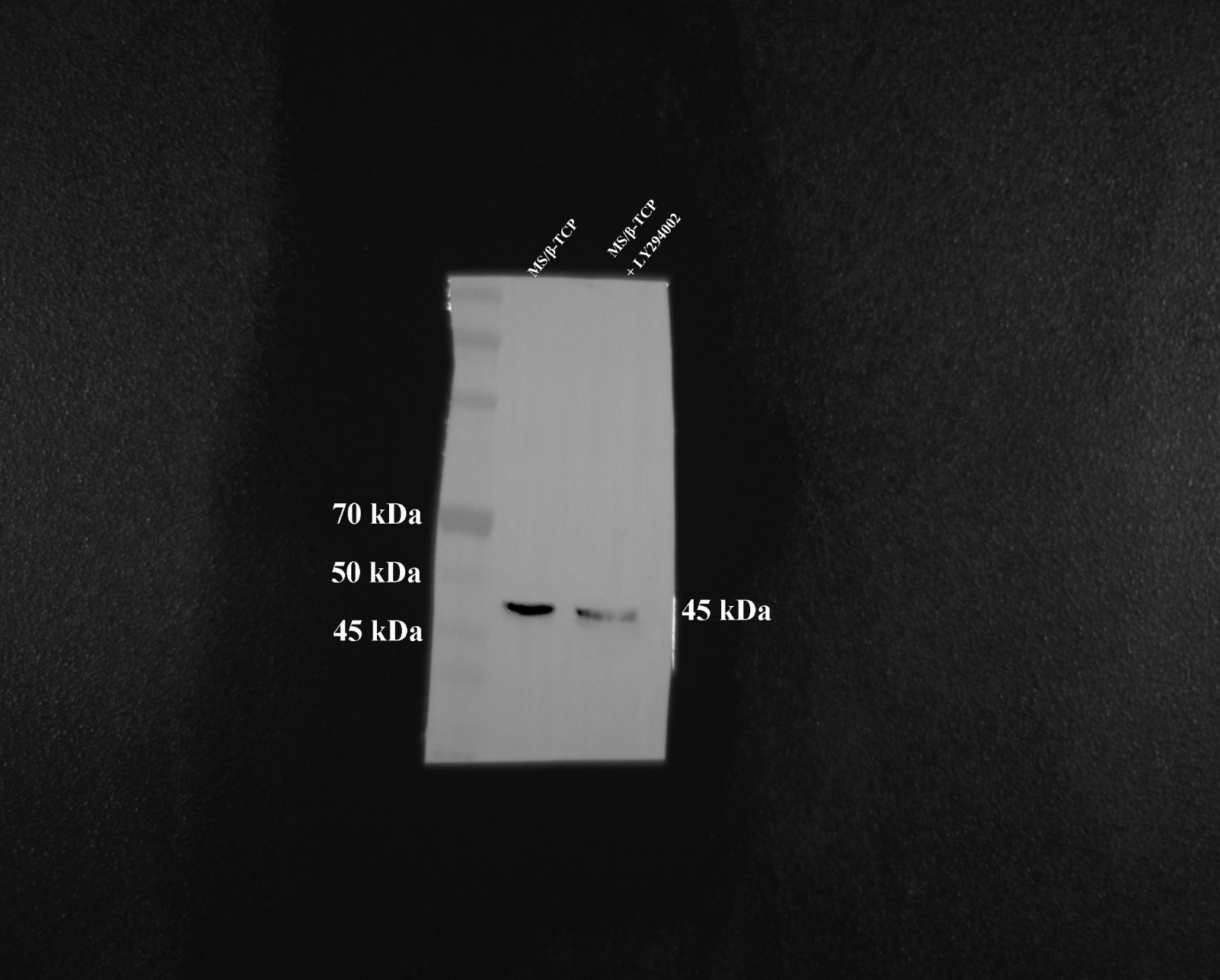

Supplement: Supplementary file 9 [file DataSheet5.zip › raw data_Figure8B D E L G H I J k/k/Osterix.png]

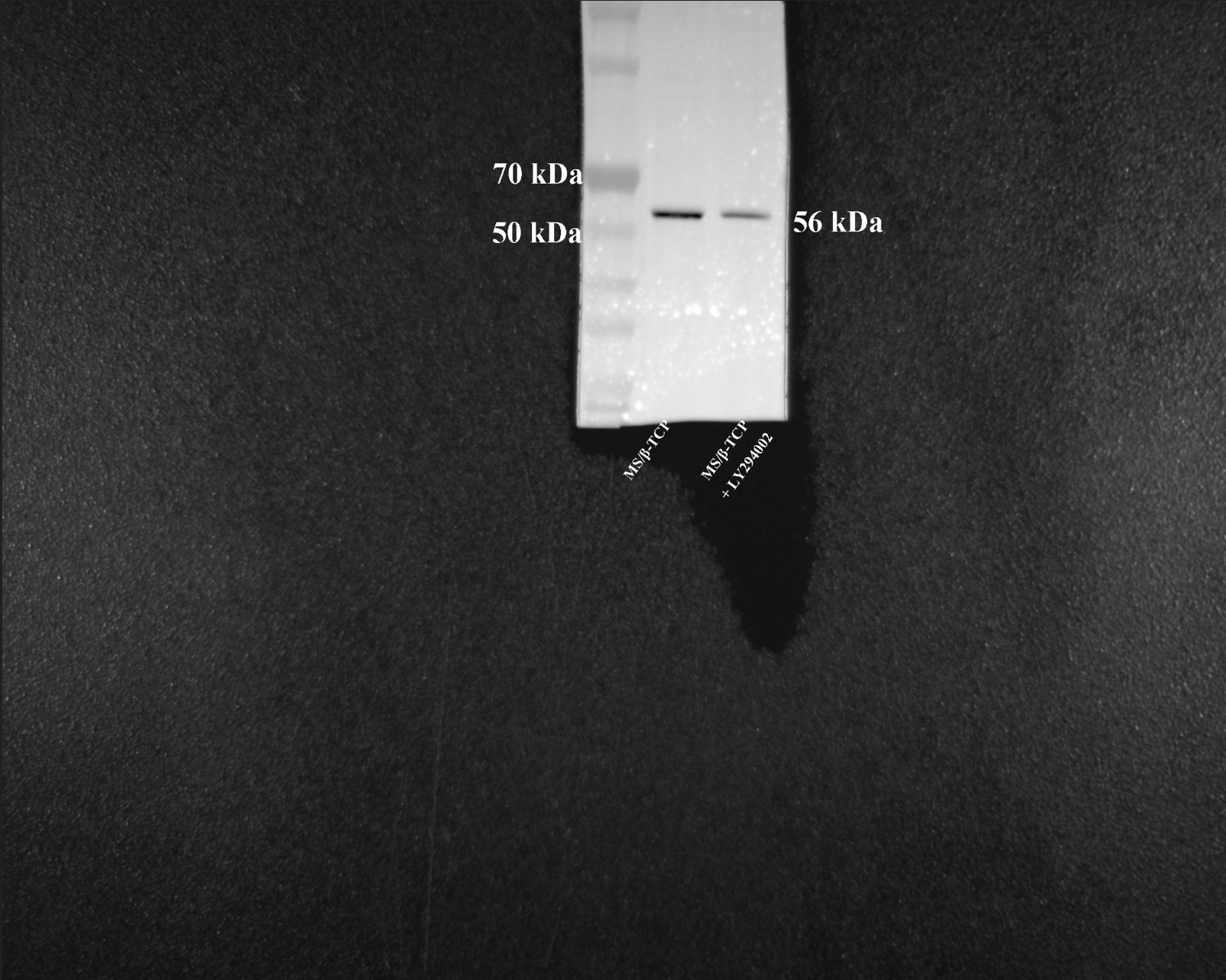

Supplement: Supplementary file 9 [file DataSheet5.zip › raw data_Figure8B D E L G H I J k/k/RUNX2.png]

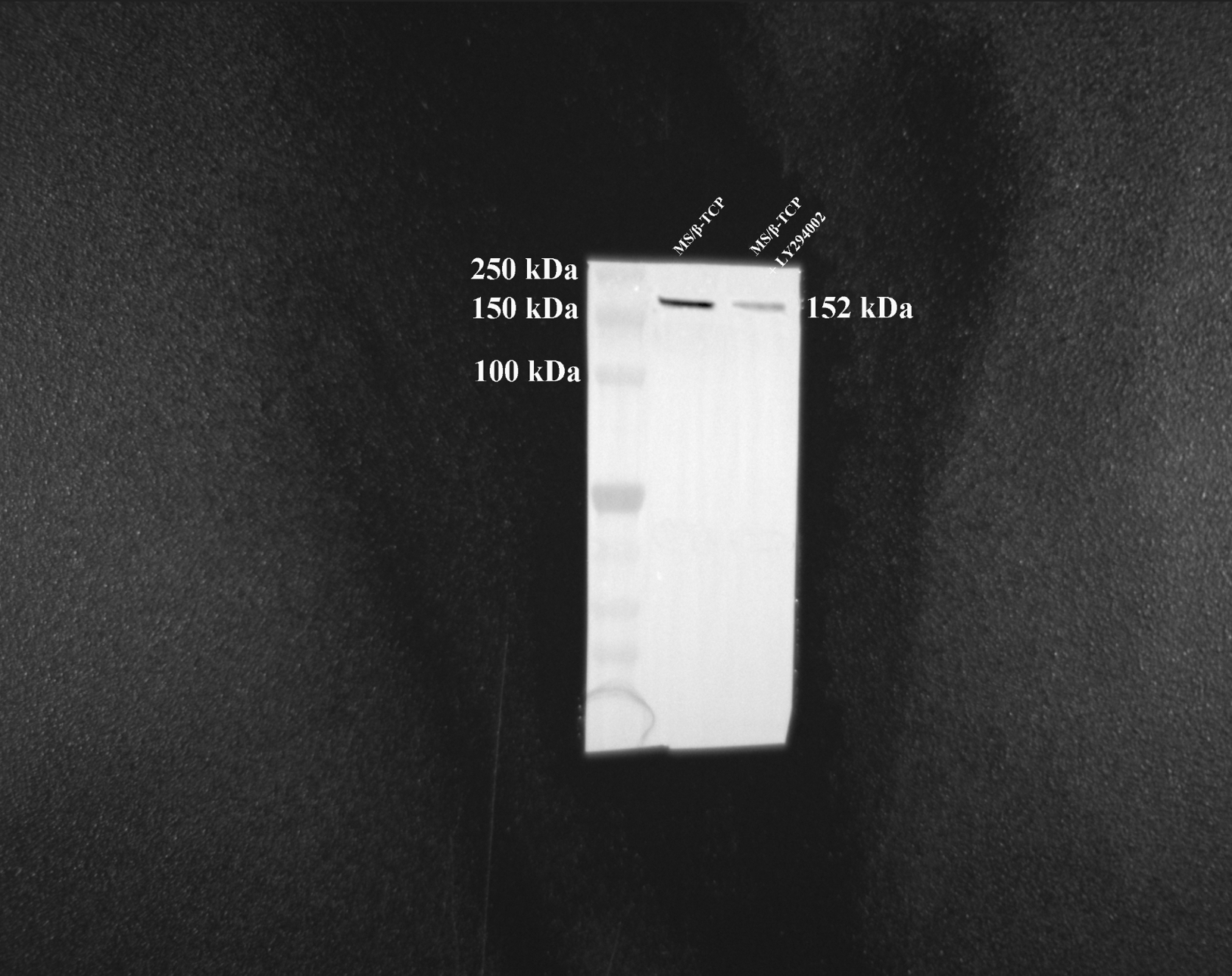

Supplement: Supplementary file 9 [file DataSheet5.zip › raw data_Figure8B D E L G H I J k/k/VEGF.png]

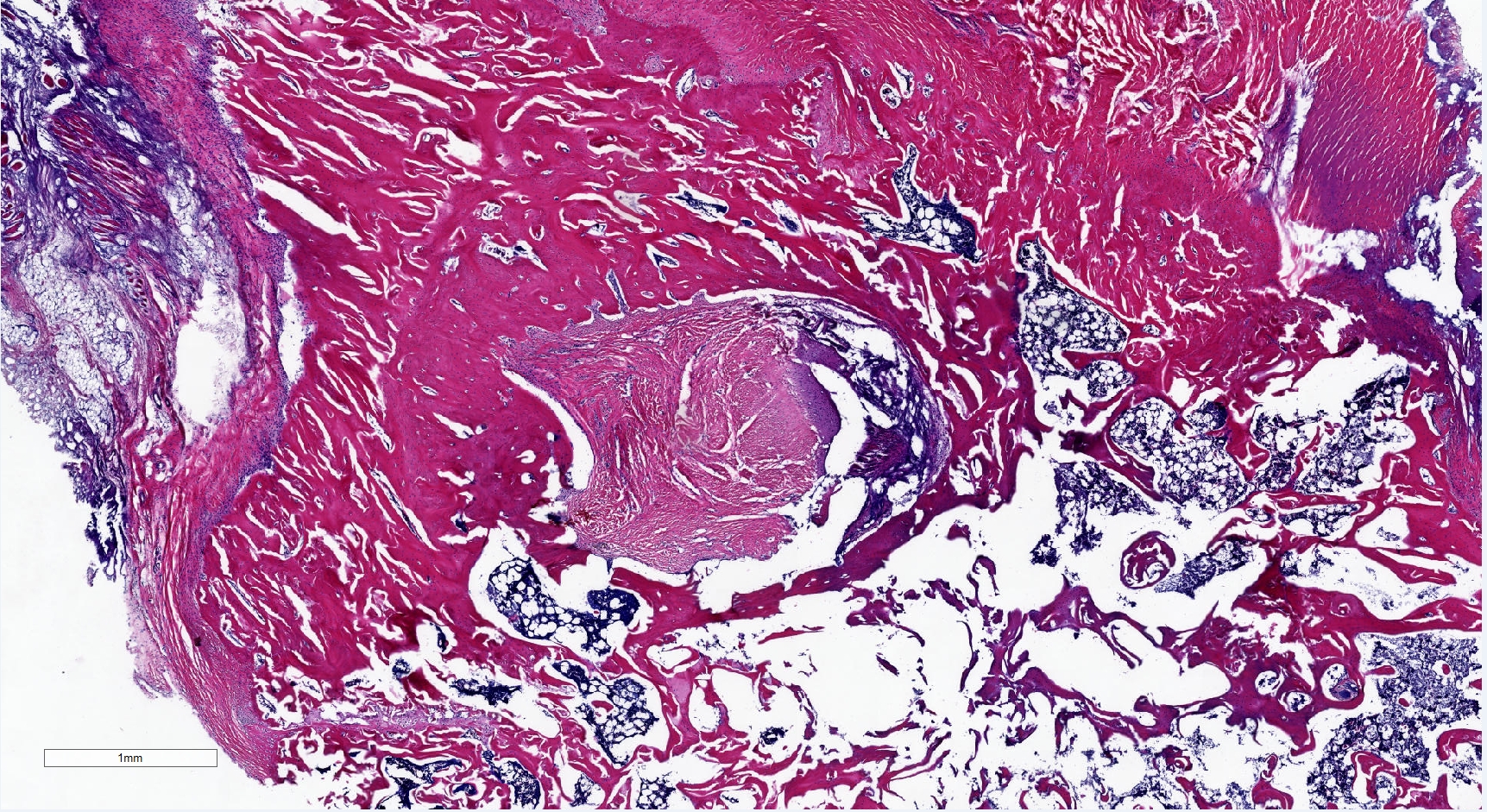

Supplement: Supplementary file 10 [file DataSheet7.zip › raw data_Figure 11A/Blank 12w.png]

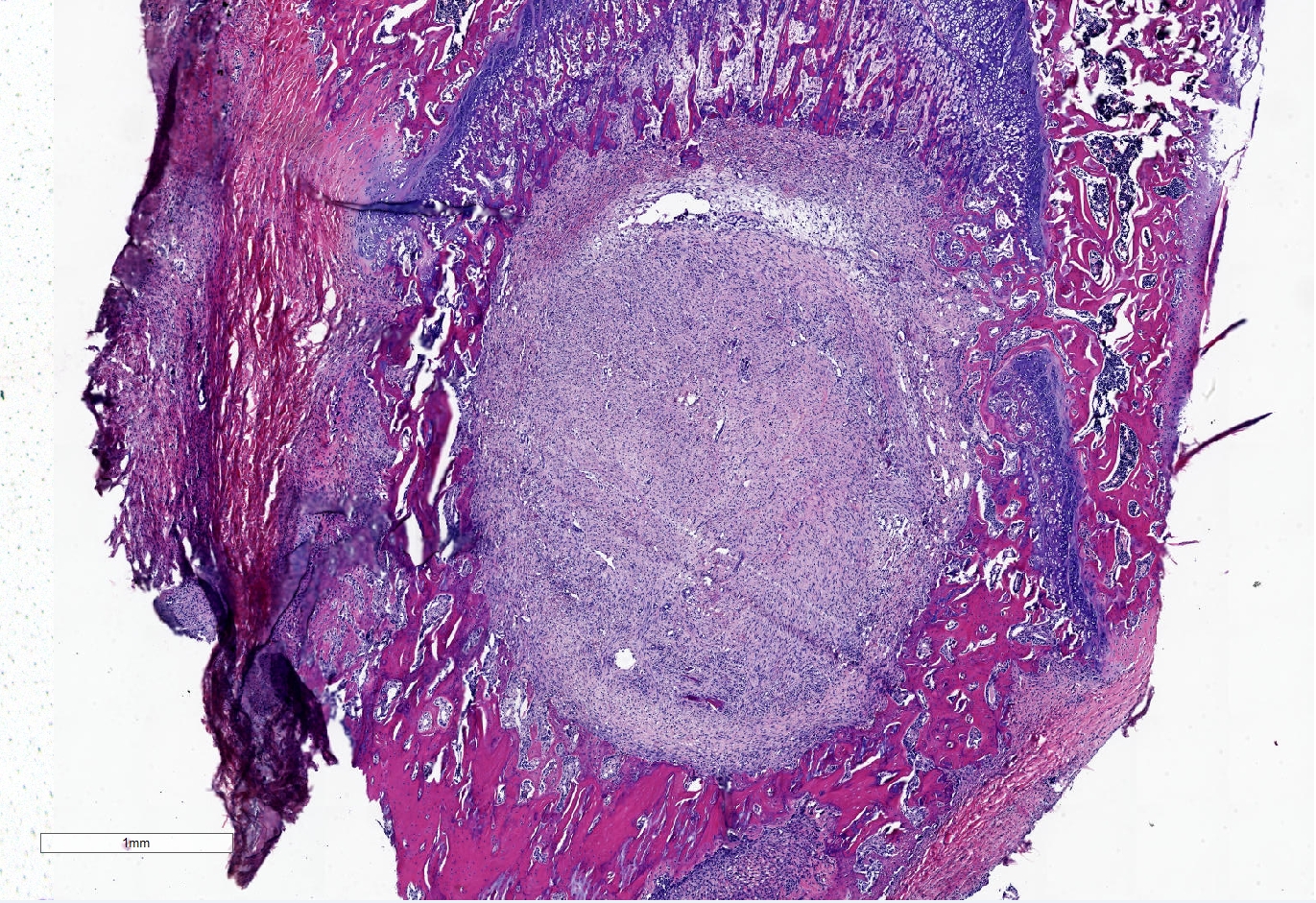

Supplement: Supplementary file 10 [file DataSheet7.zip › raw data_Figure 11A/Blank 4w.png]

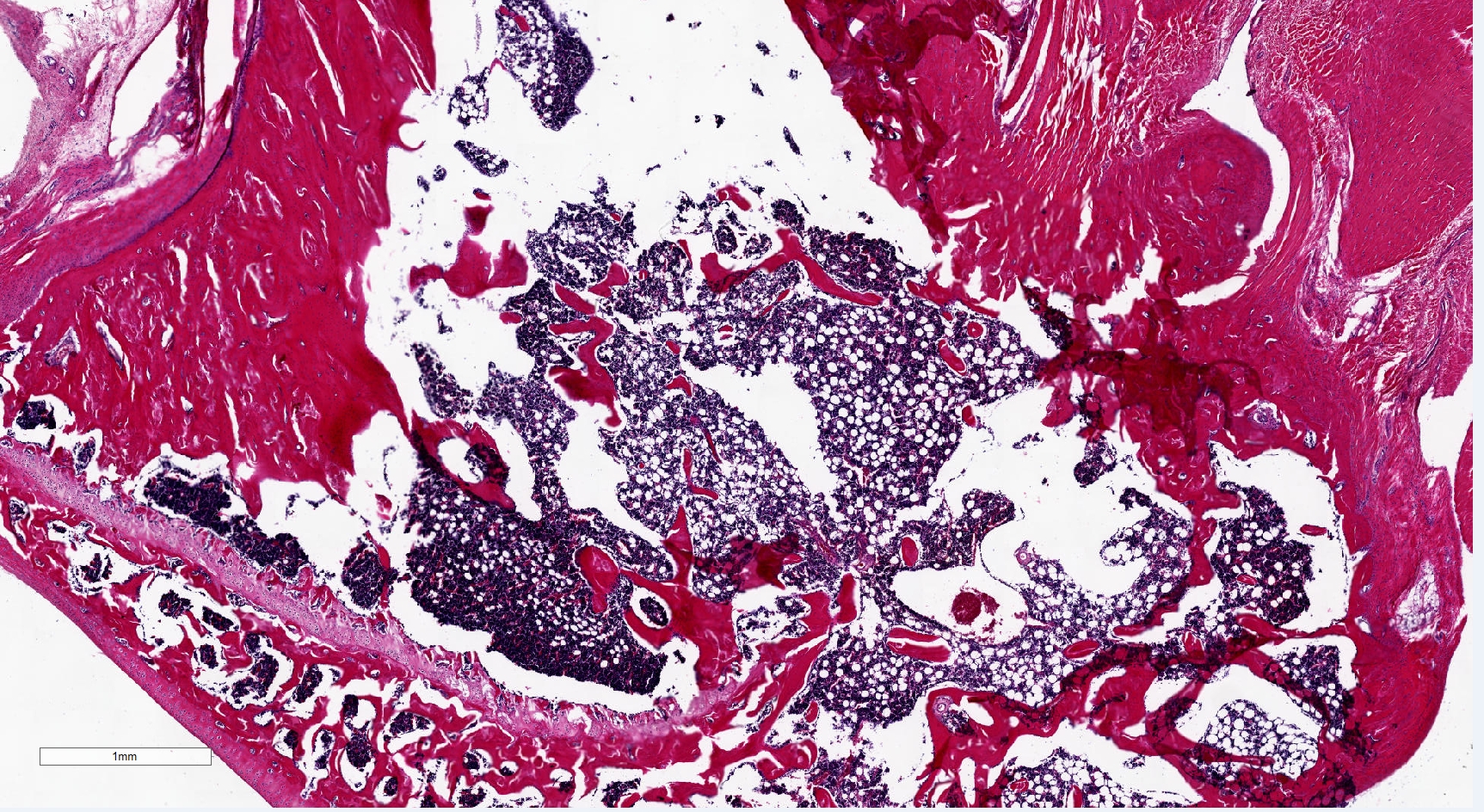

Supplement: Supplementary file 10 [file DataSheet7.zip › raw data_Figure 11A/Blank 8w.png]

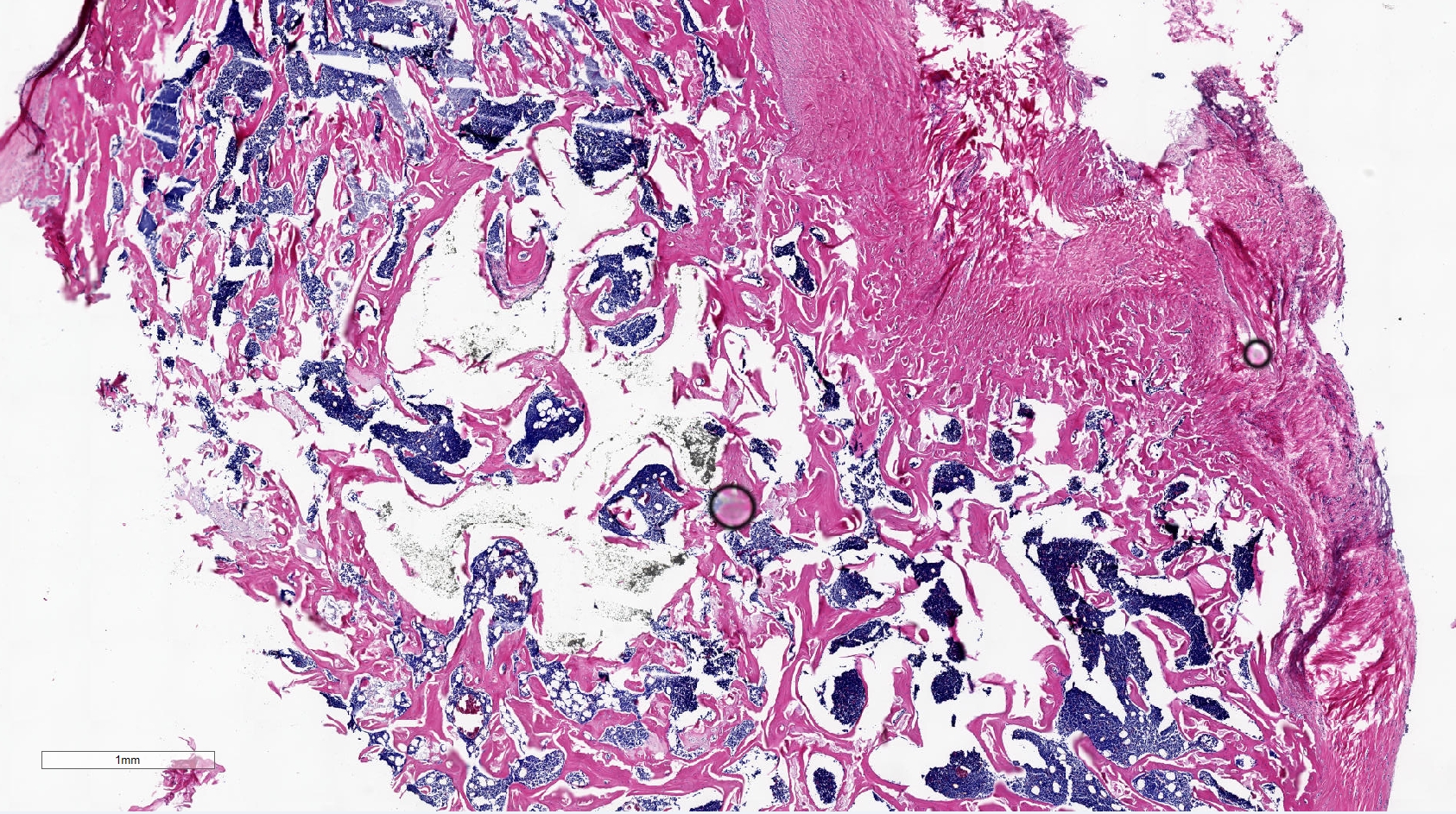

Supplement: Supplementary file 10 [file DataSheet7.zip › raw data_Figure 11A/MS 12W.png]

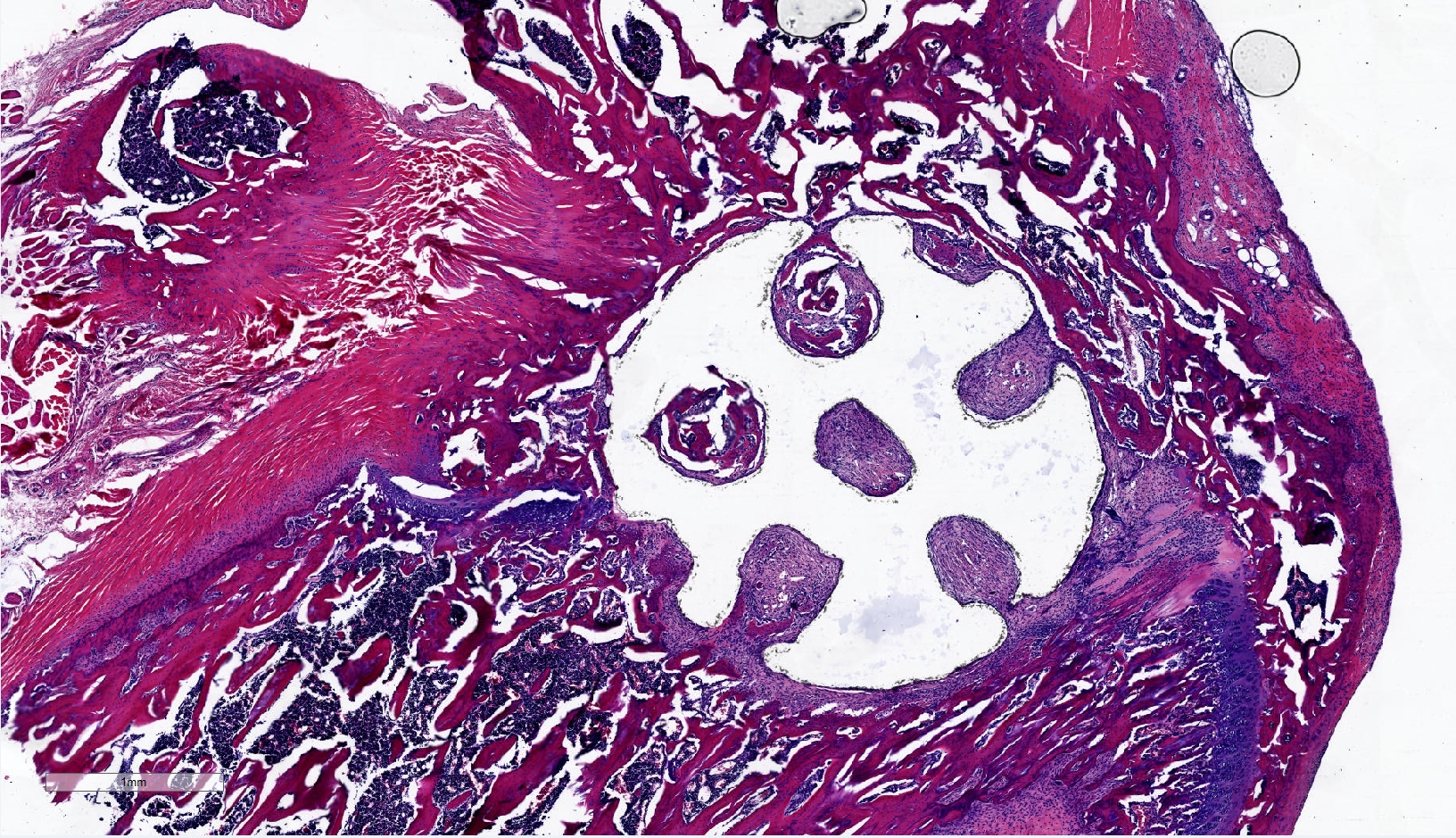

Supplement: Supplementary file 10 [file DataSheet7.zip › raw data_Figure 11A/MS 4W.png]

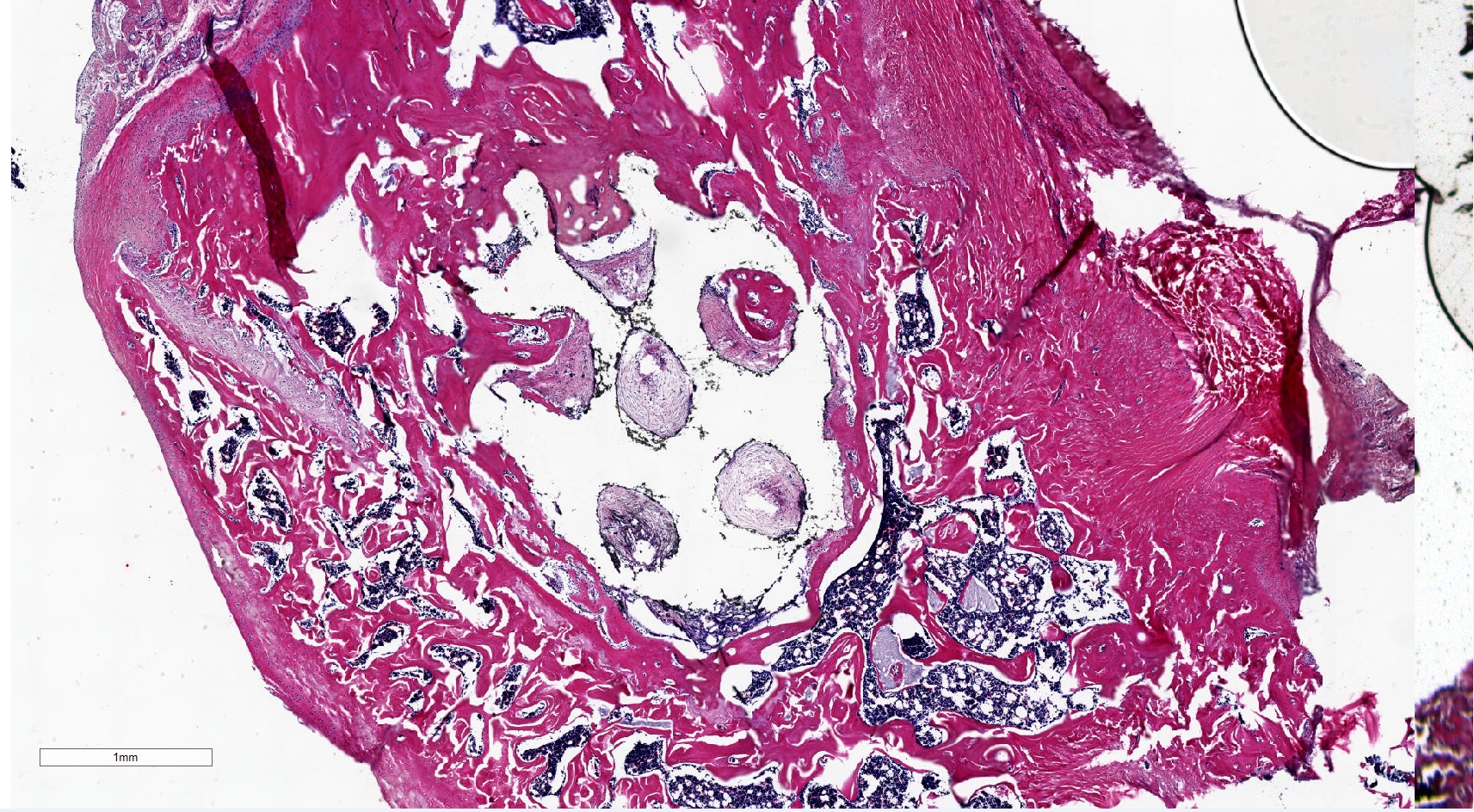

Supplement: Supplementary file 10 [file DataSheet7.zip › raw data_Figure 11A/MS 8W.png]

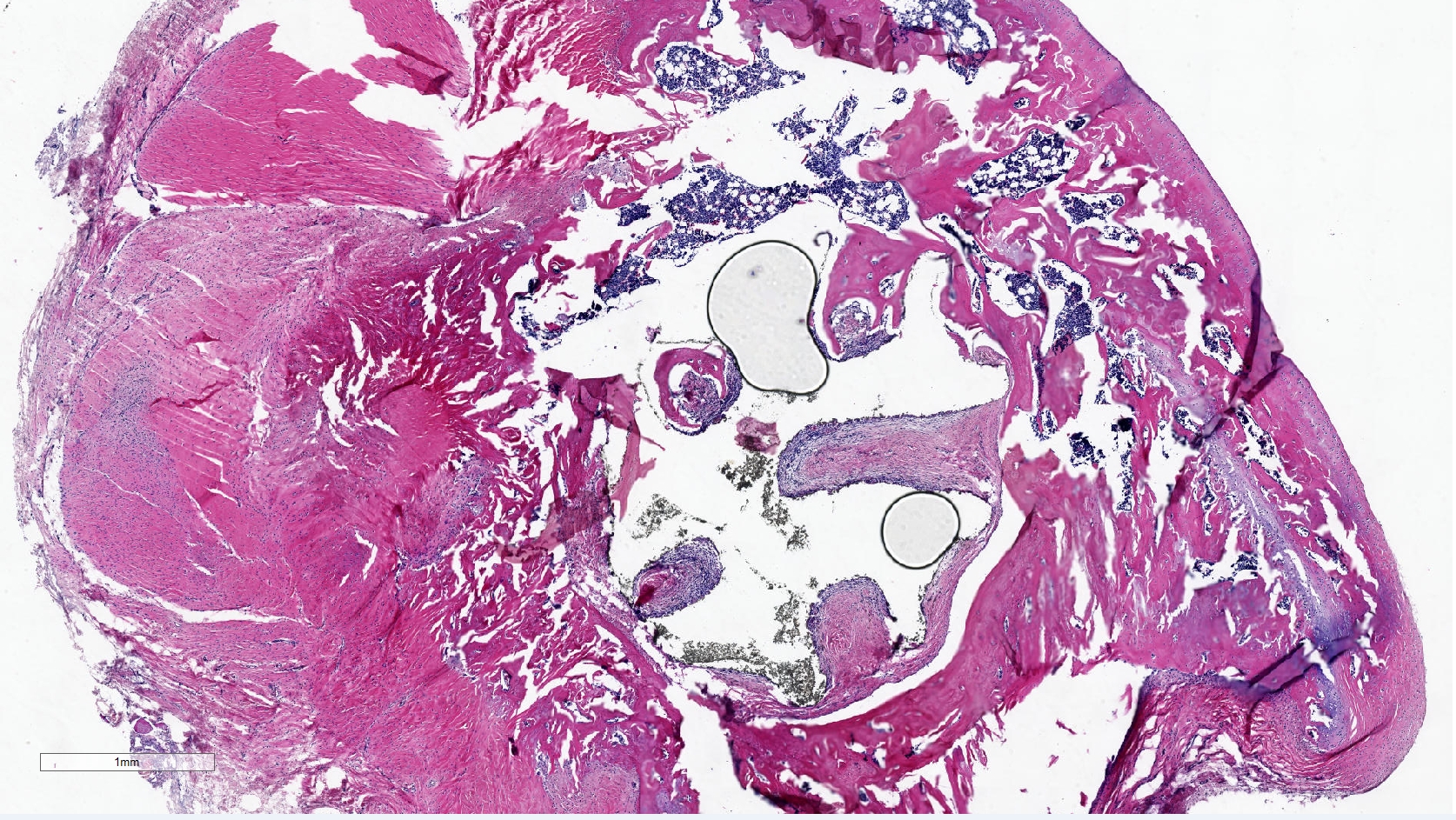

Supplement: Supplementary file 10 [file DataSheet7.zip › raw data_Figure 11A/TCP 12w.png]

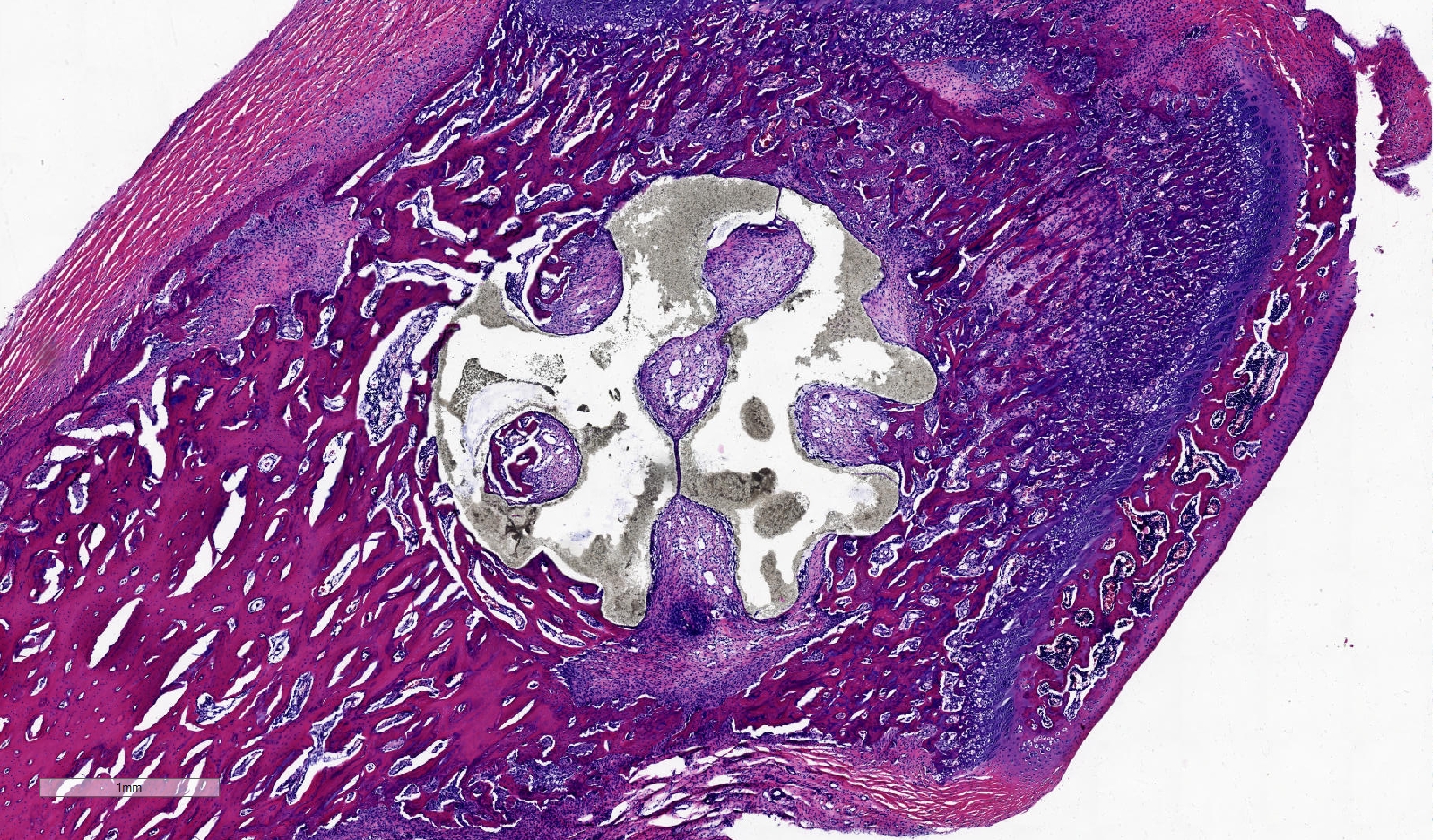

Supplement: Supplementary file 10 [file DataSheet7.zip › raw data_Figure 11A/TCP 4W.png]

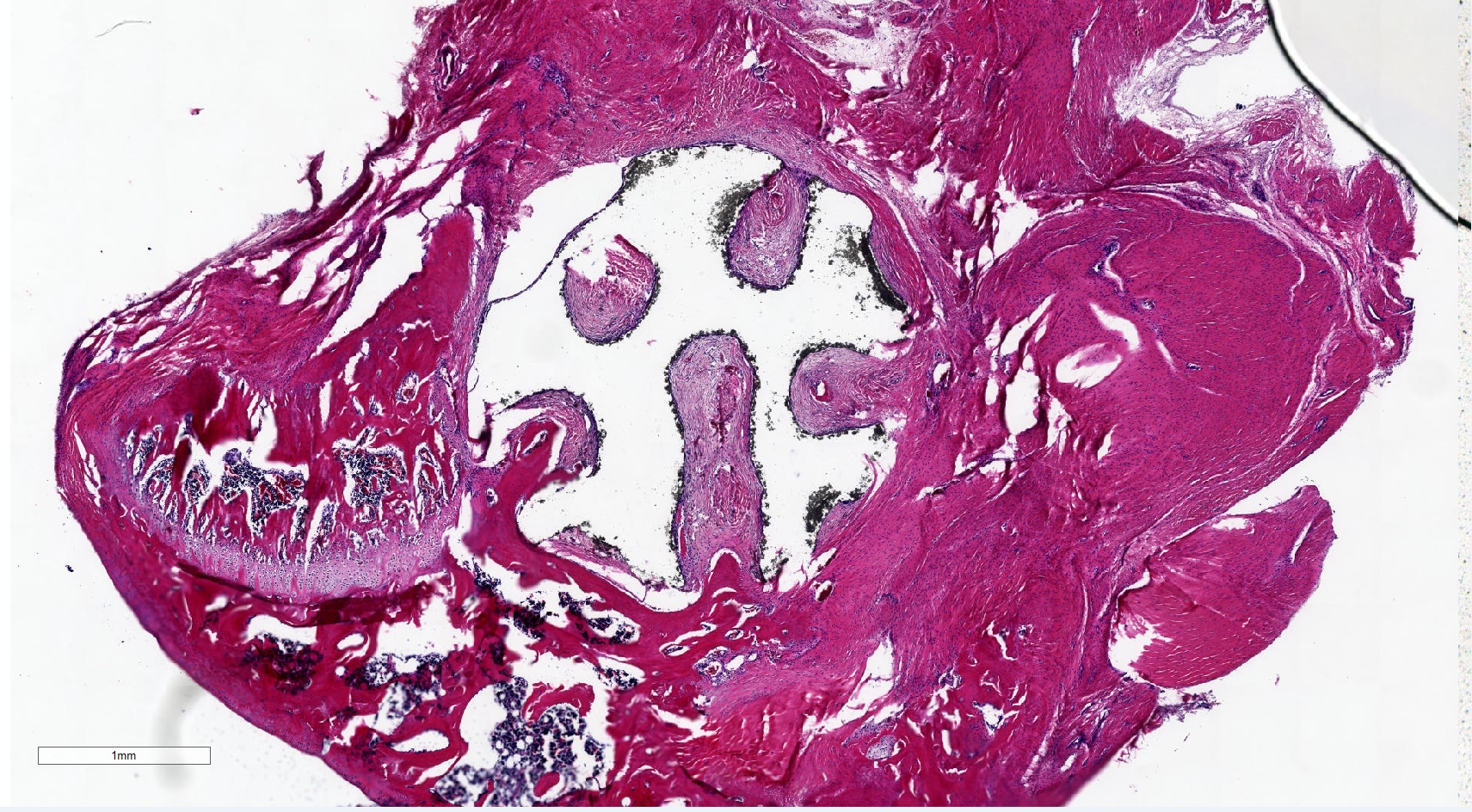

Supplement: Supplementary file 10 [file DataSheet7.zip › raw data_Figure 11A/TCP 8W.png]
